# Synthesis, Structure, and Photophysical Properties of Platinum(II) (*N*,*C*,*N*′) Pincer Complexes Derived from Purine Nucleobases†

**DOI:** 10.1021/acs.inorgchem.3c00650

**Published:** 2023-05-18

**Authors:** Carmen Lorenzo-Aparicio, Sonia Moreno-Blázquez, Montserrat Oliván, Miguel A. Esteruelas, Mar Gómez Gallego, Pablo García-Álvarez, Javier A. Cabeza, Miguel A. Sierra

**Affiliations:** ‡ Departamento de Química Orgánica, Facultad de Ciencias Químicas, Universidad Complutense, 28040 Madrid, Spain; § Departamento de Química Inorgánica, Instituto de Síntesis Química y Catálisis Homogénea (ISQCH), Universidad de Zaragoza-CSIC, 50009 Zaragoza, Spain; ∥ Departamento de Química Orgánica e Inorgánica, Facultad de Química, Universidad de Oviedo, 33071 Oviedo, Spain; ⊥ Center for Innovation in Advanced Chemistry (ORFEO-CINQA), https://orfeocinqa.es/

## Abstract

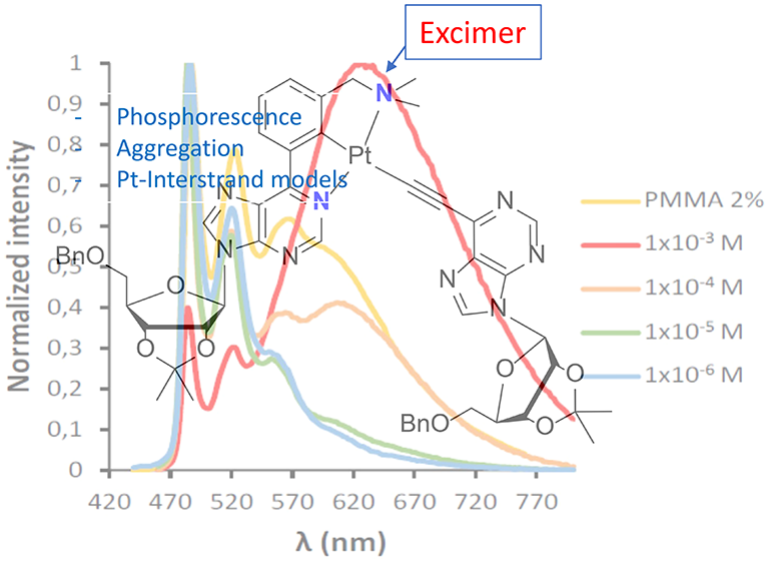

The synthesis of a series of Pt{κ^3^-*N*,*C*,*N*′-[L]}X (X = Cl, RC≡C) pincer complexes derived from purine and purine nucleosides is reported. In these complexes, the 6-phenylpurine skeleton provides the *N*,*C*-cyclometalated fragment, whereas an amine, imine, or pyridine substituent of the phenyl ring supplies the additional *N*′-coordination point to the pincer complex. The purine *N*,*C*-fragment has two coordination positions with the metal (*N*1 and *N*7), but the formation of the platinum complexes is totally regioselective. Coordination through the *N*7 position leads to the thermodynamically favored [6.5]-Pt{κ^3^-*N*7,*C*,*N*′-[L]}X complexes. However, the coordination through the *N*1 position is preferred by the amino derivatives, leading to the isomeric kinetic [5.5]-Pt{κ^3^-*N*1,*C*,*N*′-[L]}X complexes. Extension of the reported methodology to complexes having both pincer and acetylide ligands derived from nucleosides allows the preparation of novel heteroleptic bis-nucleoside compounds that could be regarded as organometallic models of Pt-induced interstrand cross-link. Complexes having amine or pyridine arms are green phosphorescence emitters upon photoexcitation at low concentrations in CH_2_Cl_2_ solution and in poly(methyl methacrylate) (PMMA) films. They undergo self-quenching at high concentrations due to molecular aggregation. The presence of intermolecular π–π stacking and weak Pt···Pt interactions was also observed in the solid state by X-ray diffraction analysis.

## Abstract 2

The synthesis of a series of Pt{κ^3^-N,C,N′-[L]}X (X = Cl, RC≡C) pincer complexes derived from purine and purine nucleosides, is reported, and their structural and photophysical properties determined. These complexes undergo self-quenching at high concentrations, due to molecular aggregation and the presence of intermolecular π-π stacking and weak Pt···Pt interactions were observed in the solid state by X-ray diffraction analysis.

## Introduction

The coordination of DNA fragments to metals is of fundamental importance in many bioinorganic processes.^1^ Most of the reported studies in this field focused on the interaction of platinum complexes with DNA and nucleobases, very likely because most of the current antitumor drugs for clinical use are based on platinum complexes.^2−5^ The high toxicity of these compounds due to concomitant DNA damage^6^ keeps alive the interest for studies that combine Pt and nucleobases to the development of new models of interaction. In this regard, platinum complexes bearing tridentate ligands have attracted particular interest, as they can bind to and intercalate DNA,^7^ trigger the formation of G-quadruplexes,^8,9^ cause interstrand cross-links (ICL), and generate extensive conformational alterations.^10^ The luminescent properties associated with many of these complexes^11−14^ have helped the study of interaction models^15,16^ and have also been used to investigate intracellular processes *in vivo.*(17)

Our research group is a pioneer in the development of methodologies to prepare cyclometalated transition metal complexes [M = Ir(III), Rh(III), Os(IV)] derived from nucleobases, nucleosides, and nucleotides.^18−21^ In these studies, purine derivatives were excellent substrates to carry out cyclometalation reactions, and we reasoned that they could be interesting scaffolds to build cyclometalated platinum(II) (pincer) complexes. The idea was challenging, as purine nucleobase derivatives are highly functionalized systems with many positions prone to interact with the metal.

**Figure 1:**
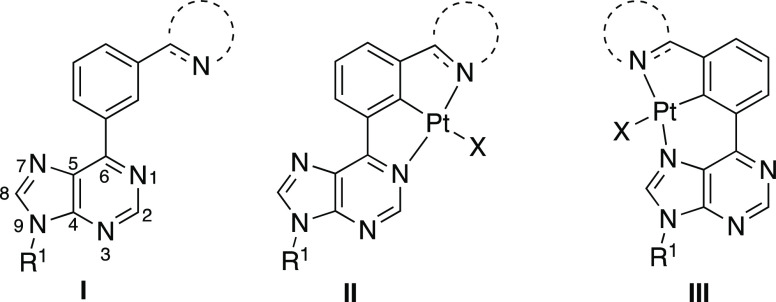
6-Phenylpurine pro-ligands (**I**) and the isomeric unsymmetrical *N*,*C*,*N*′-Pt(II) complexes (**II** and **III**).

Most of the reported tridentate cyclometalated platinum(II) complexes are derived from symmetrical ligands. This is remarkable, as unsymmetrical *N*,*C*,*N*′*-*pincer ligands would offer a great opportunity to tune the properties of the platinum complex by combining the steric and electronic characteristics of the donor N and N′ atoms. In our approach, the 6-phenylpurine skeleton would provide the rigid framework to build unsymmetrical *N*,*CH*,*N*′-pro-ligands **I** (Figure 1) by incorporation of the adequate N′-branches in the phenyl ring. Pro-ligands **I** offer two possible coordination modes to the metal since both *N*1 and *N*7 can bind to form isomers **II** and **III**, respectively. Our previous results showed that cyclometalation reactions of 6-phenylpurine derivatives promoted by group 8 and group 9 metal complexes exclusively involve the *N*1 atom of the nucleobase in the process of metallacycle formation.^19−21^ However, the coordination of *N*7 to many Pt(II) complexes is well known,^2−5^ whereas the participation of *N*7 in C-metalations has also been reported.^22^

The donor monodentate ligand (X) that occupies the fourth coordination position of isomers **II** and **III** will also be relevant for the chemical and photophysical properties of the complexes now reported. The trans effect of the cyclometalated carbon is behind the lability of the Pt–Cl bond in Pt{κ^3^-*N*,*C*,*N*′-[L]}Cl complexes, which will allow us to make further structural modifications by incorporation of diverse alkynyl ligands. Here, we describe the synthesis, reactivity, and the study of the photophysical properties of a new class of Pt{κ^3^-*N*,*C*,*N*′-[L]}X (X = Cl, RC≡C) complexes, derived from biomolecules: purine nucleobases and nucleosides. The methodology reported in this work is a step ahead in the design of a new class of photoluminescent complexes built on biocompatible moieties. Combination, in the metal coordination sphere, of a pincer containing a purine nucleoside arm together with purine nucleoside-substituted alkynyl ligands will allow us to furthermore generate complexes that can be viewed as organometallic interstrand cross-link models. In this context, it should be mentioned that interstrand cross-link is one of the most important pathways for DNA damage.^10^

## Results and Discussion

**Scheme 1:**
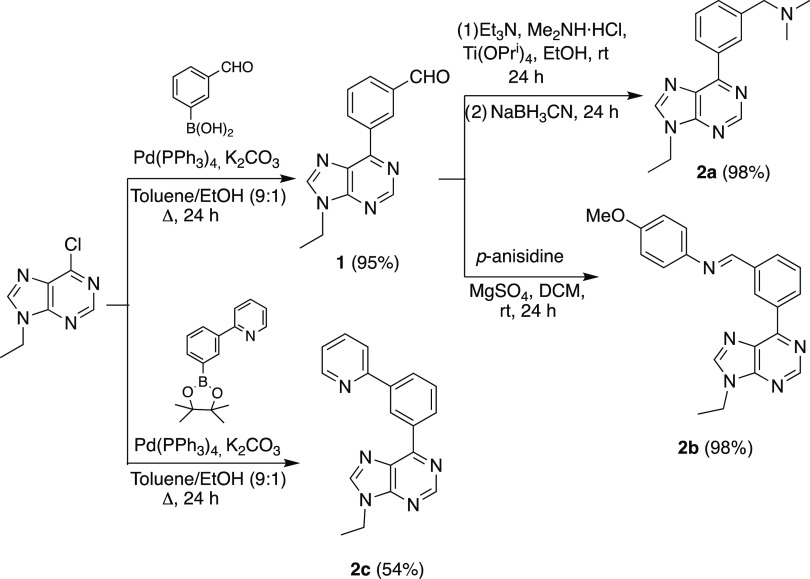
Synthesis of Pro-ligands **2a**–**c**

Scheme 1 summarizes the preparation of the *N*,*CH*,*N*′-pro-ligands **2a**–**c**. The synthesis of **2a** and **2b** was designed in a stepwise manner using a common precursor, aldehyde **1**. This compound was generated through a Suzuki coupling between 6-chloro-9-ethylpurine and 3-formylboronic acid, using Pd(PPh_3_)_4_ as a catalyst precursor and K_2_CO_3_ as a co-catalytic base. Amine **2a** was generated by reductive amination of **1**, with dimethylamine and NaBH_3_CN, using Ti(O*^i^*Pr)_4_ as a catalyst; while imine **2b** was made by the reaction of **1** with *p*-anisidine. In turn, pyridine derivative **2c** was synthesized in one step from 6-chloro-9-ethylpurine by Pd(PPh_3_)_4_/K_2_CO_3_-mediated Suzuki coupling with 3-(2-pyridynyl)phenylboronic acid pinacol ester.

**Scheme 2:**
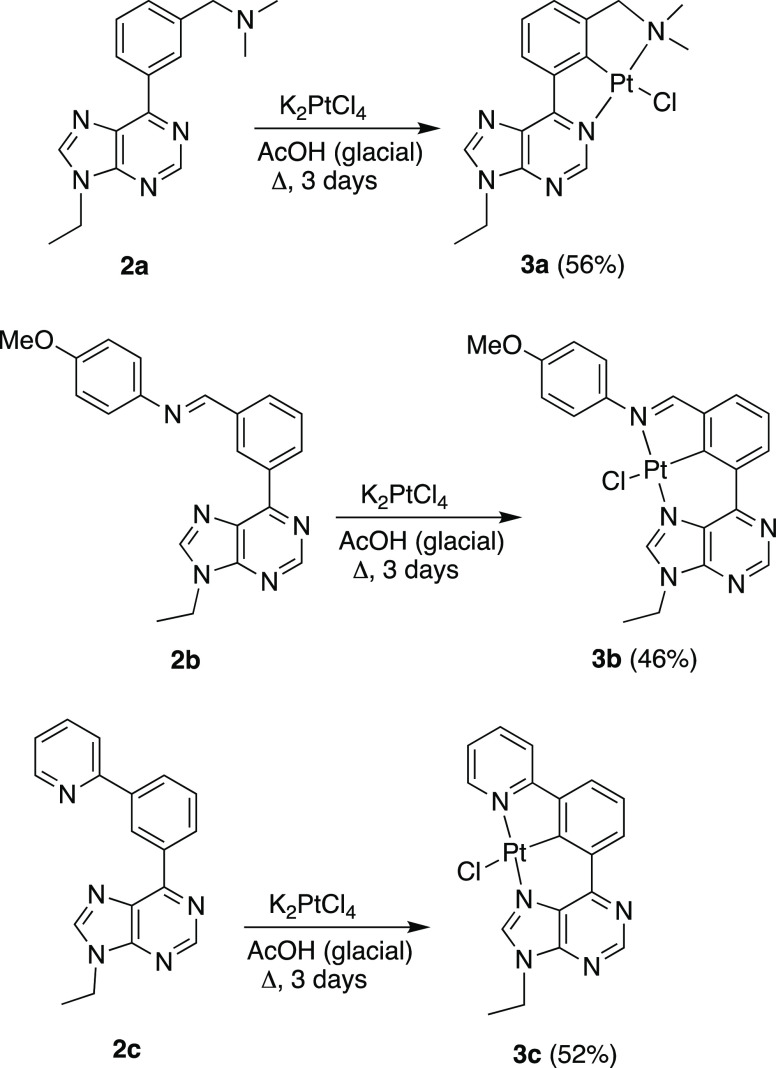
Synthesis of Complexes **3a**–**c**

Pro-ligands **2a**–**c** were subsequently used to prepare the respective chlorido-complexes Pt{κ^3^-*N*,*C*,*N*′-[L]}Cl: **3a**–**c**. The synthetic procedure involves the reaction of the salt K_2_PtCl_4_ with the organic molecules, in glacial acetic acid, under reflux (Scheme 2).^23^ Complexes **3a** and **3b** were obtained as pure products in 56 and 46% isolated yields, respectively, after chromatography of the reaction crude on silica gel. In contrast, complex **3c** was directly obtained (52% yield) by precipitation from the reaction medium with methanol and subsequent washing with methanol and diethyl ether. The three compounds were characterized by NMR spectroscopy and X-ray diffraction (XRD) analysis. The main bond distances and angles are given in Table S1.

**Figure 2:**
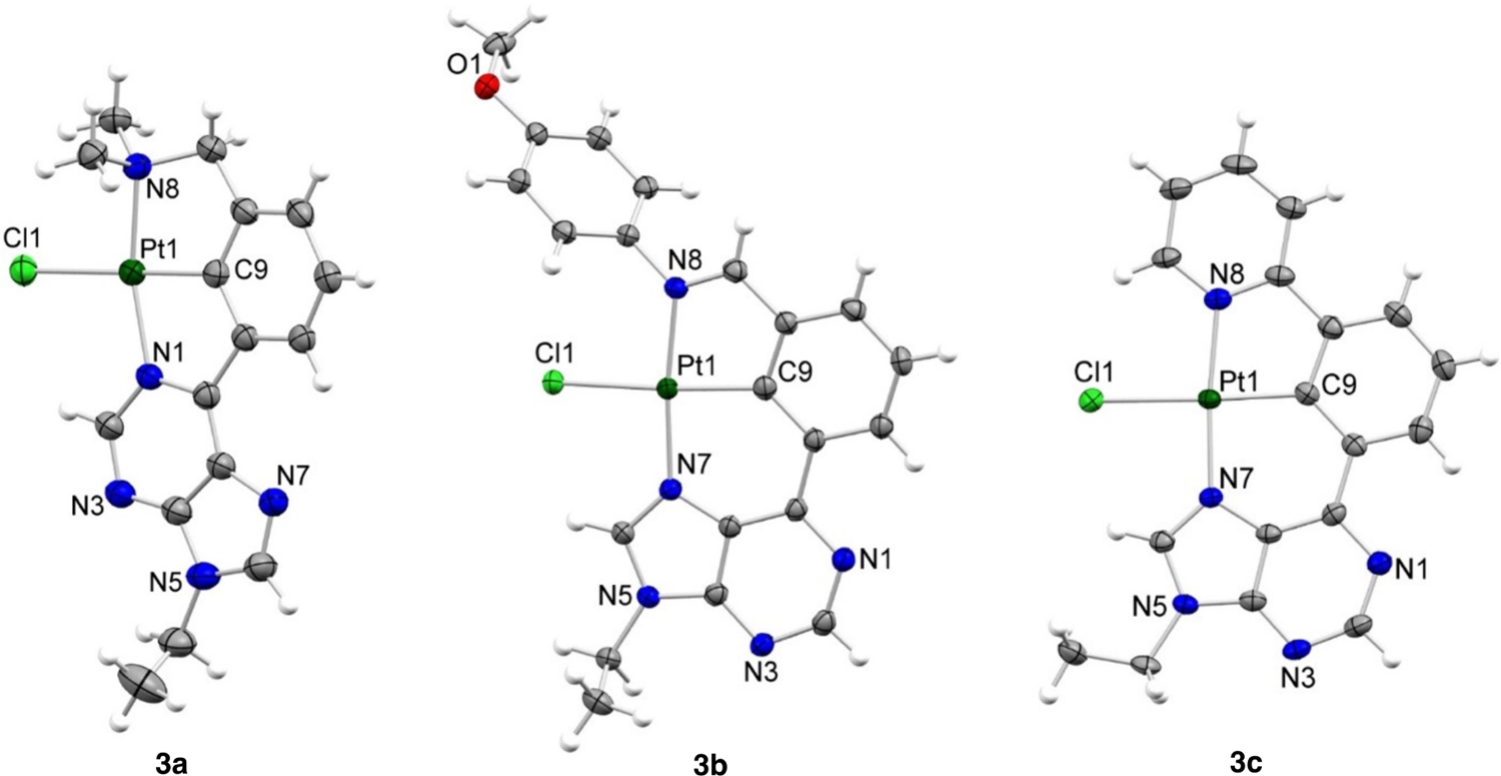
Molecular structures of complexes **3a**–**c** (30% displacement ellipsoids).

Figure 2 shows views of the molecules. The ligand environment around the platinum(II) center adopts the expected square-planar coordination, featuring a tridentate κ^3^*-N*,*C*,*N*′-ligand and a chloride anion trans to the metalated carbon atom. The atoms of the 6-phenylpurine scaffold are roughly coplanar in all cases. It should be pointed out that the purine arm coordinates the platinum atom by the *N*1 position in **3a**, while the coordination occurs through the *N*7 atom in **3b** and **3c**. In the first case, a five-membered heterometallacycle is generated, resulting in a [5.5]-Pt{κ^3^-*N*,*C*,*N*′-[L]}Cl bicycle derivative. By contrary, the *N*7 coordination gives rise to a six-membered heterometallacycle, which affords a [6.5]-Pt{κ^3^-*N*,*C*,*N*′-[L]}Cl bicycle. The N–Pt–N′ angles of complexes **3b** and **3c** are closer to the ideal value of 180° (average 173(1)°) than in **3a** (162.2(2)°) due to the strain imposed by the five-membered ring in the latter. All complexes show C9–Pt–Cl angles very close to 180° (average 176.8(5)°) and similar Pt–N, Pt–C, and Pt–Cl bond distances, which are in the range of those reported for related Pt{κ^3^-*N*,*C*,*N*-[L]}Cl complexes.^24^

**Figure 3:**
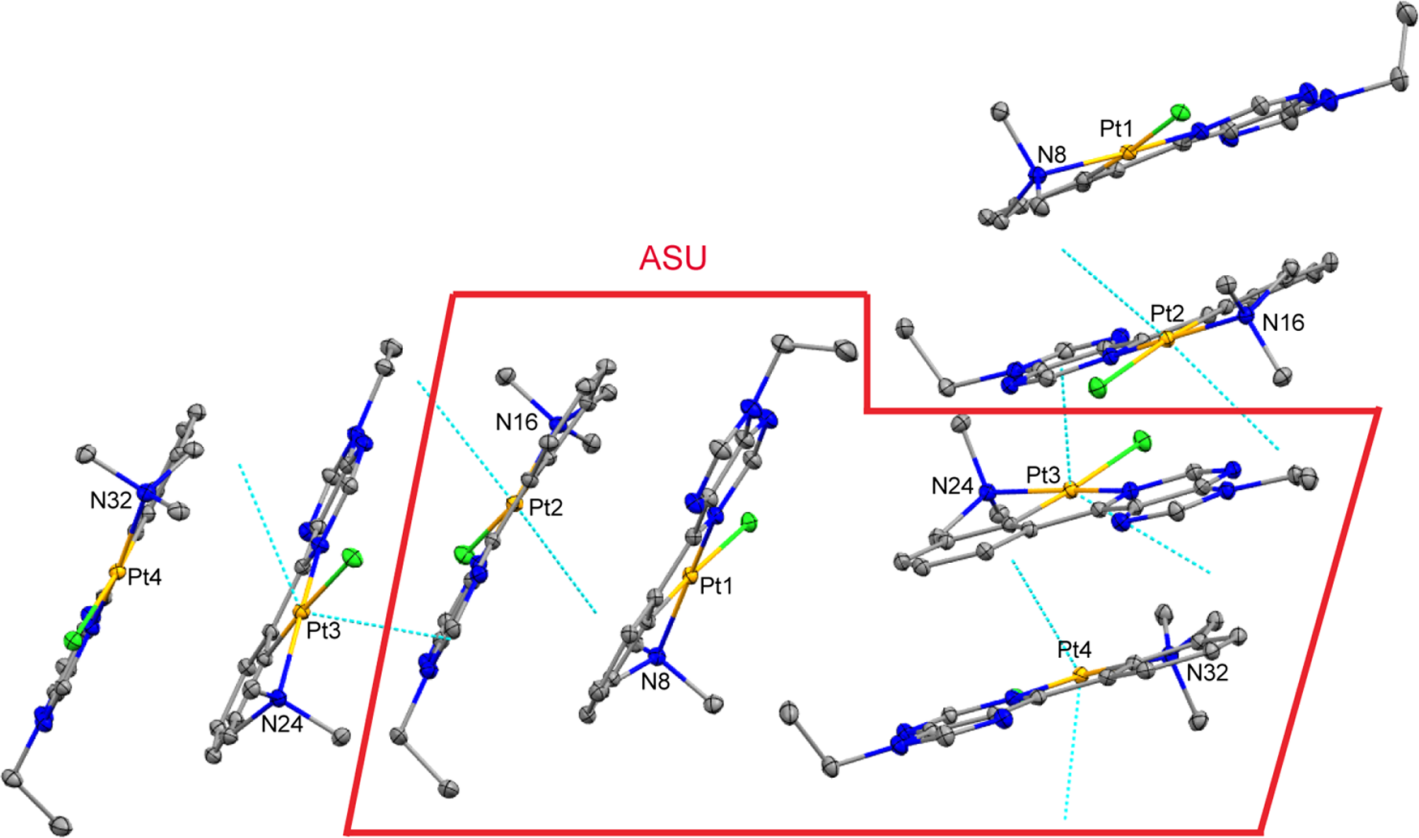
View of two groups of four molecules found in the crystal of **3a** (10% displacement ellipsoids), showing the approximate parallel disposition of the planes defined by the 6-phenylpurine scaffolds (separated by 3.3(3) Å (Pt1···Pt2), 3.4(3) Å (Pt3···Pt4), and 3.6(3) Å (Pt2···Pt3)). The shortest Pt···Pt distances are 3.835 Å (Pt1···Pt2) and 3.861 Å (Pt3···Pt4). The Pt2···Pt3 distance is 9.467 Å. The asymmetric unit (ASU) contains four molecules.

**Figure 4:**
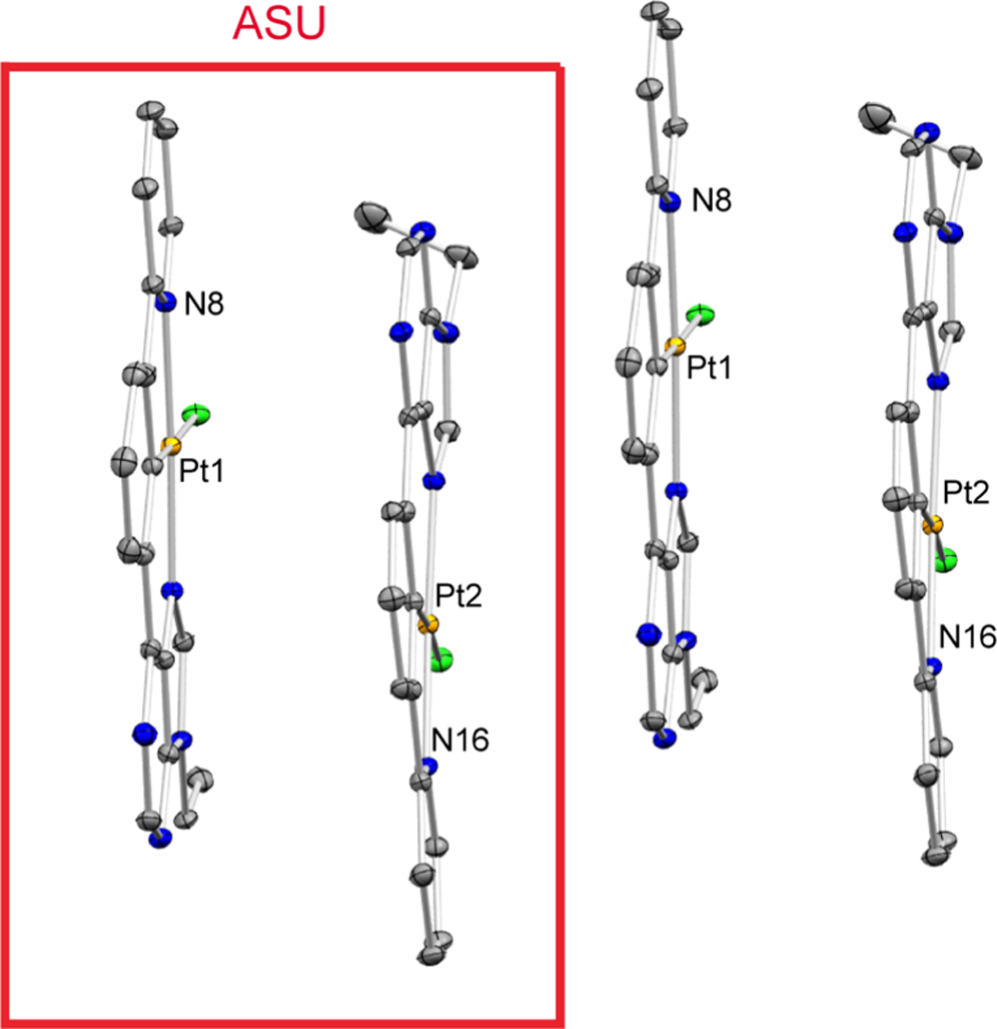
View of the molecular arrangement of **3c** in the crystal (10% displacement ellipsoids), showing the approximate parallel disposition of the planes defined by the 6-phenylpurine scaffolds [separated by 3.48(5) Å (within the asymmetric unit) and 3.38(5) Å (between molecules of different asymmetric units)]. This approximate parallel orientation is maintained along the entire crystal lattice. The shortest Pt···Pt distances are 4.279 Å (between the molecules of the asymmetric unit) and 5.141 Å (between molecules of different asymmetric units). The asymmetric unit (ASU) contains two molecules.

Inspection of the packing within the crystals of complexes **3a** (Figure 3), **3b** (Figure S1), and **3c** (Figures 4 and S2) reveals that the planes defined by the 6-phenylpurine scaffolds are close to each other (less than 4 Å) and arranged in an approximately or totally parallel manner, which points to the existence of intermolecular π–π stacking interactions. The asymmetric units contain four (two pairs in **3a**) or two close molecules (**3c**), which in the crystal generate stacks, with Pt···Pt distances of 3.835–3.861 Å for **3a** and 4.279 Å for **3c**; indicating the existence of significant metal–metal interactions (Figure 3).^25^ The asymmetric units of complex **3b** contain only one molecule (see the Supporting Information (SI)), and the closest intermolecular Pt···Pt distance in the crystal is 6.750 Å, longer than 2 × *r*_vdw_(Pt) = 4.6 Å,^26^ which rules out the existence of significant metal–metal interactions in this case.

The study of the ^1^H NMR spectra was congruent with the crystal structures. The coordination through the *N*7 position of the purine ring in complexes **3b** and **3c** was confirmed by the noticeable deshielding of the signals of purine *H*8, from 8.17 ppm in pro-ligands **2b** and **2c** to 9.29 (*J*_H–Pt_ = 11.2 Hz) and 9.37 ppm (*J*_H–Pt_ = 14.2 Hz) for **3b** and **3c**, respectively. However, in complex **3a**, the signal coupled to the neighboring platinum in the ^1^H NMR spectrum was that of purine *H*2 (9.40 ppm, *J*_H–Pt_ = 11.5 Hz), being also deshielded with regard to that of pro-ligand **2a** (8.98 ppm).

**Figure 5:**
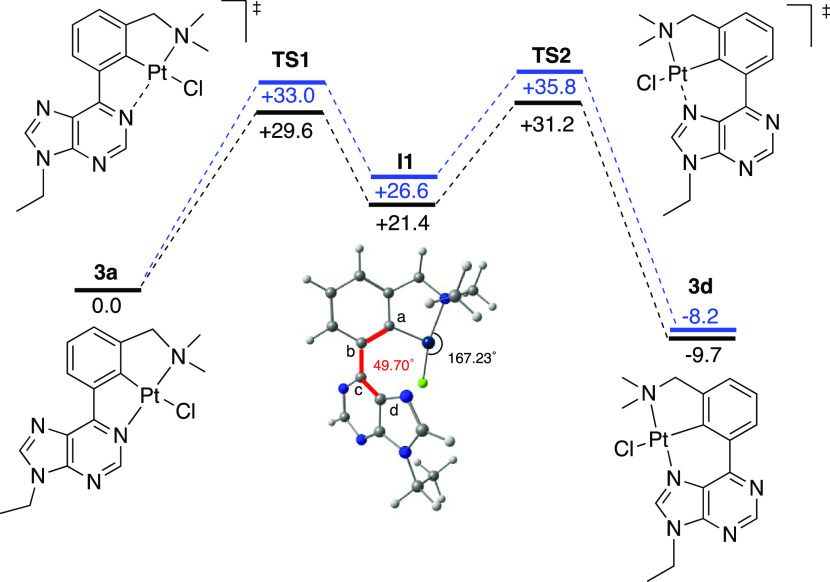
Energy profiles for the isomerization of **3a** to **3d** (B3LYP-D3/def2-SVP) in acetic acid (black) and toluene (blue). Relative energy values at 298 K (kcal mol^–1^).

The *N*1 coordination of the purine arm in **3a** is in line with our previous results in Ir(III)-, Rh(III)-, and Os(IV)-chemistry, which pointed out that only the *N*1 position of the purine ring was involved in the cyclometalation reactions of 6-phenylpurine derivatives, on complexes of these ions.^19,21^ To understand the difference in behavior between the pro-ligands, we analyzed the equilibrium between the isomers [5.5]-Pt{κ^3^-*N*,*C*,*N*′-[L]}Cl and [6.5]-Pt{κ^3^-*N*,*C*,*N*′-[L]}Cl by density functional theory (DFT) calculations (B3LYP-D3/def2-SVP) (Figures S7–S9). The first analysis of the computed data revealed that the isomer [6.5]-Pt{κ^3^-*N*,*C*,*N*′-[L]}Cl is more stable than the [5.5]-Pt{κ^3^-*N*,*C*,*N*′-[L]}Cl, in all cases. That is, the *N*7 coordination of the purine arm is thermodynamically favored over the coordination through *N*1 (between 9.01 and 10.38 kcal mol^–1^). In consequence, complex **3a** must be regarded as the kinetic product of the reaction of **2a** with the platinum salt. A more detailed DFT analysis showed that the transformation **3a** into the more stable isomer, [6.5]-Pt{κ^3^-*N*,*C*,*N*′-[L]}Cl, **3d** takes place in two stages (Figure 5), via the three-coordinate intermediate **I1** (dihedral angle Ca–Cb–Cc–Cd 49.70°). The first stage involves the rupture of the *N*1–Pt bond via the transition state **TS1**, whereas the formation of the *N*7–Pt bond occurs in the second step through the transition state **TS2**. The activation energies of both steps are 29.6 and 31.2 kcal mol^–1^, respectively, considering acetic acid as the solvent. These barriers increase up to 33.0 and 35.8 kcal mol^–1^ in toluene.

**Scheme 3:**
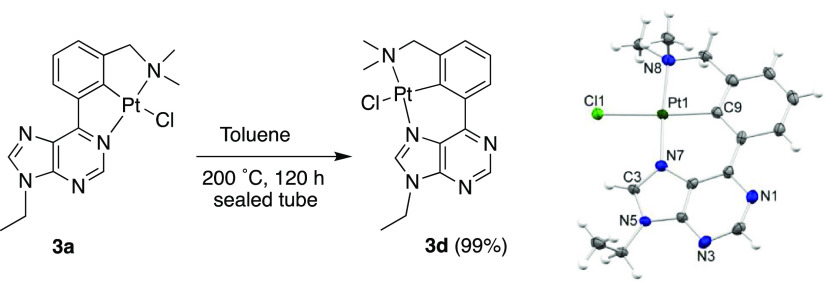
Isomerization of **3a** to **3d** and Molecular Structure of **3d** (30% Displacement Ellipsoids) Only one of the two analogous molecules found in the asymmetric unit is shown.

Based on these studies, we first tested the isomerization **3a** to **3d** in acetic acid, at 200 °C, in a sealed tube. Unfortunately, complete decomposition to a black solid occurred. Thence, we tried the process in toluene. To our delight, this time, the clean and quantitative transformation of **3a** into the more stable species **3d** took place after 120 h (Scheme 3). In agreement with **3b** and **3c**, the ^1^H NMR spectrum of **3d** showed a clean singlet at 8.99 ppm due to H2, while the signal corresponding to H8 showed platinum satellites (^3^*J*(^1^H–^195^Pt) = 10.5 Hz). The formation of **3d** was confirmed by XRD. Inspection of the crystal packing did not reveal significant Pt···Pt interactions in this case (Scheme 3 and Figure S3).

**Scheme 4:**
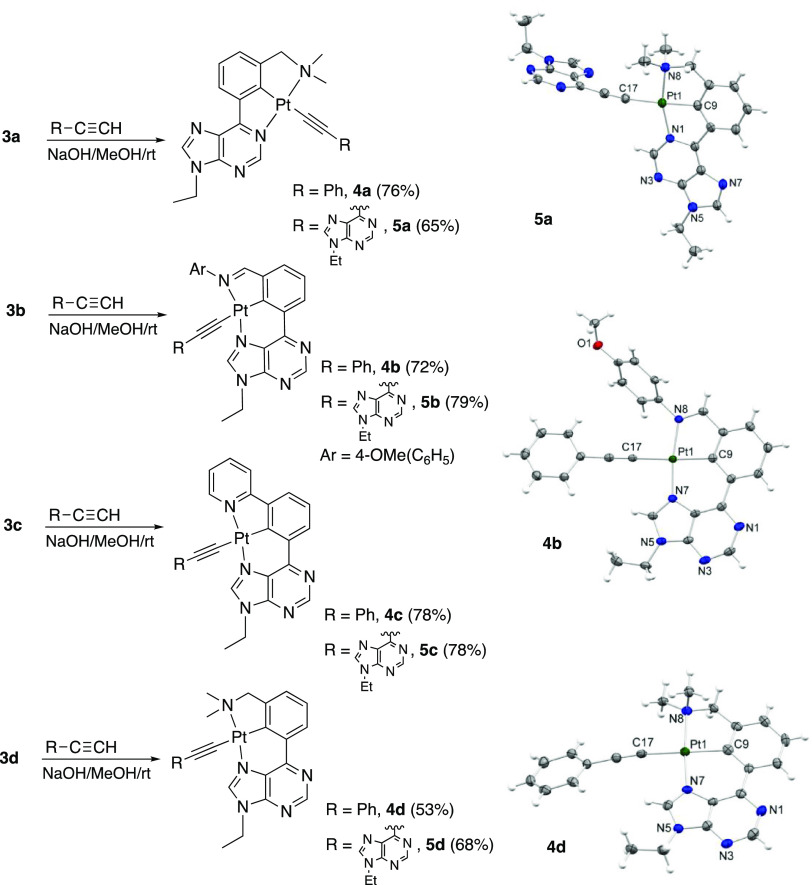
Synthesis of the Alkynyl Derivatives **4a–d** and **5a**–**d** and Molecular Structures of **5a**, **4b**, and **4d** (30% Displacement Ellipsoids)

The chloride ligand of complexes **3a**–**d** was further replaced by acetylide ligands. Reaction with phenylacetylene, in the presence of NaOH, at room temperature (rt), in methanol led to acetylido derivatives **4a**–**d**, which were isolated as yellow-orange solids in 65–79% yields by precipitation in the reaction media and subsequent washing with cold methanol and diethyl ether (Scheme 4). Extension of this methodology to more sensitive acetylide ligands was tested by the reaction of **3a**–**d** with freshly prepared 6-ethynyl-9-ethylpurine. In this case, complexes **5a**–**d** were also obtained in high yields (65–80%) as yellow solids (Scheme 4).

Acetylido derivatives were characterized by NMR and mass spectrometry and, in the case of **4b**, **4d**, and **5a**, by XRD (Scheme 4 and Figures S4–S6). The C≡C bond distances of 1.202(9), 1.190(5), and 1.20(2) Å, respectively, compare well with those reported for the compounds of this class previously characterized by XRD.^27^ This fact is indicative of the lack of significant π-back-bonding from the Pt atoms to the alkynyl ligands.

**Scheme 5:**
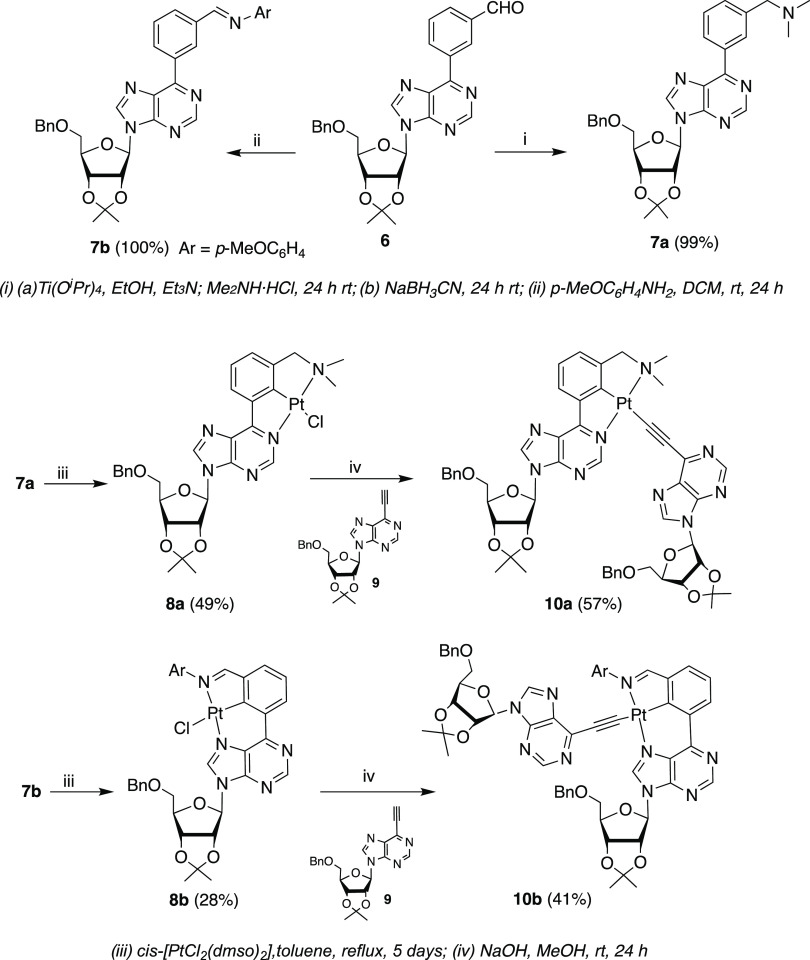
Synthesis of Acetylido Complexes **10a** and **10b**

To go a step ahead, we explored this methodology to prepare both types of [5.5]- and [6.5]-Pt{κ^3^-*N*,*C*,*N*′-[L]}X (X = Cl, RC≡C) pincer complexes in purine nucleosides as ligand precursors (Scheme 5). Pro-ligands **7a** and **7b** were obtained in quantitative yields from aldehyde **6** (see the SI) using the reaction conditions previously employed for the preparation of **3a** and **3b**. However, the synthesis of the respective chlorido compounds [5.5]-Pt{κ^3^-*N*,*C*,*N*′-[L]}Cl could not be achieved by the reaction of **7a** and **7b** with K_2_PtCl_4_, in glacial acetic acid, under reflux, as these harsh conditions caused the decomposition of the pro-ligands. Chlorido derivatives **8a** and **8b** were successfully prepared by refluxing **7a** and **7b** with [PtCl_2_(DMSO)_2_] (DMSO = dimethyl sulfoxide) in toluene for 48 h, in 49 and 28% yields, after chromatography on silica gel. Further, the reaction of **8a** and **8b** with freshly prepared ethynylpurine nucleoside **9** (see SI) in a NaOH solution in methanol afforded heteroleptic bis-nucleoside compounds **10a** and **10b** in 57 and 41% yields, respectively. Complexes **10a** and **10b** join two purine nucleosides in their structures through the alkynyl–Pt complex and could be regarded as simple organometallic models of interstrand cross-link (ICL) in oligonucleotides.^10^

### Photophysical Properties of Emissive Complexes

The square-planar platinum(II) *d*^8^-complexes are considered one of the noble families of phosphorescent emitters.^11−14,28^ This fact, along with the novelty of the purine–pincer–platinum(II) skeleton prompted us to study the absorption and emission characteristics of the emissive compounds prepared, which were those of the **3**, **4**, and **5**-types bearing an amine- or pyridine arm (imine derivatives were not emissive).

**Figure 6:**
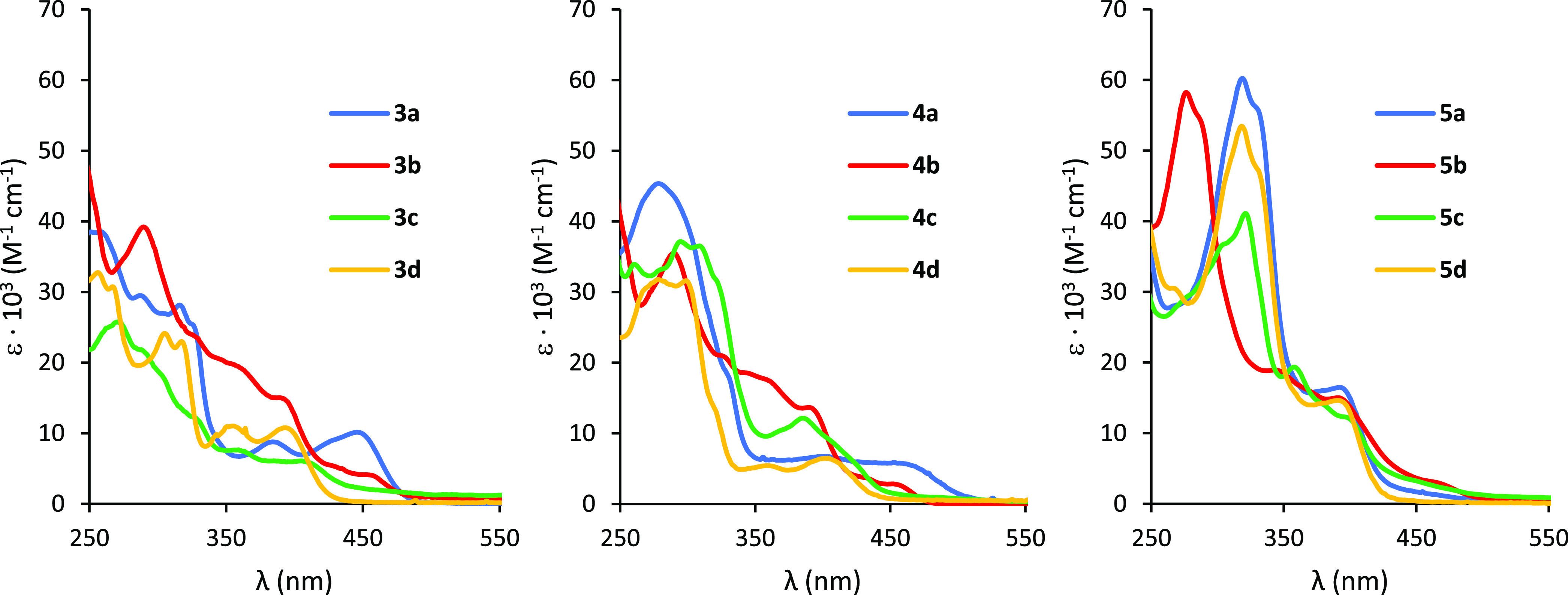
UV–vis spectra of complexes **3a**–**d** (left), **4a**–**d** (center), and **5a**–**d** (right) in CH_2_Cl_2_ (10^–5^ M).

The UV–vis spectra of complexes **3**, **4**, and **5** (10^–5^ M, in dichloromethane (DCM), at room temperature) are depicted in Figure 6, and the selected absorptions (assigned by time-dependent density functional theory calculations (TD-DFT-B3LYP-D3/def2-SVP) in dichloromethane) are shown in Tables S3–S6. The frontier molecular orbitals of complexes **3**–**5** are provided in Figures S10–S14. The lowest unoccupied molecular orbitals (LUMOs) are very similar, mainly localized on the cyclometalating (*N*,*C*) fragments of the pincer ligand. For the chloride complexes **3a–d**, the highest occupied molecular orbitals (HOMOs) are composed of the Pt and halide centers with some contribution of the phenyl moiety. In turn, the HOMOs of the acetylide derivatives **4a**–**d** and **5a**–**d** are mainly formed by the contribution of the aryl acetylide ligand and the Pt centers. All compounds display intense bands in the region 270–330 nm, with extinction coefficients (ε) of about 10^4^ dm^3^ mol^–1^ cm^–1^, which can be assigned to the intraligand IL [π–π*] transition of the pincer ligand, mixed with metal to ligand charge transfer (MLCT) [dπ(Pt) to π*(*N*,*C*,*N*′)] transitions. In addition, moderately intense absorption bands were observed at about 390–450 nm with extinction coefficients on the order of 10^3^–10^4^ dm^3^ mol^–1^ cm^–1^. In the case of the halide derivatives **3a**–**d**, these absorptions are ascribed to IL [π–π*] mixed with MLCT [dπ(Pt) to π*(*N*,*C*,*N*′)] transitions. For acetylide complexes **4a**–**d** and **5a**–**d**, those lower energy absorptions are ascribed to LLCT [π(phenylacetylide) to π*(*N*,*C*,*N*′)] or [π(9-ethylpurineacetylide) to π*(*N*,*C*,*N*′)] transitions. The very weak bands at lower energy, about 470–550 nm, are attributed to the direct population of the triplet π–π* state facilitated by the high spin–orbit coupling associated with the Pt(II) ion. There is a good agreement between the computed selected transitions and the experimental absorption maxima.

Emissions take place upon photoexcitation and occur in the green region of the spectrum (476–571 nm). Measurements were performed in doped poly(methyl methacrylate) films at 5 and 2 wt % (PMMA_5%_ and PMMA_2%_) at 298 K and in dichloromethane (CH_2_Cl_2_) and 2-methyltetrahydrofuran (2-MeTHF) at 298 and at 77 K. Table 1 summarizes the main features of the emissions, which occur from the respective T_1_ excited states as supported by the excellent agreement observed between the maximum of the emission wavelengths in CH_2_Cl_2_ and the calculated values in the same solvent for the differences in energy between the optimized triplet states T_1_ and the singlet states S_0_. According to the spin density distribution calculated for the T_1_ states in their minimum energy geometries (Table S8 and Figures S102–S110), the emissions appear to have a mixed MLTC/LC/LLCT character in all cases. Consistently, the bands are highly structured.

**Table 1:** Selected Emission Data of Complexes **3**, **4**, and **5**^a^^,^^b^

complex	medium (*T*/K)	λ_em(calc)_ (nm)^c^	λ_em_ (nm)	τ (μs)^d^	Φ^e^ (%)
**3a**	PMMA_5%_ (298)	539	**530**, 566	0.2 (4.2%), 4.0 (95.8%)	36
	CH_2_Cl_2_ (298)		**530**, 562	10.1	62
	CH_2_Cl_2_ (77)		**520**, 560, 696 (exc)	12.7 (45.5%), 5.1 (54.5%)	
	2-MeTHF (298)		**533**, 570	3.3	25
	2-MeTHF (77)		479, **523**, 559	11.5	
**3c**	PMMA_5%_ (298)	476	487, **524**, 550(sh)	3.3 (14.1%), 9.7 (85.9%)	28
	CH_2_Cl_2_ (298)		**484**, 516, 548	5.0	20
	CH_2_Cl_2_ (77)		486, **526**, 556, 644 (exc)	15.4 (83.4%), 36.0 (16.6%)	
	2-MeTHF (298)		**486**, 522, 556	7.3	20
	2-MeTHF (77)		**480**, 506, 518, 548	24.9 (14.9%), 14.4 (85.1%)	
**3d**	PMMA_5%_ (298)	488	**488**, 521, 558	10.5	70
	CH_2_Cl_2_ (298)		**487**, 521, 559	11.9	16
	CH_2_Cl_2_ (77)		**486**, 524, 559	34.7 (53.5%), 17.4 (46.5%)	
	2-MeTHF (298)		**488**, 522, 560	20.1 (90.4%), 2.6 (9.6%)	35
	2-MeTHF (77)		**479**, 515, 551	36.2 (93.0%), 10.1 (7.0%)	
**4a**	PMMA_2%_ (298)	545	**529**, 559	0.5 (1.7%), 5.1 (98.3%)	22
	CH_2_Cl_2_ (298)		**530**, 560	10.2	23
	CH_2_Cl_2_ (77)		**521**, 559, 688 (exc)	46.0 (12.8%), 15.1 (87.2%)	
	2-MeTHF (298)		499, **531**, 567 (sh)	3.3 (89.8%), 0.3 (10.2%)	6
	2-MeTHF (77)		480, 490, **518**, 556	12.8	
**4c**	PMMA_2%_ (298)	487	**490**, 524, 563(sh), 622 (exc)	4.2 (26.0%), 9.4 (74.0%)	26
	CH_2_Cl_2_ (298)		**488**, 522, 556	5.4	21
	CH_2_Cl_2_ (77)		**490**, 526, 568	21.0 (82.3%), 11.5 (17.7%)	
	2-MeTHF (298)		**486**, 520, 550	6.8	18
	2-MeTHF (77)		**480**, 516, 550	20.3 (41.1%), 13.3 (58.9%)	
**4d**	PMMA_5%_ (298)	491	**492**, 521, 563(sh)	7.7	61
	CH_2_Cl_2_ (298)		493, **524**, 562(sh)	11.0 (17.6%), 2.2 (82.4%)	7
	CH_2_Cl_2_ (77)		489, **520**, 559 (sh)	21.3 (29.8%), 6.9 (70.2%)	
	2-MeTHF (298)		**488**, 523, 559 (sh)	16.5 (19.7%), 10.0 (80.3%)	10
	2-MeTHF (77)		**478**, 514, 550	36.8 (86.5%), 18.0 (13.5%)	
**5a**	PMMA_5%_ (298)	524	**484**, 517, 556(sh)	6.0	50
	CH_2_Cl_2_ (298)		**482**, 515, 554(sh)	2.4	12
	CH_2_Cl_2_ (77)		**482**, 516, 555 (sh)	23.5 (44.1%), 10.4 (55.9%)	
	2-MeTHF (298)		**488**, 522, 559	4.7	12
	2-MeTHF (77)		**476**, 507, 546	19.5	
**5c**	PMMA_2%_ (298)	477	**487**, 524, 568, 609 (exc)	4.4 (16.1%), 12.1 (83.9%)	50
	CH_2_Cl_2_ (298)		**484**, 520, 558	7.1	23
	CH_2_Cl_2_ (77)		**488**, 526, 608 (exc)	27.6 (26.9%), 12.4 (73.1%)	
	2-MeTHF (298)		**486**, 522, 558	6.4	15
	2-MeTHF (77)		**480**, 508, 518, 552	30.3 (5.6%), 14.0 (94.4%)	
**5d**	PMMA_5%_ (298)	447	**484**, 516, 553	7.8	48
	CH_2_Cl_2_ (298)		**484**, 517, 555	11.7 (47.9%), 3.3 (52.1%)	5
	CH_2_Cl_2_ (77)		**482**, 517, 555 (sh)	32.0 (46.9%), 15.0 (53.1%)	
	2-MeTHF (298)		**483**, 517, 554	11.3 (23.1%), 4.1 (76.9%)	9
	2-MeTHF (77)		**478**, 511, 548 (sh)	53.3 (10.4%), 25.8 (89.6%)	

a Table 1 summarizes the data in Table S7.

b Solutions 1 × 10^–5^ M. The most intense peak is in bold. (exc) λ_em_ excimer.

c Computed values (SMD(CH_2_Cl_2_)-B3LYP-D3/def2-SVP) obtained from the differences in energy between the optimized triplet states T_1_ and the singlet states S_0_.

d Relative amplitudes (%) are given in parentheses for biexponential decays.

e Absolute quantum yield.

Amine kinetic isomers **3a**, **4a**, and **5a** display moderated quantum yields in PMMA_5%_ (0.36–0.50). The values significantly increase for the thermodynamic counterparts, which lie in the range 0.70–0.48 and decrease in the sequence **3d** > **4d** > **5d**. Two factors play in favor of the thermodynamic isomers. A comparison of the molecular packing for **3a** and **3d** reveals that the aggregation is higher in the kinetic isomers and is known to favor self-quenching.^29^ In addition, increasing emission efficiency with emitter stability is a common effect, which has also been previously observed for *N*,*C*,*N*-pincer emitters of osmium(IV) and iridium(III). The rise in stability is ascribed to the approach of the pincer bite angles to the ideal values corresponding to the coordination polyhedron of the complex.^30^

**Figure 7:**
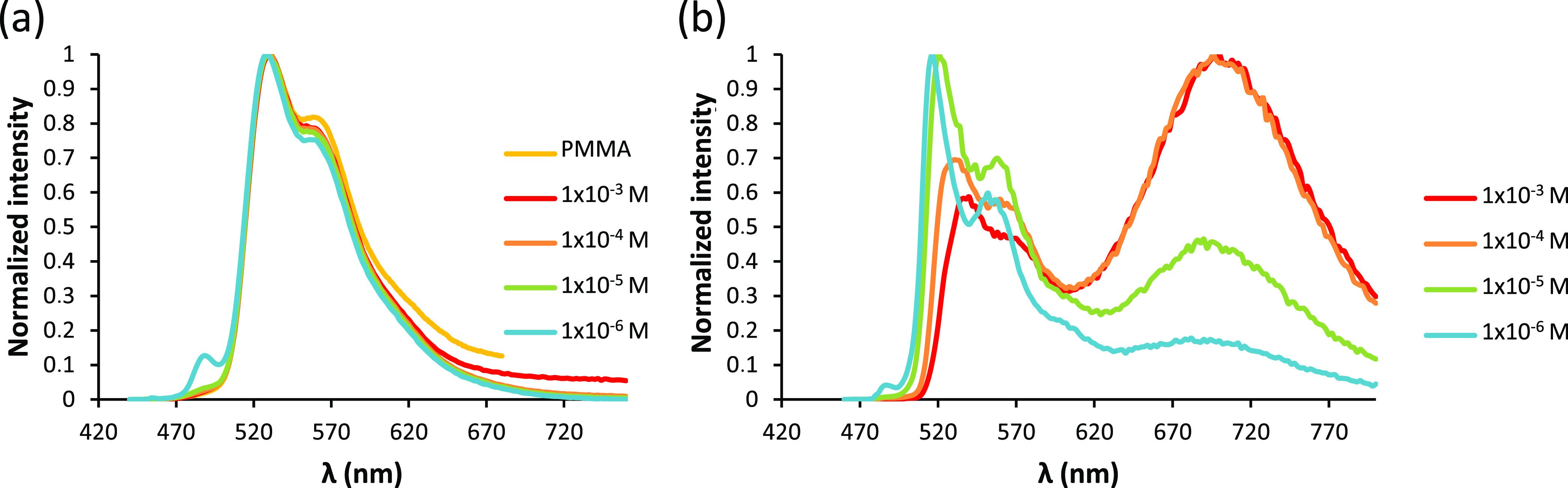
(a) Emission spectra of **3a** at 298 K in PMMA_5%_ and in dichloromethane at concentrations between 1 × 10^–3^ and 1 × 10^–6^ M. (b) Emission spectra of **3a** at 77 K in dichloromethane at concentrations between 1 × 10^–3^ and 1 × 10^–6^ M.

The emission spectrum of **3a**, in CH_2_Cl_2_, at 298 K is independent of the emitter concentration, in the range (1 × 10^–3^)–(1 × 10^–6^) M, and superimposable with that observed in PMMA_5%_ (Figure 7a). The lifetime increases from 0.8 to 11.9 μs, and the quantum yields from 0.05 to 0.60 as the emitter concentration decreases. This is indicative of self-quenching induced by ground-state aggregation.^31^ Although excimer emission is not observed in the 700 nm region,^32^ the rate of emission decay (*k*_obs_ = 1/τ) fits well to the modified Stern–Volmer expression shown in eq 1, where *k*_q_ is the rate constant for the excimer formation, [Pt] is the emitter concentration, and *k*_0_ (=1/τ_0_) is the rate of excited-state decay at infinite dilution. A plot of *k*_obs_*versus* [Pt] (Figure S15) provides values for the self-quenching rate constant *k*_q_ and the intrinsic lifetime τ_0_ of 1.2 × 10^9^ M^–1^ s^–1^ and 11.5 μs, respectively.^33^ At 77 K, the excimer life rises. As a consequence, at this temperature, the emission spectra of solutions, more concentrated than 1 × 10^–6^ M, clearly show the excimer broadband at about 700 nm, which increases its intensity as the emitter concentration also increases (Figure 7b). An analogous behavior was observed for the phenylacetylide derivative **4a**, which displays *k*_q_ and τ_0_ values of 0.9 × 10^9^ M^–1^ s^–1^ and 10.6 μs, respectively (see Figure S16). These values compare well with those reported for other emissive platinum complexes.^31,33a,34^
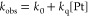
1

The solvent has a dramatic influence on the emission. For example, 2-MeTHF prevents self-quenching of **4a**, as shown in Figure S101, although changes in the relative intensity of the structured band peaks are observed as a consequence of variations in the emitter concentration. In addition, a mitigation of the quantum yield in PMMA_5%_ from 0.22 to a constant value of about 0.06 also occurs. The effect can be assigned to the different solvation abilities of the solvents and to a noticeable coordinating ability of the ether, which protects the unsaturated monomers.

There are significant differences between **4a** and its thermodynamic isomer **4d** in CH_2_Cl_2_. At 298 K, the structured emission of the latter consists of two peaks of similar intensity at 493 and 524 nm and a shoulder at 562 nm. As for **4a**, this shape is independent of the emitter concentration, in the range (1 × 10^–3^)–(1 × 10^–6^ M) (Figures S63–S66) and superimposable with that obtained in PMMA_5%_ (Figures S62). However, in contrast to **4a**, the quantum yield for **4d** is constant in the concentration range and about ten times lower than in PMMA_5%_ (0.61 *versus* 0.07). Although the relative intensity of the peaks of the emission changes at 77 K, an excimer band is not observed. In this case, the significant mitigation of the quantum yields in CH_2_Cl_2_ appears to be due to a notable increase of the nonradiative rate constant in solution, which is an order of magnitude higher than that in PMMA_5%_ (5.1 × 10^4^*versus* 5.2 × 10^5^ s^–1^ (1 × 10^–3^ M)).

**Figure 8:**
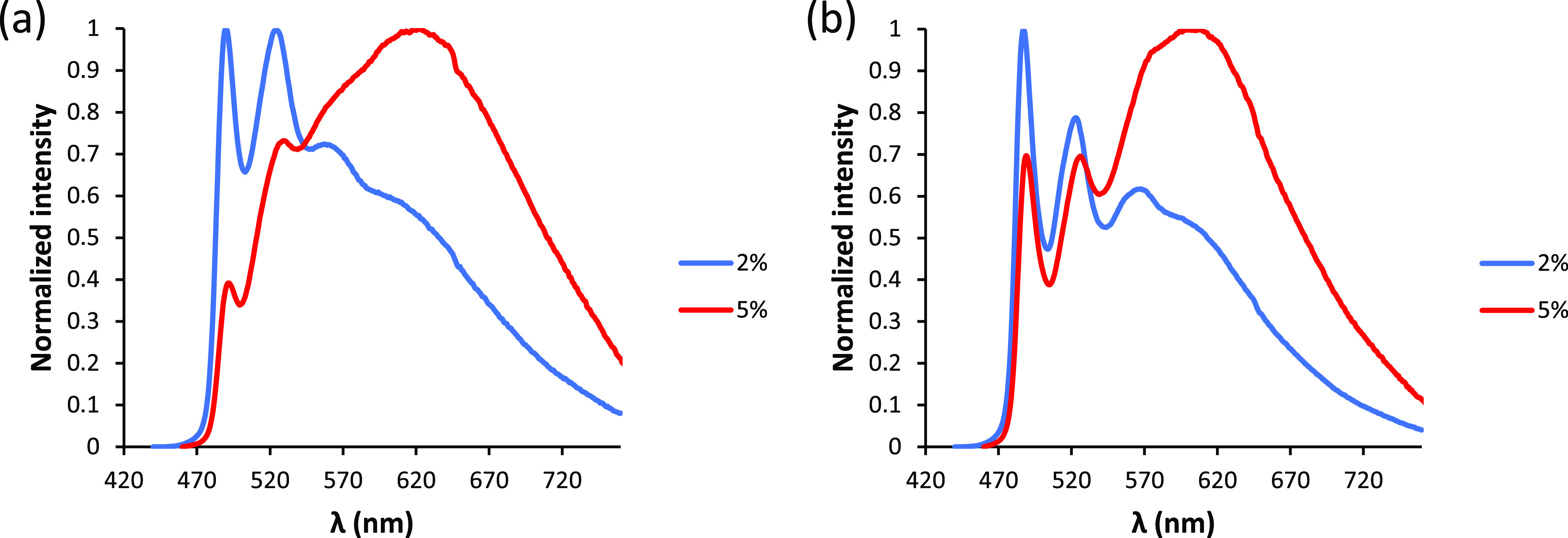
(a) Emission spectra of **4c** in PMMA_2%_ and PMMA_5%_. (b) Emission spectra of **5c** in PMMA_2%_ and PMMA_5%_.

Acetylido derivatives with a pyridine substituent **4c** and **5c** undergo self-quenching in the solid state (Figure 8).^35^ Thus, the emission spectra in PMMA show an excimer broadband centered around 640 nm, in addition to the structured pattern of two peaks and a shoulder in the 490–570 nm region, which is characteristic of this class of complexes. As expected, the excimer emission significantly rises its intensity as the emitter concentration increases, being the most intense band at 5 wt %. The increase of the intensity of this band is accompanied by a decrease of the quantum yield of the emission, which diminishes from 0.26 to 0.15 for **4c** and from 0.50 to 0.35 for **5c** when the emitter concentration in the film increases from 2 to 5 wt %.

**Figure 9:**
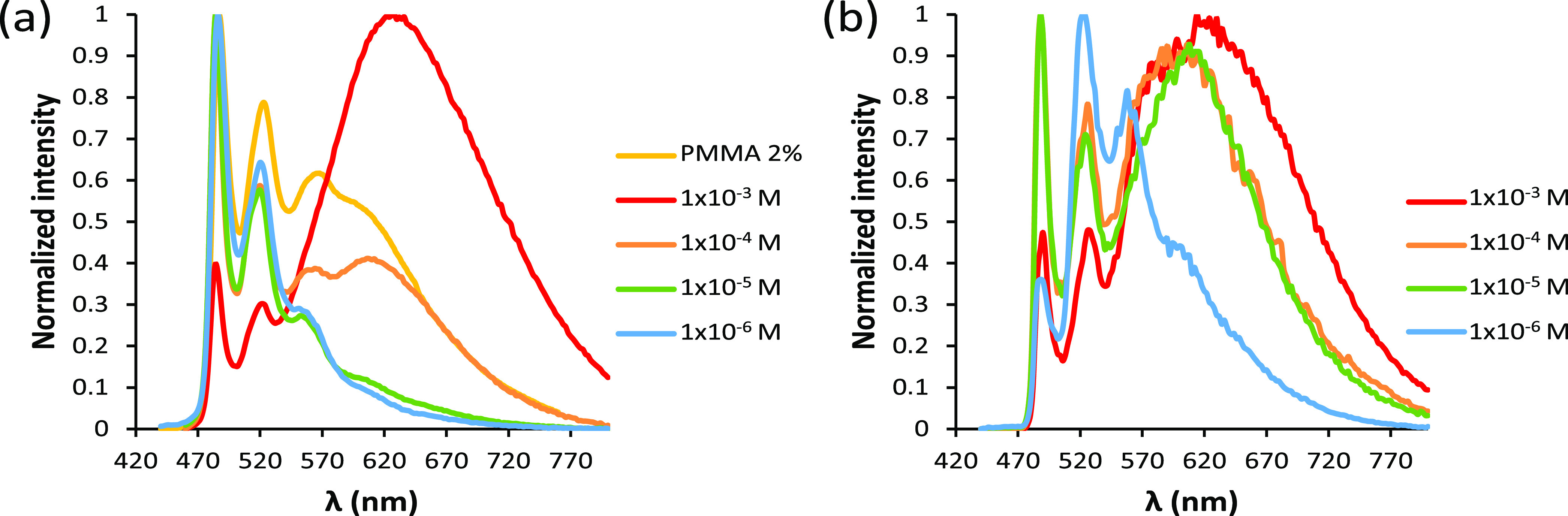
(a) Emission spectra of **5c** at 298 K in PMMA_2%_ and in dichloromethane at concentrations between 1 × 10^–3^ and 1 × 10^–6^ M. (b) Emission spectra of **5c** at 77 K in dichloromethane at concentrations between 1 × 10^–3^ and 1 × 10^–6^ M.

The behavior of emitter **5c** was also studied in solution, as a function of its concentration, in the range (1 × 10^–3^)–(1 × 10^–6^) M, in CH_2_Cl_2_, at 298 and 77 K. Consistently with the behavior in the PMMA film, **5c** undergoes self-quenching in the solvent at both temperatures (Figure 9). As the emitter concentration rises, the intensity of the excimer broadband around 640 nm increases at the expense of peaks between 480 and 560 nm of the structured emission. At 298 K, the values obtained for the self-quenching rate constant *k*_q_ and the intrinsic lifetime τ_0_ are 1.9 × 10^9^ M^–1^ s^–1^ and 7.8 μs, respectively.

## Conclusions

We describe an efficient methodology to prepare a new class of pincer complexes, with structures [5.5]- and [6.5]-Pt{κ^3^-*N*,*C*,*N*′-[L]}X (X = Cl, RC≡C), built on 6-phenyl purines. The nucleobase skeleton provides an *N*,*C*-cyclometalated fragment, which was additionally functionalized with an amine, imine, or pyridine arm to afford the *N*,*C*,*N*′*-*pincer structure. The reactions were totally regioselective. Although the formation of [5.5]-bicycle compounds, bearing the purine bonded by *N*1, is kinetically favored for the amine arm, the [6.5]-bicycle isomers resulting from the *N*7 coordination of the purine are more stable in all of the cases. The combination of purine nucleoside pincer ligands with ethynylpurine nucleosides has furthermore allowed the preparation of novel heteroleptic bis-nucleoside compounds, which may be viewed as organometallic models of Pt-induced interstrand cross-link. The pincer compounds Pt{κ^3^-*N*,*C*,*N*′-[L]}X (X = Cl, RC≡C) [Pt(N∧C∧N′)L] are square-planar complexes having the monodentate X ligand trans to the cyclometalated phenyl ring. In the solid state, noncovalent interactions between adjacent molecules have been observed by XRD. The complexes bearing an amine or pyridine arm are phosphorescent green emitters upon photoexcitation, displaying high quantum yields in PMMA and dichloromethane at low concentrations. At high concentrations, they undergo self-quenching favored by molecular aggregation of monomers through aromatic π–π interactions that are reinforced by weak platinum–platinum interactions.

## Experimental Section

### General Methods

Unless stated otherwise, all of the reactions were carried out under an Ar atmosphere using anhydrous solvents. The reaction work-ups were performed in air. Commercially available reagents were used as received without further purification. 6-Chloro-9-ethylpurine,^36^ 3-(2-pyridynyl)phenylboronic acid pinacol ester,^37^ 6-ethynyl-9-ethylpurine,^38^ and PtCl_2_(DMSO)_2_^39^ were prepared according to reported protocols. ^1^H and ^13^C{^1^H} NMR spectra were recorded at ambient temperature in CDCl_3_ or CD_2_Cl_2_ on Bruker 500 or 300 MHz spectrometers. Chemical shifts are expressed in ppm and are referenced to residual solvent peaks. Through the experimental part, in the NMR spectra, the numbering of the purine ring system has been used to denote the positions *C*2 (*H*2) and *C*8 (*H*8) of the nucleobase. Fourier transform infrared (FT-IR) spectra (attenuated total reflection (ATR)) were recorded with solid or films (by slow evaporating CHCl_3_ solutions of the compounds) on a Bruker Alpha spectrometer. Electrospray ionization-high-resolution mass spectrometry (ESI-HRMS) was performed on an Agilent 6500 accurate mass spectrometer with a Q-TOF analyzer. UV–visible spectra were registered on an Evolution 600 spectrophotometer. Steady-state photoluminescence spectra were recorded with either a Jobin-Yvon Horiba Fluorolog FL-3-11 Tau 3 spectrometer (PMMA films) or with a PicoQuant FluoTime 300 spectrometer (CH_2_Cl_2_ and 2-MeTHF solutions). Lifetime measurements were performed at the maximum emission wavelength of the complexes either on a Jobin-Yvon Horiba Fluorolog FL-3-11 Tau 3 spectrometer (PMMA films) or a PicoQuant FluoTime 300 spectrometer (CH_2_Cl_2_ and 2-MeTHF solutions). Data were fitted to either monoexponential or biexponential functions. Quantum yields were measured using the Hamamatsu Absolute PL Quantum Yield Measurement System C11347-11.

### Computational Details

All calculations were performed at the DFT level using the B3LYP functional as implemented in Gaussian09^40^ supplemented with the Grimme’s dispersion correction D3^41^ and the def2-SVP basis set.^42^ All minima were verified to have no negative frequencies. The geometries were fully optimized in vacuo and in the appropriate solvent using the continuum SMD model.^43^

### X-ray Diffraction Analyses

Crystals of **3a·**0.5(CHCl_3_), **3b**, **3c·**0.5(CH_2_Cl_2_), **3d**, **4b**, **4d**, and **5a** were analyzed by X-ray diffraction. A selection of crystal, measurement, and refinement data is given in Tables S1 and S2. Diffraction data were collected on an Oxford Diffraction Xcalibur Onyx Nova single-crystal diffractometer with Cu Kα radiation. Empirical absorption corrections were applied using the SCALE3 ABSPACK algorithm as implemented in CrysAlisPro RED.^44^ The structures were solved with SIR-97.^45^ Isotropic and full matrix anisotropic least-squares refinements were carried out using SHELXL.^46^ All non-H atoms were refined anisotropically. H atoms were set in calculated positions and were refined riding on their parent atoms. The CH_2_ and CH_3_ groups of the fragment CH_2_N(CH_3_)_2_ of **5a** were disordered over two positions with a 54:46 occupancy ratio, requiring restraints on its thermal parameters. The structure of **4d** was refined as a 2-component inversion twin. The high-resolution reflections 8̅ 3 4, 8̅ 2 3, and 5 21 2 were left out from the refinement of **3b** since their intensities were likely affected by some unresolved twinning, resulting in high *S* values. The low-resolution reflections 3̅ 3̅ 2 and 1̅ 7̅ 13 were left out from the refinement of **3d** since their intensities were likely affected by the beamstop, resulting in high *S* values. The WINGX program system^47^ was used throughout the structure determinations. The molecular plots were made with MERCURY.^48^

#### Synthesis of **1**

3-Formylphenylboronic acid (1.69 g, 8.7 mmol), Pd(PPh_3_)_4_ (388 mg, 0.34 mmol), and K_2_CO_3_ (1.20 g, 8.7 mmol) were added to a solution of 6-chloro-9-ethylpurine (1.22 g, 6.7 mmol) in 60 mL of toluene/ethanol (9:1). The mixture was refluxed under argon for 24 h. The solvent was removed under reduced pressure, and the crude residue was purified by flash SiO_2_ chromatography (hexane/ethyl acetate, 1:1 to ethyl acetate) to yield **1** (white solid) (1.61 g, 95%). ^1^H NMR (300 MHz, CDCl_3_), δ (ppm): 10.2 (s, 1H, CHO), 9.33 (t, 1H, CH_arom_), 9.12 (dt, 1H, CH_arom_), 9.06 (s, 1H, CH2), 8.19 (s, 1H, CH8), 8.07 (dt, 1H, CH_arom_), 7.74 (t, *J* = 7.5 Hz, 1H, CH_arom_), 4.41 (q, 2H, *J* = 6.7 Hz, CH_2_), 1.62 (t, 3H, *J* = 6.7 Hz, CH_3_). ^13^C NMR (75 MHz, CDCl_3_), δ (ppm): 192.4 (CHO), 153.2 (C_quaternary_), 152.5 (C2), 149.2 (C_quaternary_), 144.4 (C8), 137.0 (C_quaternary_), 135.7 (C_arom_), 132.4 (C_arom_), 131.5 (C_quaternary_), 130.7 (C_arom_), 129.6 (C_arom_), 39.2 (CH_2_), 15.6 (CH_3_). IR (cm^–1^): ν 3104, 2919, 1711, 1576. ESI-HRMS *m*/*z*: calcd for C_14_H_13_N_4_O [M + H]^+^ 253.1083; found 253.1082.

#### Synthesis of **2a**

HNMe_2_·HCl (679 mg, 8.32 mmol), Ti(O*^i^*Pr)_4_ (2.4 mL, 7.93 mmol), and Et_3_N (2.5 mL) were added to a solution of **1** (1.00 g, 3.96 mmol) in 100 mL of ethanol, and the mixture was stirred at room temperature overnight. Then, NaBH_3_CN (374 mg, 5.94 mmol) was added, and the solution was stirred at room temperature for 24 h. The reaction mixture was quenched with water and extracted with DCM. The organic layers were dried over Na_2_SO_4_, and the solvent was evaporated under reduced pressure to yield **2a** (colorless oil) (1.09 g, 98%). ^1^H NMR (300 MHz, CDCl_3_), δ (ppm): 8.98 (s, 1H, CH2), 8.65–8.61 (m, 2H, CH_arom_), 8.10 (s, 1H, CH8), 7.52–7.44 (m, 2H, CH_arom_), 4.32 (q, *J* = 7.3 Hz, 2H, CH_2_), 3.54 (s, 2H, NCH_2_), 2.25 (s, 6H, N(CH_3_)_2_), 1.53 (t, *J* = 7.3 Hz, 3H, CH_3_). ^13^C NMR (75 MHz, CDCl_3_), δ (ppm): 154.8 (C_quaternary_), 152.3 (C_quaternary_), 152.3 (C2), 143.9 (C8), 139.2 (C_quaternary_), 135.8 (C_quaternary_), 131.8 (C_arom_), 131.2 (C_quaternary_), 130.4 (C_arom_), 128.9 (C_arom_), 128.7 (C_quaternary_), 64.3 (NCH_2_), 45.3 (N(CH_3_)_2_), 39.0 (CH_2_), 15.6 (CH_3_). IR (cm^–1^): 2923, 2852, 1572, 1498. ESI-HRMS *m*/*z*: calcd for C_16_H_20_N_5_ [M + H]^+^ 282.1713, found 282.1718.

#### Synthesis of **2b**

*p*-Anisidine (244 mg, 1.98 mmol) and MgSO_4_ (10% w/w) were added to a solution of **1** (500 mg, 1.98 mmol) in 10 mL of DCM. The mixture was stirred at room temperature for 24 h. Then, MgSO_4_ was filtered off, and the solvent was removed under reduced pressure to yield **2b** (white solid) (687.5 mg, 97%, EtOH). ^1^H NMR (300 MHz, CDCl_3_), δ (ppm): 9.19 (t, *J* = 1.7 Hz, 1H, CH_arom_), 9.06 (s, 1H, CH2), 8.93 (dt, *J* = 7.8, 1.5 Hz, 1H, CH_arom_), 8.66 (s, 1H, CH=N), 8.19 (m, 1H, CH_arom_), 8.17 (s, 1H, CH8), 7.68 (t, *J* = 7.8 Hz, 1H, CH_arom_), 7.29 (d, *J* = 8.9 Hz, 2H, CH_arom_), 6.95 (d, *J* = 8.9 Hz, 2H, CH_arom_), 4.40 (q, *J* = 7.3, 2H, CH_2_), 3.85 (s, 3H, CH_3_), 1.62 (t, *J* = 7.3, 3H, CH_3_). ^13^C NMR (75 MHz, CDCl_3_), δ (ppm): 158.5 (C_quaternary_), 158.2 (CH=N), 154.2 (C_quaternary_), 152.6 (C_quaternary_), 152.5 (C2), 145.0 (C_quaternary_), 144.2 (C8), 137.2 (C_quaternary_), 136.5 (C_quaternary_), 132.5 (C_arom_), 131.5 (C_quaternary_), 130.9 (C_arom_), 130.0 (C_arom_), 129.3 (C_arom_), 122.5 (C_arom_), 114.5 (C_arom_), 55.6 (OCH_3_), 39.2 (CH_2_), 15.6 (CH_3_). IR (film) ν (cm^–1^): 2923, 1618, 1572, 1498, 1235. ESI-HRMS *m*/*z*: calcd for C_21_H_20_N_5_O1 [M + H]^+^, 358.1662; found 358.1660.

#### Synthesis of **2c**

To a solution of 6-chloro-9-ethylpurine (174 mg, 0.95 mmol) in 15 mL of a mixture of toluene and ethanol (9:1) were added 3-(2-pyridynyl)phenylboronic acid pinacol ester (348 mg, 1.23 mmol), Pd(PPh_3_)_4_ (79 mg, 0.07 mmol), and K_2_CO_3_ (246 mg, 1.8 mmol). The mixture was refluxed under argon for 24 h. The solvent was removed under reduced pressure, and the residue was purified by flash SiO_2_ chromatography (hexane/ethyl acetate, 1:1 to ethyl acetate) to yield **2c** (white solid) (155 mg, 54%). ^1^H NMR (300 MHz, CDCl_3_), δ (ppm): 9.39 (br s, 1H, CH_arom_), 9.04 (s, 1H, CH2), 8.89 (d, *J* = 6.8 Hz, 1H, CH_arom_), 8.74 (d, *J* = 4.4 Hz, 1H, CH_arom_), 8.23 (d, *J* = 6.6 Hz, 1H, CH_arom_), 8.16 (s, 1H, CH8), 7.92 (d, *J* = 8.93 Hz, 1H, CH_arom_), 7.80 (td, *J* = 7.7 and 1.7 Hz, 1H, CH_arom_), 7.68 (t, *J* = 7.8 Hz, 1H, CH_arom_), 7.29–7.25 (m, 1H, CH_arom_), 4.37 (q, *J* = 7.3 Hz, 2H, CH_2_), 1.59 (t, *J* = 7.3 Hz, 3H, CH_3_). ^13^C NMR (75 MHz, CDCl_3_), δ (ppm): 157.0 (C_quaternary_), 154.4 (C_quaternary_), 152.5 (C_quaternary_), 152.3 (C2), 149.5 (C_arom_), 144.0 (C8), 139.5 (C), 137.1 (C_arom_), 136.3 (C_quaternary_), 132.1 (C_quaternary_), 130.6 (CH_arom_), 129.5 (CH_arom_), 129.3 (CH_arom_), 128.2 (CH_arom_), 122.4 (CH_arom_), 121.0 (CH_arom_), 39.0 (CH_2_), 15.4 (CH_3_). IR (film) ν (cm^–1^): 2982, 1565, 1315. ESI-HRMS *m*/*z:* calcd for C_18_H_16_N_5_, [M + H]^+^, 302.1400; found 302.1400.

#### Synthesis of **3a**

Compound **2a** (213 mg, 0.756 mmol) was added to a suspension of K_2_PtCl_4_ (314 mg, 0.756 mmol) in 15 mL of AcOH. The mixture was refluxed under argon for 3 days. The solvent was removed under reduced pressure, and the residue was purified by flash SiO_2_ chromatography (DCM/ethyl acetate 1:1 to 1:9) to yield **3a** (bright yellow solid) (215 mg, 56%). ^1^H NMR (500 MHz, CDCl_3_), δ (ppm): 9.40 (s, *J*(^1^H–^195^Pt) = 11.5 Hz, 1H, CH2), 8.16 (m, 1H, CH_arom_), 8.14 (s, 1H, CH8), 7.12 (t, *J* = 7.6 Hz, 1H, CH_arom_), 6.99 (d, *J* = 7.0 Hz, 1H, CH_arom_), 4.37 (q, *J* = 7.3 Hz, 2H, CH_2_), 4.25 (s, *J*(^1^H–^195^Pt) = 17.0 Hz, 2H, NCH_2_), 3.25 (s, *J*(^1^H–^195^Pt) = 15.8 Hz, 6H, N(CH_3_)), 1.60 (t, *J* = 7.4 Hz, 3H, CH_3_). ^13^C NMR (126 MHz, CDCl_3_), δ (ppm): 165.7 (C–Pt), 157.0 (C_quaternary_), 154.3 (C2), 152.5 (C_quaternary_), 145.9 (C8), 143.4 (C_quaternary_), 138.4 (C_quaternary_), 129.2 (C_quaternary_), 127.7 (C_arom_), 125.1 (C_arom_), 123.2 (C_arom_), 77.9 (NCH_2_), 54.2 (N(CH_3_)_2_), 39.2 (CH_2_), 15.2 (CH_3_). IR (film) ν (cm^–1^): 2928, 1747, 1577, 1499, 1457. ESI**-**HRMS *m*/*z:* calcd for C_16_H_18_N_5_Pt, [M–Cl]^+^, 475.1206; found 475.1209.

#### Synthesis of **3b**

As for **3a**, from K_2_PtCl_4_ (232 mg, 0.56 mmol) in 15 mL of AcOH and **2b** (200 mg, 0.56 mmol). The solvent was removed under reduced pressure, and the residue was purified by flash SiO_2_ chromatography (DCM/ethyl acetate, 2:8) to yield **3b** (bright yellow solid) (151 mg, 46%). ^1^H NMR (300 MHz, CD_2_Cl_2_), δ (ppm): 9.29 (s, *J*(^1^H–^195^Pt) = 11.2 Hz, 1H, CH8), 8.99 (s, 1H, CH2), 8.54 (dd, *J* = 7.9, 1.2 Hz, 1H, CH_arom_), 8.33 (s, *J*(^1^H–^195^Pt) = 58.6 Hz, 1H, CH=N), 7.65 (dd, *J* = 6.7, 1.3 Hz, 1H, CH_arom_), 7.45 (d, *J* = 8.8 Hz, 2H, CH_arom_), 7.37 (t, *J* = 8.2 Hz, 1H, CH_arom_), 6.96 (d, *J* = 8.8 Hz, 2H, CH_arom_), 4.39 (q, *J* = 7.4 Hz, 2H, CH_2_), 3.87 (s, 3H, OCH_3_), 1.58 (t, *J* = 7.5 Hz, 3H, CH_3_). ^13^C NMR (75 MHz, CD_2_Cl_2_), δ (ppm): 178.1 (C=N), 159.8 (C–Pt), 155.3 (C_quaternary_), 154.0 (C2), 150.7 (C_quaternary_), 150.0 (C_quaternary_), 147.9 (C_quaternary_), 145.8 (C8), 142.9 (C_quaternary_), 132.5 (C_arom_), 130.1 (C_arom_), 128.7 (C_quaternary_), 126.0 (C_arom_), 124.3 (C_arom_), 121.7 (C_quaternary_), 113.6 (C_arom_), 56.1 (OCH_3_), 41.2 (CH_2_), 15.3 (CH_3_). IR (film) ν (cm^–1^): 2965, 1607, 1507. ESI-HRMS *m*/*z:* calcd for C_21_H_18_N_5_OPt, [M–Cl]^+^, 551.1155; found 551.1158.

#### Synthesis of **3c**

As for **3a**, from K_2_PtCl_4_ (276 mg, 0.66 mmol) in 15 mL of AcOH and **2c** (200 mg, 0.66 mmol). The mixture was refluxed under argon for 4 days. The product precipitated after cooling. The solid was filtered and washed with cold MeOH and diethyl ether to yield **3c** (bright yellow solid) (183 mg, 52%). ^1^H NMR (500 MHz, CD_2_Cl_2_), δ (ppm): 9.62 (d, *J* = 5.9 Hz, *J*(^1^H–^195^Pt) = 15.9 Hz, 1H, CH_arom_), 9.37 (s, *J*(^1^H–^195^Pt) = 14.2 Hz, 1H, CH8), 8.98 (s, 1H, CH2), 8.61 (d, *J* = 10.0 Hz, 1H, CH_arom_), 7.92 (t, *J* = 10.0 Hz, 1H, CH_arom_), 7.77 (d, *J* = 8.3 Hz, 1H, CH_arom_), 7.71 (d, *J* = 7.7 Hz, 1H, CH_arom_), 7.36 (t, *J* = 6.2 Hz, 1H, CH_arom_), 7.25 (t, *J* = 6.6 Hz, 1H, CH_arom_), 4.45 (q, *J* = 7.4 Hz, 2H, CH_2_), 1.64 (t, *J* = 7.4 Hz, 3H, CH_3_). ^13^C NMR (126 MHz, CD_2_Cl_2_), δ (ppm): 166.6 (C_quaternary_), 156.1 (C–Pt), 154.2 (C2), 150.9 (C_arom_), 150.7 (C_quaternary_), 146.9 (C_quaternary_), 146.4 (C_quaternary_), 146.2 (C8), 139.4 (C_arom_), 129.3 (C_quaternary_), 128.5 (C_arom_), 127.4 (C_arom_), 124.6 (C_arom_), 123.2 (C_arom_), 121.9 (C_quaternary_), 119.4 (C_quaternary_), 41.1 (CH_2_), 15.5 (CH_3_). IR (film) ν (cm^–1^): 2963, 1607, 1581. ESI-HRMS *m*/*z:* calcd for C_18_H_14_N_5_Pt, [M – Cl]^+^, 495.0893; found, 495.0883.

#### Synthesis of **3d**

A solution of **3a** (200 mg, 0.39 mmol) in 5 mL of dry toluene was heated in a sealed tube at 200 °C for 5 days. After the removal of the solvent under vacuum, the crude product was purified by flash SiO_2_ chromatography (DCM/ethyl acetate, 2:8) to yield **3d** (yellow solid, 197 mg, 99%). ^1^H NMR (500 MHz, CD_2_Cl_2_), δ (ppm): 9.14 (s, *J*(^1^H–^195^Pt) = 10.5 Hz, 1H, CH8), 8.99 (s, 1H, CH2), 8.42–8.39 (m, 1H, CH_arom_), 7.25–7.21 (m, 2H, CH_arom_), 4.37 (q, *J* = 7.3 Hz, 2H, CH_2_), 4.05 (s, *J*(^1^H–^195^Pt) = 19.7 Hz, 2H, CH_2_N), 3.10 (s, *J*(^1^H–^195^Pt) = 16.2 Hz, 6H, N(CH_3_)_2_), 1.57 (t, *J* = 7.3 Hz, 3H, CH_3_). ^13^C NMR (126 MHz, CD_2_Cl_2_), δ (ppm): 156.6 (C–Pt), 154.3 (C2), 150.6 (C_quaternary_), 150.3 (C_quaternary_), 145.6 (C8), 141.5 (C_quaternary_), 129.2 (C_quaternary_), 124.9 (C_arom_), 124.3 (C_arom_), 124.0 (C_arom_), 121.7 (C_quaternary_), 76.1 (CH_2_N), 54.2 (N(CH_3_)_2_), 40.9 (CH_2_), 15.5 (CH_3_). IR (film) ν (cm^–1^): 2899, 1605, 1411, 1202. ESI-HRMS *m*/*z:* calcd for C_16_H_18_N_5_Pt, [M – Cl]^+^, 475.1206; found 475.1198.

#### Synthesis **4a**

A mixture of phenylacetylene (15 mg, 0.15 mmol) and NaOH (6.0 mg, 0.15 mmol) in 5 mL of MeOH was stirred for 30 min at rt. Then, **3a** (50 mg, 0.1 mmol) was added to the reaction mixture and further stirred for 24 h. After filtration, the resulting solid was washed with cold methanol and diethyl ether to yield **4a** (bright yellow solid) (43 mg, 76%). ^1^H NMR (500 MHz, CD_2_Cl_2_), δ (ppm): 9.54 (s, *J*(^1^H–^195^Pt) = 13.9 Hz, 1H, CH2), 8.25 (dd, *J* = 6.1, 2.4 Hz, 1H, CH_arom_), 8.16, (s, 1H, CH8), 7.43–7.40 (m, 2H, CH_arom_), 7.27–7.22 (m, 2H, CH_arom_), 7.16–7.10 (m, 3H, CH_arom_), 4.41–4.27 (m, 4H, CH_2_ and CH_2_N), 3.36 (s, *J*(^1^H–^195^Pt) = 21.4 Hz, 6H, N(CH_3_)_2_), 1.57 (t, *J* = 7.3 Hz, 3H, CH_3_). ^13^C NMR (126 MHz, CD_2_Cl_2_), δ (ppm): 177.2 (C–Pt), 168.5 (C–Pt), 157.7 (C2), 152.9 (C_quaternary_), 147.0 (C_quaternary_), 146.7 (C8), 141.4 (C_quaternary_), 136.5 (C_quaternary_), 131.9 (C_arom_), 129.5 (C_quaternary_), 128.5 (C_arom_), 127.9 (C_arom_), 125.4 (C_arom_), 125.2 (C_arom_), 123.9 (C_arom_), 123.7 (C_quaternary_), 110.5 (C_quaternary_), 80.8 (CH_2_N), 56.3 (N(CH_3_)_2_), 39.8 (CH_2_), 15.6 (CH_3_). IR (film) ν (cm^–1^): 2926, 2355, 2080, 1602, 1575. ESI-HRMS *m*/*z:* calcd for C_24_H_24_N_5_Pt, [M + H]^+^, 577.1676; found 577.1681.

#### Synthesis of **4b**

As for **4a**, from phenylacetylene (13 mg, 0.13 mmol), NaOH (5.1 mg, 0.13 mmol), and **3b** (50 mg, 0.09 mmol). Complex **4b** was obtained as a bright yellow solid (40 mg, 72%). ^1^H NMR (500 MHz, CD_2_Cl_2_), δ (ppm): 9.32 (s, *J*(^1^H–^195^Pt) = 13.4 Hz, 1H, CH8), 8.96 (s, 1H, CH2), 8.60 (dd, *J* = 7.9, 13 Hz, 1H, CH_arom_), 8.48 (s, *J*(^1^H–^195^Pt) = 53.0 Hz, 1H, CH=N), 7.72 (dd, *J* = 7.3, 1.0 Hz, 1H, CH_arom_), 7.68 (d, *J* = 8.8 Hz, 2H, CH_arom_), 7.38 (t, *J* = 7.5 Hz, 1H, CH_arom_), 7.22–7.19 (m, 4H, CH_arom_), 7.16–7.12 (m, 1H, CH_arom_), 6.95 (d, *J* = 8.8 Hz, 2H, CH_arom_), 4.35 (q, *J* = 7.4 Hz, 2H, CH_2_), 3.85 (s, 3H, OCH_3_), 1.55 (t, *J* = 7.3 Hz, 3H, CH_3_). ^13^C NMR (126 MHz, CD_2_Cl_2_), δ (ppm): 179.4 (C=N), 171.2 (C–Pt), 159.8 (C–Pt), 157.2 (C_quaternary_), 153.9 (C2), 150.6 (C_quaternary_), 149.9 (C_quaternary_), 148.1 (C8), 144.8 (C_quaternary_), 131.8 (C_arom_), 131.7 (C_arom_), 130.7 (C_quaternary_), 129.4 (C_arom_), 129.0 (C_quaternary_), 128.5 (C_arom_), 126.1 (C_arom_), 125.9 (C_quaternary_), 125.7 (C_arom_), 124.5 (C_arom_), 122.0 (C_quaternary_), 113.5 (C_arom_), 108.5 (C_quaternary_), 56.1 (OCH_3_), 41.0 (CH_2_), 15.1 (CH_3_). IR (film) ν (cm^–1^): 2922, 2095, 1603, 1503, 1247. ESI-HRMS *m*/*z*: calcd for C_29_H_24_N_5_OPt, [M + H]^+^, 653.1625; found, 653.1621.

#### Synthesis of **4c**

As for **4b**, from phenylacetylene (12 mg, 0.13 mmol), NaOH (5.1 mg, 0.13 mmol), and **3c** (40 mg, 0.08 mmol). Complex **4c** was obtained as a bright orange solid (35 mg, 78%). ^1^H NMR (500 MHz, CD_2_Cl_2_), δ (ppm): 9.93 (dd, *J* = 6.2 Hz, *J*(^1^H–^195^Pt) = 23.5 Hz, 1H, CH_arom_), 9.36 (s, *J*(^1^H–^195^Pt) = 13.5 Hz, 1H, CH8), 9.04 (s, 1H, CH2), 8.78 (d, *J* = 7.8, 1H, CH_arom_), 7.95–7.91 (m, 1H, CH_arom_), 7.83 (d, *J* = 7.2 Hz, 2H, CH_arom_), 7.55 (dd, *J* = 8.1, 1.4 Hz, 2H, CH_arom_), 7.42 (t, *J* = 7.7 Hz, 1H, CH_arom_), 7.32 (t, *J* = 6.2 Hz, 1H, CH_arom_), 7.24–7.20 (m, 2H, CH_arom_), 4.45 (q, *J* = 7.4 Hz, 2H, CH_2_), 1.63 (t, *J* = 7.3 Hz, 3H, CH_3_). ^13^C NMR (126 MHz, CD_2_Cl_2_), δ (ppm): 168.7 (C–Pt), 167.0 (C–Pt), 158.2 (C_quaternary_), 154.6 (C_arom_), 154.2 (C2), 150.6 (C_quaternary_), 148.7 (C_quaternary_), 148.5 (C8), 139.9 (C_arom_), 131.9 (C_arom_), 131.4 (C_quaternary_), 129.0 (C_arom_), 128.7 (C_arom_), 128.0 (C_arom_), 127.1 (C_quaternary_), 126.4 (C_quaternary_), 126.0 (C_arom_), 124.9 (C_arom_), 123.7 (C_arom_), 122.0 (C_quaternary_), 119.7 (C_arom_), 106.6 (C_quaternary_), 40.9 (CH_2_), 15.4 (CH_3_). IR (film) ν (cm^–1^): 2086, 1605, 1480. ESI-HRMS *m*/*z*: calcd for C_26_H_20_N_5_Pt, [M + H]^+^, 597.1363; found, 597.1345.

#### Synthesis of **4d**

As for **4b**, from phenylacetylene (11 mg, 0.10 mmol), NaOH (3 mg, 0.10 mmol), and **3d** (35 mg, 0.08 mmol). Complex **4d** was obtained as a yellow solid, (21 mg, 53%). ^1^H NMR (500 MHz, CD_2_Cl_2_), δ (ppm): 9.16 (s, *J*(^1^H–^195^Pt) = 15.3 Hz, 1H, CH8), 9.03 (s, 1H, CH2), 8.54 (d, *J* = 6.4 Hz, 1H, CH_arom_), 7.45–7.41 (m, 2H, CH_arom_), 7.35–7.24 (m, 4H, CH_arom_), 7.20–7.14 (m, 1H, CH_arom_), 4.40 (q, *J* = 7.3 Hz, 2H, CH_2_), 4.16 (s, *J*(^1^H–^195^Pt) = 19.0 Hz, 2H, NCH_2_), 3.30 (s, *J*(^1^H–^195^Pt) = 20.8 Hz, 6H, N(CH_3_)_2_), 1.59 (t, *J* = 7.3 Hz, 3H, CH_3_). ^13^C NMR (126 MHz, CD_2_Cl_2_), δ (ppm): 164.7 (C–Pt), 158.7 (C–Pt), 154.2 (C2), 152.2 (C_quaternary_), 150.6 (C_quaternary_), 147.9 (C8), 131.8 (C_arom_), 131.4 (C_quaternary_), 129.2 (C_quaternary_), 128.6 (C_arom_), 125.8 (C_quaternary_), 125.7 (C_arom_), 124.4 (C_arom_), 123.4 (C_arom_), 121.9 (C_quaternary_), 105.2 (C_quaternary_), 78.1 (NCH_2_), 56.0 (N(CH_3_)_2_), 40.7 (CH_2_), 15.5 (CH_3_). IR (film) ν (cm^–1^): 2915, 2088, 1600, 1440, 1200. ESI-HRMS *m*/*z*: calcd for C_24_H_24_N_5_Pt, [M + H]^+^, 577.1676; found, 577.1679.

#### Synthesis of **5a**

As for **4a**, from 9-ethyl-6-ethynylpurine (25 mg, 0.15 mmol), NaOH (5.9 mg, 0.15 mmol), and **3a** (50 mg, 0.1 mmol). Complex **5a** was obtained as a bright yellow solid (41 mg, 65%). ^1^H NMR (500 MHz, CDCl_3_), δ (ppm): 9.74 (s, *J*(^1^H–^195^Pt) = 12.7 Hz, 1H, CH2), 8.84 (s, 1H, CH2′), 8.28 (dd, *J* = 7.7, 1.5 Hz, 1H, CH_arom_), 8.14 (s, 1H, CH8), 8.04 (s, 1H, CH8′), 7.18–7.10 (m, 2H, CH_arom_), 4.43–4.28 (m, 6H, 2CH_2_ and NCH_2_), 3.46 (s, *J*(^1^H–^195^Pt) = 20.4 Hz, 6H, N(CH_3_)_2_), 1.60–1.54 (m, 6H, 2CH_3_). ^13^C NMR (126 MHz, CDCl_3_), δ (ppm): 176.0 (C–Pt), 168.1 (C–Pt), 157.9 (C2), 155.6 (C_quaternary_), 152.7 (C2′), 152.4 (C_quaternary_), 150.7 (C_quaternary_), 146.4 (C_quaternary_), 145.9 (C8), 145.4 (C_quaternary_), 142.9 (C8′), 141.4 (C_quaternary_), 135.0 (C_quaternary_), 129.8 (C_quaternary_), 127.8 (C_arom_), 124.9 (C_arom_), 124.1 (C_arom_), 106.4 (C_quaternary_), 80.4 (NCH_2_), 56.3 (N(CH_3_)_2_), 39.4 (CH_2_), 38.9 (CH_2_), 15.6 (CH_3_), 15.4 (CH_3_). IR (film) ν (cm^–1^): 2936, 2078, 1603, 1560, 1208. ESI-HRMS *m*/*z*: calcd for C_25_H_26_N_9_Pt, [M + H]^+^, 647.1955; found, 647.1951.

#### Synthesis of **5b**

As for **4a**, from 9-ethyl-6-ethynylpurine (22 mg, 0.13 mmol), NaOH (5.1 mg, 0.13 mmol), and **3b** (50 mg, 0.09 mmol). Complex **5b** was obtained as a bright orange solid (49 mg, 79%). ^1^H NMR (500 MHz, CD_2_Cl_2_), δ (ppm): 10.5 (s, 1H, *J*(^1^H–^195^Pt) = 12.7 Hz, C8), 8.83 (s, 1H, C2), 8.66 (s, 1H, C2′), 8.48 (dd, 1H, *J* = 7.9, 1.2 Hz, CH_arom_), 8.42 (s, 1H, *J*(^1^H–^195^Pt) = 53.4 Hz, CH=N), 7.72 (d, 2H, *J* = 8.7 Hz, CH_arom_), 7.71 (s, 1H, C8′), 7.66 (dd, 1H, *J* = 7.3 and 1.2 Hz, CH_arom_), 7.35 (t, 1H, *J* = 7.6 Hz, CH_arom_), 6.94 (d, 2H, *J* = 8.9 Hz, CH_arom_), 4.45 (q, 2H, *J* = 7.4 Hz, CH_2_), 4.15 (q, 2H, *J* = 7.3 Hz, CH_2_), 3.84 (s, 3H, CH_3_), 1.52 (t, 3H, *J* = 7.4 Hz, CH_3_), 1.49 (t, 3H, *J* = 7.3 Hz, CH_3_). ^13^C NMR (126 MHz, CD_2_Cl_2_), δ (ppm): 179.2 (C=N), 169.6 (C–Pt), 159.8 (C–Pt), 156.4 (C_quaternary_), 153.4 (C2), 152.7 (C2), 150.8, (C8), 150.6 (C_quaternary_), 150.6 (C_quaternary_), 149.8 (C_quaternary_), 145.2 (C_quaternary_), 144.9 (C_quaternary_), 144.6 (C_quaternary_), 142.8 (C8), 135.6 (C_quaternary_), 131.6 (C_arom_), 130.8 (C_quaternary_), 129.3 (C_arom_), 126.1 (C_arom_), 124.8 (C_arom_), 122.0 (C_quaternary_), 113.6 (C_arom_), 104.9 (C_quaternary_), 56.1 (OCH_3_), 40.8 (CH_2_), 39.3 (CH_2_), 15.6 (CH_3_), 15.5 (CH_3_). IR (film) ν (cm^–1^): 3012, 2079, 1602, 1572, 1208. ESI-HRMS *m*/*z*: calcd for C_30_H_26_N_9_OPt, [M + H]^+^, 723.1904; found, 723.1882.

#### Synthesis of **5c**

As for **4a**, from 9-ethyl-6-ethynylpurine (12 mg, 0.13 mmol), NaOH (5.1 mg, 0.13 mmol), and **3c** (40 mg, 0.08 mmol). Complex **5c** was obtained as a bright orange solid (35 mg, 78%). ^1^H NMR (500 MHz, CD_2_Cl_2_), δ (ppm): 10.6 (s, *J*(^1^H–^195^Pt) = 14.8 Hz, 1H, CH8), 9.86 (d, *J* = 6.3 Hz, *J*(^1^H–^195^Pt) = 24.6 Hz, 1H, CH_arom_), 8.97 (s, 1H, CH2), 8.81 (s, 1H, CH2′), 8.73 (d, *J* = 7.4 Hz, 1H, CH_arom_), 8.01 (s, 1H, CH8′), 7.92 (t, *J* = 7.4 Hz, 1H, CH_arom_), 7.80 (d, *J* = 9.8 Hz, 2H, CH_arom_), 7.40 (t, *J* = 7.4, 1H, CH_arom_), 7.24 (t, *J* = 7.4 Hz, 1H, CH_arom_), 4.61 (q, *J* = 7.1 Hz, 2H, CH_2_), 4.32 (q, *J* = 7.6 Hz, 2H, CH_2_), 1.73 (t, *J* = 7.1 Hz, 3H, CH_3_), 1.58 (t, *J* = 7.4 Hz, 3H, CH_3_). ^13^C NMR (126 MHz, CD_2_Cl_2_), δ (ppm): 170.1 (C–Pt), 160.3 (C_arom_), 156.8 (C–Pt), 153.8 (C2), 153.8 (C2), 153.1, (C8), 151.2 (C_quaternary_), 151.0 (C_quaternary_), 150.2 (C_quaternary_), 145.6 (C_quaternary_), 154.3 (C_quaternary_), 145.0 (C_quaternary_), 143.3 (C8), 136.0 (C_quaternary_), 131.2 (C_arom_), 129.7 (C_quaternary_), 126.5 (C_arom_), 125.2 (C_arom_), 122.4 (C_arom_), 105.4 (C_quaternary_), 41.3 (CH_2_), 39.8 (CH_2_), 16.1 (CH_3_), 15.9 (CH_3_). IR (film) ν (cm^–1^): 2964, 2366, 2086, 1563. ESI-HRMS *m*/*z*: calcd for C_27_H_22_N_9_Pt, [M + H]^+^, 667.1642; found, 667.1623.

#### Synthesis of **5d**

As for **4a**, from 9-ethyl-6-ethynylpurine (15.2 mg, 0.09 mmol), NaOH (3.5 mg, 0.09 mmol), and **3d** (30 mg, 0.06 mmol). Complex **5d** was obtained as a yellow solid (25.8 mg, 68%). ^1^H NMR (500 MHz, CD_2_Cl_2_), δ (ppm): 10.4 (s, *J*(^1^H–^195^Pt) = 13.3 Hz, 1H, CH8), 8.99 (s, 1H, CH2), 8.88 (s, 1H, CH_2_′), 8.53 (dd, *J* = 6.6, 2.6 Hz, 1H, CH_arom_), 8.01 (s, 1H, CH8′), 7.33–7.24 (m, 2H, CH_arom_), 4.55 (q, *J* = 7.3 Hz, 2H, CH_2_), 4.36 (q, *J* = 7.3 Hz, 2H, CH_2_), 4.17 (s, *J*(^1^H–^195^Pt) = 18.6 Hz, 2H, NCH_2_), 3.39 (s, *J*(^1^H–^195^Pt) = 20.1 Hz, 6H, N(CH_3_)_2_), 1.70 (t, *J* = 7.3 Hz, 3H, CH_3_), 1.62 (t, *J* = 7.3 Hz, 3H, CH_3_). ^13^C NMR (126 MHz, CD_2_Cl_2_), δ (ppm): 163.7 (C–Pt), 158.2 (C–Pt), 154.0 (C2), 153.2 (C2), 152.4 (C_quaternary_), 151.1 (C_quaternary_), 150.6 (C_quaternary_), 149.8 (C8), 145.9 (C_quaternary_), 145.6 (C_quaternary_), 143.2 (C8), 136.6 (C_quaternary_), 131.7 (C_quaternary_), 124.8 (C_arom_), 123.4 (C_arom_), 123.3 (C_arom_), 121.9 (C_quaternary_), 102.2 (C_quaternary_), 77.8 (NCH_2_), 56.3 (N(CH_3_)_2_), 40.6 (CH_2_), 39.4 (CH_2_), 15.7 (CH_3_). IR (film) ν (cm^–1^): 2927, 2083, 1604, 1572, 1210. ESI-HRMS *m*/*z*: calcd for C_25_H_26_N_9_Pt, [M + H]^+^, 647.1955; found, 647.1931.

#### Synthesis of **7a**

HNMe_2_·HCl (106 mg, 1.30 mmol), Ti(O*^i^*Pr)_4_ (0.40 mL, 1.24 mmol), and Et_3_N (0.40 mL) were added to a solution of aldehyde **6** (300 mg, 0.62 mmol) in 20 mL of ethanol. The mixture was stirred at rt for 24 h. Next, NaBH_3_CN (59 mg, 0.93 mmol) was added, and the mixture was stirred at rt for an additional 24 h. The reaction mixture was quenched with water and extracted with DCM. The organic layers were dried over Na_2_SO_4_, and the solvent was evaporated under reduced pressure to yield **7a** (colorless oil) (315 mg, 99%). ^1^H NMR (300 MHz, CDCl_3_), δ (ppm): 8.98 (s, 1H, CH2), 8.72–8.68 (m, 1H, CH_arom_), 8.62 (br s, 1H, CH_arom_), 8.34 (s, 1H, CH8), 7.57–7.52 (m, 2H, CH_arom_), 7.24–7.14 (m, 5H, CH_arom_), 6.29 (d, *J* = 2.4 Hz, 1H, CH_anomeric_), 5.39 (dd, *J* = 6.4, 2.5 Hz, 1H, CH), 5.00 (dd, *J* = 6.0, 2.5 Hz, 1H, CH), 4.58 (br s, 1H, CH), 4.47 (d, *J* = 3.9 Hz, 2H, PhCH_2_O), 3.75 −3.56 (m, 4H, CH_2_N, OC*H*_2_CH), 2.33 (s, 6H, N(CH_3_)_2_), 1.65 (s, 3H, CH_3_), 1.42 (s, 3H, CH_3_). ^13^C NMR (75 MHz, CDCl_3_), δ (ppm): 154.9 (C_quaternary_), 152.5 (C2), 151.9 (C_quaternary_), 143.2 (C8), 138.6 (C_quaternary_), 137.1 (C_quaternary_), 135.8 (C_quaternary_), 132.1 (C_arom_), 131.8 (C_quaternary_), 130.5 (C_arom_), 129.3 (C_arom_), 128.9 (C_arom_), 128.5 (C_arom_), 128.1 (C_arom_), 127.8 (C_arom_), 114.3 (C_quaternary_), 92.1 (CH_anomeric_), 86.4 (CH), 85.1 (CH), 82.2 (CH), 73.7 (Ph*C*H_2_O), 70.4 (*C*H_2_N), 64.2 (O*C*H_2_CH), 45.2 (N(*C*H_3_)_2(amina)_), 27.3 (CH_3_), 25.5 (CH_3_). IR (film) ν (cm^–1^): 2928, 1747, 1577, 1221. ESI-HRMS *m*/*z:* calcd for C_29_H_34_N_5_O_4_, [M + H]^+^, 516.2605; found, 516.2610.

#### Synthesis of **7b**

*p*-Anisidine (49 mg, 0.40 mmol) was added to a solution of aldehyde **6** (194 mg, 0.40 mmol) in 10 mL of DCM that contains MgSO_4_ (10% w/w). The mixture was stirred at room temperature for 24 h. Then, MgSO_4_ was filtered off, and the solvent was removed under reduced pressure to yield **7b** as a white solid (237 mg, 100%). ^1^H NMR (300 MHz, CDCl_3_), δ (ppm): 9.15 (t, *J* = 2.0 Hz, 1H, CH_arom_), 9.02 (s, 1H, CH2), 8.89 (dt, *J* = 8.1, 1.4 Hz, 1H, CH_arom_), 8.66 (s, 1H, CH=N), 8.38 (s, 1H, CH8), 8.18 (dt, *J* = 7.6, 1.4 Hz, 1H, CH_arom_), 7.67 (t, *J* = 7.8 Hz, 1H, CH_arom_), 7.30 (d, *J* = 8.3 Hz, 2H, CH_arom_), 7.24–7.13 (m, 5H, CH_arom_), 6.95 (d, *J* = 8.7 Hz, 2H, CH_arom_), 6.31 (d, *J* = 2.3 Hz, 1H, CH_anomeric_), 5.41 (dd, *J* = 6.1, 2.8 Hz, 1H, CH), 5.01 (dd, *J* = 6.1, 2.7 Hz, 1H, CH), 4.60 (br s, 1H, CH), 4.47 (d, *J* = 3.0 Hz, 2H, PhCH_2_O), 3.85 (s, 3H, OCH_3_), 3.74 and 3.65 (dd, *J* = 10.0, 3.4 Hz, 2H, OC*H*_2_CH), 1.66 (s, 3H, CH_3_), 1.43 (s, 3H, CH_3_). ^13^C NMR (75 MHz, CDCl_3_), δ (ppm): 158.5 (C_quaternary_), 158.2 (C=N), 154.1 (C_quaternary_), 152.6 (C2), 152.1 (C_quaternary_), 145.0 (C_quaternary_), 143.3 (C8), 137.1 (C_quaternary_), 137.1 (C_quaternary_), 136.3 (C_quaternary_), 132.5 (C_arom_), 131.8 (C_quaternary_), 130.9 (C_arom_), 130.0 (C_arom_), 129.3 (C_arom_), 128.5 (C_arom_), 128.1 (C_arom_), 127.8 (C_arom_), 122.5 (C_arom_), 114.5 (C_arom_), 114.3 (C_quaternary_), 92.3 (CH_anomeric_), 86.5 (CH), 85.2 (CH), 82.2 (CH), 73.7 (Ph*C*H_2_O), 70.4 (O*C*H_2_CH), 55.6 (OCH_3_), 27.4 (CH_3_), 25.5 (CH_3_). IR (film) ν (cm^–1^): 2953, 2836, 1748, 1241, 1221. ESI-HRMS *m*/*z:* calcd for C_34_H_34_N_5_O_5_, [M + H]^+^, 592.2554; found, 592.2547.

#### Synthesis of **8a**

A solution of **7a** (201 mg, 0.39 mmol) in 35 mL of dry toluene was bubbled with argon. Then, PtCl_2_(DMSO)_2_ (173 mg, 0.39 mmol) was added, and the mixture was refluxed under argon for 4 days. The solvent was removed under reduced pressure, and the residue was purified by flash SiO_2_ chromatography (CHCl_3_/ethyl acetate, 4:6) to yield **8a** (brilliant yellow solid) (134 mg, 49%). ^1^H NMR (500 MHz, CD_2_Cl_2_), δ (ppm): 9.35 (s, *J*(^1^H–^195^Pt) = 13.2 Hz, 1H, CH2), 8.38 (s, 1H, CH8), 8.20 (d, *J* = 7.9 Hz, 1H, CH_arom_), 7.26–7.15 (m, 6H, CH_arom_), 7.06 (d, *J* = 7.6 Hz, 1H, CH_arom_), 6.24 (d, *J* = 1.8 Hz, 1H, CH_anomeric_), 5.35 (dd, *J* = 5.8, 2.8 Hz, 1H, CH), 4.98 (dd, *J* = 6.1, 2.4 Hz, 1H, CH), 4.58 (br s, 1H, CH), 4.44 (d, *J* = 3.4 Hz, 2H, PhCH_2_O), 4.25 (s, *J*(^1^H–^195^Pt) = 22.0 Hz, 2H, NCH_2_), 3.73 and 3.62 (dd, *J* = 10.3, 2.6 Hz, 2H, OC*H*_2_CH), 3.22 (d, *J* = 5.6 Hz, *J*(^1^H–^195^Pt) = 20.4 Hz, 6H, N(CH_3_)_2_), 1.62 (s, 3H, CH_3_), 1.40 (s, 3H, CH_3_). ^13^C NMR (126 MHz, CD_2_Cl_2_), δ (ppm): 166.5 (C–Pt), 158.0 (C_quaternary_), 154.8 (C2), 152.6 (C_quaternary_), 146.1 (C8), 144.5 (C_quaternary_), 139.0 (C_quaternary_), 137.8 (C_quaternary_), 130.4 (C_quaternary_), 128.9 (C_arom_), 128.5 (C_arom_), 128.4 (C_arom_), 128.3 (C_arom_), 126.0 (C_arom_), 123.8 (C_arom_), 114.6 (C_quaternary_), 92.8 (CH_anomeric_), 87.2 (CH), 85.6 (CH), 82.7 (CH), 78.6 (Ph*C*H_2_O), 74.0 (N*C*H_2_), 71.0 (O*C*H_2_CH), 54.7 (N(*C*H_3_)_2_), 27.5 (CH_3_), 25.6 (CH_3_). IR (film) ν (cm^–1^): 2926, 1602, 1575. ESI-HRMS *m*/*z:* calcd for C_29_H_32_N_5_O_4_Pt, [M–Cl]^+^, 709.2099; found, 709.2090.

#### Synthesis of **8b**

As for **8a**, from **7a** (189 mg, 0.32 mmol) in 32 mL of dry toluene and PtCl_2_(DMSO)_2_ (141 mg, 0.32 mmol). The mixture was refluxed under argon for 5 days. The solvent was removed under reduced pressure, and the residue was purified by flash SiO_2_ chromatography (CHCl_3_/ethyl acetate, 2:8) to yield **8b** as a yellow solid (66 mg, 28%). ^1^H NMR (300 MHz, CDCl_3_), δ (ppm): 9.55 (s, *J*(^1^H–^195^Pt) = 12.3 Hz, 1H, CH8), 9.02 (s, 1H, CH2), 8.58 (dd, *J* = 7.9, 1.6 Hz, *J*(^1^H–^195^Pt) = 11.8 Hz, 1H, CH_arom_), 8.34 (s, *J*(^1^H–^195^Pt) = 58.0 Hz, 1H, CH=N), 7.65 (dd, *J* = 7.6, 1.7 Hz, 1H, CH_arom_), 7.47 (d, *J* = 8.9 Hz, 2H, CH_arom_), 7.38 (t, *J* = 7.8 Hz, 1H, CH_arom_), 7.02–6.84 (m, 7H, CH_arom_), 6.32 (d, *J* = 1.9 Hz, 1H, CH_anomeric_), 5.40 (dd, *J* = 5.9, 1.8 Hz, 1H, CH), 4.90 (d, *J* = 6.3 Hz, 1H, CH), 4.71 (br s, 1H, CH), 4.57 and 4.32 (d, *J* = 11.7 Hz, 2H, PhCH_2_O), 3.86 (s, 3H, OCH_3_), 3.78–3.59 (m, 2H, OC*H*_2_CH), 1.64 (s, 3H, CH_3_), 1.44 (s, 3H, CH_3_). ^13^C NMR (75 MHz, CDCl_3_), δ (ppm): 177.4 (C=N), 159.3 (C–Pt), 155.0 (C_quaternary_), 153.7 (C2), 150.2 (C_quaternary_), 149.2 (C_quaternary_), 147.4 (C_quaternary_), 144.7 (C8), 142.5 (C_quaternary_), 136.8 (C_quaternary_), 132.0 (C_arom_), 129.7 (C_arom_), 128.5 (C_quaternary_), 128.0 (C_arom_), 127.7 (C_arom_), 127.6 (C_arom_), 125.4 (C_arom_), 123.8 (C_arom_), 121.6 (C_quaternary_), 113.9 (C_quaternary_), 113.5 (C_arom_), 94.8 (CH_anomeric_), 87.7 (CH), 85.0 (CH), 82.6 (CH), 73.3 (Ph*C*H_2_O), 70.4 (O*C*H_2_CH), 55.6 (OCH_3_), 27.2 (CH_3_), 25.5 (CH_3_). IR (film) ν (cm^–1^): 2953, 2836, 1748, 1241, 1221. ESI-HRMS *m*/*z:* calcd for C_34_H_32_N_5_O_5_Pt, [M – Cl]^+^, 785.2048; found, 785.2025.

#### Synthesis of **10a**

Freshly prepared **9** (34 mg, 0.08 mmol) was added to a solution of 4 mg (0.08 mmol) of NaOH in 3 mL of MeOH, and the mixture was stirred at rt for 30 min. Then, **8a** (30 mg, 0.04 mmol) was added, and the reaction mixture was stirred at rt for 24 h. The solvent was removed under reduced pressure, and the residue was purified by flash SiO_2_ chromatography (CHCl_3_/ethyl acetate, 1:9) to yield **10a** as an orange solid (27 mg, 57%). ^1^H NMR (500 MHz, CD_2_Cl_2_), δ (ppm): 9.54 (s, *J*(^1^H–^195^Pt) = 9.8 Hz, 1H, CH2), 8.72 (s, 1H, CH2′), 8.37 (s, 1H, CH8), 8.27 (d, *J* = 7.2 Hz, 1H, CH_arom_), 8.24 (s, 1H, CH8′), 7.35–7.14 (m, 12H, CH_arom_), 6.23 (d, *J* = 2.4 Hz, 1H, CH_anomeric_), 6.21 (d, *J* = 2.4 Hz, 1H, CH_anomeric_), 5.37–5.33 (m, 2H, 2CH), 5.01 (dd, *J* = 5.7, 2.1 Hz, 1H, CH), 4.96 (dd, *J* = 5.7, 2.3 Hz, 1H, CH), 4.58 (br s, 1H, CH), 4.53–4.51 (br s, 1H, CH), 4.49 (d, *J* = 9.2 Hz, 2H, PhCH_2_O), 4.43 (d, *J* = 3.4 Hz, 2H, PhCH_2_O), 4.38 (br s, 2H, NCH_2_), 3.74–3.69 (m, 2H, OC*H*_2_CH), 3.63–3.59 (m, 2H, OC*H*_2_CH), 3.43 (d, *J* = 6.0 Hz, *J*(^1^H–^195^Pt) = 13.2 Hz, 6H, N(CH_3_)_2_), 1.63 (s, 3H, CH_3_), 1.62 (s, 3H, CH_3_), 1.39 (s, 3H, CH_3_) and 1.38 (s, 3H, CH_3_). ^13^C NMR (126 MHz, CD_2_Cl_2_), δ (ppm): 176.4 (C–Pt), 138.5 (C–Pt), 157.7 (C2), 156.0 (C_quaternary_), 153.1 (C2), 152.3 (C_quaternary_), 150.8 (C_quaternary_), 147.2 (C_quaternary_), 145.9 (C8), 145.7 (C_quaternary_), 142.7 (C8), 141.6 (C_quaternary_), 138.2 (C_quaternary_), 137.7 (C_quaternary_), 135.7 (C_quaternary_), 130.8 (C_quaternary_), 128.9 (2C_arom_), 128.5 (C_arom_), 128.4 (2C_arom_), 128.3 (2C_arom_), 125.5 (C_arom_), 124.6 (C_arom_), 114.5 (2C_quaternary_), 106.9 (C_quaternary_), 93.0 and 92.0 (CH_anomeric_), 87.2 and 86.7 (CH), 85.7 and 85.4 (CH), 82.7 (2CH), 80.8 (N*C*H_2_), 74.0 (2Ph*C*H_2_O), 71.0 and 70.9 (CH_2_), 56.7 (N(*C*H_2_)_3_), 27.5, 27.4, 25.7, and 25.6 (CH_3_). IR (film) ν (cm^–1^): 2074, 1574, 1479. ESI-HRMS *m*/*z:* calcd for C_51_H_54_N_9_O_8_Pt, [M + H]^+^, 1115.3741; found, 1115.3737.

#### Synthesis of **10b**

As for **10a**, from a solution of 4 mg (0.09 mmol) of NaOH in 3 mL of MeOH, 35 mg (0.09 mmol) of **9**, and 35 mg (0.04 mmol) of **8b**. Purification of the crude product by SiO_2_ flash chromatography (CHCl_3_/ethyl acetate, 5:95 and 1% MeOH) yielded **10b** (orange solid) (21 mg, 41%). ^1^H NMR (500 MHz, CD_2_Cl_2_), δ (ppm): 9.96 (s, *J*(^1^H–^195^Pt) = 12.2 Hz, 1H, CH8), 9.00 (s, 1H, CH2), 8.75 (br s, 1H, CH_arom_), 8.74 (s, 1H, CH2′), 8.61 (s, *J*(^1^H–^195^Pt) = 54.7 Hz, 1H, CH=N), 8.26 (s, 1H, CH8′), 7.79–7.75 (m, 3H, CH_arom_), 7.43 (t, *J* = 7.3 Hz, 1H, CH_arom_), 7.30–7.22 (m, 5H, CH_arom_), 6.99–6.87 (m, 7H, CH_arom_), 6.35 (d, *J* = 2.0 Hz, 1H, CH_anomeric_), 6.22 (d, *J* = 2.0 Hz, 1H, CH_anomeric_), 5.52 (d, *J* = 5.5 Hz, 1H, CH), 5.37 (dd, *J* = 7.1, 2.7 Hz, 1H, CH), 5.01 (dd, *J* = 6.0, 2.0 Hz, 2H, 2CH), 4.60 (br s, 1H, CH), 4.56–4.52 (m, 1H, CH), 4.51–4.29 (m, 4H, PhCH_2_O), 3.80 (s, 3H, OCH_3_), 3.79 (dd, *J* = 10.6, 3.0 Hz, 1H, OC*H*_2_CH), 3.71 (dd, *J* = 10.6, 3.0 Hz, 1H, OC*H*_2_CH), 3.63 (dd, *J* = 10.6, 3.9 Hz, 1H, OC*H*_2_CH), 3.47 (dd, *J* = 10.6, 3.9 Hz, 1H, OC*H*_2_CH), 1.63 (s, 6H, CH_3_), 1.44 (s, 3H, CH_3_) and 1.40 (s, 3H, CH_3_). ^13^C NMR (126 MHz, CD_2_Cl_2_), δ (ppm): 179.7 (C=N), 170.6 (C–Pt), 159.8 (C–Pt), 157.2 (C_quaternary_), 154.0 (C2), 153.2 (C2), 150.9 (C_quaternary_), 150.5 (C_quaternary_), 149.4 (C_quaternary_), 148.5 (C8), 145.5 (C_quaternary_), 144.9 (C_quaternary_), 142.7 (C8), 138.1 (C_quaternary_), 137.7 (C_quaternary_), 135.9 (C_quaternary_), 132.0 (C_arom_), 131.0 (C_quaternary_), 129.1 (C_arom_), 129.0 (C_arom_), 128.5 (C_arom_), 128.4 (C_arom_), 128.3 (C_arom_), 128.1 (C_quaternary_), 128.1 (C_arom_), 127.8 (C_arom_), 126.0 (C_arom_), 125.0 (C_arom_), 122.3 (C_quaternary_), 114.6 (C_quaternary_), 114.1 (C_quaternary_), 113.9 (C_arom_), 105.5 (C_quaternary_), 95.0 and 92.0 (CH_anomeric_), 88.5 and 86.6 (CH), 86.0 and 85.3 (CH), 83.3 and 82.7 (CH), 74.0 and 73.4 (PhCH_2_O), 70.9 and 70.7 (O*C*H_2_CH), 56.0 (OCH_3_), 27.5 and 27.4 (CH_3_), 25.7 (2CH_3_). IR (film) ν (cm^–1^): 2927, 2083, 1604, 1572. ESI-HRMS *m*/*z:* calcd for C_56_H_54_N_9_O_9_Pt, [M + H]^+^, 1192.3705; found, 1192.3701.

## Supporting Information Available

Synthesis and characterization of compounds **6** and **9**; computed frontier orbitals and their energies; XRD data, absorption, and emission spectra and NMR spectra (PDF)
Cartesian coordinates (TXT)

## Notes

^†^ Dedicated to Prof. Joaquín Tamariz from Instituto Politécnico Nacional (México) on the occasion of his retirement.
