# Supplementary material for: Synthesis, Structure, and Photophysical Properties of Platinum(II) (N,C,N′) Pincer Complexes Derived from Purine Nucleobases†
Source: Inorg Chem. 2023 May 18;62(21):8232–48. doi: 10.1021/acs.inorgchem.3c00650 (PMC10230507; doi:10.1021/acs.inorgchem.3c00650)
Supplement: Supplementary file 1 — ic3c00650_si_001.pdf [file ic3c00650_si_001.pdf]

SUPPORTING INFORMATION

Belonging to the manuscript:

Synthesis, Structure and Photophysical Properties of Platinum (II) (N,C,N') Pincer Complexes Derived from Purine Nucleobases

Carmen Lorenzo-Aparicio,[a,d] *Sonia Moreno-Blázquez,*[b,d] *Montserrat Oliván,*[b,d] *Miguel A. Esteruelas,*[b,d] *Mar Gómez Gallego,**[*a,d] *Pablo García-Álvarez,*[c,d] *Javier A. Cabeza,*[c,d] and *Miguel A. Sierra** [a,d]

[a] Departamento de Química Orgánica, Facultad de Ciencias Químicas, Universidad Complutense, 28040 Madrid, Spain. [b] Departamento de Quımica Inorgánica, Instituto de Síntesis Química y Catálisis Homogénea (ISQCH), Universidad de Zaragoza-CSIC, 50009 Zaragoza, Spain. [c]Departamento de Química Orgánica e Inorgánica, Facultad de Química, Universidad de Oviedo, 33071 Oviedo, Spain. [d] Center for Innovation in Advanced Chemistry (ORFEO-CINQA).

Index

S.1. Synthesis of compounds 6 and 9 **S2**

S.2. Crystal data **S6**

S.3. Computational studies of* [5.5]- and [6.5]-[Pt(N^C^N´)Cl] *isomers **S15**

S.4. Absorption spectra **S17**

S.5. HOMO and LUMO orbitals of complexes 3, 4 and 5 **S21**

S.6. Emission Studies **S26**

S.7. NMR spectra **S61**

S.8. References **S87**

S.1. Synthesis of compounds 6 and 9

Compound **6** was prepared according to the following sequence of reactions (Scheme S1). 9-(2,3-*O*-Isopropylidene-β-D-ribofuranosyl)-6-chloropurine (**S1**) was prepared according to the literature procedure.[S1]

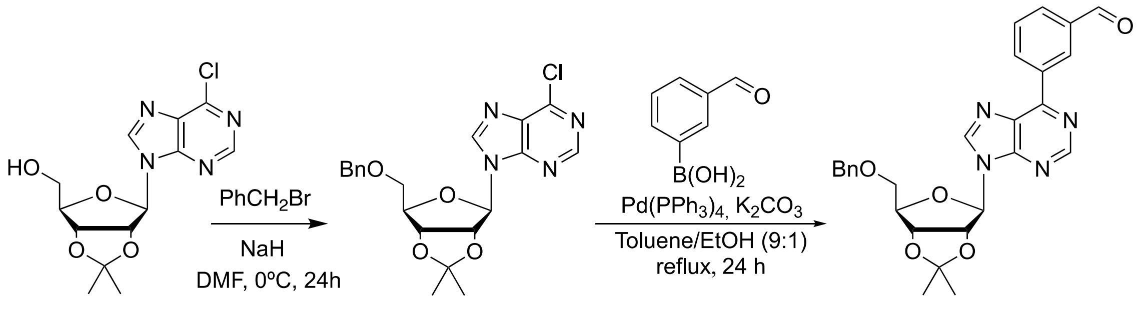

S1 **S2** (45%) **6** (98%)

Scheme S1

Synthesis of S2

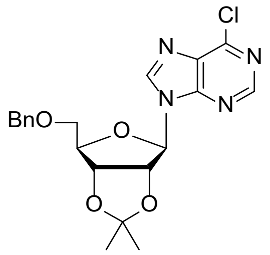

Benzyl bromide (0.50 mL, 4.18 mmol) was added to a solution of 9-(2,3-*O*-isopropylidene-β-D-ribofuranosyl)-6-chloropurine (**S1**) (805 mg, 2.46 mmol) in 15 mL of dry DMF. The solution was cooled to 0°C before the addition of NaH (65 mg, 2.72 mmol). The mixture was stirred at rt for 4 h, cooled to 0°C, treated with 20 mL of water and subsequently extracted with ethyl acetate. The organic layer was dried over Na_2SO_4, filtered and the solvent removed under reduced pressure. The crude product was purified by flash SiO_2 chromatography (hexane, to hexane/ethyl acetate 1:1) to yield **S2** (colorless oil) (461 mg, 45%). ^{1}H-NMR (300 MHz, $CDCl_3$), δ (ppm): 8.70 (s, 1H, CH*2*), 8.36 (s, 1H, CH*8*), 7.25 – 7.09 (m, 5H, CH_{arom}), 6.23 (d, *J* = 6.1 Hz, 1H, $CH_{anomeric}$), 5.32 (dd, *J* = 6.1, 2.1 Hz, 1H, CH), 4.95 (dd, *J* = 6.1, 1.9 Hz, 1H, CH), 4.61 (br s, 1H, CH), 4.41 (br s, 2H, $PhCH_2O$), 3.71 and 3.62 (dd, *J* = 9.9, 3.0 Hz, 2H, OCH_2CH), 1.63 (s, 3H, CH_3), 1.41 (s, 3H, CH_3). ^{13}C-NMR (75 MHz, $CDCl_3$), δ (ppm): 152.1 (C*2*), 151.1 ($C_{quaternary}$), 143.9 (C*8*), 136.8 ($C_{quaternary}$), 132.3 ($C_{quaternary}$), 128.6 ($C_{quaternary}$), 128.6 (C_{arom}), 128.2 (C_{arom}), 127.8 (C_{arom}), 114.3 ($C_{quaternary}$), 92.8 ($CH_{anomeric}$), 86.6 (CH), 85.3 (CH), 82.2 (CH), 73.8

(Ph*C*H_2O), 70.4 (O*C*H_2CH), 27.3 (CH_3), 25.5 (CH_3). IR (film) ν (cm^{-1}): 2919, 1611, 1569, 1498. ESI-HRMS *m/z:* Calc for $C_{20}H_{22}N_4O_4Cl$, $[M+H]^+$, 417.1324; found, 417.1328.

Synthesis of 6

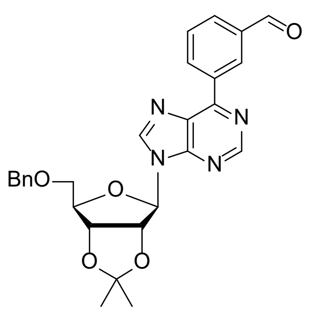

3-Formylphenylboronic acid (302 mg, 1.56 mmol), $Pd(PPh_3)_4$ (81 mg, 0.07 mmol) and K_2CO_3 (215 mg, 1.56 mmol) were added to a solution of **S2** (433 mg, 1.04 mmol) in 20 mL of degasified (9:1) toluene/ethanol. The mixture was refluxed for 24 h, cooled to rt and the solvent was removed under reduced pressure. The crude product was purified by flash SiO_2 chromatography (hexane to hexane/ethyl acetate 1:1) to yield **6** (colorless oil) (495 mg, 98%). ^{1}H-NMR (300 MHz, $CDCl_3$), δ (ppm): 10.2 (s, 1H, CHO), 9.29 (t, J = 2.0 Hz, 1H, CH_{arom}), 9.08 (dt, J = 7.7, 1.6 Hz, 1H, CH_{arom}), 9.03 (s, 1H, CH*2*), 8.39 (s, 1H, CH*8*), 8.07 (dt, J = 7.7, 1.6 Hz, 1H, CH_{arom}), 7.74 (t, J = 7.8 Hz, 1H, CH_{arom}), 7.23 – 7.11 (m, 5H, CH_{arom}), 6.31 (d, J = 2.3 Hz, 1H, $CH_{anomeric}$), 5.40 (dd, J = 5.6, 2.5 Hz, 1H, CH), 5.00 (dd, J = 6.2, 2.5 Hz, 1H, CH), 4.62 (br s, 1H, CH), 4.47 (br s, 2H, PhC*H_2*O), 3.75 and 3.66 (dd, J = 9.8, 2.4 Hz, 2H, OC*H_2*CH), 1.66 (s, 3H, CH_3), 1.43 (s, 3H, CH_3). ^{13}C-NMR (75 MHz, $CDCl_3$), δ (ppm): 192.3 (CHO), 153.1 ($C_{quaternary}$), 152.5 (C*2*), 152.2 ($C_{quaternary}$), 143.7 (C*8*), 137.1 ($C_{quaternary}$), 137.0 ($C_{quaternary}$), 136.7 ($C_{quaternary}$), 135.7 (C_{arom}), 132.4 (C_{arom}), 131.8 ($C_{quaternary}$), 130.8 (C_{arom}), 129.6 (C_{arom}), 128.5 (C_{arom}), 128.1 (C_{arom}), 127.8 (C_{arom}), 114.4 ($C_{quaternary}$), 92.4 ($CH_{anomeric}$), 86.5 (CH), 85.2 (CH), 82.2 (CH), 73.7 (Ph*C*H_2O), 70.5 (O*C*H_2CH), 27.4 (CH_3), 25.5 (CH_3). IR (film) ν (cm^{-1}): 3451, 1695, 1571, 1233. ESI-HRMS *m/z:* Calc for $C_{27}H_{27}N_4O_5$, $[M+H]^+$, 487.1976; found, 487.1952.

Synthesis of alkyne 9

Compound **9** was prepared from **S2** according to the following sequence of reactions (Scheme S2).

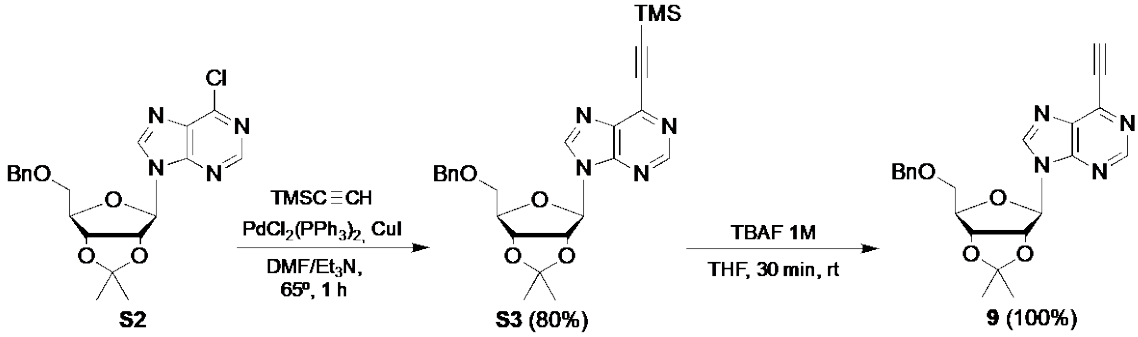

Scheme S2

Synthesis of S3

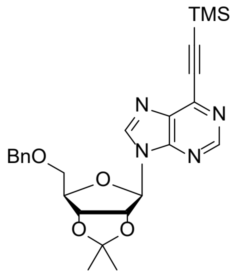

Ethynyltrimethylsilane (0.20 mL, 1.30 mmol) was added to a solution of **S2** (400 mg, 1.08 mmol), $PdCl_2(PPh_3)_2$ (14.37 mg, 0.03 mmol) and CuI (12 mg, 0.07 mmol) in 5 mL of previously degasified DMF/Et_3N (2:1). The mixture was heated at 65 °C for 1 h, cooled to rt and the solvent was removed under reduced pressure. The crude product was purified by flash SiO_2 chromatography (hexane/ethyl acetate 1:1) to yield **S3** (yellow oil) (412 mg, 80%). ^{1}H-NMR (300 MHz, $CDCl_3$), δ (ppm): 8.87 (s, 1H, CH*2*), 8.36 (s, 1H, CH*8*), 7.27–7.12 (m, 5H, CH_{arom}), 6.23 (d, J = 2.4 Hz, 1H, $CH_{anomeric}$), 5.34 (dd, J = 6.0, 2.4 Hz, 1H, CH), 4.95 (dd, J = 6.1, 2.1 Hz, 1H, CH), 4.58 (br s, 1H, CH), 4.43 (d, J = 2.6 Hz, 2H, $PhCH_2O$), 3.68 (dd, J = 10.2, 3.9 Hz, 1H, OCH_2CH), 3.58 (dd, J = 10.2, 3.9 Hz, 1H, OCH_2CH), 1.64 (s, 3H, CH_3), 1.41 (s, 3H, CH_3), 0.34 (s, 9H, $Si(CH_3)_3$). ^{13}C-NMR (75 MHz, $CDCl_3$), δ (ppm): 152.2 (C*2*), 143.7 (C*8*), 136.5 ($C_{quaternary}$), 128.1 (C_{arom}), 127.7 (C_{arom}), 127.4 (C_{arom}), 123.4 ($C_{quaternary}$), 113.8 ($C_{quaternary}$), 108.6 ($C_{quaternary}$), 91.8 ($CH_{anomeric}$), 86.0 (CH), 84.6 (CH), 81.7 (CH), 73.2 ($PhCH_2O$), 69.8 (OCH_2CH), 26.8 (CH_3), 25.0 (CH_3), -0.7 ($Si(CH_3)_3$). IR (film) ν (cm^{-1}): 2968, 1741, 1568, 1207. ESI-HRMS *m/z:* Calc for $C_{25}H_{31}N_4O_4Si$, $[M+H]^+$, 476.2109; found, 479.2125.

Synthesis of 9

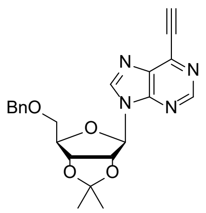

A solution of TBAF (1M in THF) (0.4 mL, 0.4 mmol) was added dropwise to a solution of **S3** (165 mg, 0.34 mmol) in 5 mL THF at 0 °C. The mixture was stirred at rt for 20 min, quenched with saturated NH_4Cl solution and extracted with ethyl acetate. The organic layer was dried over Na_2SO_4, filtered and the solvent removed under

reduced pressure. The crude product was purified by flash SiO_2 chromatography (hexane/ethyl acetate 1:1) to yield **9** (pale brown oil) (142 mg, 100%). ^{1}H-NMR (300 MHz, $CDCl_3$), δ (ppm): 8.90 (s, 1H, CH*2*), 8.37 (s, 1H, CH*8*), 7.29–7.10 (m, 5H, CH_{arom}), 6.25 (d, *J* = 2.4, 1H, $CH_{anomeric}$), 5.33 (dd, *J* = 6.0, 2.4 Hz, 1H, CH), 4.96 (dd, *J* = 6.1, 2.0 Hz, 1H, CH), 4.60 (br s, 1H, CH), 4.43 (s, 2H, $PhCH_2O$), 3.73–3.58 (m, 2H, OCH_2CH), 3.71 (s, 1H, CH alkyne), 1.64 (s, 3H, CH_3), 1.41 (s, 3H, CH_3). ^{13}C-NMR (75 MHz, $CDCl_3$), δ (ppm): 152.7 (C*2*), 151.1 ($C_{quaternary}$), 144.4 (C*8*), 140.6 ($C_{quaternary}$), 136.9 ($C_{quaternary}$), 135.6 ($C_{quaternay}$), 128.5 (C_{arom}), 128.2 (C_{arom}), 127.8 (C_{arom}), 114.3 ($C_{quaternary}$), 92.4 ($CH_{anomeric}$), 86.5 (CH_{alkyne}), 86.2 (CH), 85.2 (CH), 82.2 (CH), 73.7 ($PhCH_2O$), 70.4 (OCH_2CH), 27.3 (CH_3), 25.4 (CH_3). IR (film) ν (cm^{-1}): 2851, 1740, 1518, 1026. ESI-HRMS *m/z:* Calc for $C_{22}H_{23}N_4O_4$, $[M+H]^+$, 407.1714; found, 407.1695.

Table S1. Selected Interatomic Distances (Å) and Angles (º) in Compounds **3a**, **5a**, **3b**, **4b**, **3c**, **3d** and **4d**

Bond	**3a**[a]	**5a**	**3b**	**4b**	**3c**[a]	**3d**[a]	**4d**
C9–Pt1	1.923(5)	1.967(6)	2.001(4)	2.025(3)	1.983(6)	1.992(6)	2.05(1)
N1–Pt1	2.045(4)	2.039(5)					
N7–Pt1			2.010(3)	1.994(3)	1.993(5)	1.990(5)	2.02(1)
N8–Pt1	2.082(5)	2.088(6)	2.025(4)	2.027(3)	2.029(5)	2.068(5)	2.08(1)
Cl1–Pt1	2.408(1)		2.382(1)		2.400(1)	2.383(2)	
C17–Pt1		2.063(6)		2.042(3)			2.05(1)
C9–Pt1–N1	80.3(2)	80.4(2)					
C9–Pt1–N7			92.0(2)	91.4(1)	92.0(2)	92.0(2)	91.7(5)
C9–Pt1–N8	82.5(2)	82.4(3)	81.1(2)	80.5(1)	82.1(2)	83.0(2)	83.0(5)
N1–Pt1–N8	162.2(2)	162.8(2)					
N7–Pt1–N8			173.2(1)	171.8(1)	174.0(2)	173.7(2)	171.6(4)
C9–Pt1–Cl1(C17)	176.8(2)	176.9(3)	176.0(1)	176.8(1)	177.5(2)	177.0(2)	176.3(5)
N1–Pt1–Cl1(C17)	100.0(1)	101.5(2)					
N7–Pt1–Cl1(C17)			90.6(1)	91.3(0)	90.4(1)	90.3(2)	90.1(4)
N8–Pt1–Cl1(C17)	97.0(1)	95.7(2)	96.2(1)	96.8(1)	95.5(2)	94.6(2)	95.6(4)
Pt···Pt[b]	3.835, 3.861	8.66	6.750	5.842	4.279	5.156	5.882
Interplanar distance	3.3(3), 3.4(3), 3.6(3)[c]	3.564[d]	3.341[d]	3.468[d]	3.48(5), 3.38(3)[e]	3.3(3), 3.8(3)[e]	3.464[d]

[a]Distances and angles for one (Pt1) of the four (for **3a**) or of the two (for **3c**, **3d**) analogous molecules found in the asymmetric unit.
[b]Closest Pt···Pt contacts in the crystal.
[c]Distances between the mean planes of the four 6-phenylpurine scaffolds of the groups of 4 molecules found in the crystal.
[d]Distance between the mean planes of the 6-phenylpurine scaffolds of contiguous molecules.
[e]Distances between the mean planes of the 6-phenylpurine scaffolds of the two molecules found in the asymmetric unit and of the molecules of two different asymmetric units, respectively.

S.2. Crystal data

Table S2. Crystal, measurement and refinement data for the compounds studied by X-ray diffraction.

	**3a**·0.5($CHCl_3$)	**3b**	**3c**·0.5(CH_2Cl_2)	**3d**	**4b**·(C_7H_8)	**4d**	**5a**
formula	4($C_{16}H_{18}ClN_5Pt$)· 2($CHCl_3$)	$C_{21}H_{18}ClN_5OPt$	2($C_{18}H_{14}ClN_5Pt$) (CH_2Cl_2)	2($C_{16}H_{18}ClN_5Pt$)	$C_{29}H_{23}N_5OPt$	$C_{24}H_{23}N_5Pt$	$C_{25}H_{25}N_9Pt$
fw	2282.31	586.94	1146.69	1021.79	652.61	576.56	646.63
cryst syst	Monoclínico	Monoclínico	Monoclínico	Triclínico	Ortorrómbico	Ortorrómbico	Monoclínico
space group	$P2_1/c$	$P2_1/n$	$P2_1/c$	$P-1$	$Pbca$	$P2_12_12_1$	$P2_1/n$
a, Å	14.0169(6)	6.7504(1)	18.9027(3)	10.0903(3)	22.6150(2)	5.8821(1)	15.6342(4)
b, Å	25.5681(3)	24.2694(4)	6.9784(1)	11.2333(3)	8.4290(1)	14.0560(2)	8.2731(3)
c, Å	28.382(1)	11.4756(2)	26.5747(5)	15.6863(4)	24.9233(3)	24.6859(4)	18.3177(5)
α, deg	90	90	90	75.413(2)	90	90	90
β, deg	133.044(7)	95.999(2)	90.972(2)	89.939(2)	90	90	101.840(3)
γ, deg	90	90	90	70.533(3)	90	90	90
V, Å^3	7433.9(8)	1869.73(5)	3505.0(1)	1615.62(8)	4750.92(9)	2041.00(6)	2318.9(1)
Z	4	4	4	2	8	4	4
F(000)	4368	1128	2184	976	2544	1120	1264
D_{calcd}, g cm^{-3}	2.039	2.085	2.173	2.100	1.825	1.876	1.852
μ, mm^{-1} (Cu Kα)	17.511	15.555	17.899	17.815	11.314	13.021	11.595
cryst size, mm	0.20 x 0.20 x 0.20	0.13 x 0.09 x	0.19 x 0.15 x	0.28 x 0.13 x	0.19 x 0.15 x	0.37 x 0.06 x	0.29 x 0.11 x
T, K	150(2)	150(2)	150(2)	150(2)	150(2)	150(2)	150(2)
θ range, deg	3.42 a 69.57	3.64 a 69.39	3.33 a 69.58	2.92 a 69.59	3.55 a 69.63	3.58 a 69.57	3.39 a 69.60
min./max. h, k, l	−17/16, −30/30, −34/28	−7/8, −29/28, −13/7	−19/22, −6/8, −26/32	−12/11, −12/13, −15/18	−24/27, −7/10, −30/26	−7/4, −16/16, −28/29	−18/18, −8/9, −22/21
no. collected reflns	42990	9113	17968	13491	25003	17718	11688
no. unique reflns	13802	3458	6525	5992	4427	3810	4285
no. reflns with $I > 2\sigma(I)$	12369	3072	5653	5296	3941	3670	3684
no. params/restraints	913/0	264/0	480/0	421/0	327/0	275/0	326/0
GOF (on F^2)	1.023	0.981	1.112	1.034	1.050	1.104	1.047
R_1 (on F, $I > 2\sigma(I)$)	0.039	0.024	0.035	0.037	0.025	0.041	0.045
wR_2 (on F^2, all data)	0.092	0.065	0.104	0.104	0.058	0.104	0.123

min./max. $\Delta\rho$, e Å^{-3}	−0.937/2.284	−0.447/0.731	−0.697/2.410	−2.074/1.232	−1.118/0.650	−1.221/2.287	−1.190/1.548
CCDC dep. no.	2242639	2242640	2242641	2242642	2242643	2242644	2242645

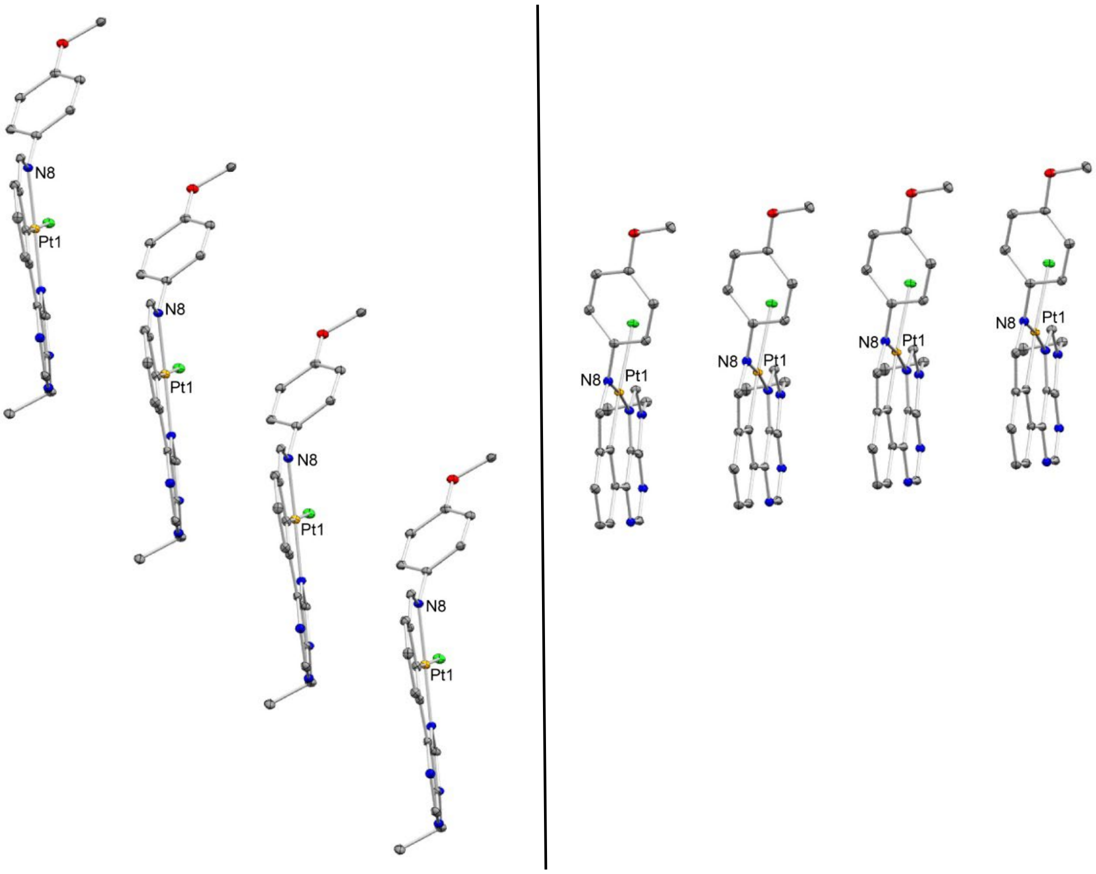

Figure S1. Two views (separated by a vertical line) of the molecular arrangement of **3b** in the crystal (10% displacement ellipsoids) showing the parallel disposition of the planes defined by the 6-phenylpurine scaffolds (separated by 3.341 Å). This parallel orientation is maintained along the entire crystal lattice. The shortest Pt···Pt distance is 6.750 Å. The asymmetric unit contains only one molecule.

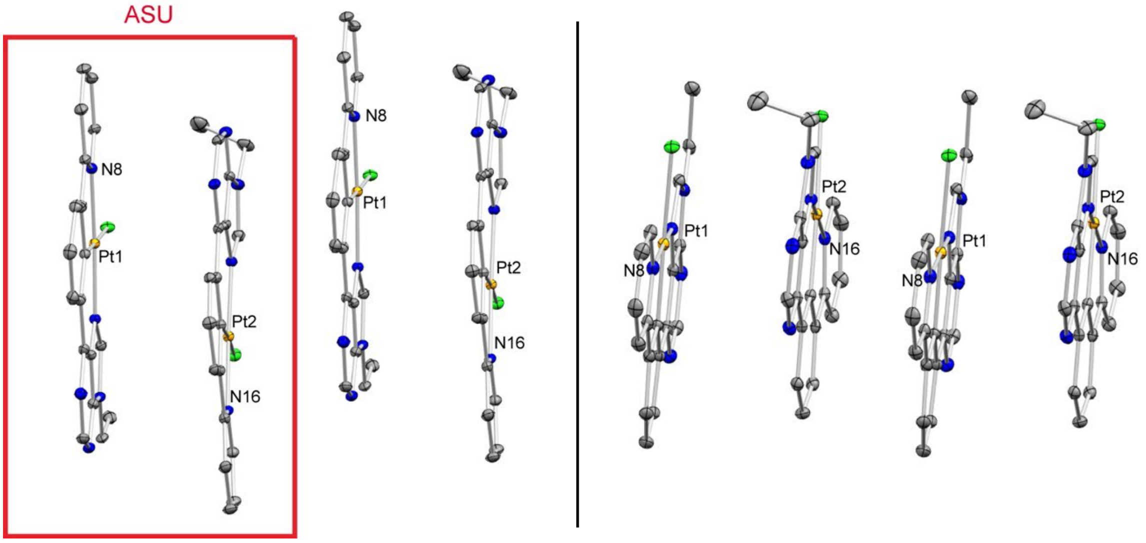

Figure S2. Two views (separated by a vertical line) of the molecular arrangement of **3c** in the crystal (10% displacement ellipsoids), showing the approximate parallel disposition of the planes defined by the 6-phenylpurine scaffolds [separated by 3.48(5) Å (within the asymmetric unit) and 3.38(5) Å (between molecules of different asymmetric units)]. This approximate parallel orientation is maintained along the entire crystal lattice. The shortest Pt···Pt distances are 4.279 Å (between the molecules of the asymmetric unit) and 5.141 Å (between molecules of different asymmetric units). The asymmetric unit (ASU) contains two molecules.

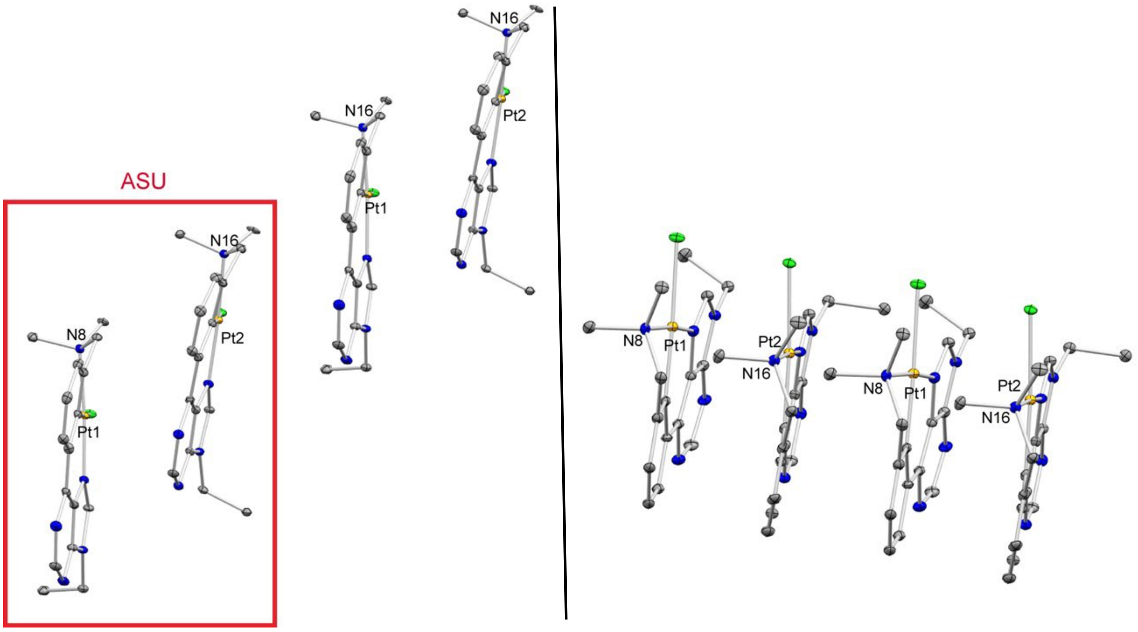

Figure S3. Two views (separated by a vertical line) of the molecular arrangement of **3d** in the crystal (10% displacement ellipsoids), showing the approximate parallel disposition of the planes defined by the 6-phenylpurine scaffolds, which are separated by 3.3(3) Å (within the asymmetric unit) and 3.8(3) Å (between different asymmetric units). This approximate parallel disposition is maintained along the entire crystal lattice. The shortest Pt···Pt distances are 5.156 Å (within the asymmetric unit) and 6.089 Å (between different asymmetric units). The asymmetric unit (ASU) contains two molecules.

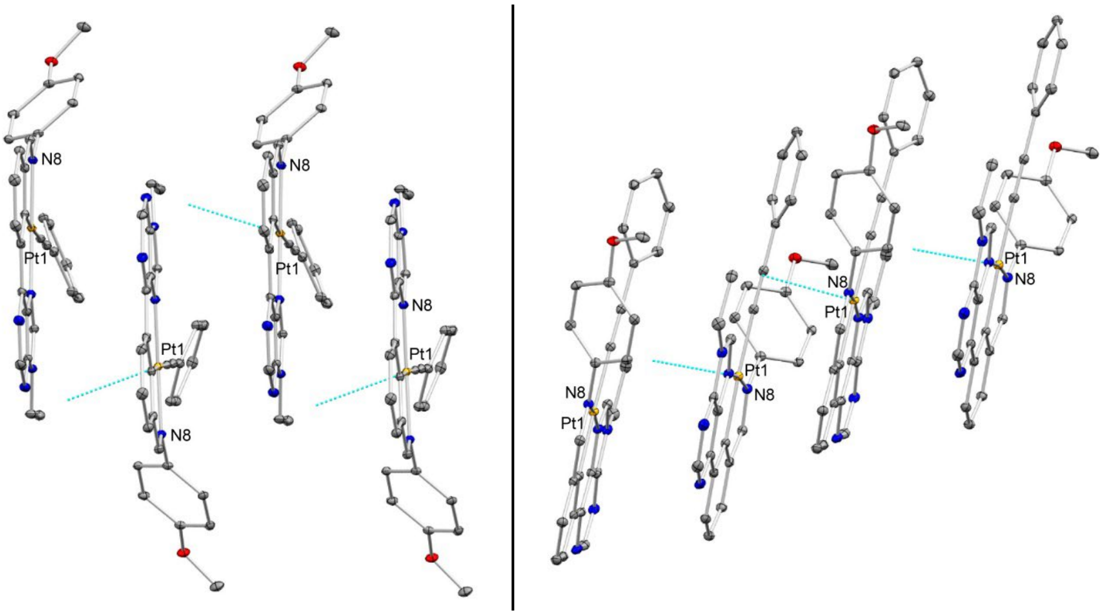

Figure S4. Two views (separated by a vertical line) of the molecular arrangement of **4b** in the crystal (10% displacement ellipsoids), showing the parallel disposition of the planes defined by the 6-phenylpurine scaffolds (separated by 3.468 Å). This parallel orientation is maintained along the entire crystal lattice. The The shortest Pt···Pt distance is 5.842 Å. The asymmetric unit contains only one molecule.

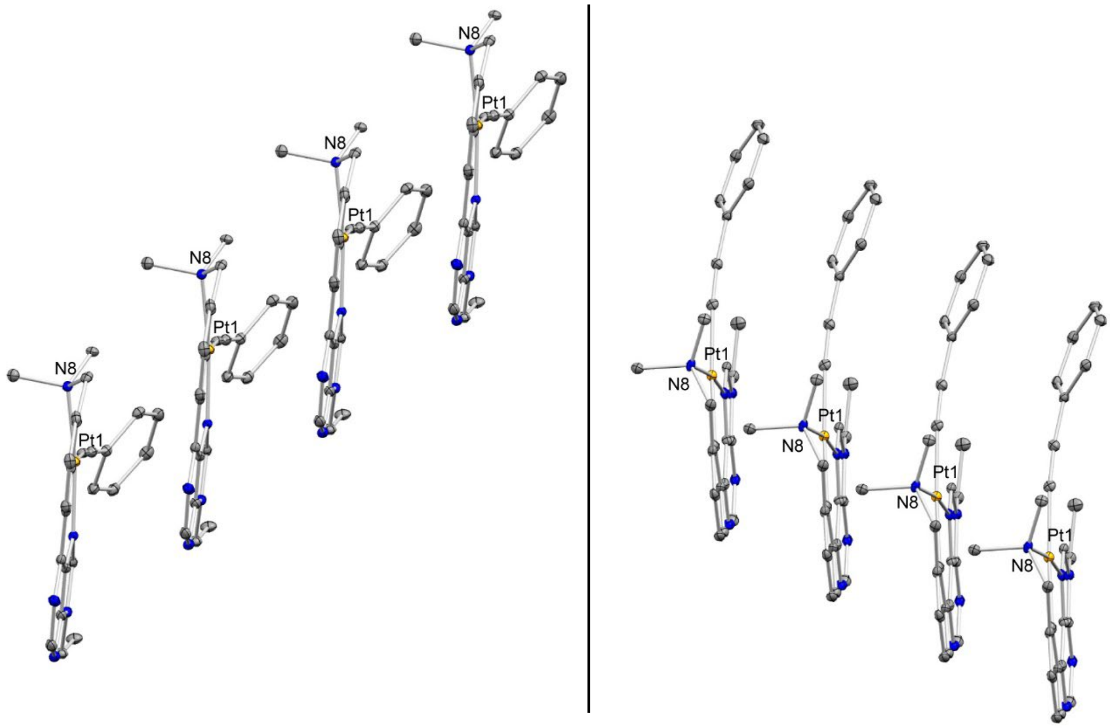

Figure S5. Two views (separated by a vertical line) of the molecular arrangement of **4d** in the crystal (10% displacement ellipsoids), showing the parallel disposition of the planes defined by the 6-phenylpurine scaffolds (separated by 3.464 Å). This parallel orientation is maintained along the entire crystal lattice. The shortest Pt···Pt distance is 5.882 Å. The asymmetric unit contains only one molecule.

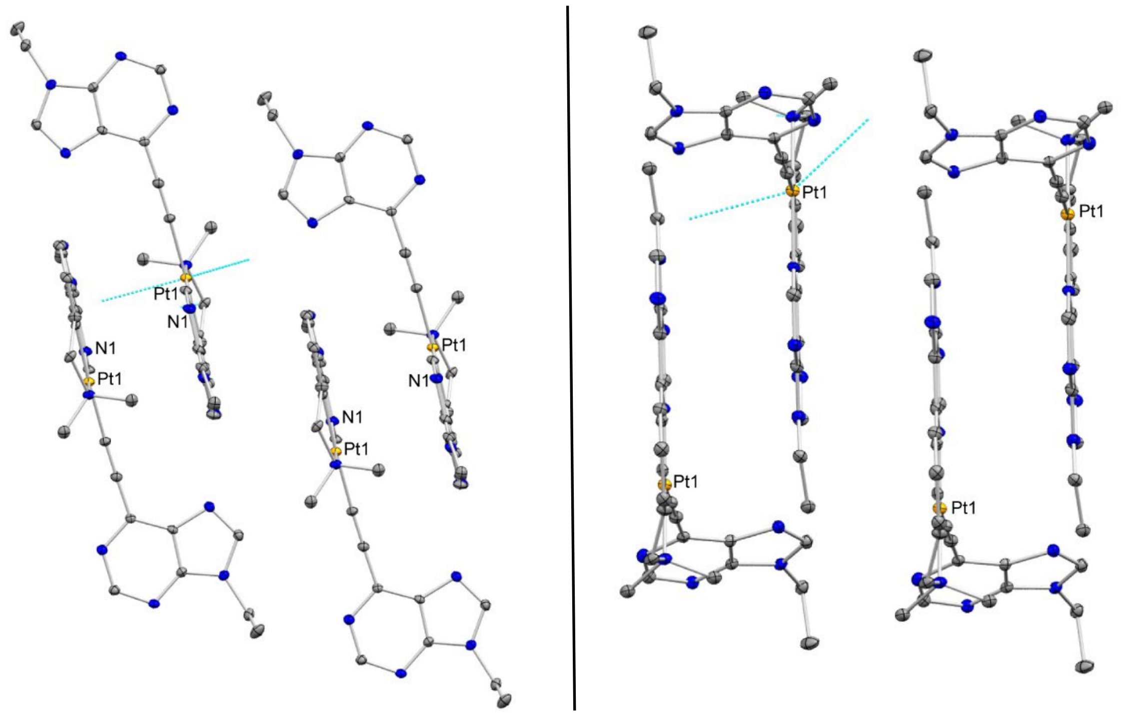

Figure S6. Two views (separated by a vertical line) of the molecular arrangement of **5a** in the crystal (10% displacement ellipsoids), showing the parallel disposition of the planes defined by the 6-phenylpurine scaffolds (separated by 3.564 Å). This parallel orientation is maintained along the entire crystal lattice. The The shortest Pt···Pt distance is 8.66 Å. The asymmetric unit contains only one molecule.

S.3. Computational studies of **[5.5]- and [6.5]-[Pt(N^C^N´)Cl]** *isomers*

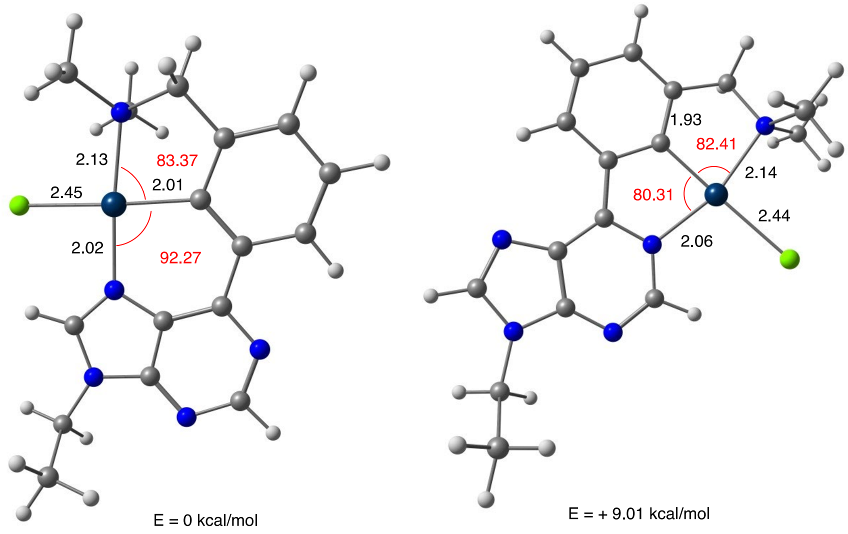

Figure S7. Computed structures (B3LYP-D3/def2-SVP, gas phase) of the possible [6.5]- and [5.5]-[Pt(N^C^N)Cl] pincer complexes derived from ligand **2a**. Bond distances (Å) in black. Angles (deg) in red.

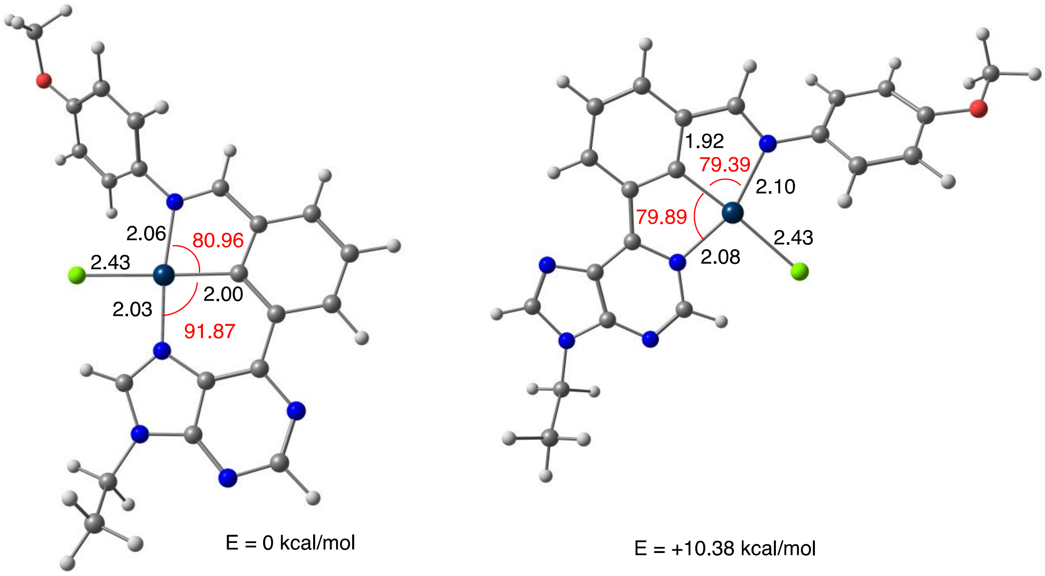

Figure S8. Computed structures (B3LYP-D3/def2-SVP, gas phase) of the possible [6.5]- and [5.5]-[Pt(N^C^N)Cl] pincer complexes derived from ligand **2b**. Bond distances (Å) in black. Angles (deg) in red.

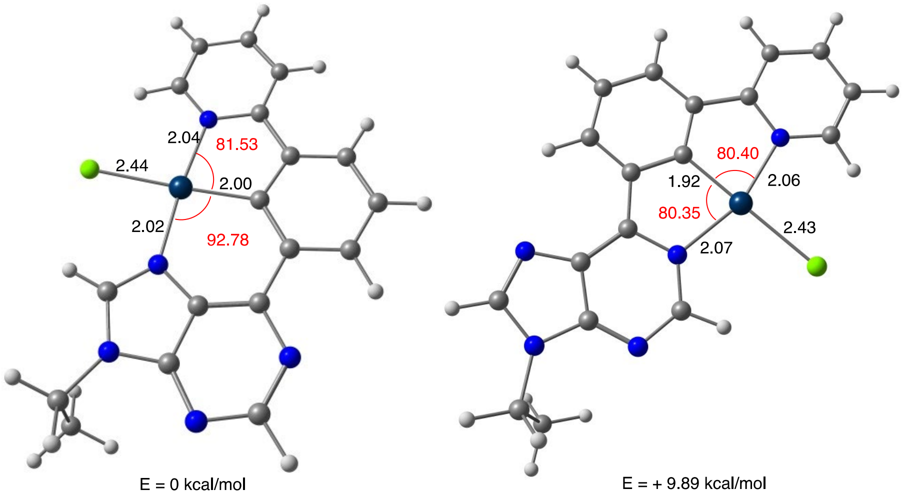

Figure S9. Computed structures (B3LYP-D3/def2-SVP, gas phase) of the possible [6.5]- and [5.5]-[Pt(N^C^N)Cl] pincer complexes derived from ligand **2c**. Bond distances (Å) in black. Angles (deg) in red.

S.4. Absorption spectra

Table S3. Selected experimental UV-vis absorption data for complexes **3a, 4a** and **5a** (10^{-5} M), computed TD-DFT vertical excitation energies (in DCM) and their major contributions.*

**Complex**	**λ_{max}(nm)**	**ε($M^{-1}cm^{-1}$)**	**excitation energy***	**oscillator strength *f***	**transition contribution(%)**	**character of the transition**
**3a**	515	110	529 (T_1)	0	H-L (67)	IL/MLCT
	445	10141	453 (S_1)	0.0964	H-L (70)	IL/MLCT
	384	8782	403	0.0026	H-1-L(66)	MLCT
	315	28009	307	0.3256	H-4-L(57)	IL/MLCT
					H-L+1(35)	IL/MLCT
**4a**	555	1300	541 (T_1)	0	H-L(64)	IL/MLCT/LLCT
					H-1-L(16)	IL/MLCT/LLCT
	461	56662	484 (S_1)	0.1323	H-L(70)	IL/MLCT/LLCT
	401	6719	414	0.0091	H-1-L(52)	IL/MLCT/LLCT
	279	45330	313	0.1605	H-5-L(52	IL/MLCT
					H-1-L+1(12)	IL/MLCT/LLCT
					H-L+2(51)	IL/MLCT/LLCT
**5a**	515	568	518 (T_1)	0	H-L (64)	IL/MLCT/LLCT
					H-L+1(13)	LLCT/MLCT
	392	16496	452 (S_1)	0.1338	H-L (70)	IL/MLCT/LLCT
	396	23475	387	0.0032	H-2-L(61)	IL/MLCT
					H-1-L(33)	MLCT/LLCT
	322	58647	344	0.4307	H-3-L(11)	IL/MLCT/LLCT
					H-L+1(66))	IL/MLCT/LLCT
	319	60236	313	0.4423	H-5-L(44)	IL/LMCT
					H-L+1(16)	IL/MLCT/LLCT

*Computed TD-DFT data (SMD(CH_2Cl_2)-B3LYP-D3/def2-SVP). H (HOMO); L (LUMO).

Table S4. Selected experimental UV-vis absorption data for complexes **3b**, **4b** and **5b** (10^{-5} M), computed TD-DFT vertical excitation energies (in DCM) and their major contributions.*

Complex	λ_{max}(nm)	ε (M^{-1} cm^{-1})	excitation energy*	oscillator strength *f*	transition (contribution%)	character of the transition
**3b**	521	133	546 (T_1)	0	H-L (45)	IL/MLCT
	454	5644	442 (S_1)	0.0881	H-L (64)	IL/MLCT
	396	22744	403	0.1390	H-1-L(54)	IL/MLCT
	329	38214	330	0.0471	H-4-L+1 (58)	IL/MLCT
					H-2-L (11)	MLCT
	289	65075	289	0.2730	H-5-L (37)	IL/MLCT
					H-5-L+1 (50)	IL/MLCT
**4b**	535	2124	542 (T_1)	0	H-L (29)	IL/MLCT/LLCT
					H-1-L (39)	IL/MLCT/LLCT
	451	12464	472 (S_1)	0.0427	H-L (64)	IL/MLCT/LLCT
	391	48953	398	0.1684	H-1-L (58)	IL/MLCT/LLCT
					H-L+1 (28)	IL/MLCT/LLCT
	360	62197	369	0.1280	H-3-L (43)	IL/MLCT/LLCT
					H-3-L+1 (26)	MLCT
					H-1-L (14)	IL/MLCT/LLCT
	293	120694	295	0.2868	H-L+2(64)	IL/MLCT
						IL/MLCT/LLCT
**5b**	532	809	549 (T_1)	0	H-L (48)	IL/MLCT/LLCT
					H-1-L (36)	IL/MLCT/LLCT
	470	8389	442 (S_1)	0.0883	H-L (68)	IL/MLCT/LLCT
	395	50886	386	0.2277	H-1-L (57)	IL/MLCT/LLCT
	345	64120	373	0.1404	H-3-L (53)	MLCT
					H-1-L (34)	IL/MLCT/LLCT
	290	183238	313	0.4307	H-3-L (11)	MLCT
					H-L+1(66))	IL/MLCT/LLCT
	276	201957	284	0.1154	H-6-L (34)	IL/MLCT
					H-5-L+2 (35)	IL/MLCT/LLCT

*Computed TD-DFT data (SMD(CH_2Cl_2)-B3LYP-D3/def2-SVP). H (HOMO); L (LUMO).

Table S5. Selected experimental UV-vis absorption data for complexes **3c**, **4c** and **5c** (10^{-5} M), computed TD-DFT vertical excitation energies (in DCM) and their major contributions.*

Complex	λ_{max}(nm)	ε($M^{-1}cm^{-1}$)	excitation energy*	oscillator strength *f*	transition (contribution%)	character of the transition
**3c**	495	2457	480(T_1)	0	H-L (43)	IL/MLCT
	454	12269	410 (S_1)	0.1197	H-L (69)	IL/MLCT
					H-L+1 (14)	IL/MLCT
	390	48953	318	0.1552	H-4-L (44)	IL
					H-2-L+1 (23)	MLCT
	290	123218	288	0.1905	H-5-L (67)	IL/MLCT
			297	0.1981	H-4-L+1 (50)	IL
**4c**	470	4314	478 (T_1)	0	H-L (30)	IL/MLCT/LLCT
					H-1-L (27)	IL/MLCT/LLCT
	404	18501	419 (S_1)	0.0677	H-L (68)	IL/MLCT/LLCT
					H-1-L (16)	IL/MLCT/LLCT
	356	22562	322	0.1011	H-4-L (41)	IL
					H-L+2 (30)	IL/MLCT/LLCT
	330	62197	301	0.3044	H-4-L+1 (40)	IL/MLCT
					H-1-L+2 (25)	IL/MLCT/LLCT
					H-4-L (21)	IL
	275	120694	298	0.1996	H-5-L (55)	IL/MLCT
					H-4-L (21)	IL
					H-4-L+1 (26)	IL
**5c**	492	3775	483 (T_1)	0	H-L+1 (46)	IL/MLCT/LLCT
	405	38982	404 (S_1)	0.1140	H-L (66)	IL/MLCT/LLCT
			398	0.1152	H-L+1 (65)	IL/MLCT/LLCT
	358	66546	333	0.4331	H-L+2 (48)	IL/MLCT/LLCT
					H-2-L (24)	MLCT
	321	143933	323	0.1627	H-3-L (46)	IL/MLCT/LLCT
	300	183238	293	0.2671	H-5-L (39)	IL/MLCT/LLCT
					H-L+3 (35)	IL/MLCT/LLCT

*Computed TD-DFT data (SMD(CH_2Cl_2)-B3LYP-D3/def2-SVP). H (HOMO); L (LUMO).

Table S6. Selected experimental UV-vis absorption data for complexes **3d**, **4d** and **5d** (10^{-5} M), computed TD-DFT vertical excitation energies (in DCM) and their major contributions.*

**Complex**	**λ_{max}(nm)**	**ε($M^{-1}cm^{-1}$)**	**excitation energy***	**oscillator strength *f***	**transition contribution(%)**	**character of the transition**
**3d**	474	214	479 (T_1)	0	H-L (62)	IL/MLCT
	395	10689	411 (S_1)	0.0747	H-L (70)	IL/MLCT
	355	11026	340	0.1251	H-2-L (65)	MLCT
					H-4-L (23)	IL/MLC
	318	22933	301	0.1370	H-4-L (60)	IL/MLCT
	305	24147			H-L+2 (17)	IL/MLCT
**4d**	484	592	477 (T_1)	0	H-L (52)	IL/MLCT/LLCT
					H-1-L (31)	IL/MLCT/LLCT
	404	6480	422 (S_1)	0.0826	H-L (69)	IL/MLCT/LLCT
	360	5412	336	0.1193	H-3-L (64)	IL/MLCT
	298	31594	304	0.1610	H-4-L (57)	IL/MLCT/LLCT
					H-5-L (18)	LLCT
					H-L+2 (40)	IL/MLCT/LLCT
**5d**	478	256	476 (T_1)	0	H-L (51)	IL/MLCT/LLCT
					H-L+1 (35)	IL/MLCT
	395	14394	407 (S_1)	0.1533	H-L (70)	IL/MLCT/LLCT
	332	46233	346	0.3370	H-2-L (25)	MLCT
					H-L+1(56)	IL/MLCT/LLCT
	318	53457	330	0.3550	H-L+1(33)	IL/MLCT
					H-3-L(53)	IL/MLCT

*Computed TD-DFT data (SMD(CH_2Cl_2)-B3LYP-D3/def2-SVP). H (HOMO); L (LUMO).

S.5. HOMO and LUMO orbitals of complexes 3, 4 and 5

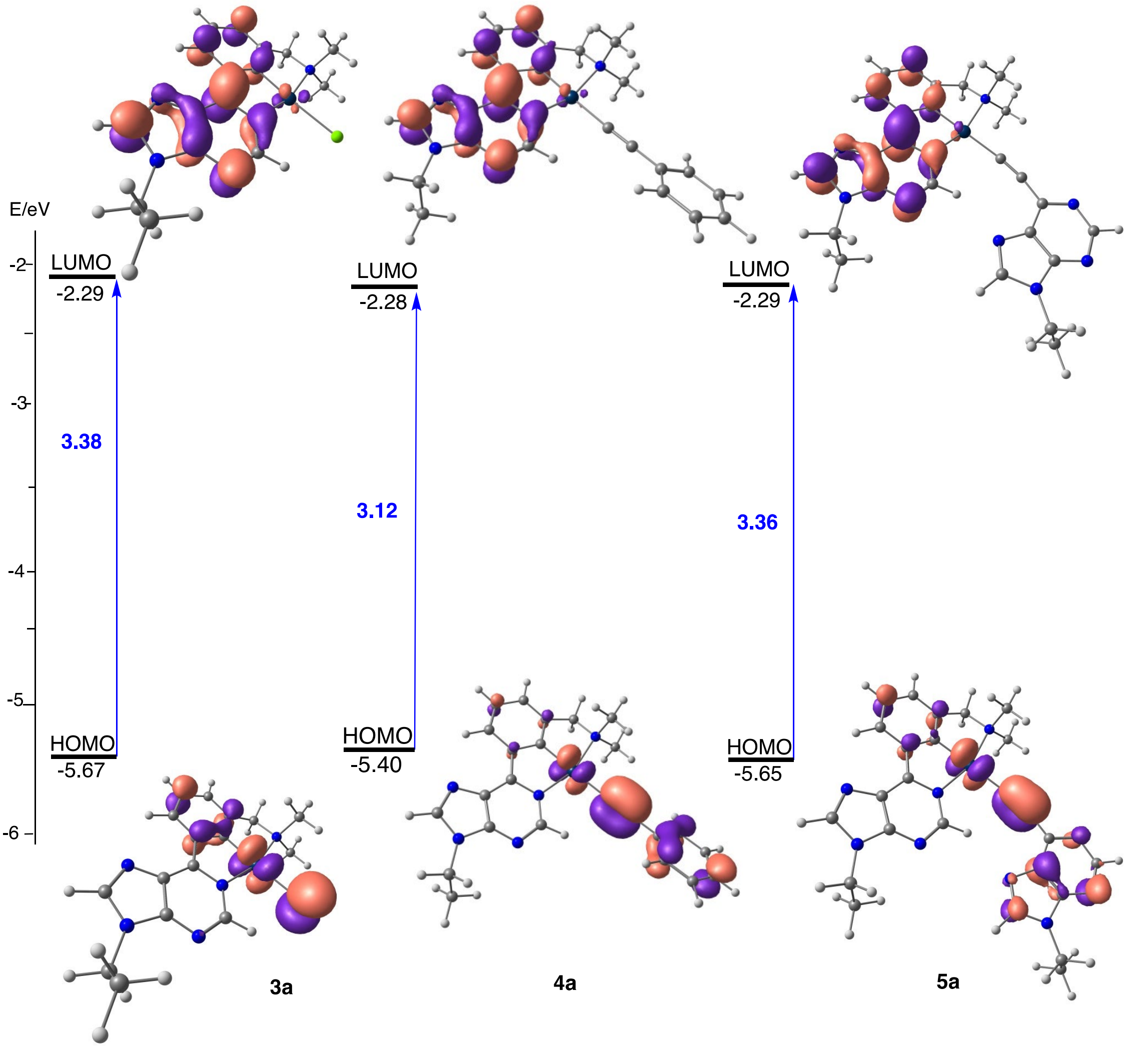

Figure S10. Computed frontier orbitals and energies (eV) of complexes **3a**, **4a** and **5a** (SMD(CH_2Cl_2)-B3LYP-D3/def2-SVP). Isosurface value 0.045.

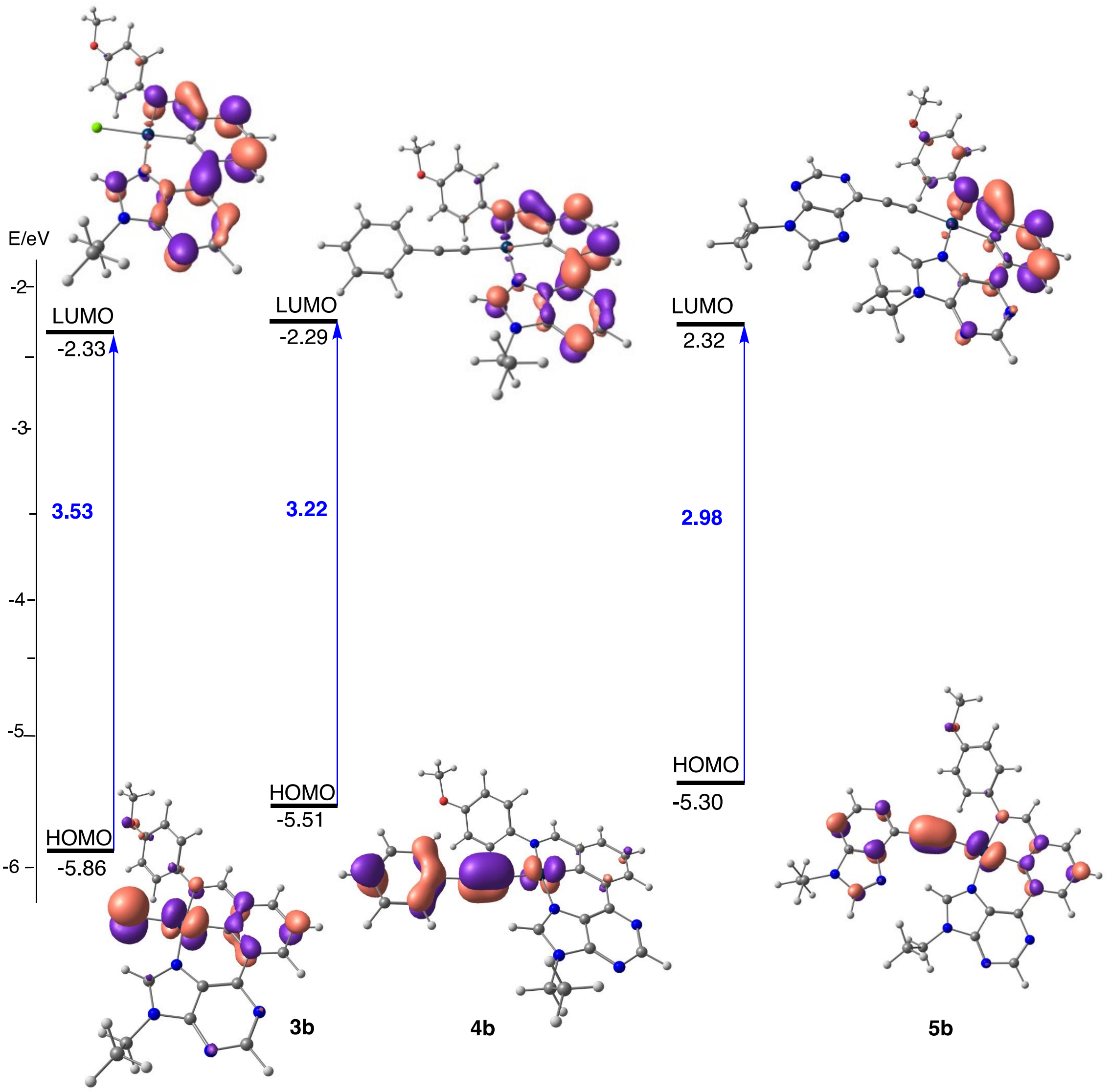

Figure S11. Computed frontier orbitals and energies (eV) of complexes **3b**, **4b** and **5b** (SMD(CH_2Cl_2)-B3LYP-D3/def2-SVP). Isosurface value 0.045.

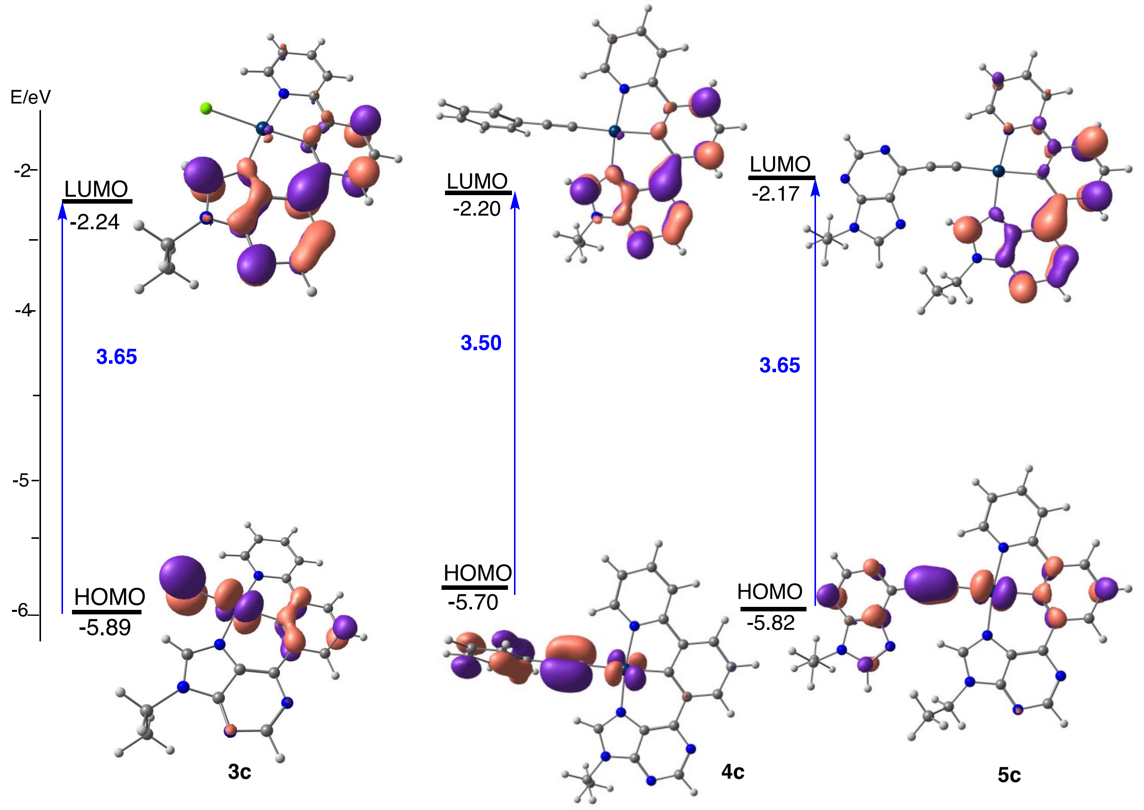

Figure S12 Computed frontier orbitals and energies (eV) of complexes **3c**, **4c** and **5c** (SMD(CH_2Cl_2)-B3LYP-D3/def2-SVP). Isosurface value 0.045.

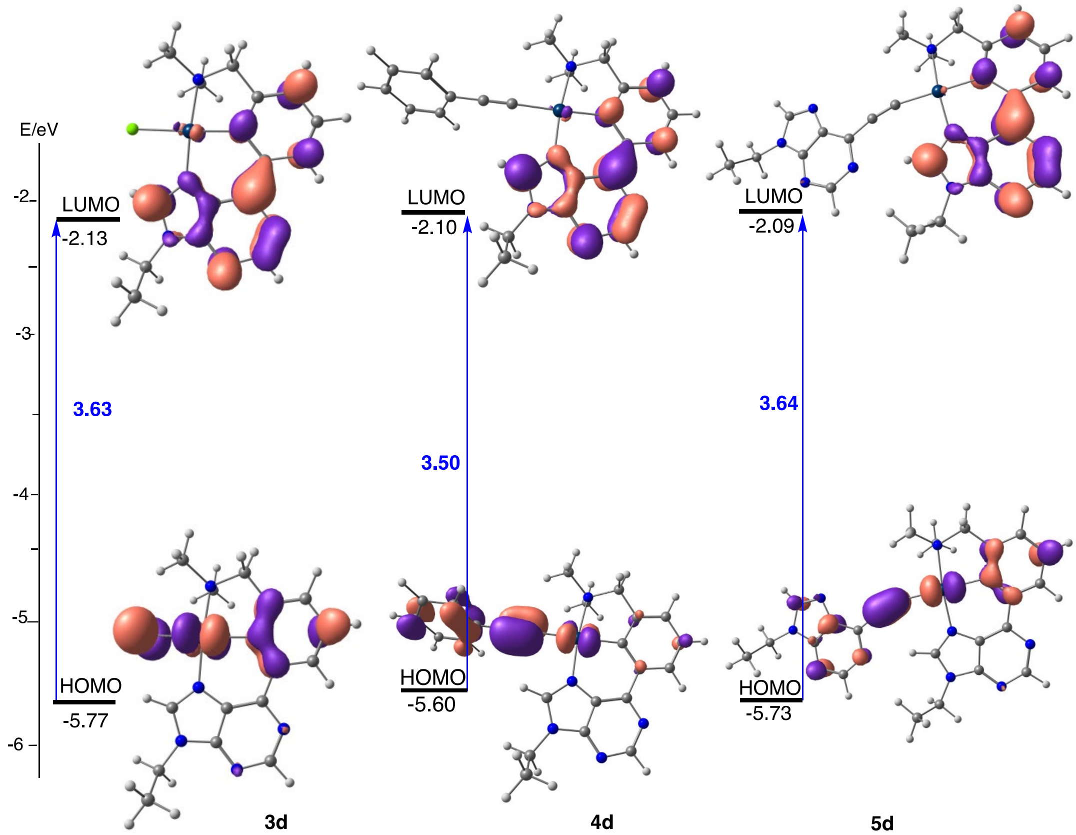

Figure S13. Computed frontier orbitals and energies (eV) of complexes **3d**, **4d** and **5d** (SMD(CH_2Cl_2)-B3LYP-D3/def2-SVP). Isosurface value 0.045.

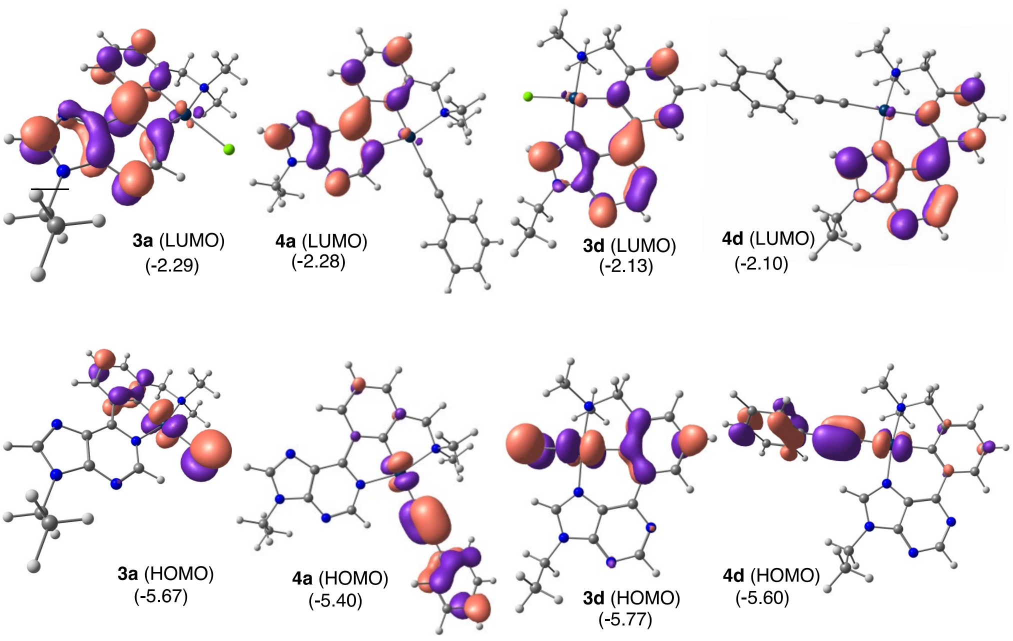

Figure S14. Computed HOMO and LUMO orbitals and energies (eV) of isomers **3a**, and **4a** (left) and **3d** and **4d** (right) (SMD(CH_2Cl_2)-B3LYP-D3/def2-SVP). Isosurface value 0.045.

S.6. Emission studies

Table S7. Emission Data of Complexes 3a,c-d, 4a,c-d, and 5a,c-d [a]

Complex	Medium	*T* (K)	Concentration	λ_{em} (nm)	τ (*μ*s)[b]	*Φ* [c]
**3a**	PMMA	298	5% weight	**530**, 566	0.2 (4.2%), 4.0 (95.8%)	36
	CH_2Cl_2	298	1 x 10^{-3} M	**530**, 560	0.8	5
	CH_2Cl_2	298	1 x 10^{-4} M	**530**, 560	4.8	40
	CH_2Cl_2	298	1 x 10^{-5} M	**530**, 562	10.1	62
	CH_2Cl_2	298	1 x 10^{-6} M	**528**, 560	11.9	-
	CH_2Cl_2	77	1 x 10^{-3} M	540, 570, **700** (exc)	14.5 (62.3%), 5.0 (37.3%)	-
	CH_2Cl_2	77	1 x 10^{-4} M	530, 562, **696** (exc)	13.9 (38.9%), 3.9 (61.1%)	-
	CH_2Cl_2	77	1 x 10^{-5} M	**520**, 560, 696 (exc)	12.7 (45.5%), 5.1 (54.5%)	-
	CH_2Cl_2	77	1 x 10^{-6} M	**516**, 556, 684 (exc)	11.6 (61.0%), 5.3 (39.0%)	-
**3c**	PMMA	298	5% weight	487, **524**, 550 (sh)	3.3 (14.1%), 9.7 (85.9%)	28
	CH_2Cl_2	298	1 x 10^{-5} M	**484**, 516, 548	5.0	20
	CH_2Cl_2	77	1 x 10^{-5} M	486, **526**, 556, 644 (exc)	15.4 (83.4%), 36.0 (16.6%)	-
**3d**	PMMA	298	5% weight	**488**, 521, 558	10.5	70
	CH_2Cl_2	298	1 x 10^{-5} M	**487**, 521, 559	11.9	16
	CH_2Cl_2	77	1 x 10^{-5} M	**486**, 524, 559	34.7 (53.5%), 17.4 (46.5%)	-
**4a**	PMMA	298	2% weight	**529**, 559	0.5 (1.7%), 5.1 (98.3%)	22
	CH_2Cl_2	298	1 x 10^{-3} M	**536**, 560	1.1 (67%), 0.8 (33%)	3
	CH_2Cl_2	298	1 x 10^{-4} M	**531**, 560	5.5 (94.3%), 0.8 (5.7%)	12
	CH_2Cl_2	298	1 x 10^{-5} M	**530**, 560	10.2	23
	CH_2Cl_2	298	1 x 10^{-6} M	**529**, 564	9.8	-
	CH_2Cl_2	77	1 x 10^{-3} M	573, **687** (exc)	35.9 (13.9%), 12.3 (86.1%)	-
	CH_2Cl_2	77	1 x 10^{-4} M	538, 571, **678** (exc)	35.0 (13.1%), 10.4 (86.9%)	-
	CH_2Cl_2	77	1 x 10^{-5} M	**521**, 559, 688 (exc)	46.0 (12.8%), 15.1 (87.2%)	-
	CH_2Cl_2	77	1 x 10^{-6} M	**518**, 558	11.7 (60.9%), 5.5 (39.1%)	-
**4c**	PMMA	298	2% weight	**490**, 524, 563 (sh), 622 (exc)	4.2 (26.0%), 9.4 (74.0%)	26
	PMMA	298	5% weight	491, 527, **622** (exc)	1.4 (56.7%), 6.0 (43.3%)	15
	CH_2Cl_2	298	1 x 10^{-5} M	**488**, 522, 556	5.4	21
	CH_2Cl_2	77	1 x 10^{-5} M	**490**, 526, 568	21.0 (82.3%), 11.5 (17.7%)	-
**4d**	PMMA	298	5% weight	**492**, 521, 563 (sh)	7.7	61
	CH_2Cl_2	298	1 x 10^{-3} M	**492**, 522, 556 (sh)	1.8	6

	CH_2Cl_2	298	1 x 10^{-4} M	**492**, 522, 560 (sh)	2.1	7
	CH_2Cl_2	298	1 x 10^{-5} M	493, **524,** 562 (sh)	11.0 (17.6%), 2.2 (82.4%)	7
	CH_2Cl_2	298	1 x 10^{-6} M	**488**, 522**,** 556 (sh)	13.3 (59.5%), 5.2 (40.5%)	7
	CH_2Cl_2	77	1 x 10^{-5} M	489, **520**, 559 (sh)	21.3 (29.8%), 6.9 (70.2%)	-
	CH_2Cl_2	77	1 x 10^{-6} M	486, **518**, 556	62 (26%), 17.5 (74%)	-
**5a**	PMMA	298	5% weight	**484**, 517, 556 (sh)	6.0	50
	CH_2Cl_2	298	1 x 10^{-5} M	**482**, 515, 554 (sh)	2.4	12
	CH_2Cl_2	77	1 x 10^{-5} M	**482**, 516, 555 (sh)	23.5 (44.1%), 10.4 (55.9%)	-
**5c**	PMMA	298	2% weight	**487**, 524, 568, 609 (exc)	4.4 (16.1%), 12.1 (83.9%)	50
	PMMA	298	5% weight	489, 526, **609** (exc)	1.4 (54.5%), 7.6 (45.5%)	35
	CH_2Cl_2	298	1 x 10^{-3} M	488, 526, **626** (exc)	0.5	13
	CH_2Cl_2	298	1 x 10^{-4} M	**484**, 520, 562, 606 (exc)	3.1	21
	CH_2Cl_2	298	1 x 10^{-5} M	**484**, 520, 558	7.1	23
	CH_2Cl_2	298	1 x 10^{-6} M	**486**, 520, 556	7.5	22
	CH_2Cl_2	77	1 x 10^{-3} M	490, 526, **614** (exc)	21.6 (28.7%), 11.2 (71.3%)	-
	CH_2Cl_2	77	1 x 10^{-4} M	**488**, 526, 606 (exc)	20.5 (40.5%), 11.7 (59.5%)	-
	CH_2Cl_2	77	1 x 10^{-5} M	**488**, 526, 608 (exc)	27.6 (26.9%), 12.4 (73.1%)	-
	CH_2Cl_2	77	1 x 10^{-6} M	488, **522**, 558, 602 (sh, exc)	78.3 (41.2%), 23.6 (58.8%)	-
**5d**	PMMA	298	5% weight	**484**, 516, 553	7.8	48
	CH_2Cl_2	298	1 x 10^{-5} M	**484**, 517, 555	11.7 (47.9%), 3.3 (52.1%)	5
	CH_2Cl_2	77	1 x 10^{-5} M	**482**, 517, 555 (sh)	32.0 (46.9%), 15.0 (53.1%)	-

(a) The most intense peak is in bold. (b) Relative amplitudes (%) are given in parentheses for biexponential decays. (c) Absolute quantum yield.

Table S8.
Computed energies of S_1 and T_1 states (kcal mol^{-1})[S2-S4]

Complex	S_1[a]	T1[b]
**3a**	62.95	53.02
**3c**	70.56	60.09
**3d**	69.41	58.55
**4a**	59.03	52.43
**4c**	69.64	58.74
**4d**	67.56	58.28
**5a**	63.18	54.52
**5c**	68.03	59.91
**5d**	70.10	63.90

a) Estimated by TD-DFT-B3LYP-D3/def2-SVP calculations, in CH_2Cl_2 from the S_0-S_1 excitation energies. b) T_1 energies obtained by the computed S_0–T_1 gaps (B3LYP-D3/def2-SVP in CH_2Cl_2). Calculated from the differences in energy between the optimized triplet states T_1 and the singlet states S_0. See ref S2, S3.

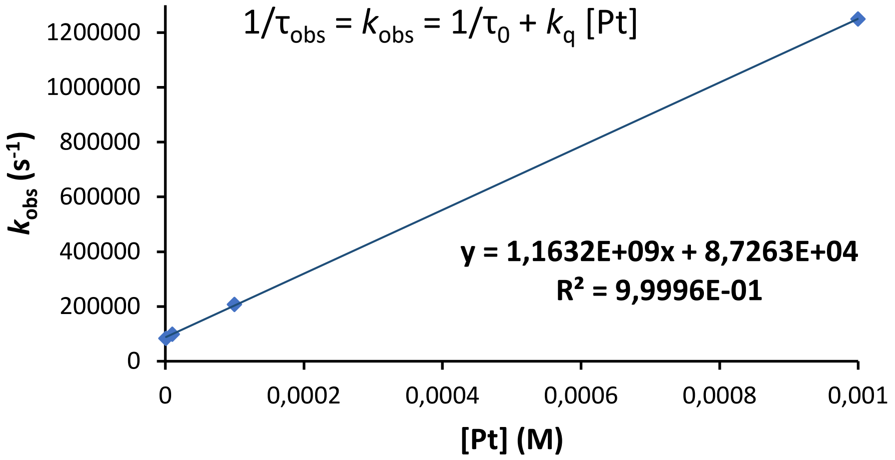

Figure S15. Stern-Volmer plot for **3a** (CH_2Cl_2 solution at 298 K). The slope is k_q and the y intercept is $1/\tau_0$.

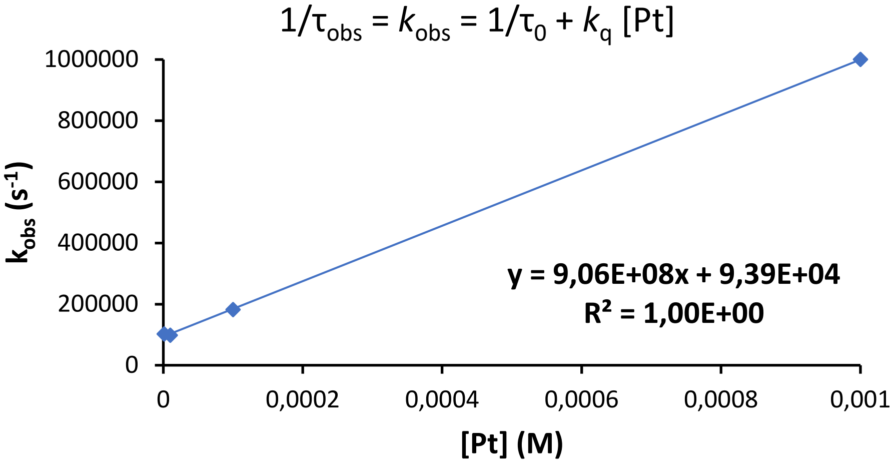

Figure S16. Stern-Volmer plot for **4a** (CH_2Cl_2 solution at 298 K). The slope is k_q and the y intercept is $1/\tau_0$.

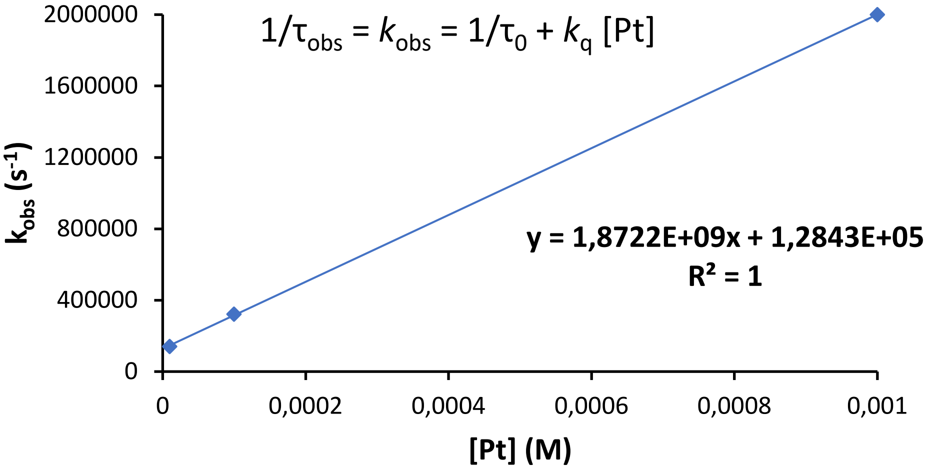

Figure S17. Stern-Volmer plot for **5c** (CH_2Cl_2 solution at 298 K). The slope is k_q and the y intercept is $1/\tau_0$.

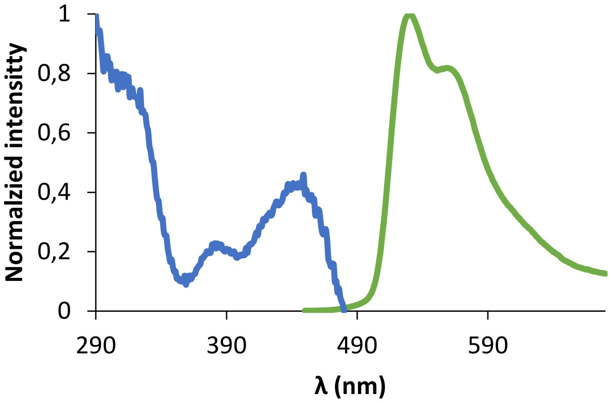

Figure S18. Normalized excitation (blue line) and emission (green line) spectra of **3a** in PMMA film (5 wt%) at 298 K.

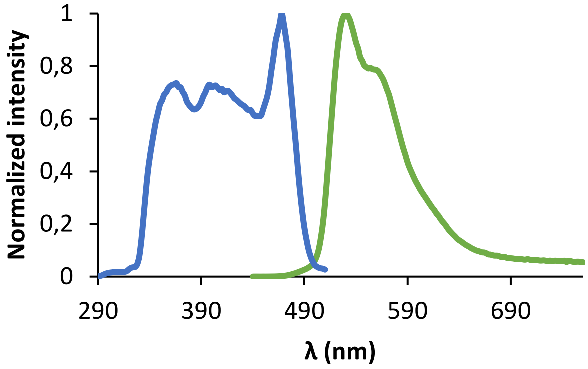

Figure S19. Normalized excitation (blue line) and emission (green line) spectra of **3a** in a 1.0 x 10^{-3} M solution in dichloromethane at 298 K.

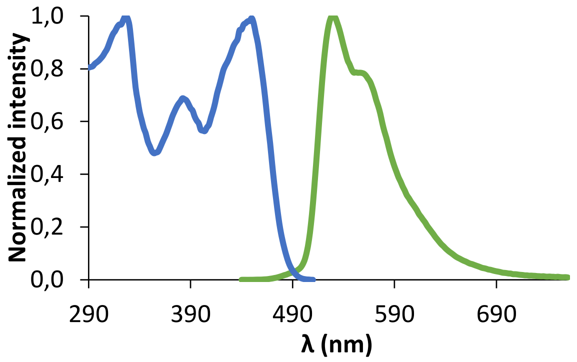

Figure S20. Normalized excitation (blue line) and emission (green line) spectra of **3a** in a 1.0 x 10^{-4} M solution in dichloromethane at 298 K.

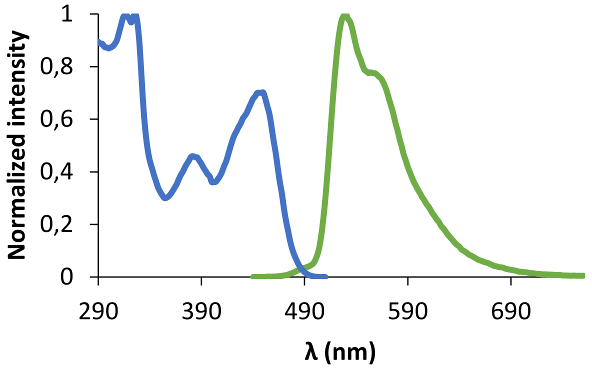

Figure S21. Normalized excitation (blue line) and emission (green line) spectra of **3a** in a 1.0 x 10^{-5} M solution in dichloromethane at 298 K.

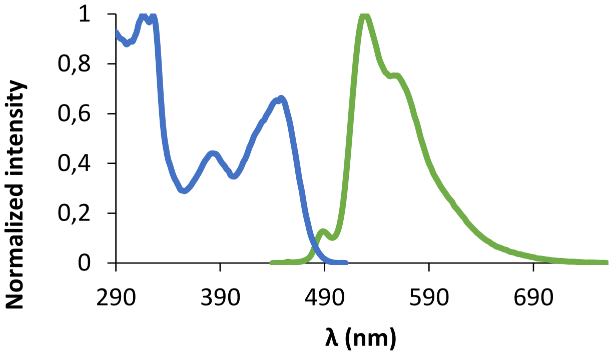

Figure S22. Normalized excitation (blue line) and emission (green line) spectra of **3a** in a 1.0 x 10^{-6} M solution in dichloromethane at 298 K.

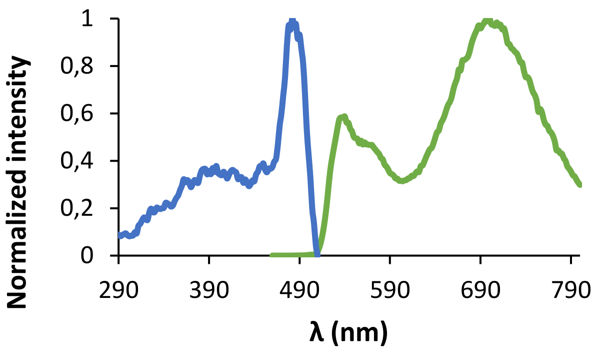

Figure S23. Normalized excitation (blue line) and emission (green line) spectra of **3a** in a 1.0 x 10^{-3} M solution in dichloromethane at 77 K.

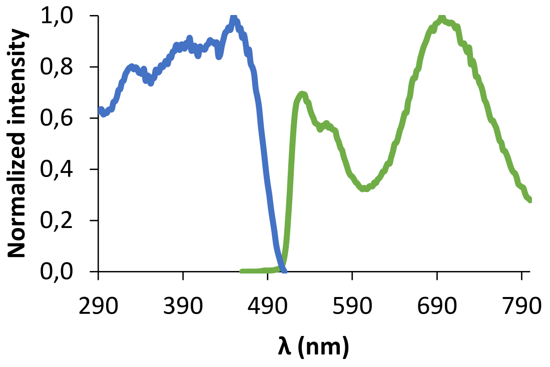

Figure S24. Normalized excitation (blue line) and emission (green line) spectra of **3a** in a 1.0 x 10^{-4} M solution in dichloromethane at 77 K.

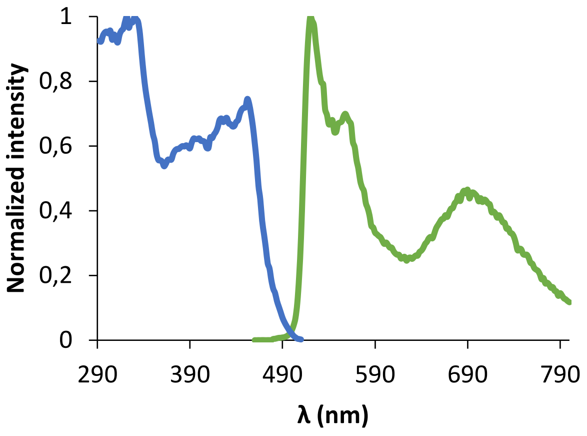

Figure S25. Normalized excitation (blue line) and emission (green line) spectra of **3a** in a 1.0 x 10^{-5} M solution in dichloromethane at 77 K.

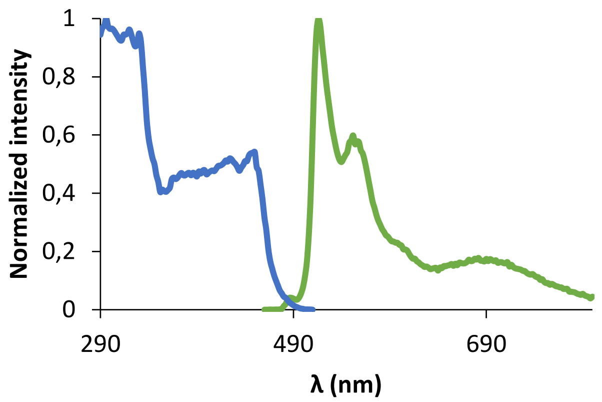

Figure S26. Normalized excitation (blue line) and emission (green line) spectra of **3a** in a 1.0 x 10^{-6} M solution in dichloromethane at 77 K.

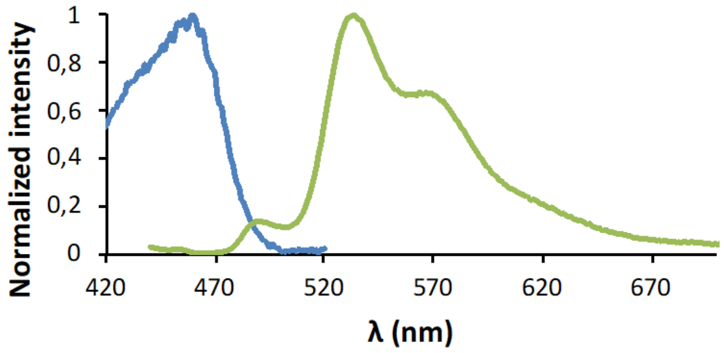

Figure S27. Normalized excitation (blue line) and emission (green line) spectra of **3a** in a 1.0 x 10^{-5} M solution in 2-MeTHF at 298 K

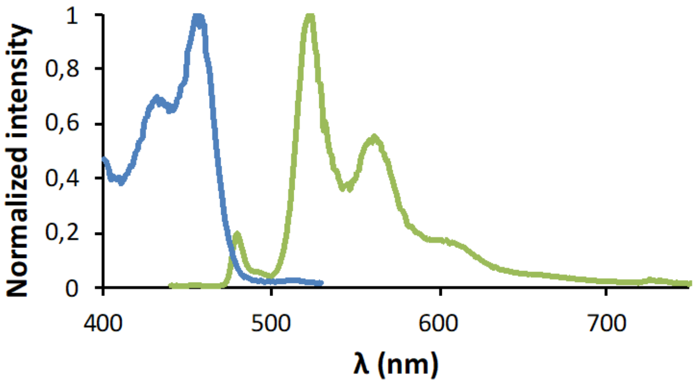

Figure S28. Normalized excitation (blue line) and emission (green line) spectra of **3a** in a 1.0 x 10^{-5} M solution in 2-MeTHF at 77 K

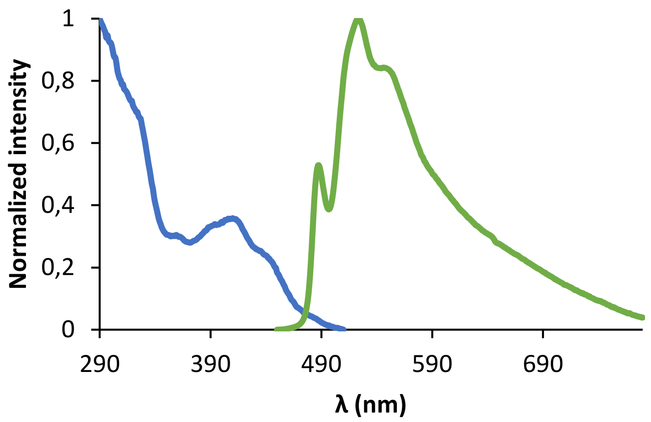

Figure S29. Normalized excitation (blue line) and emission (green line) spectra of **3c** in PMMA film (5 wt%) at 298 K.

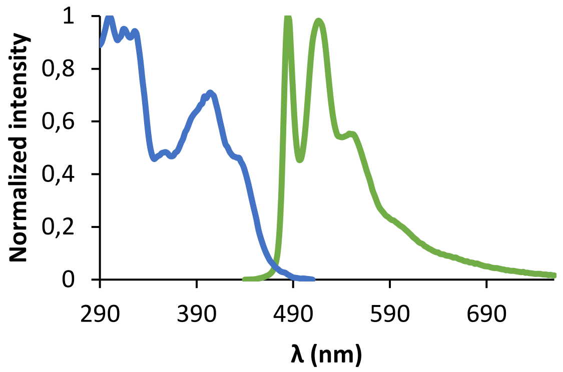

Figure S30. Normalized excitation (blue line) and emission (green line) spectra of **3c** in a 1.0 x 10^{-5} M solution in dichloromethane at 298 K.

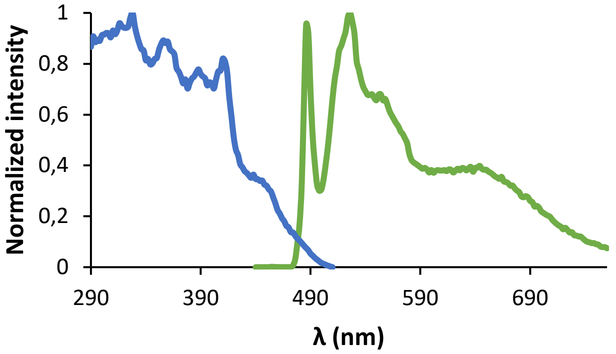

Figure S31. Normalized excitation (blue line) and emission (green line) spectra of **3c** in a 1.0 x 10^{-5} M solution in dichloromethane at 77 K.

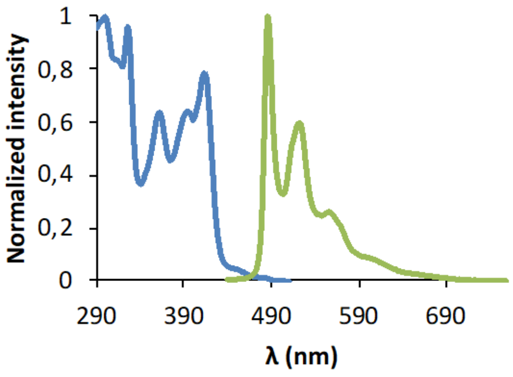

Figure S32. Normalized excitation (blue line) and emission (green line) spectra of **3c** in a 1.0 x 10^{-5} M solution in 2-MeTHF at 298 K.

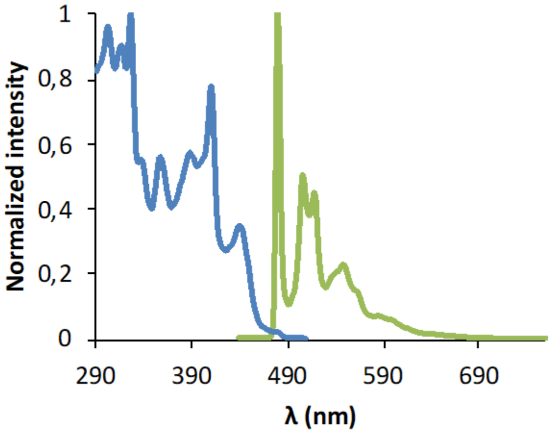

Figure S33.. Normalized excitation (blue line) and emission (green line) spectra of **3c** in a 1.0 x 10^{-5} M solution in 2-MeTHF at 77 K.

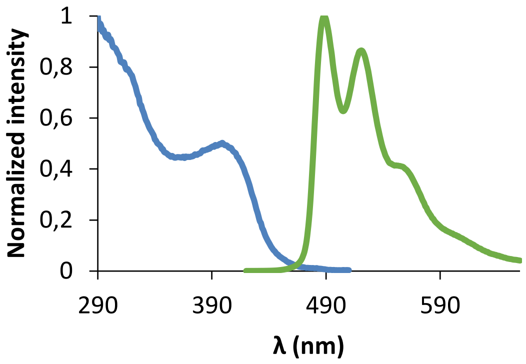

Figure S34. Normalized excitation (blue line) and emission (green line) spectra of **3d** in PMMA film (5 wt%) at 298 K.

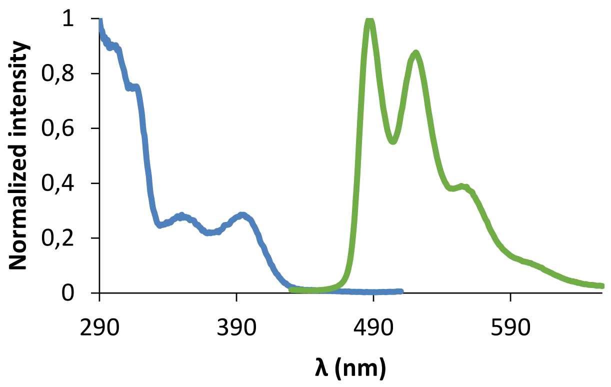

Figure S35. Normalized excitation (blue line) and emission (green line) spectra of **3d** in a 1.0 x 10^{-5} M solution in dichloromethane at 298 K.

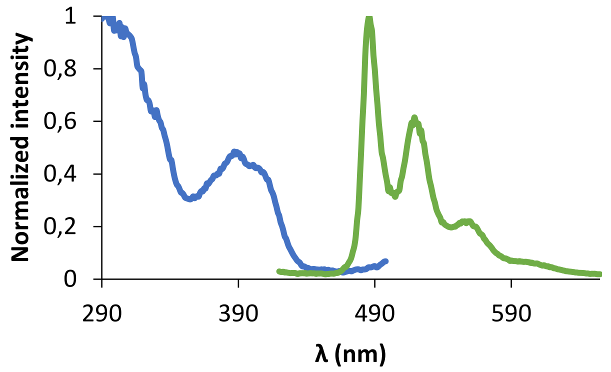

Figure S36. Normalized excitation (blue line) and emission (green line) spectra of **3d** in a 1.0 x 10^{-5} M solution in dichloromethane at 77 K.

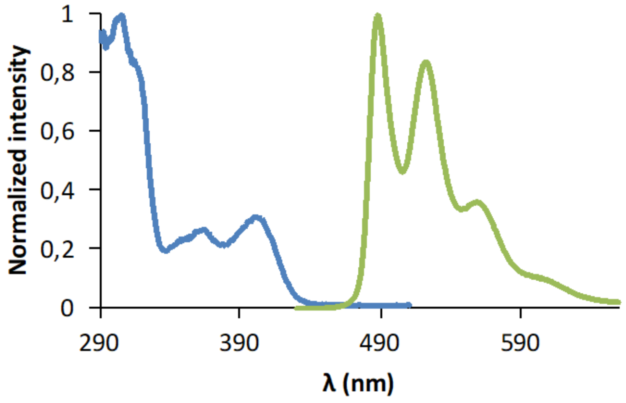

Figure S37. Normalized excitation (blue line) and emission (green line) spectra of **3d** in a 1.0 x 10^{-5} M solution in 2-MeTHF at 298 K.

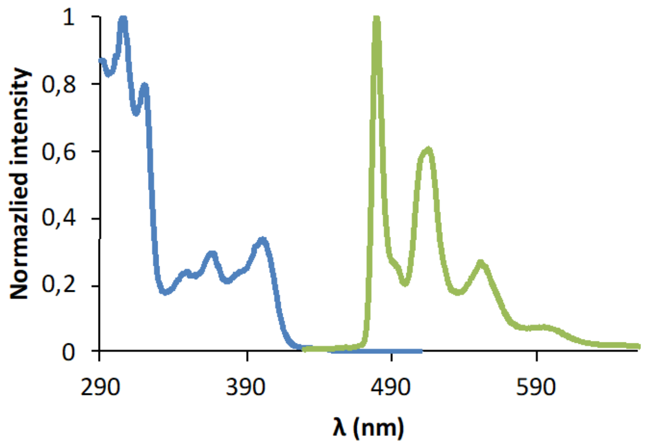

Figure S38. Normalized excitation (blue line) and emission (green line) spectra of **3d** in a 1.0 x 10^{-5} M solution in 2-MeTHF at 77 K.

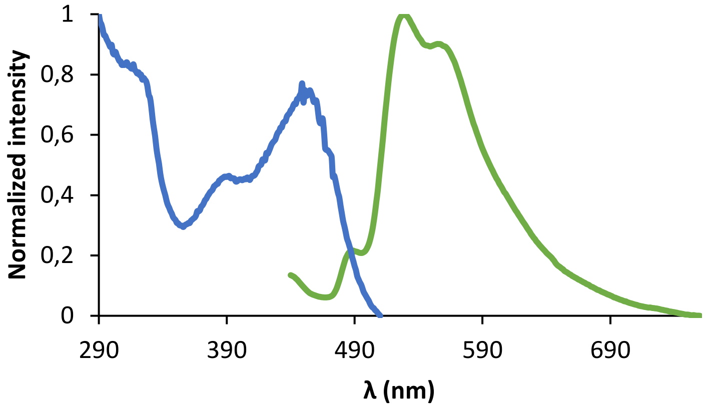

Figure S39. Normalized excitation (blue line) and emission (green line) spectra of **4a** in PMMA film (2 wt%) at 298 K.

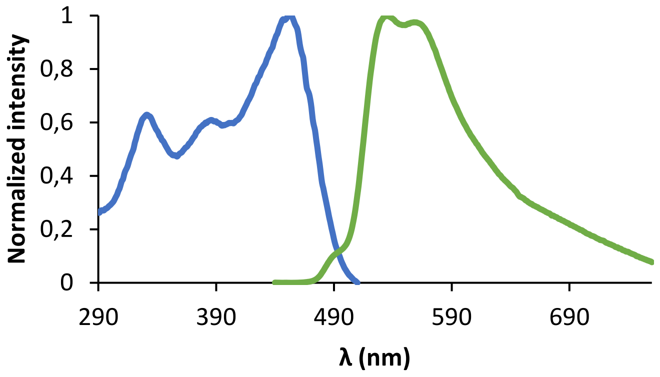

Figure S40. Normalized excitation (blue line) and emission (green line) spectra of **4a** in a 1.0 x 10^{-3} M solution in dichloromethane at 298 K.

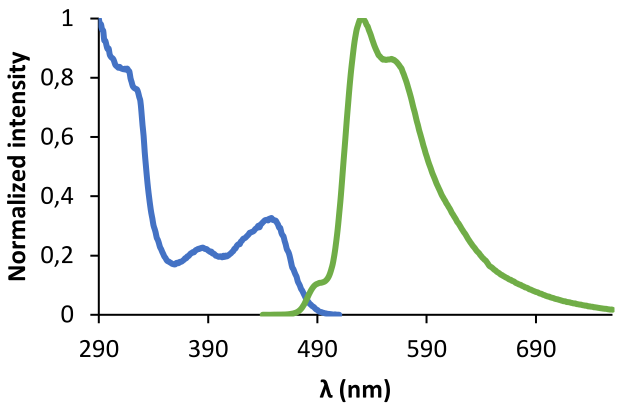

Figure S41. Normalized excitation (blue line) and emission (green line) spectra of **4a** in a 1.0 x 10^{-4} M solution in dichloromethane at 298 K.

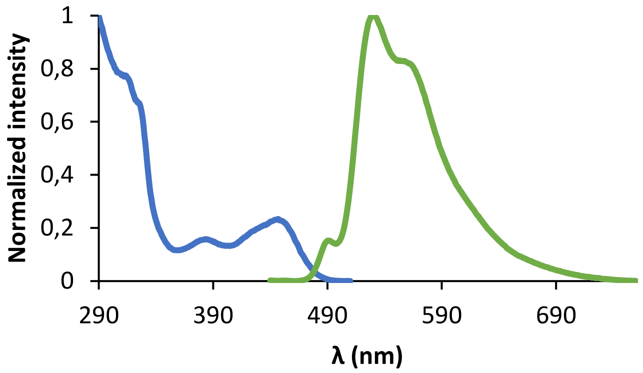

Figure S42. Normalized excitation (blue line) and emission (green line) spectra of **4a** in a 1.0 x 10^{-5} M solution in dichloromethane at 298 K.

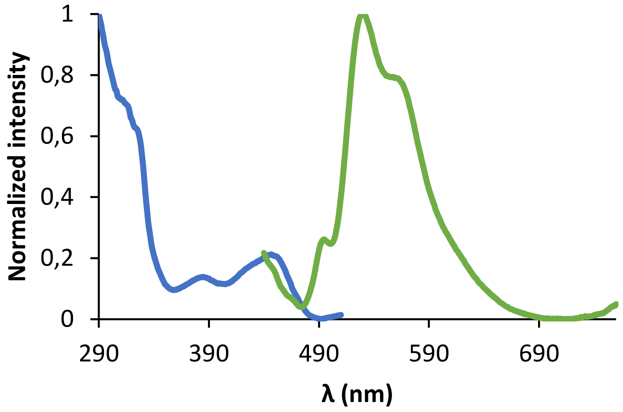

Figure S43. Normalized excitation (blue line) and emission (green line) spectra of **4a** in a 1.0 x 10^{-6} M solution in dichloromethane at 298 K.

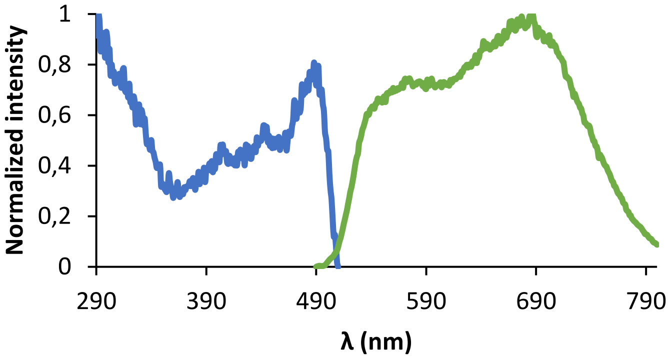

Figure S44. Normalized excitation (blue line) and emission (green line) spectra of **4a** in a 1.0 x 10^{-3} M solution in dichloromethane at 77 K.

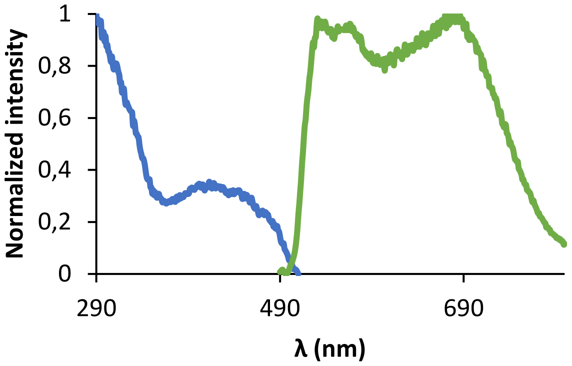

Figure S45. Normalized excitation (blue line) and emission (green line) spectra of **4a** in a 1.0 x 10^{-4} M solution in dichloromethane at 77 K.

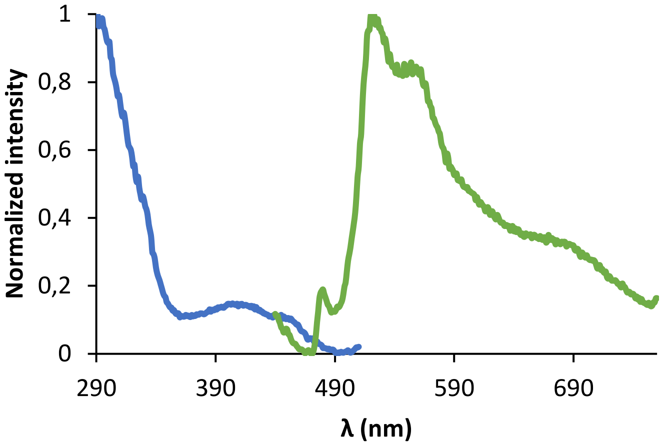

Figure S46. Normalized excitation (blue line) and emission (green line) spectra of **4a** in a 1.0 x 10^{-5} M solution in dichloromethane at 77 K.

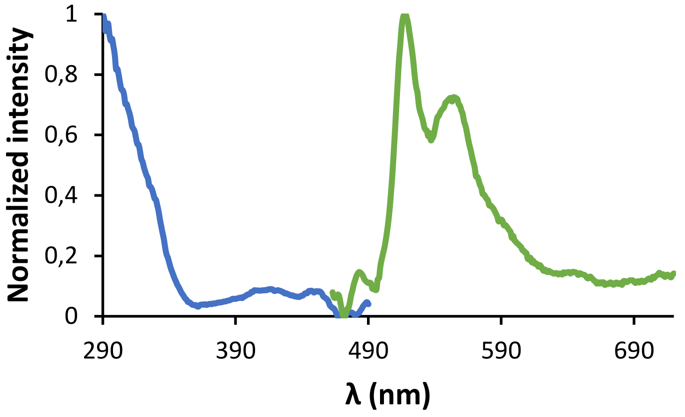

Figure S47. Normalized excitation (blue line) and emission (green line) spectra of **4a** in a 1.0 x 10^{-6} M solution in dichloromethane at 77 K.

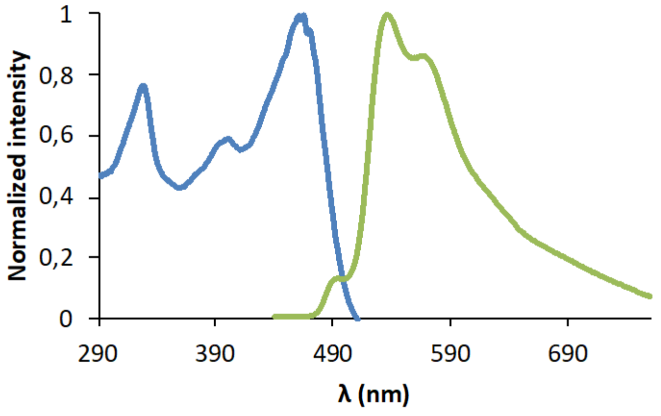

Figure S48. Normalized excitation (blue line) and emission (green line) spectra of **4a** in a 1.0 x 10^{-3} M solution in 2-MeTHF at 298 K.

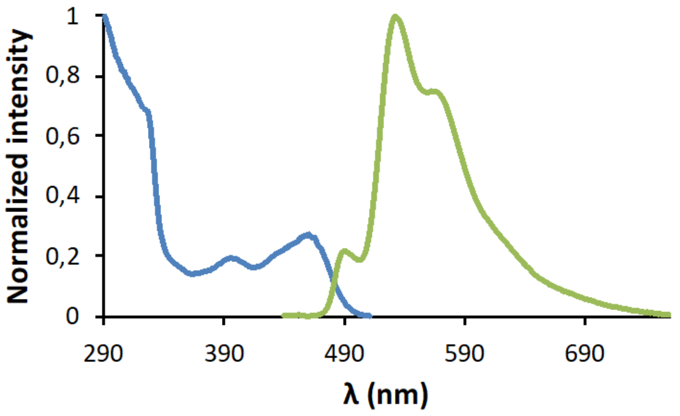

Figure S49. Normalized excitation (blue line) and emission (green line) spectra of **4a** in a 1.0 x 10^{-4} M solution in 2-MeTHF at 298 K.

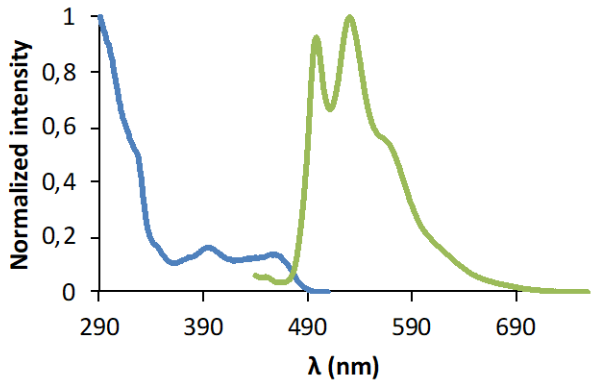

Figure S50. Normalized excitation (blue line) and emission (green line) spectra of **4a** in a 1.0 x 10^{-5} M solution in 2-MeTHF at 298 K.

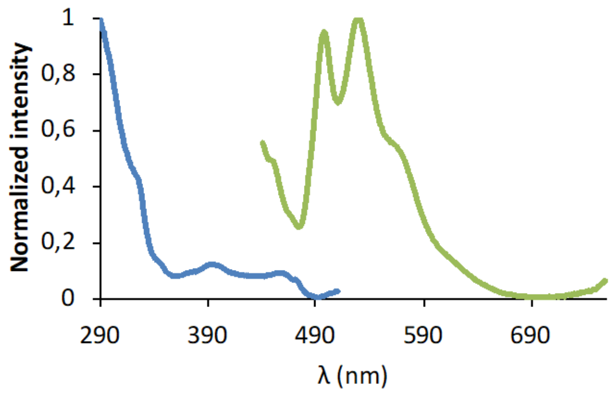

Figure S51. Normalized excitation (blue line) and emission (green line) spectra of **4a** in a 1.0 x 10^{-6} M solution in 2-MeTHF at 298 K.

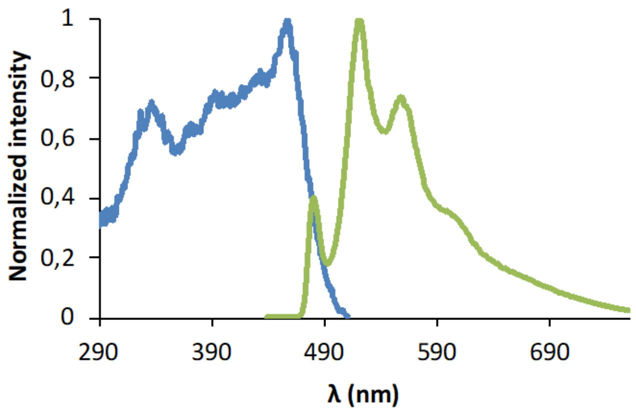

Figure S52. Normalized excitation (blue line) and emission (green line) spectra of **4a** in a 1.0 x 10^{-3} M solution in 2-MeTHF at 77 K.

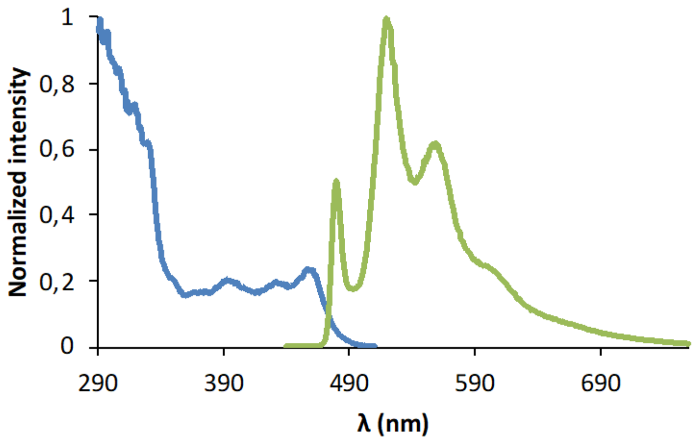

Figure S53. Normalized excitation (blue line) and emission (green line) spectra of **4a** in a 1.0 x 10^{-4} M solution in 2-MeTHF at 77 K.

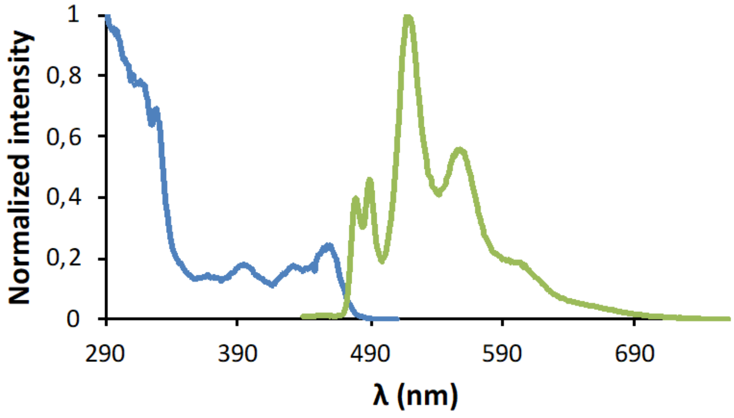

Figure S54. Normalized excitation (blue line) and emission (green line) spectra of **4a** in a 1.0 x 10^{-5} M solution in 2-MeTHF at 77 K.

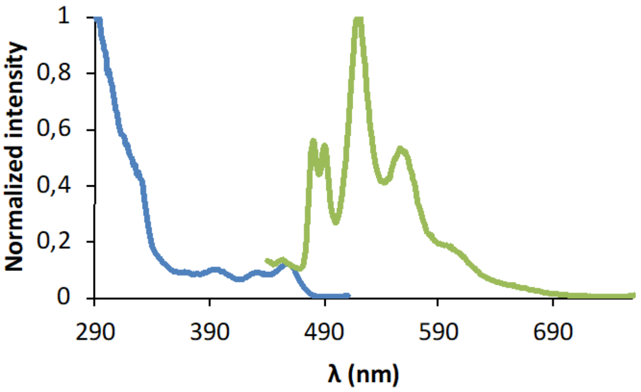

Figure S55. Normalized excitation (blue line) and emission (green line) spectra of **4a** in a 1.0 x 10^{-6} M solution in 2-MeTHF at 77 K.

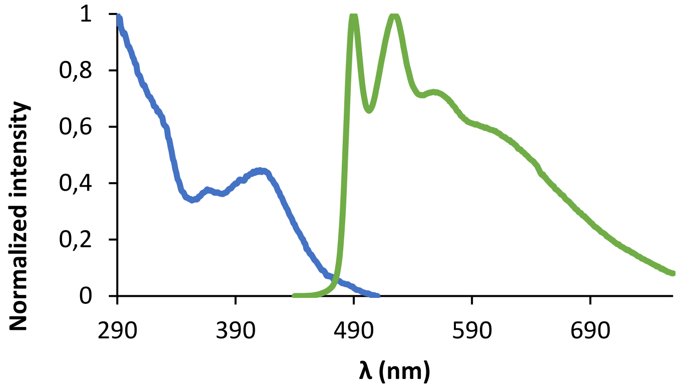

Figure S56. Normalized excitation (blue line) and emission (green line) spectra of **4c** in PMMA film (2 wt%) at 298 K.

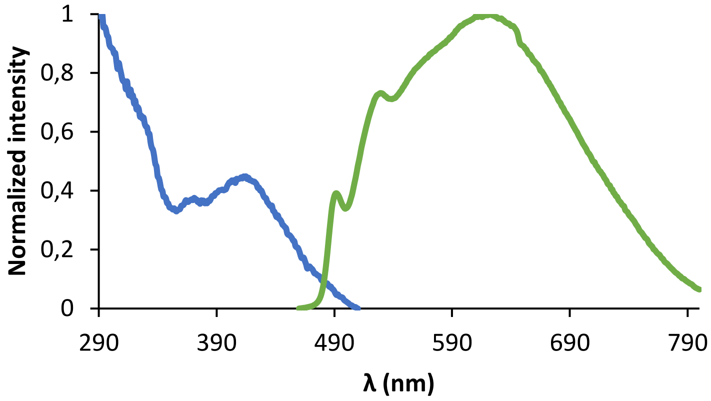

Figure S57. Normalized excitation (blue line) and emission (green line) spectra of **4c** in PMMA film (5 wt%) at 298 K.

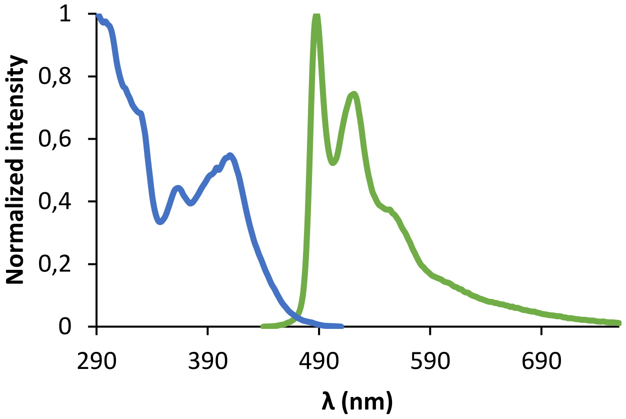

Figure S58. Normalized excitation (blue line) and emission (green line) spectra of **4c** in a 1.0 x 10^{-5} M solution in dichloromethane at 298 K.

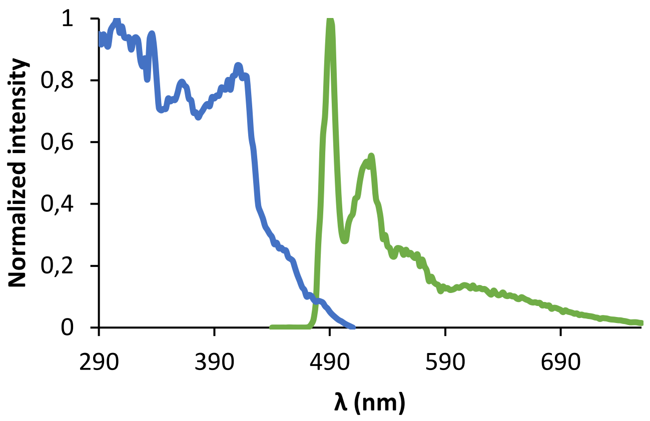

Figure S59. Normalized excitation (blue line) and emission (green line) spectra of **4c** in a 1.0 x 10^{-5} M solution in dichloromethane at 77 K.

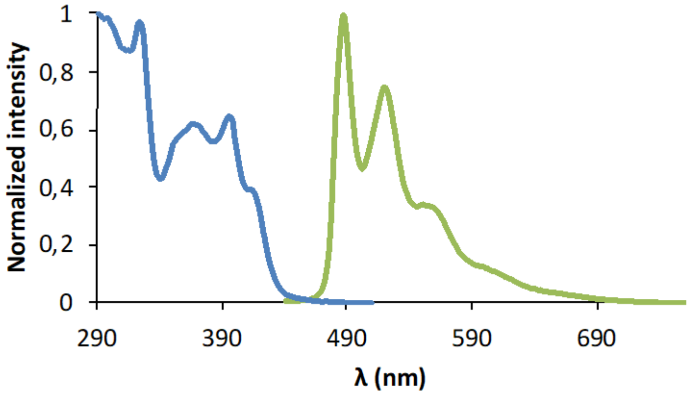

Figure S60. Normalized excitation (blue line) and emission (green line) spectra of **4c** in a 1.0 x 10^{-5} M solution in 2-MeTHF at 298 K.

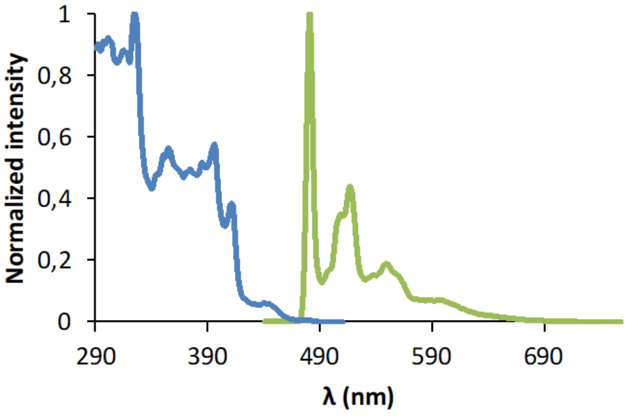

Figure S61. Normalized excitation (blue line) and emission (green line) spectra of **4c** in a 1.0 x 10^{-5} M solution in 2-MeTHF at 77 K.

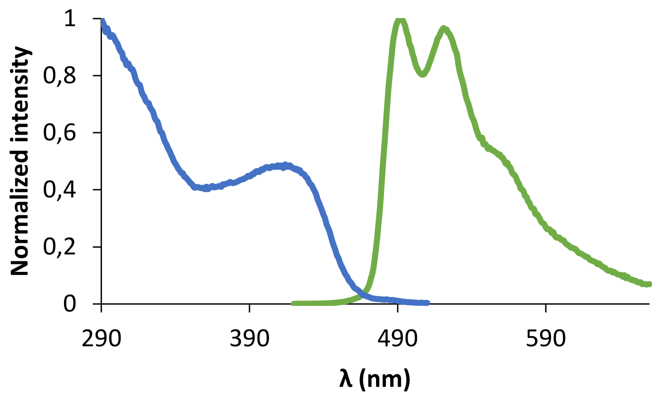

Figure S62. Normalized excitation (blue line) and emission (green line) spectra of **4d** in PMMA film (5 wt%) at 298 K.

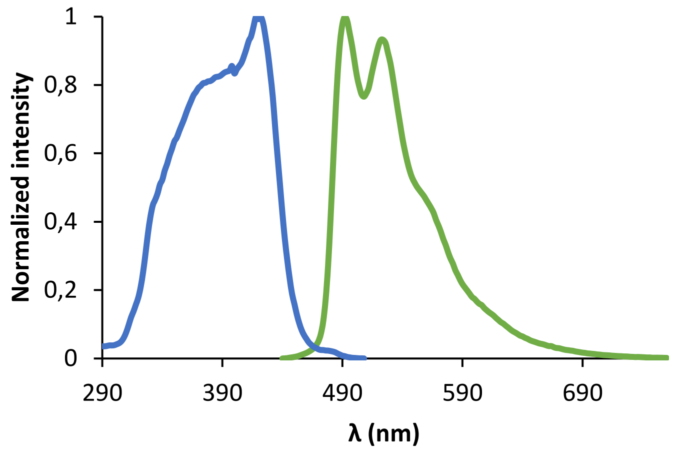

Figure S63. Normalized excitation (blue line) and emission (green line) spectra of **4d** in a 1.0 x 10^{-3} M solution in dichloromethane at 298 K.

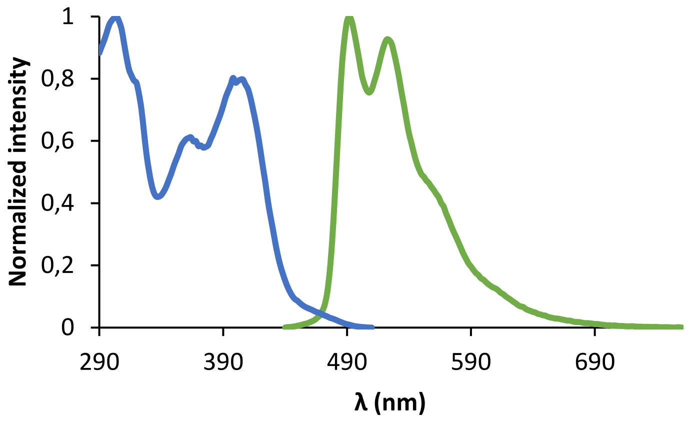

Figure S64. Normalized excitation (blue line) and emission (green line) spectra of **4d** in a 1.0 x 10^{-4} M solution in dichloromethane at 298 K.

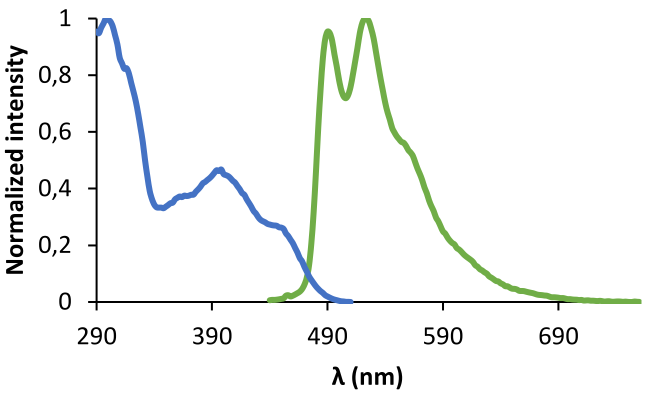

Figure S65. Normalized excitation (blue line) and emission (green line) spectra of **4d** in a 1.0 x 10^{-5} M solution in dichloromethane at 298 K.

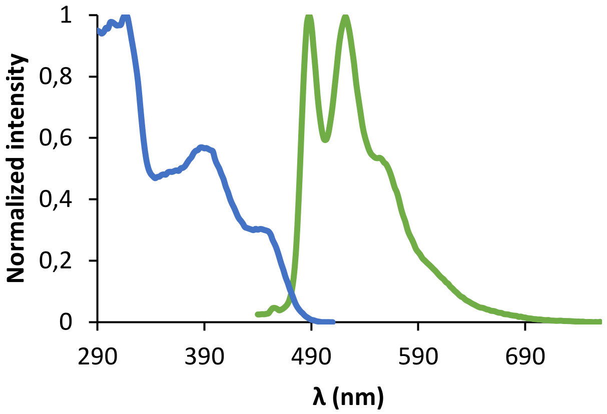

Figure S66. Normalized excitation (blue line) and emission (green line) spectra of **4d** in a 1.0 x 10^{-6} M solution in dichloromethane at 298 K.

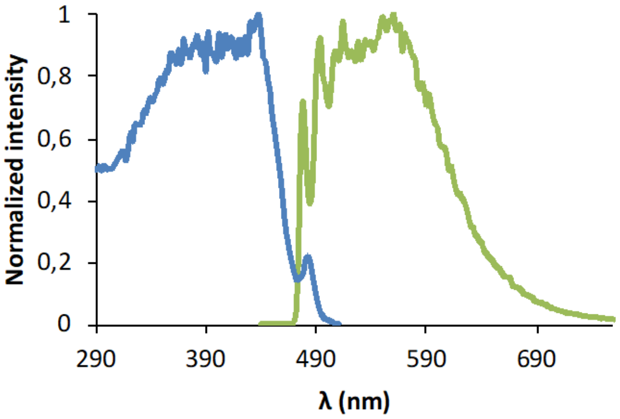

Figure S67. Normalized excitation (blue line) and emission (green line) spectra of **4d** in a 1.0 x 10^{-3} M solution in dichroromethane at 77 K.

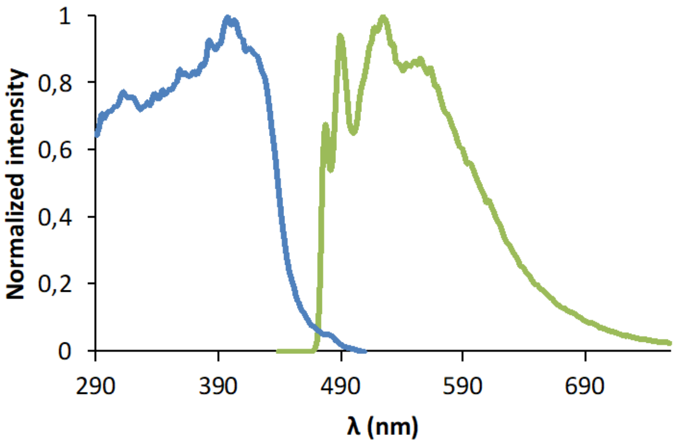

Figure S68. Normalized excitation (blue line) and emission (green line) spectra of **4d** in a 1.0 x 10^{-4} M solution in dichrorormethane at 77 K.

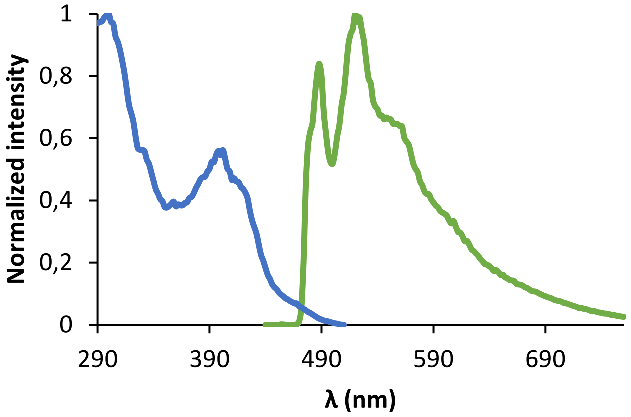

Figure S69. Normalized excitation (blue line) and emission (green line) spectra of **4d** in a 1.0 x 10^{-5} M solution in dichloromethane at 77 K.

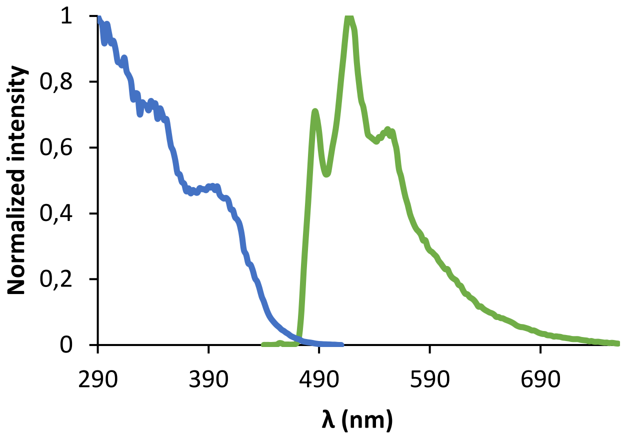

Figure S70. Normalized excitation (blue line) and emission (green line) spectra of **4d** in a 1.0 x 10^{-6} M solution in dichloromethane at 77 K.

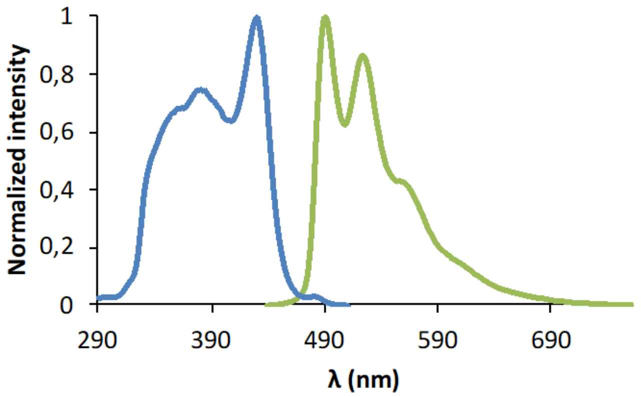

Figure S71. Normalized excitation (blue line) and emission (green line) spectra of **4d** in a 1.0 x 10^{-3} M solution in 2-MeTHF at 298 K.

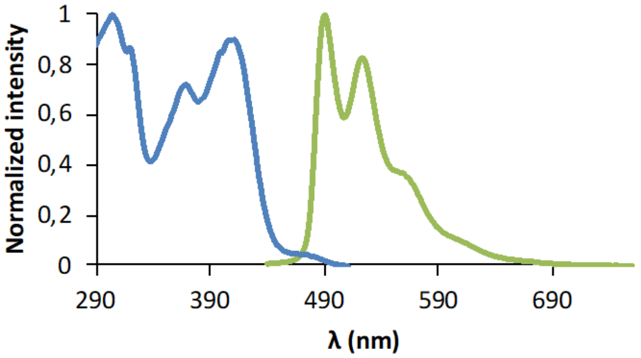

Figure S72. Normalized excitation (blue line) and emission (green line) spectra of **4d** in a 1.0 x 10^{-4} M solution in 2-MeTHF at 298 K.

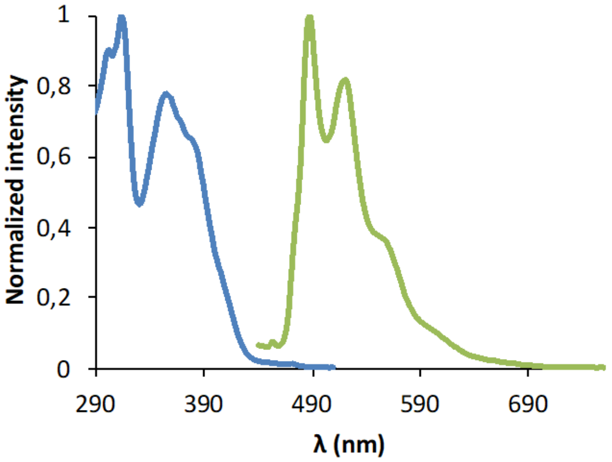

Figure S73. Normalized excitation (blue line) and emission (green line) spectra of **4d** in a 1.0 x 10^{-5} M solution in 2-MeTHF at 298 K.

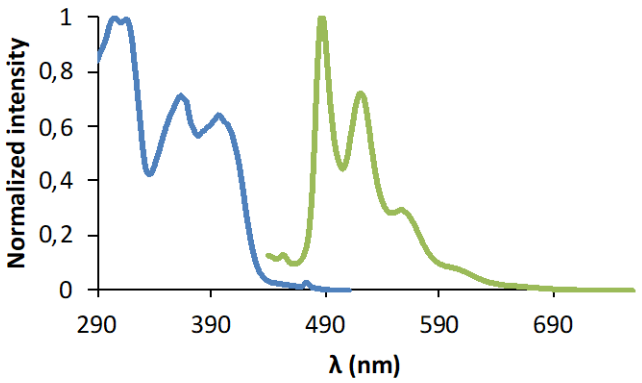

Figure S74. Normalized excitation (blue line) and emission (green line) spectra of **4d** in a 1.0 x 10^{-6} M solution in 2-MeTHF at 298 K.

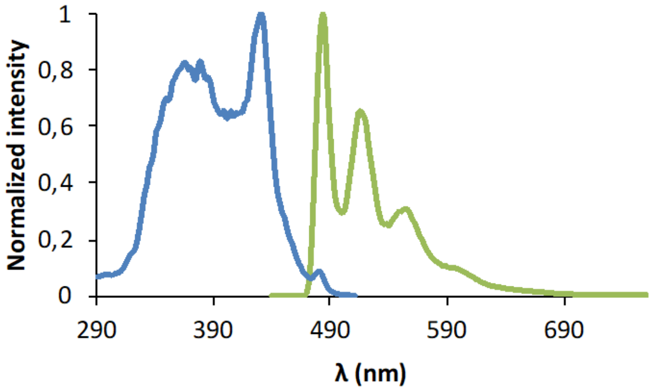

Figure S75. Normalized excitation (blue line) and emission (green line) spectra of **4d** in a 1.0 x 10^{-3} M solution in 2-MeTHF at 77 K.

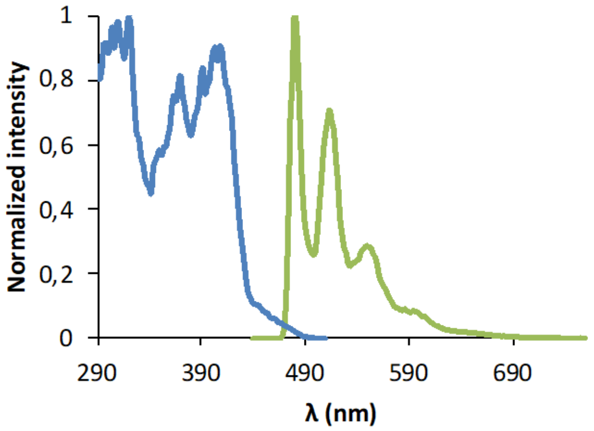

Figure S76. Normalized excitation (blue line) and emission (green line) spectra of **4d** in a 1.0 x 10^{-4} M solution in 2-MeTHF at 77 K.

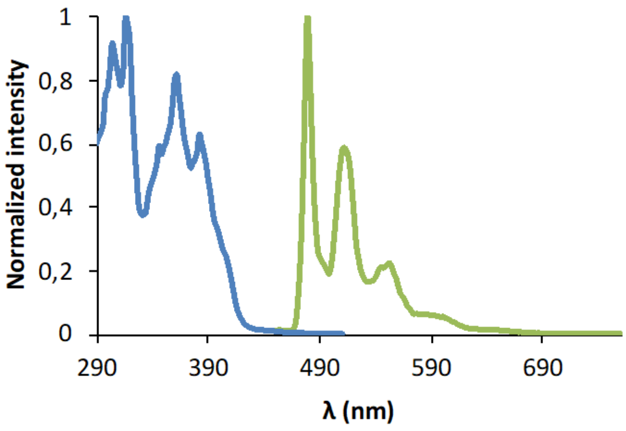

Figure S77. Normalized excitation (blue line) and emission (green line) spectra of **4d** in a 1.0 x 10^{-5} M solution in 2-MeTHF at 77 K.

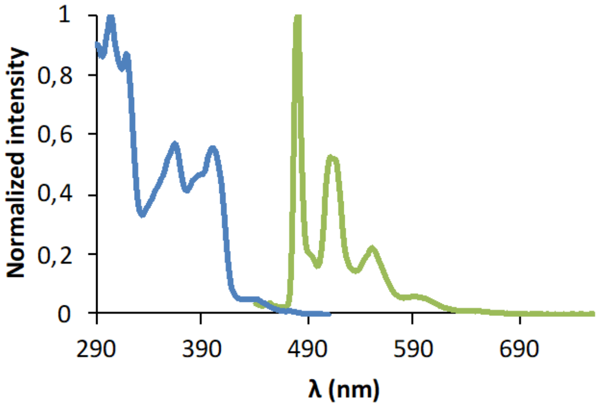

Figure S78. Normalized excitation (blue line) and emission (green line) spectra of **4d** in a 1.0 x 10^{-6} M solution in 2-MeTHF at 77 K.

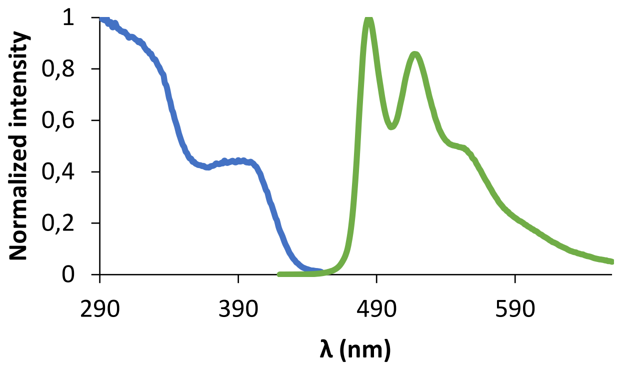

Figure S79. Normalized excitation (blue line) and emission (green line) spectra of **5a** in PMMA film (5 wt%) at 298 K.

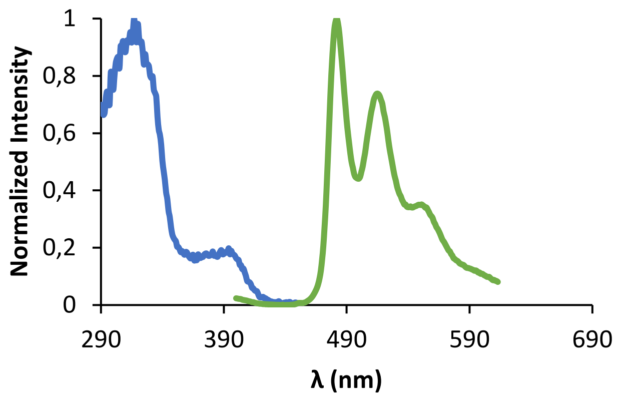

Figure S80. Normalized excitation (blue line) and emission (green line) spectra of **5a** in a 1.0 x 10^{-5} M solution in dichloromethane at 298 K.

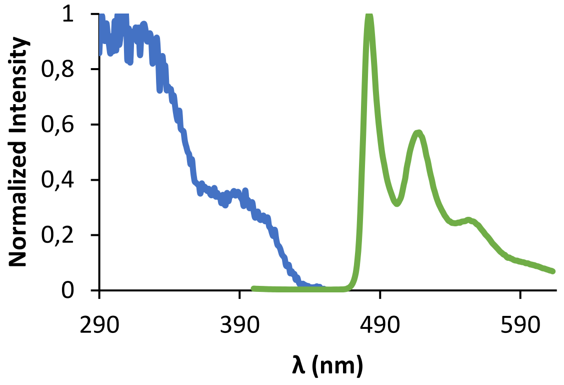

Figure S81. Normalized excitation (blue line) and emission (green line) spectra of **5a** in a 1.0 x 10^{5} M solution in dichloromethane at 77 K.

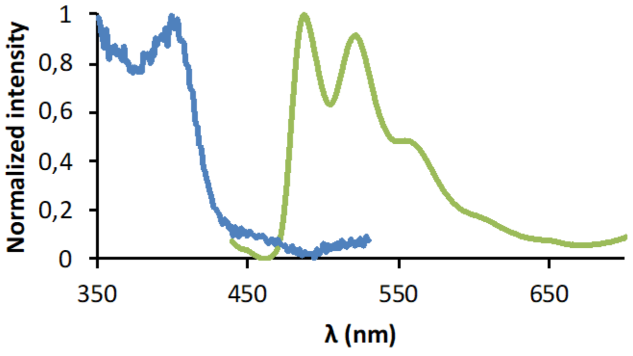

Figure S82. Normalized excitation (blue line) and emission (green line) spectra of **5a** in a 1.0 x 10^{-5} M solution in 2-MeTHF at 298 K.

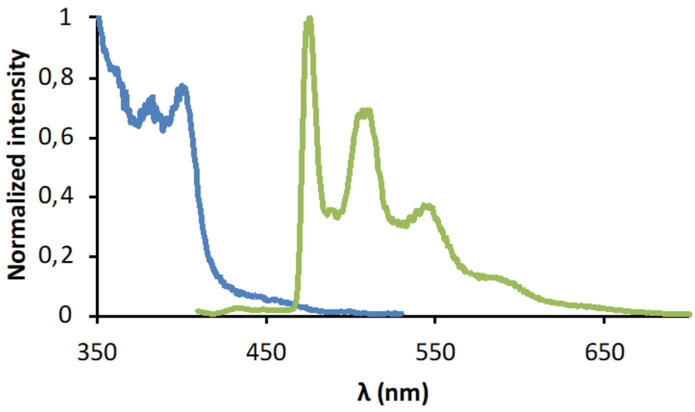

Figure S83. Normalized excitation (blue line) and emission (green line) spectra of **5a** in a 1.0 x 10^{-5} M solution in 2-MeTHF at 77 K.

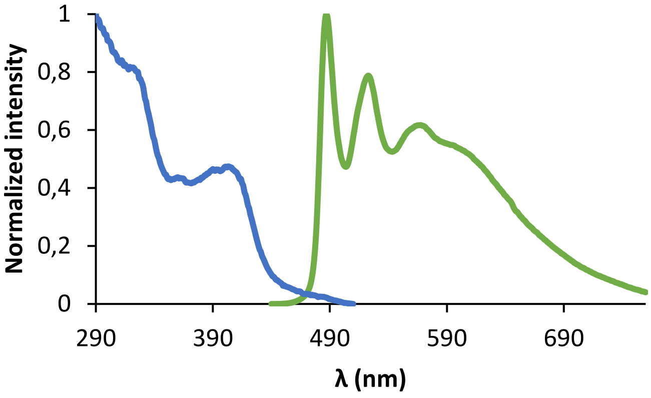

Figure S84. Normalized excitation (blue line) and emission (green line) spectra of **5c** in PMMA film (2 wt%) at 298 K.

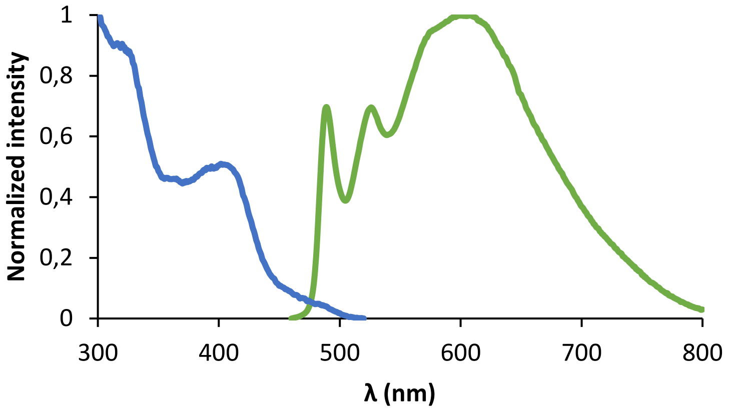

Figure S85. Normalized excitation (blue line) and emission (green line) spectra of **5c** in PMMA film (5 wt%) at 298 K.

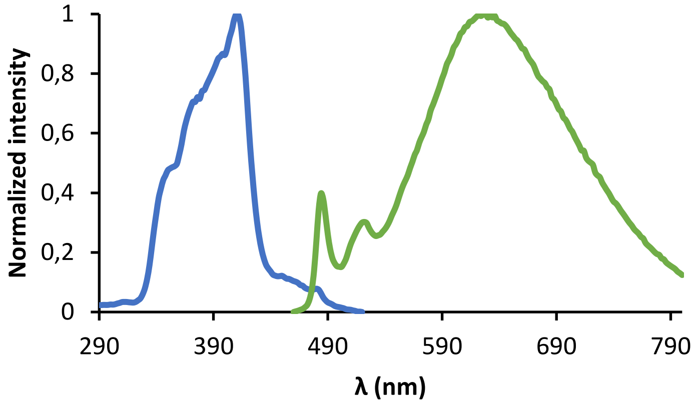

Figure S86. Normalized excitation (blue line) and emission (green line) spectra of **5c** in a 1.0 x 10^{-3} M solution in dichroromethane at 298 K.

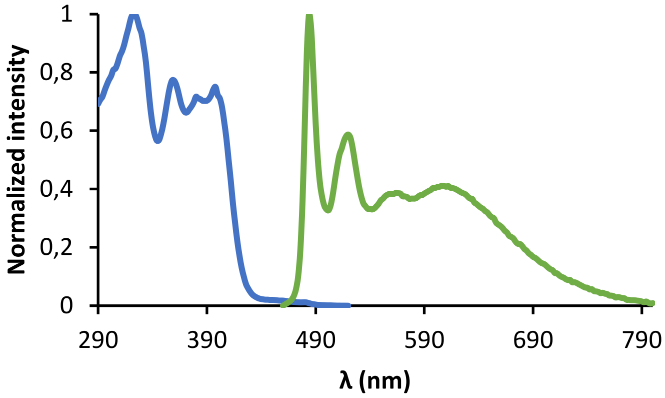

Figure S87. Normalized excitation (blue line) and emission (green line) spectra of **5c** in a 1.0 x 10^{-4} M solution in dichloromethane at 298 K.

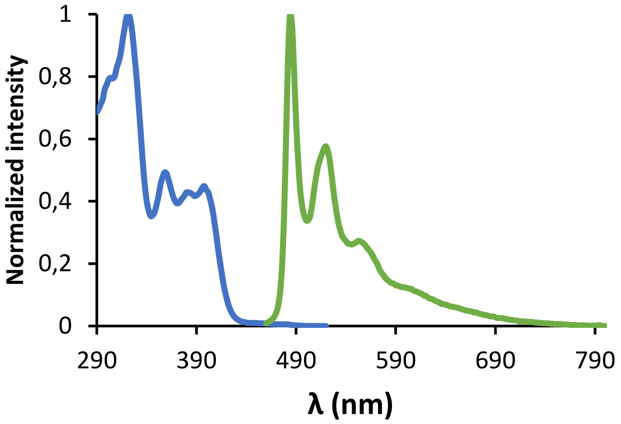

Figure S88. Normalized excitation (blue line) and emission (green line) spectra of **5c** in a 1.0 x 10^{-5} M solution in dichloromethane at 298 K.

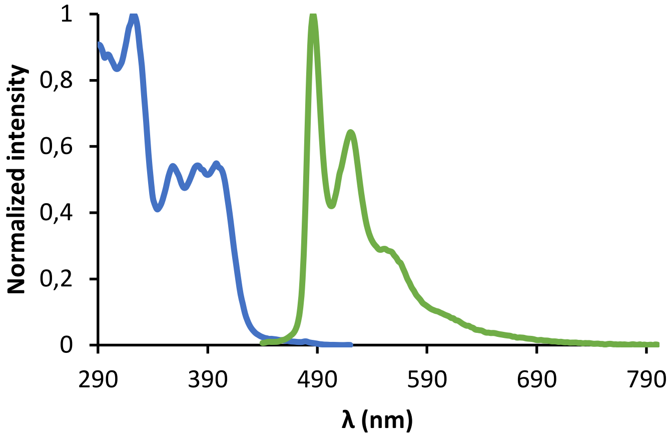

Figure S89. Normalized excitation (blue line) and emission (green line) spectra of **5c** in a 1.0 x 10^{-6} M solution in dichloromethane at 298 K.

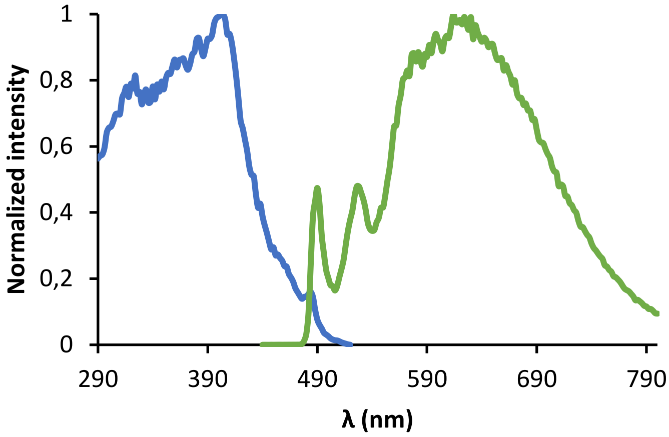

Figure S90. Normalized excitation (blue line) and emission (green line) spectra of **5c** in a 1.0 x 10^{-3} M solution in dichloromethane at 77 K.

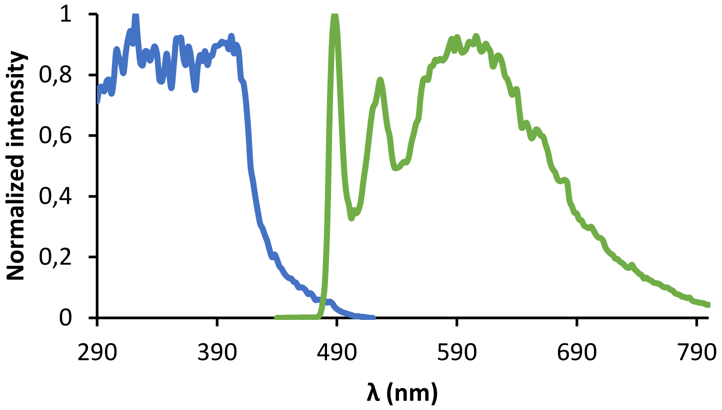

Figure S91. Normalized excitation (blue line) and emission (green line) spectra of **5c** in a 1.0 x 10^{-4} M solution in dichloromethane at 77 K.

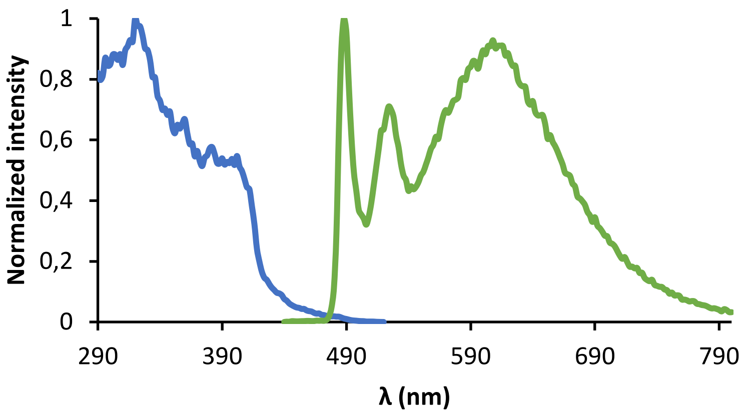

Figure S92. Normalized excitation (blue line) and emission (green line) spectra of **5c** in a 1.0 x 10^{-5} M solution in dichloromethane at 77 K.

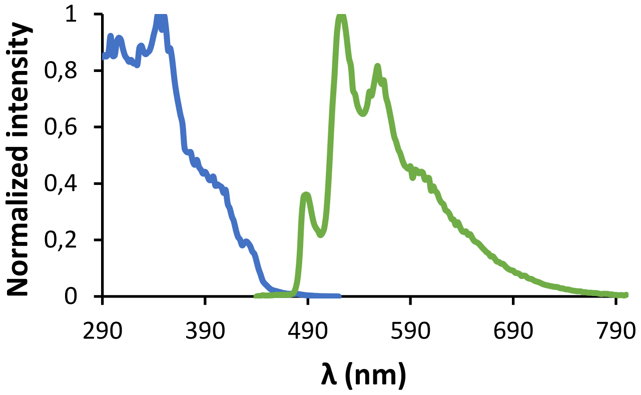

Figure S93. Normalized excitation (blue line) and emission (green line) spectra of **5c** in a 1.0 x 10^{-6} M solution in dichloromethane at 77 K.

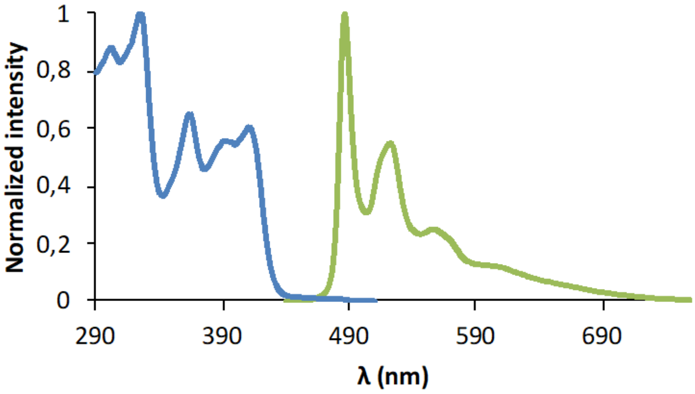

Figure S94. Normalized excitation (blue line) and emission (green line) spectra of **5c** in a 1.0 x 10^{-5} M solution in 2-MeTHF at 298 K.

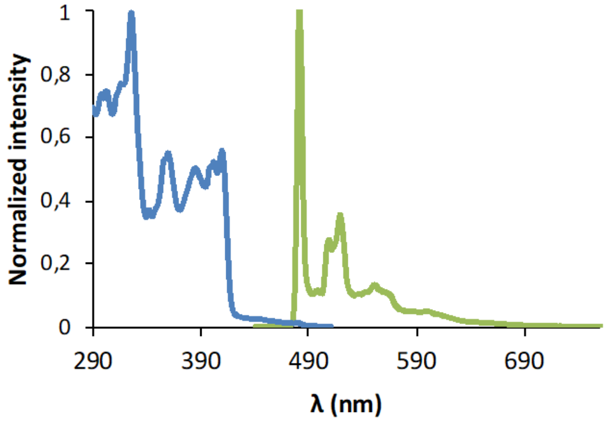

Figure S95. Normalized excitation (blue line) and emission (green line) spectra of **5c** in a 1.0 x 10^{-5} M solution in 2-MeTHF at 77 K.

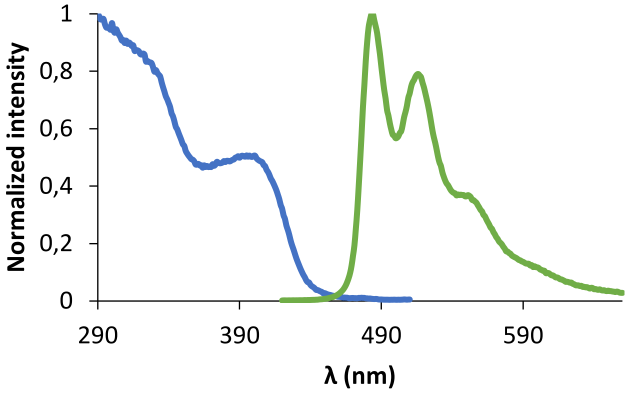

Figure S96. Normalized excitation (blue line) and emission (green line) spectra of **5d** in PMMA film (5 wt%) at 298 K.

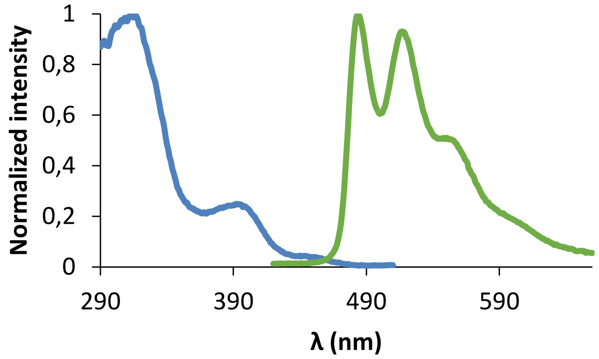

Figure S97. Normalized excitation (blue line) and emission (green line) spectra of **5d** in a 1.0 x 10^{-5} M solution in dichloromethane at 298 K.

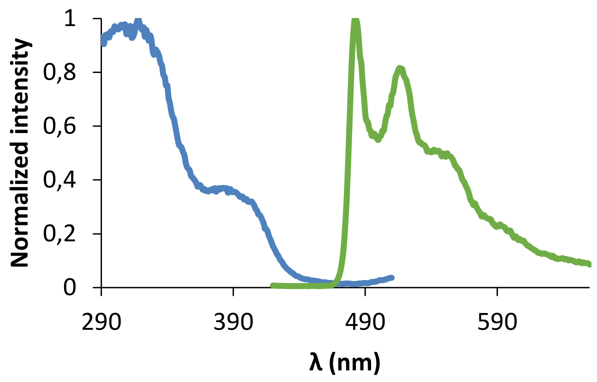

Figure S98. Normalized excitation (blue line) and emission (green line) spectra of **5d** in a 1.0 x 10^{-5} M solution in dichloromethane at 77 K.

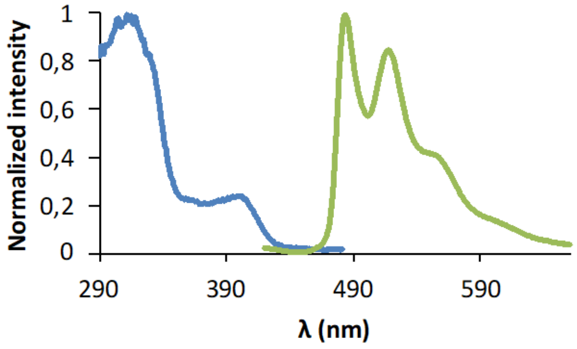

Figure S99. Normalized excitation (blue line) and emission (green line) spectra of **5d** in a 1.0 x 10^{-5} M solution in 2-MeTHF at 298 K.

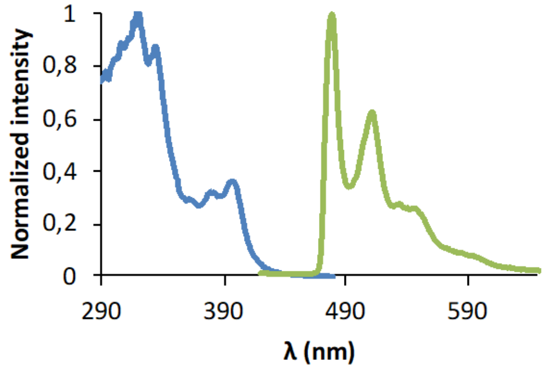

Figure S100. Normalized excitation (blue line) and emission (green line) spectra of **5d** in a 1.0 x 10^{-5} M solution in 2-MeTHF at 77 K.

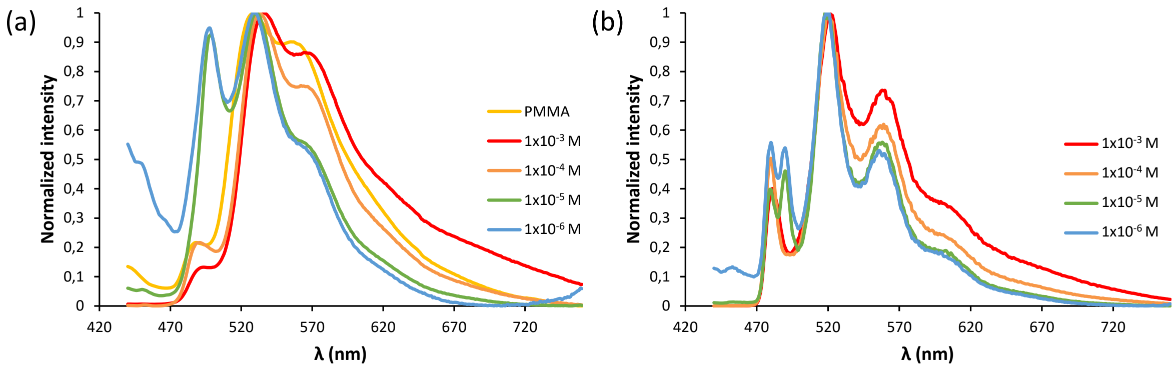

Figure S101. (a) Emission spectra of **4a** at 298 K in $PMMA_{5\%}$ and in 2-MeTHF at concentrations between $1x10^{-3}$ and $1x10^{-6}$ M. (b) Emission spectra of **4a** at 77 K in 2-MeTHF at concentrations between $1x10^{-3}$ and $1x10^{-6}$ M.

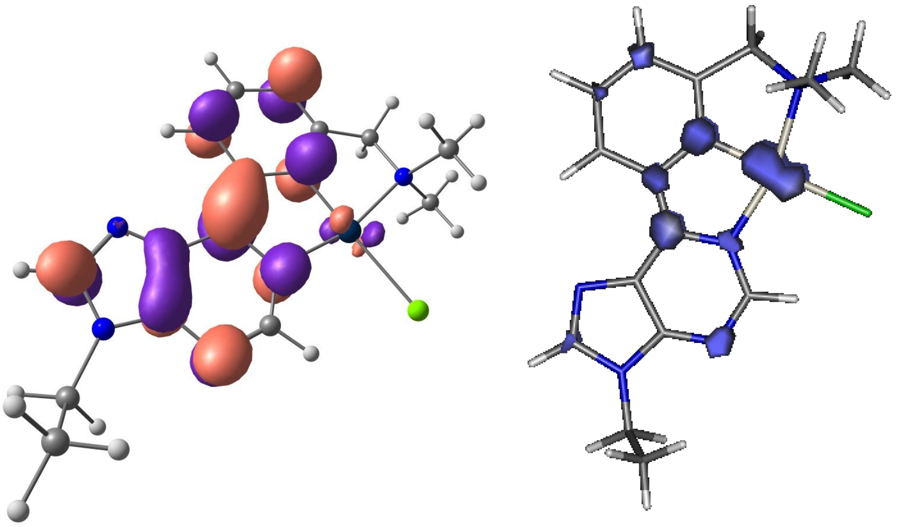

Figure S102. Computed (B3LYP-D3/def2-SVP) SOMO (left) and spin density (right) of the T1 of **3a**.

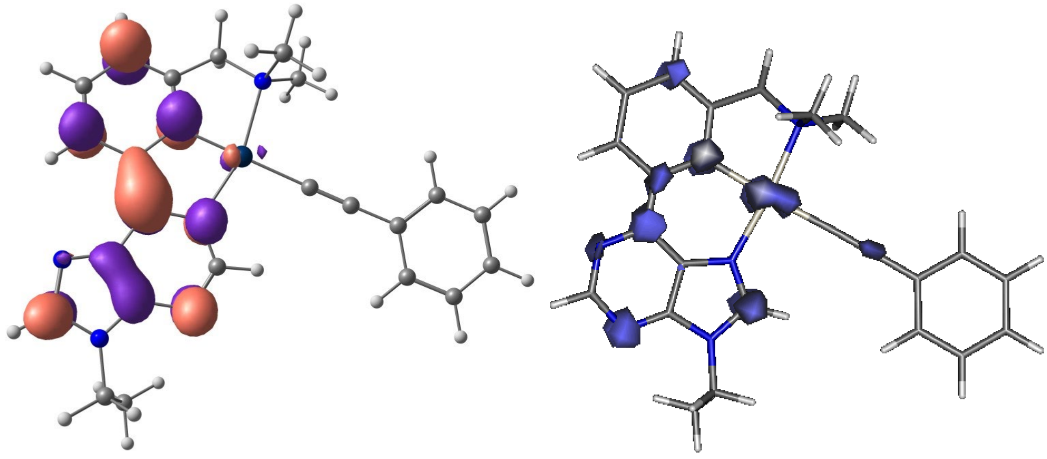

Figure S103. Computed (B3LYP-D3/def2-SVP) SOMO (left) and spin density (right) of the T1 of **4a**.

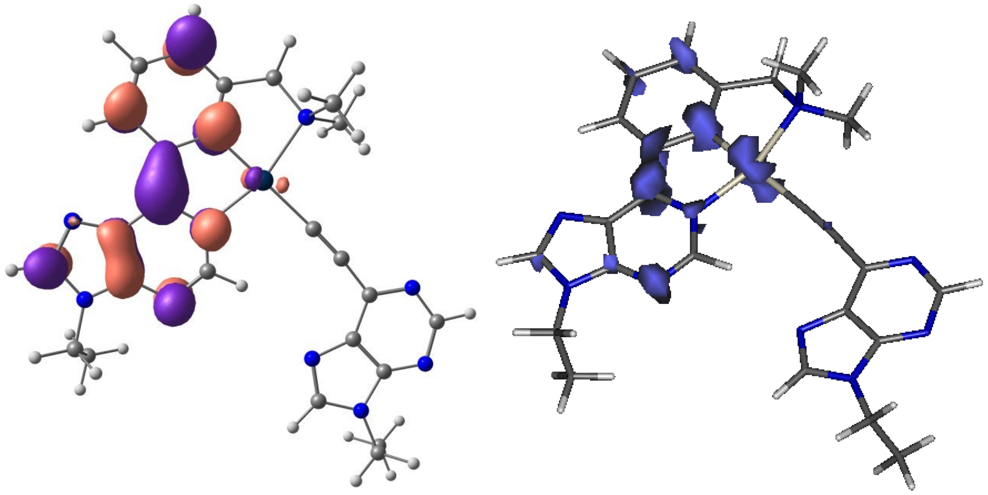

Figure S104. Computed (B3LYP-D3/def2-SVP) SOMO (left) and spin density (right) of the T1 of **5a**.

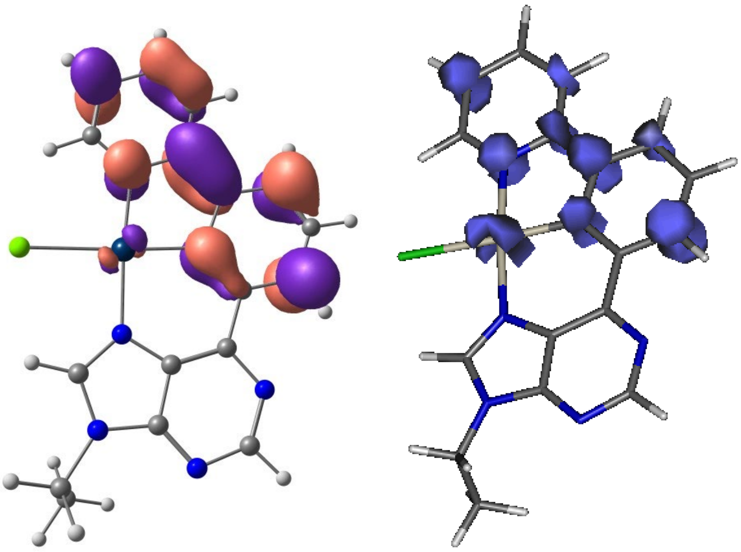

Figure S105. Computed (B3LYP-D3/def2-SVP) SOMO (left) and spin density (right) of the T1 of **3c**.

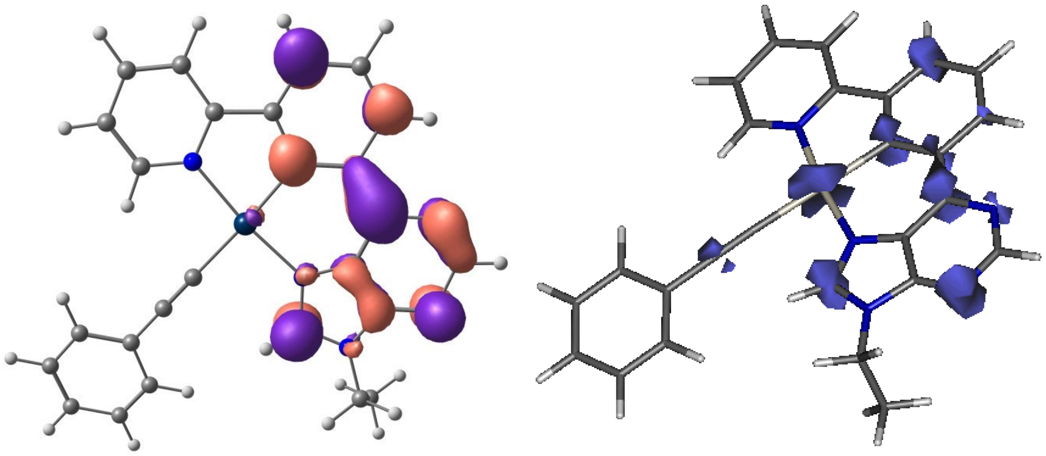

Figure S106. Computed (B3LYP-D3/def2-SVP) SOMO (left) and spin density (right) of the T1 of **4c**.

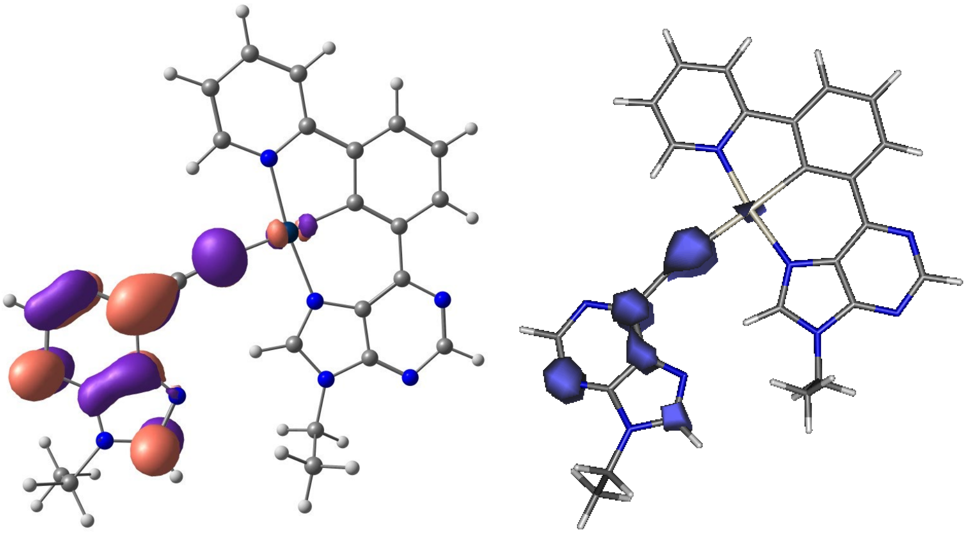

Figure S107 Computed (B3LYP-D3/def2-SVP) SOMO (left) and spin density (right) of the T1 of **5c**.

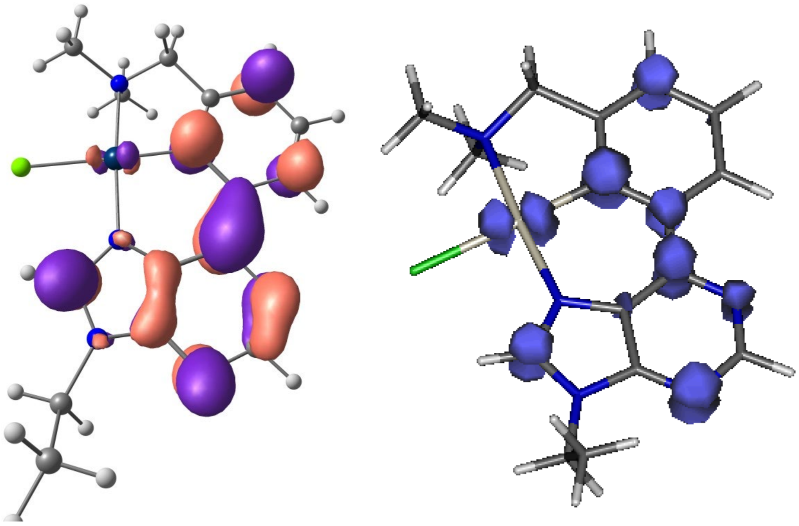

Figure S108 Computed (B3LYP-D3/def2-SVP) SOMO (left) and spin density (right) of the T1 of **3d**.

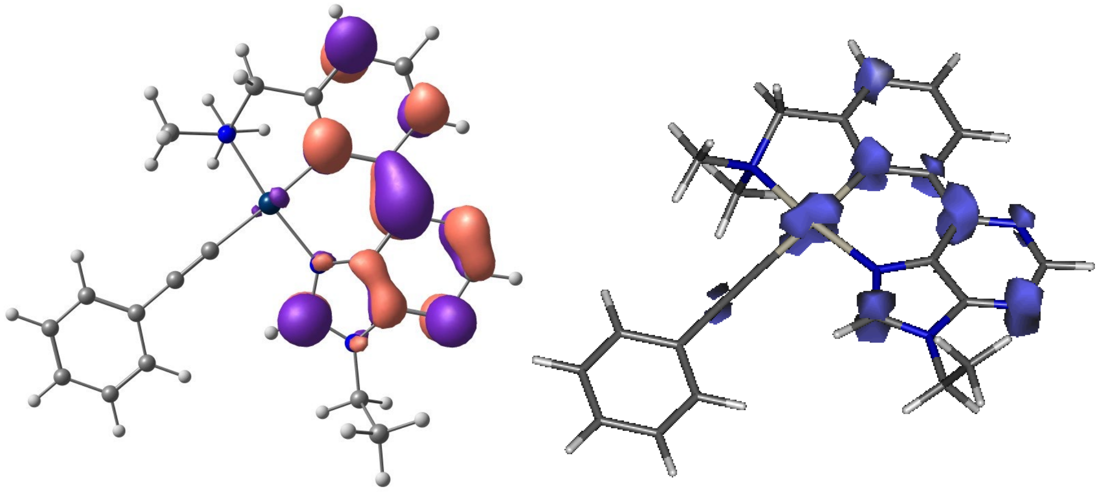

Figure S109 Computed (B3LYP-D3/def2-SVP) SOMO (left) and spin density (right) of the T1 of **4d**.

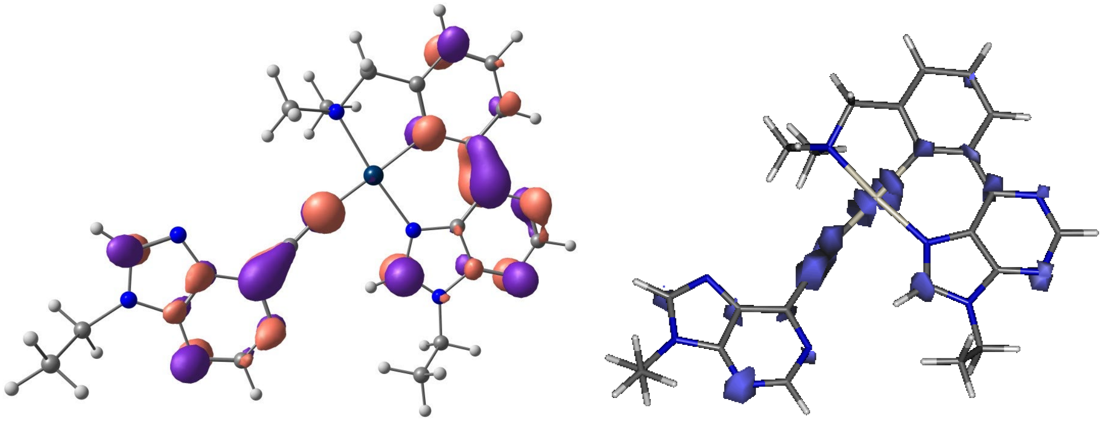

Figure S110 Computed (B3LYP-D3/def2-SVP) SOMO (left) and spin density (right) of the T1 of **5d**.

S.7. NMR Spectra

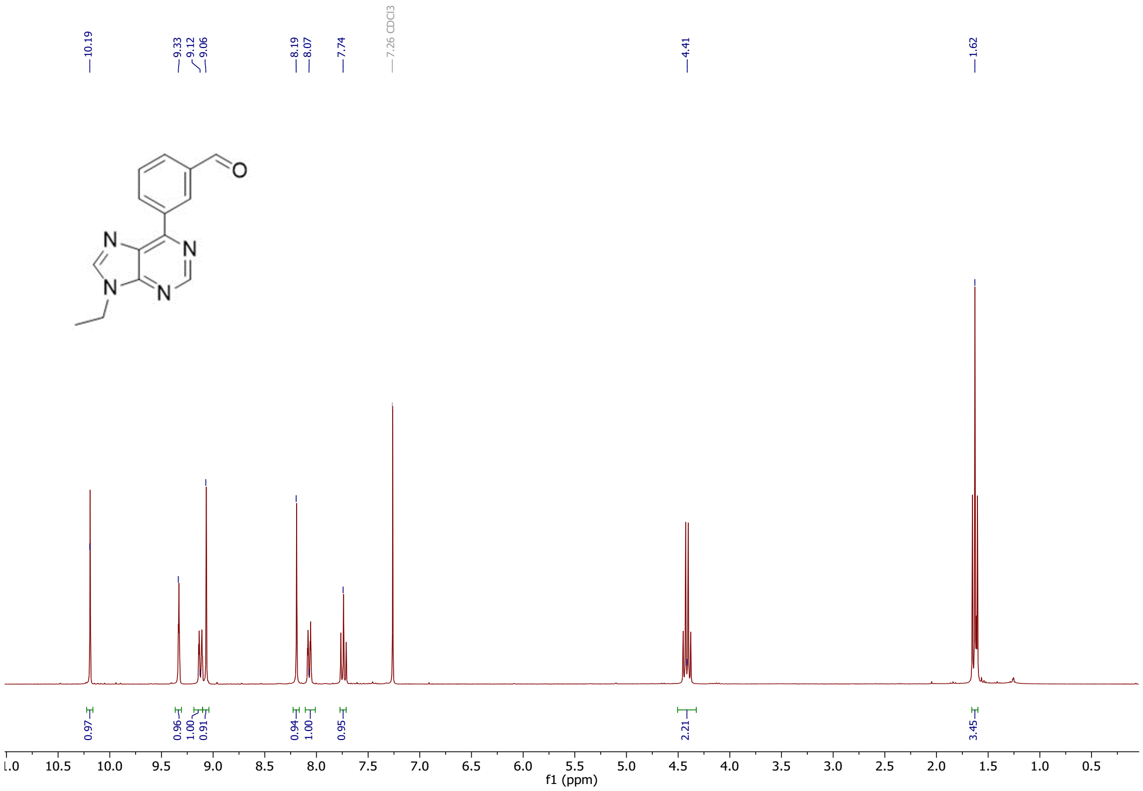

Figure S111. ^{1}H NMR (300 MHz, $CDCl_3$, 298 K) spectrum of **1**.

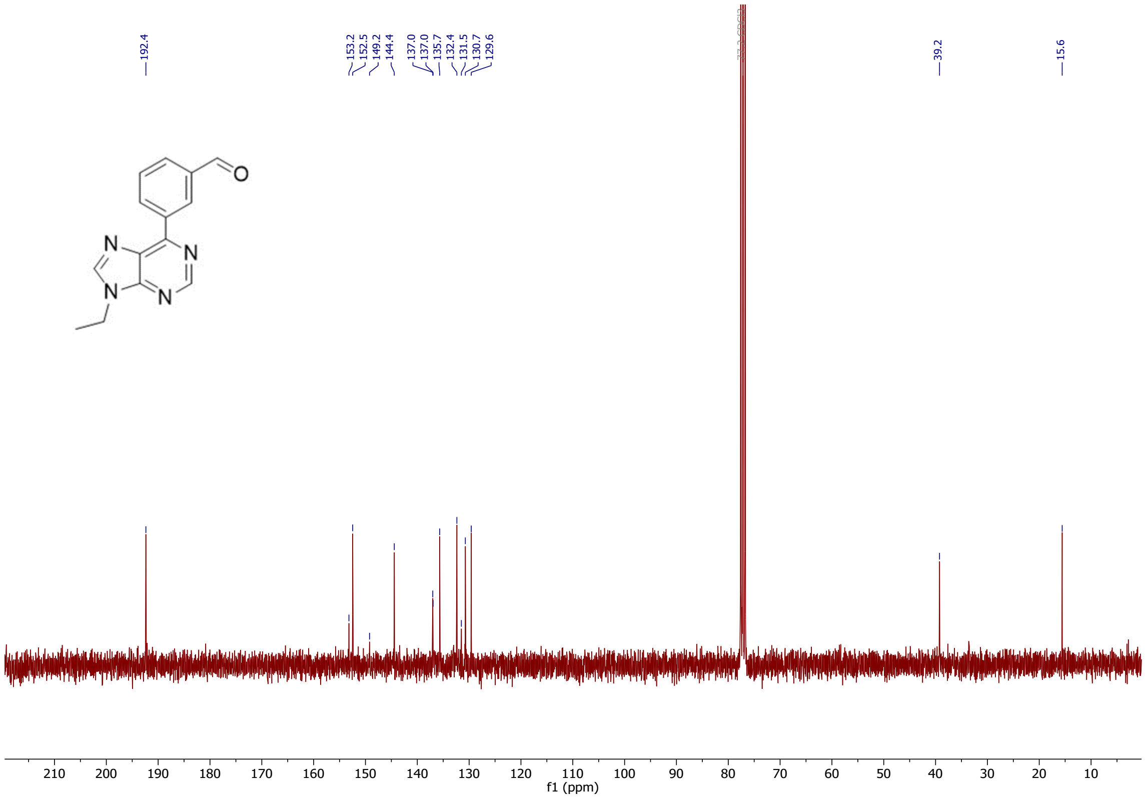

Figure S112. ^{13}C NMR (75 MHz, $CDCl_3$, 298 K) spectrum of **1**.

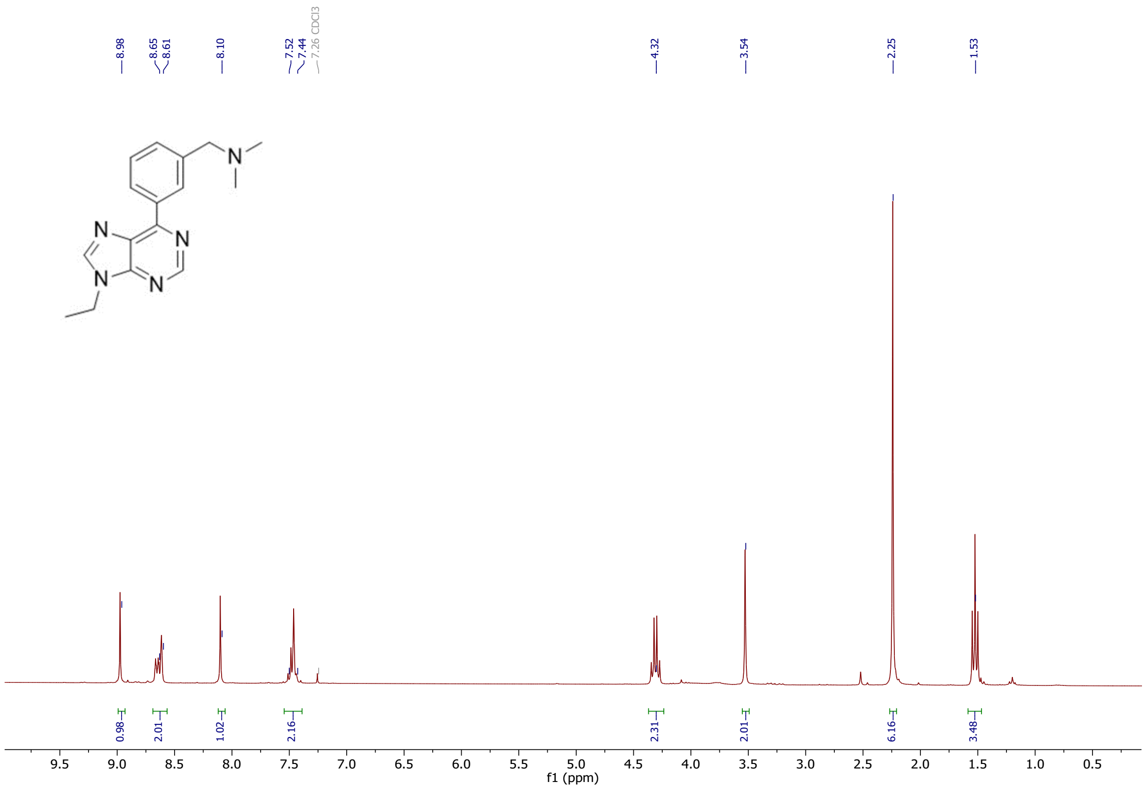

Figure S113. ^{1}H NMR (300 MHz, $CDCl_3$, 298 K) spectrum of **2a**.

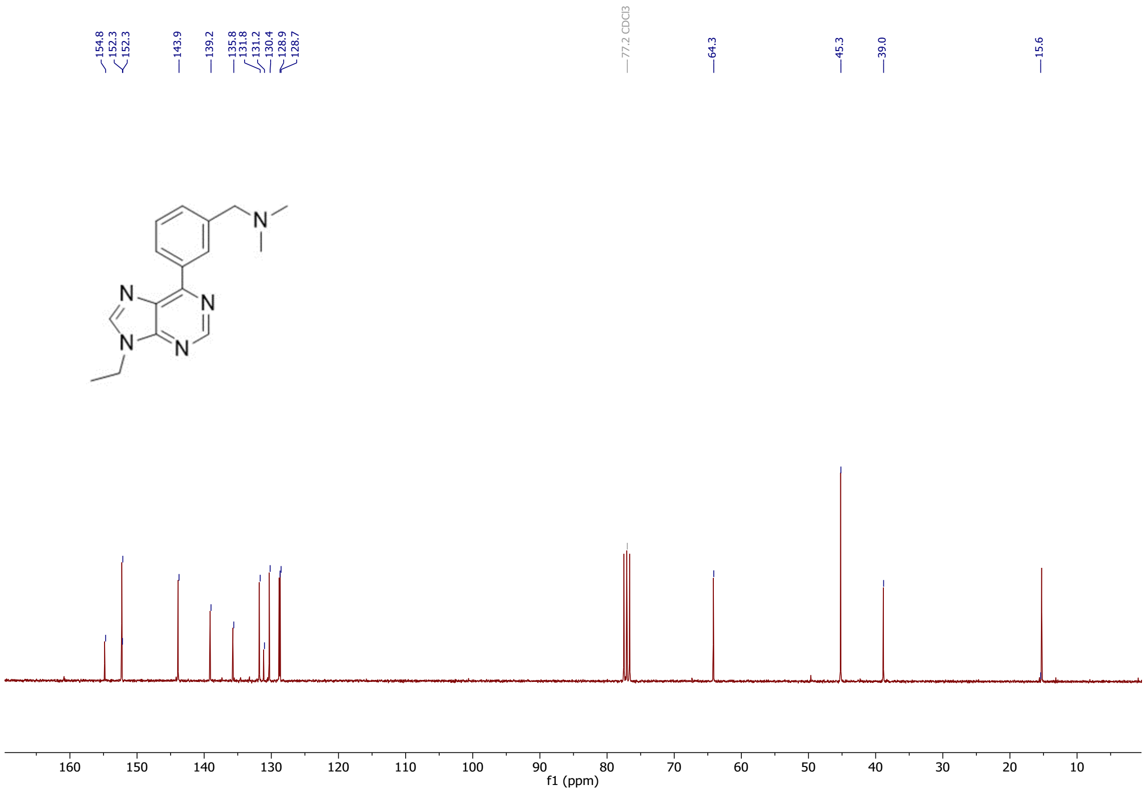

Figure S114. ^{13}C NMR (75 MHz, $CDCl_3$, 298 K) spectrum of **2a**.

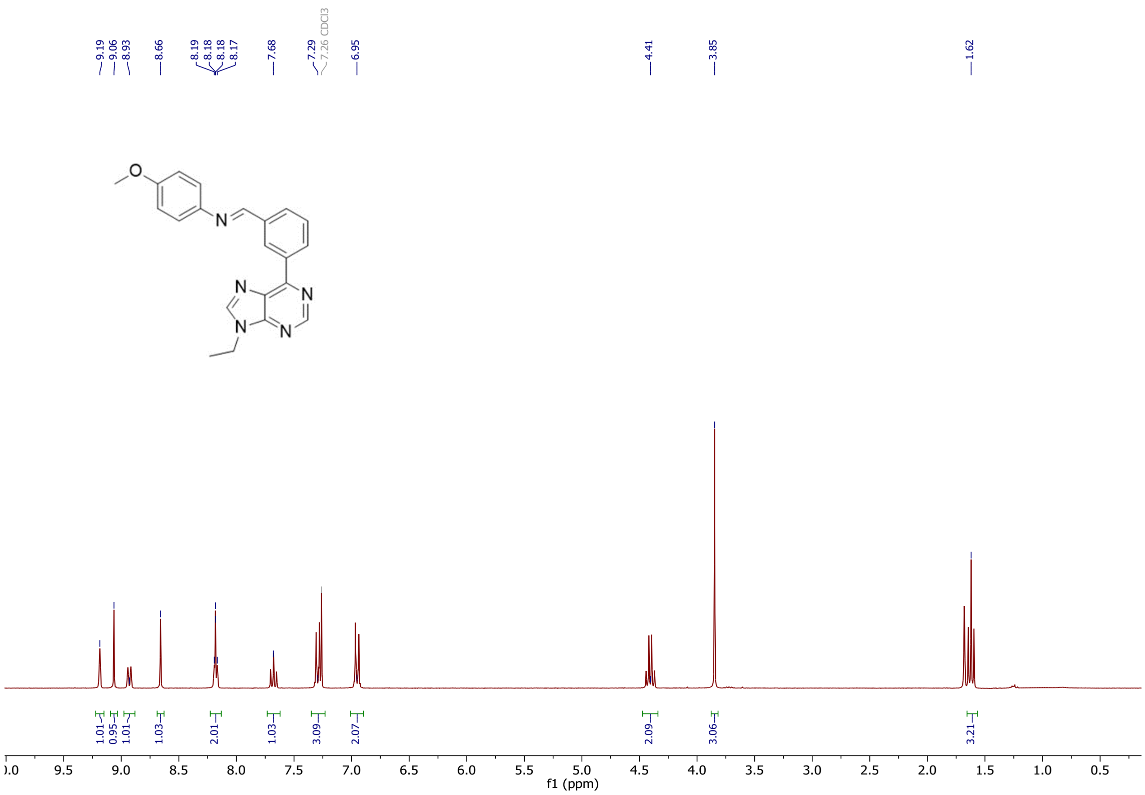

Figure S115. ^{1}H NMR (300 MHz, $CDCl_3$, 298 K) spectrum of **2b**.

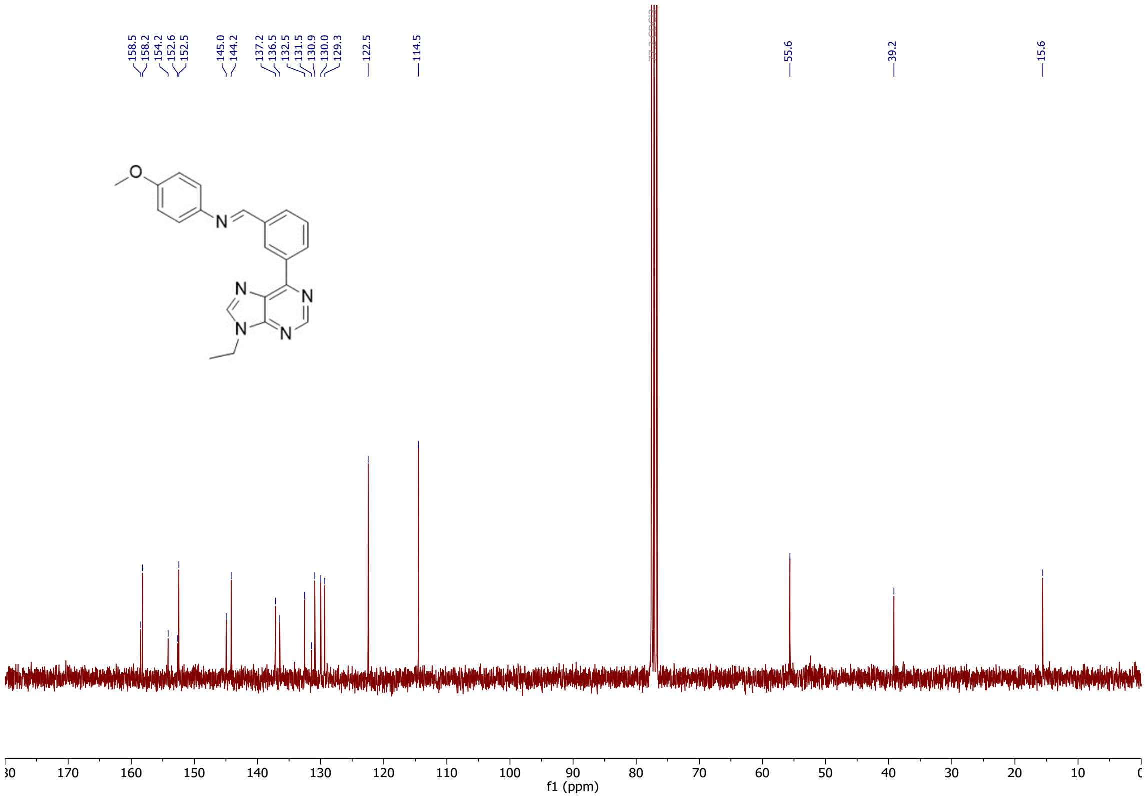

Figure S116. ^{13}C NMR (75 MHz, $CDCl_3$, 298 K) spectrum of **2b**.

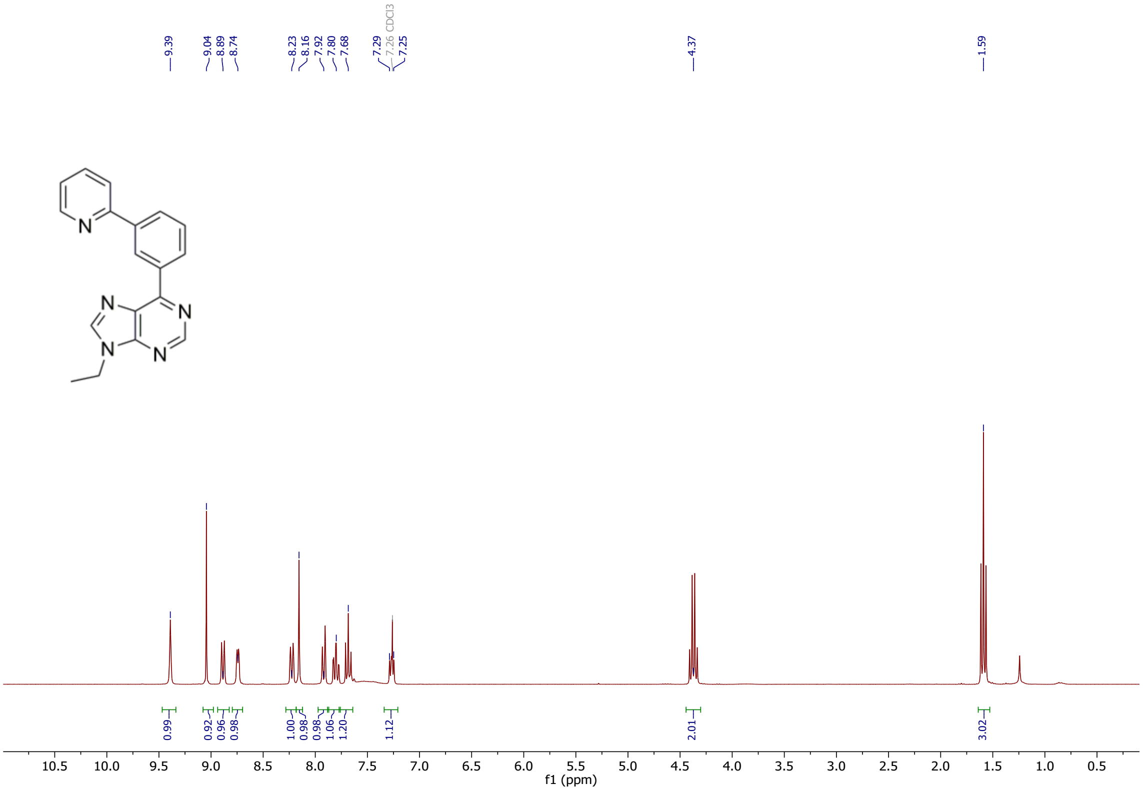

Figure S117. ^{1}H NMR (300 MHz, $CDCl_3$, 298 K) spectrum of **2c**.

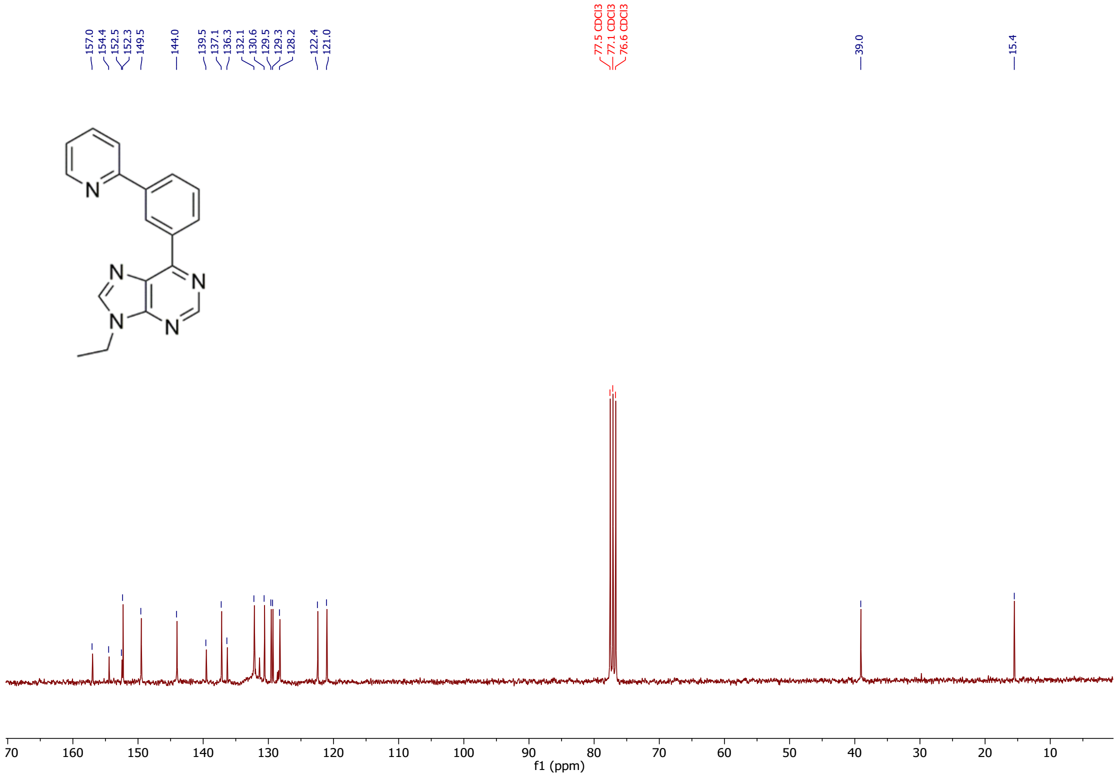

Figure S118. ^{13}C NMR (75 MHz, $CDCl_3$, 298 K) spectrum of **2c**.

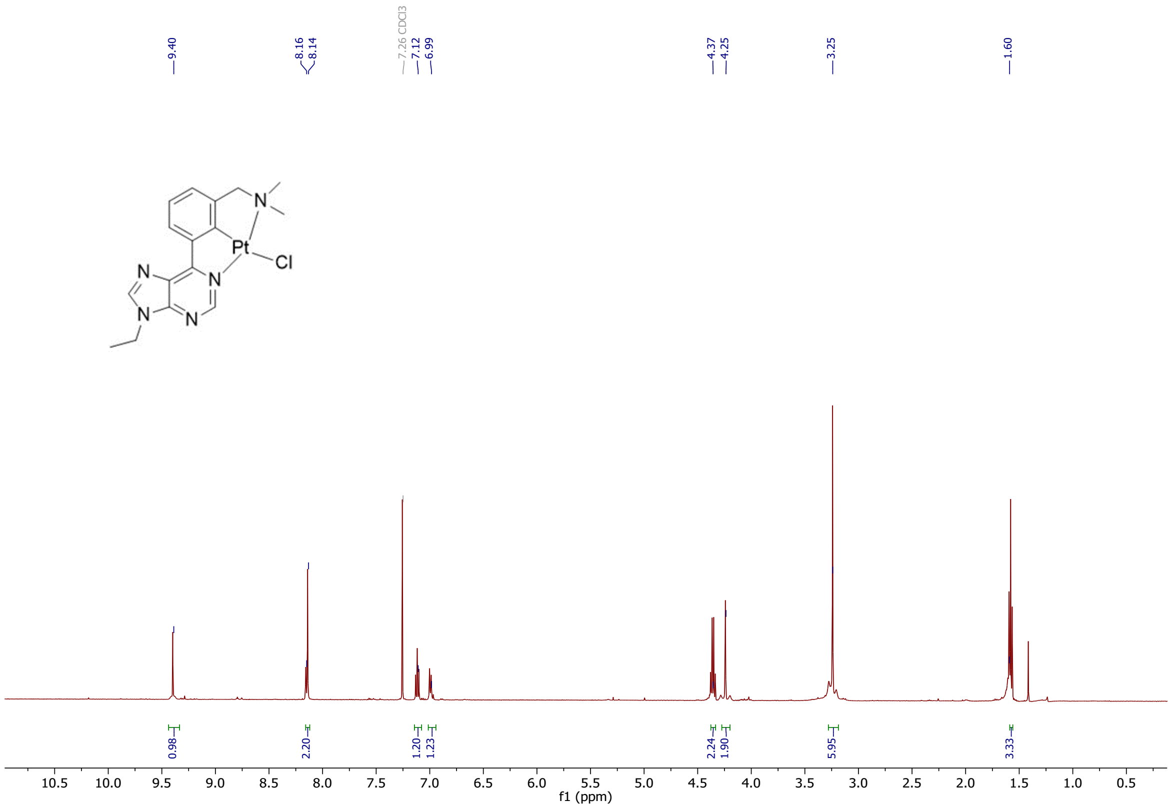

Figure S119. ^{1}H NMR (500 MHz, $CDCl_3$, 298 K) spectrum of **3a**.

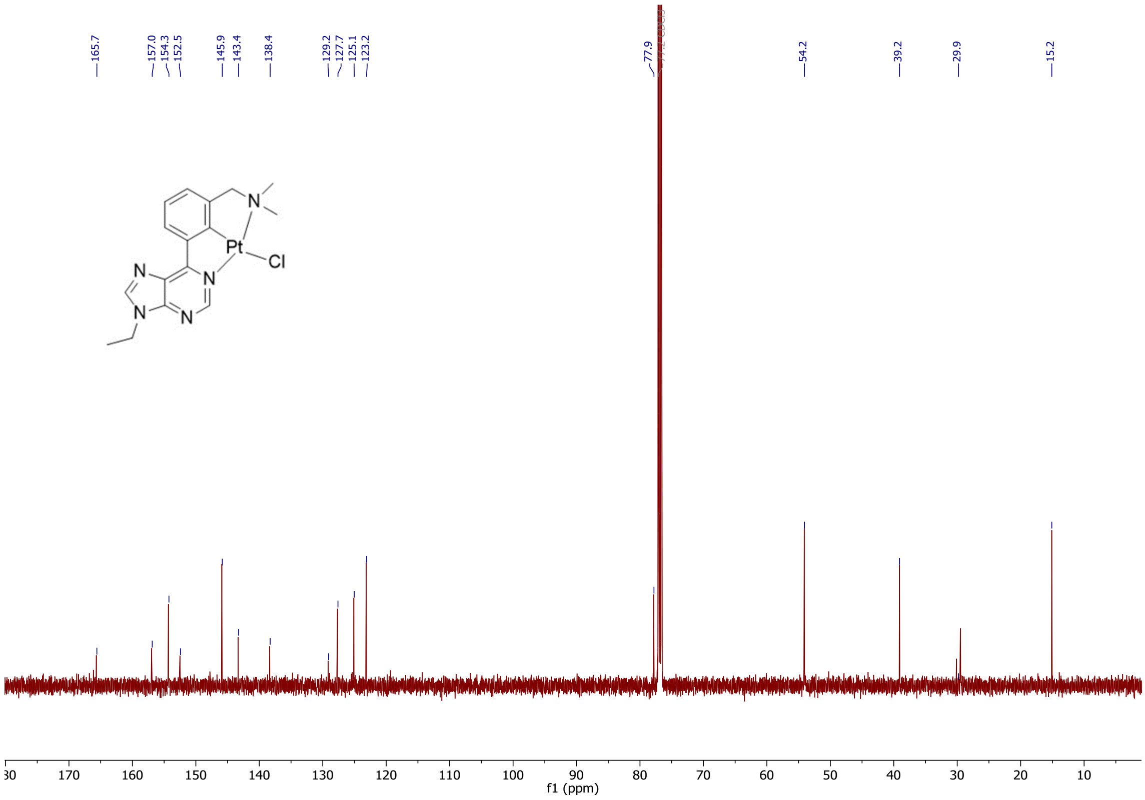

Figure S120. ^{13}C NMR (126 MHz, $CDCl_3$, 298 K) spectrum of **3a**.

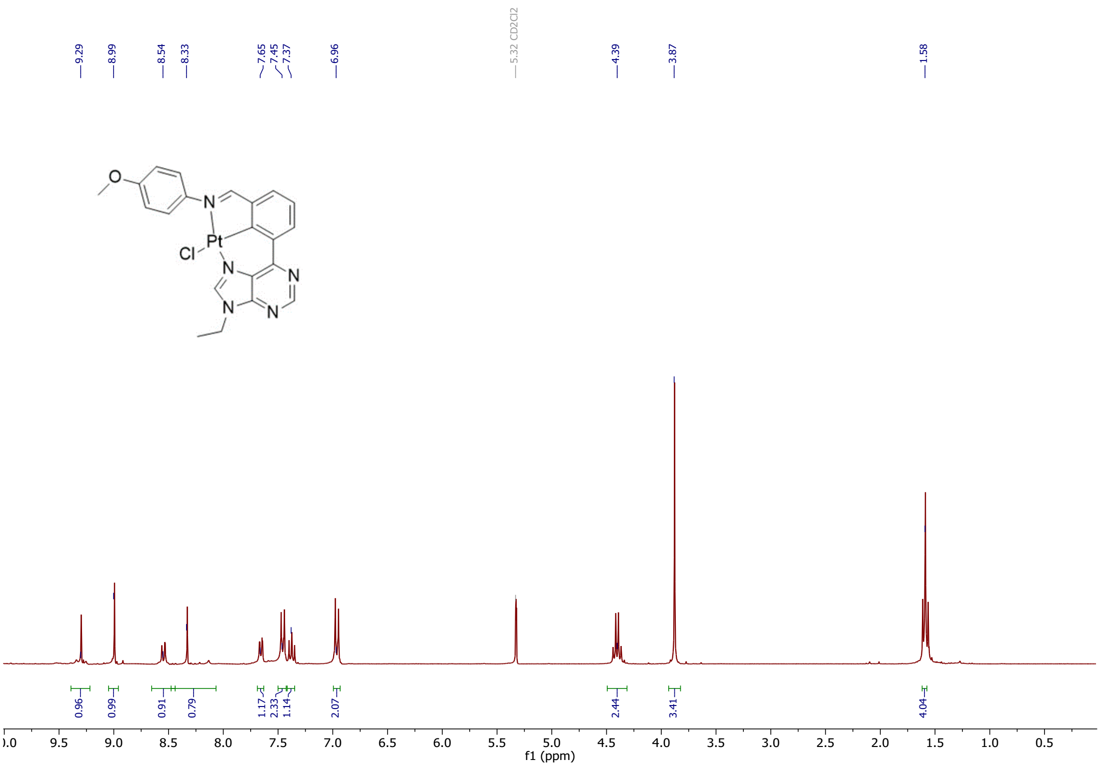

Figure S121. ^{1}H NMR (500 MHz, CD_2Cl_2, 298 K) spectrum of **3b**.

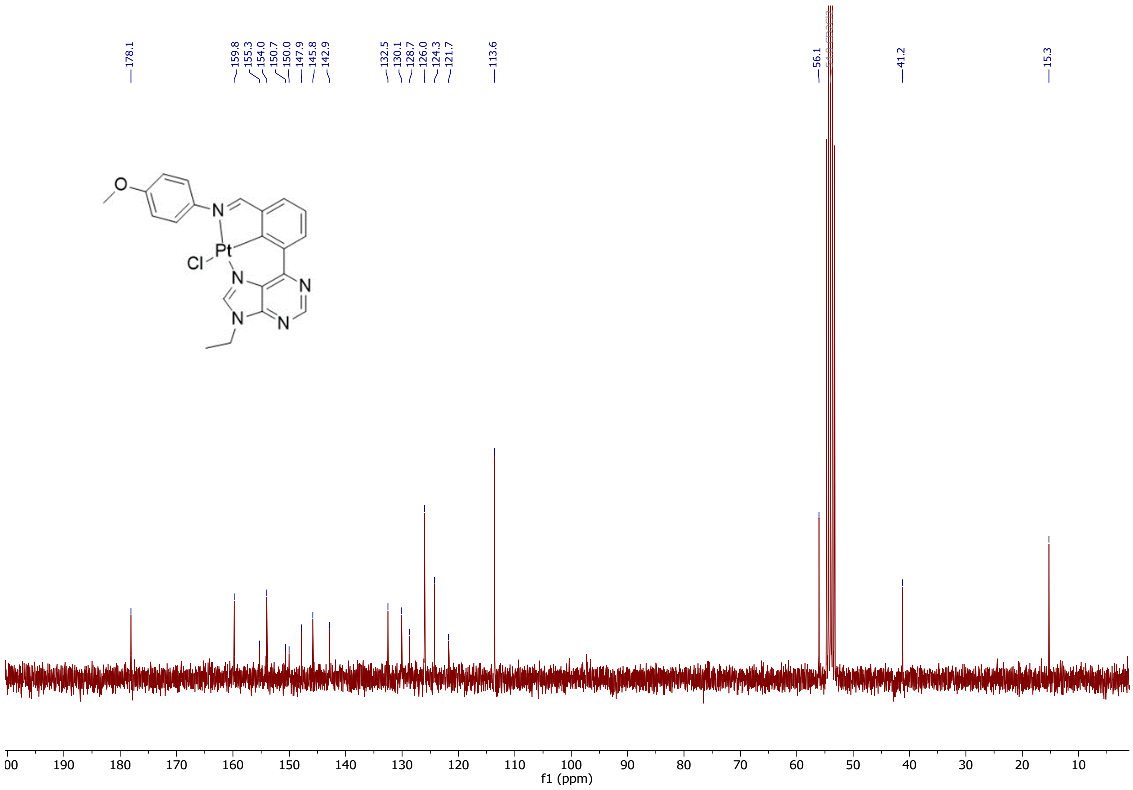

Figure S122. ^{13}C NMR (126 MHz, CD_2Cl_2, 298 K) spectrum of **3b**.

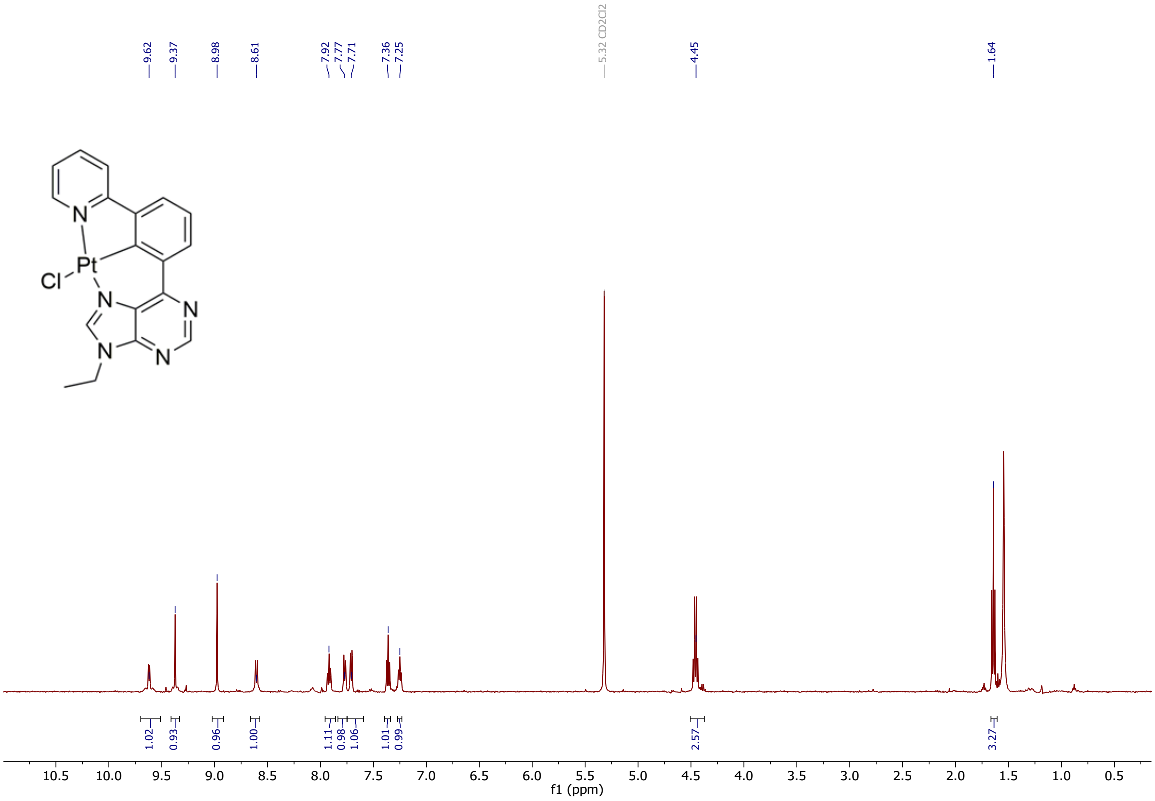

Figure S123. ^{1}H NMR (500 MHz, CD_2Cl_2, 298 K) spectrum of **3c**.

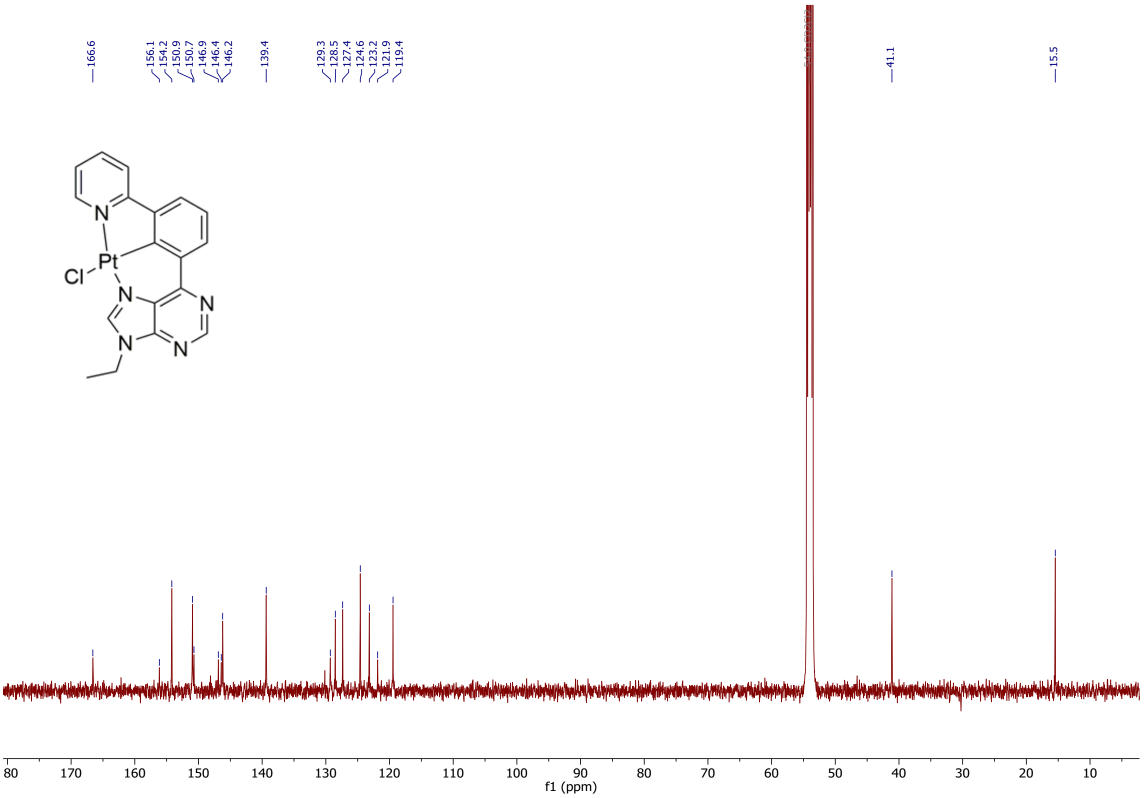

Figure S124. ^{13}C NMR (126 MHz, CD_2Cl_2, 298 K) spectrum of **3c**.

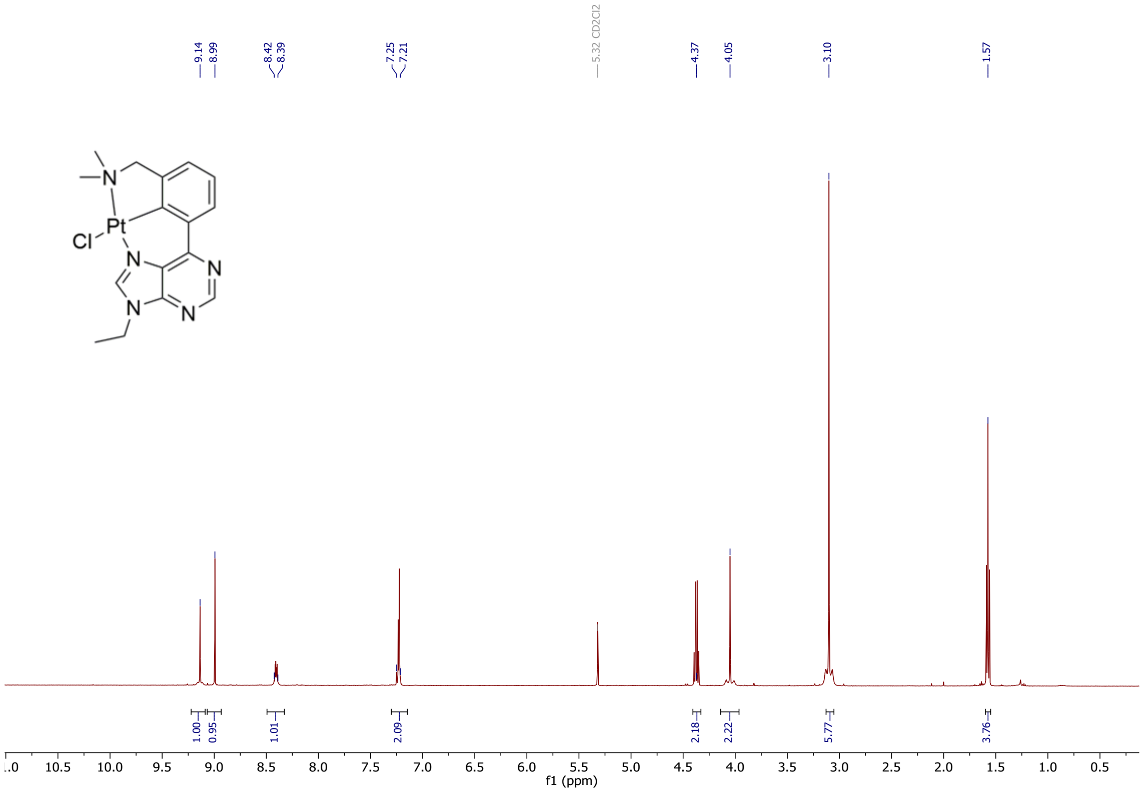

Figure S125. ^{1}H NMR (500 MHz, CD_2Cl_2, 298 K) spectrum of **3d**.

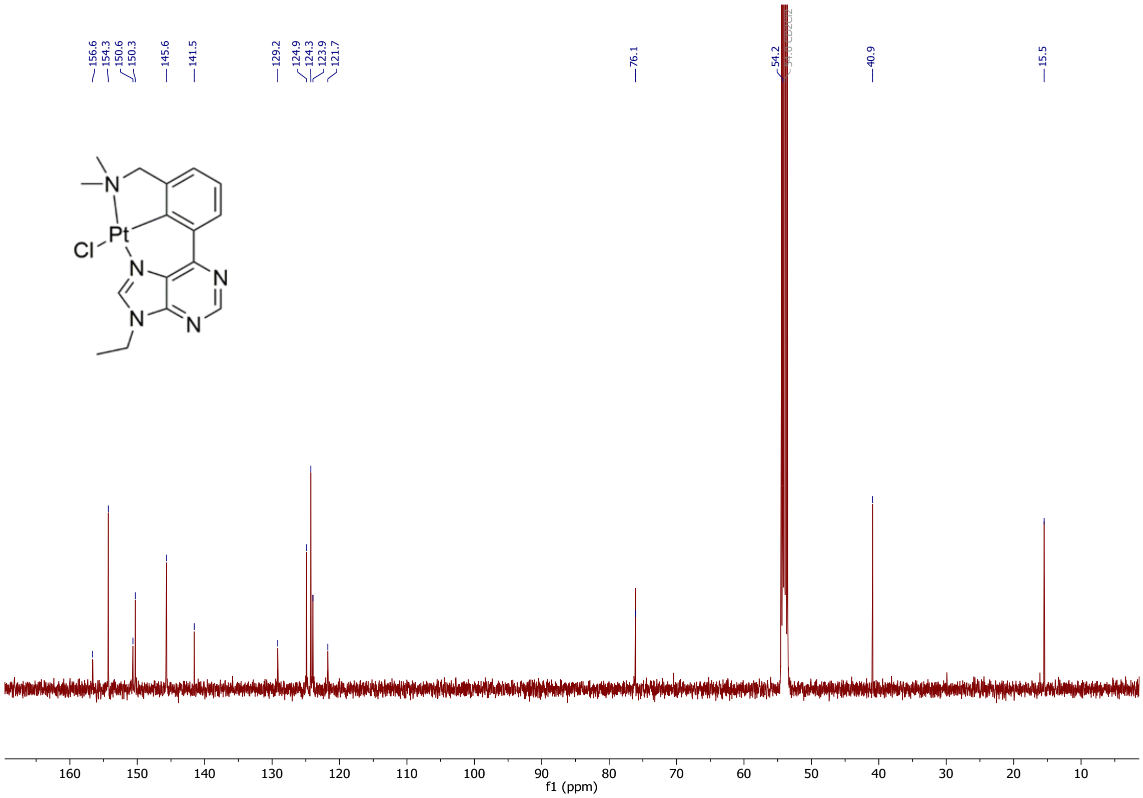

Figure S126. ^{13}C NMR (126 MHz, CD_2Cl_2, 298 K) spectrum of **3d**.

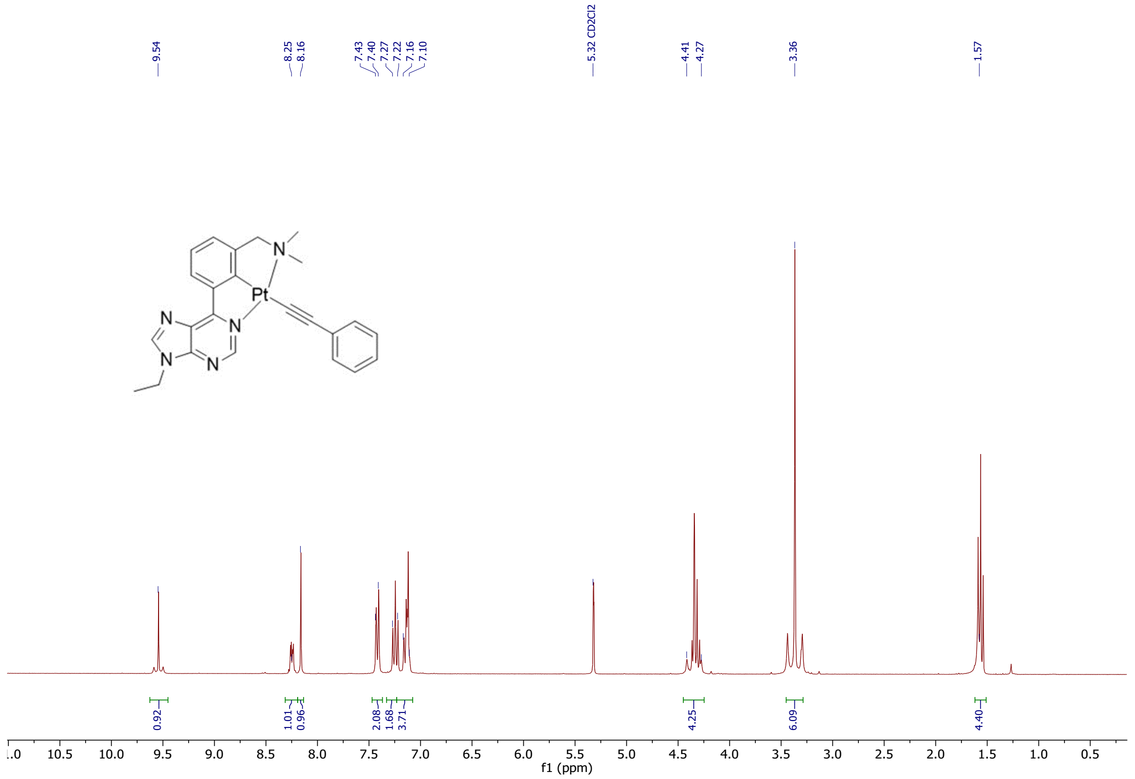

Figure S127. ^{1}H NMR (500 MHz, CD_2Cl_2, 298 K) spectrum of **4a**.

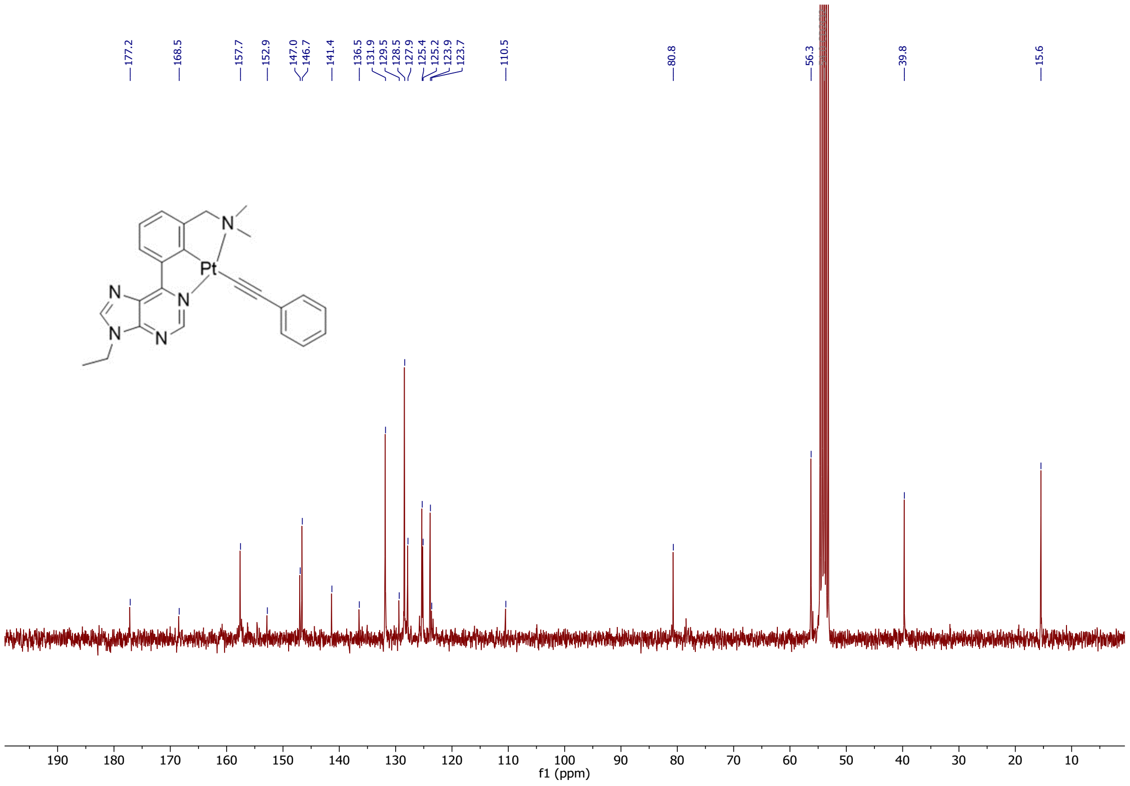

Figure S128. ^{13}C NMR (126 MHz, CD_2Cl_2, 298 K) spectrum of **4a**.

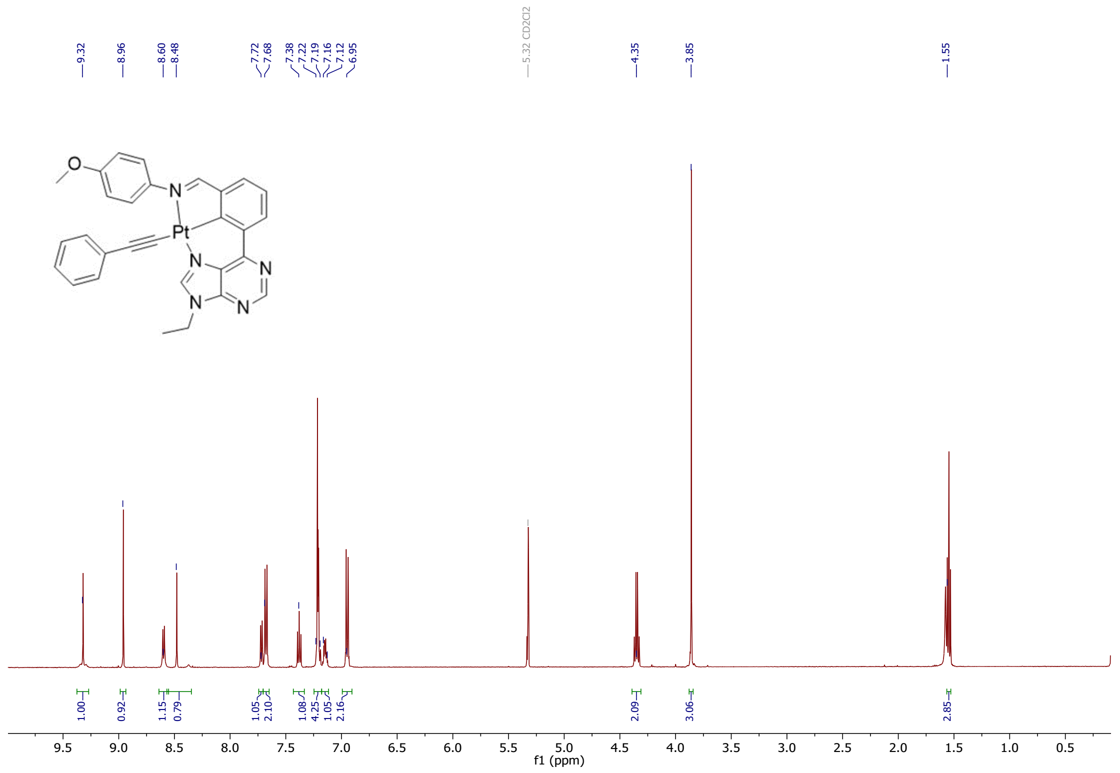

Figure S129. ^{1}H NMR (500 MHz, CD_2Cl_2, 298 K) spectrum of **4b**.

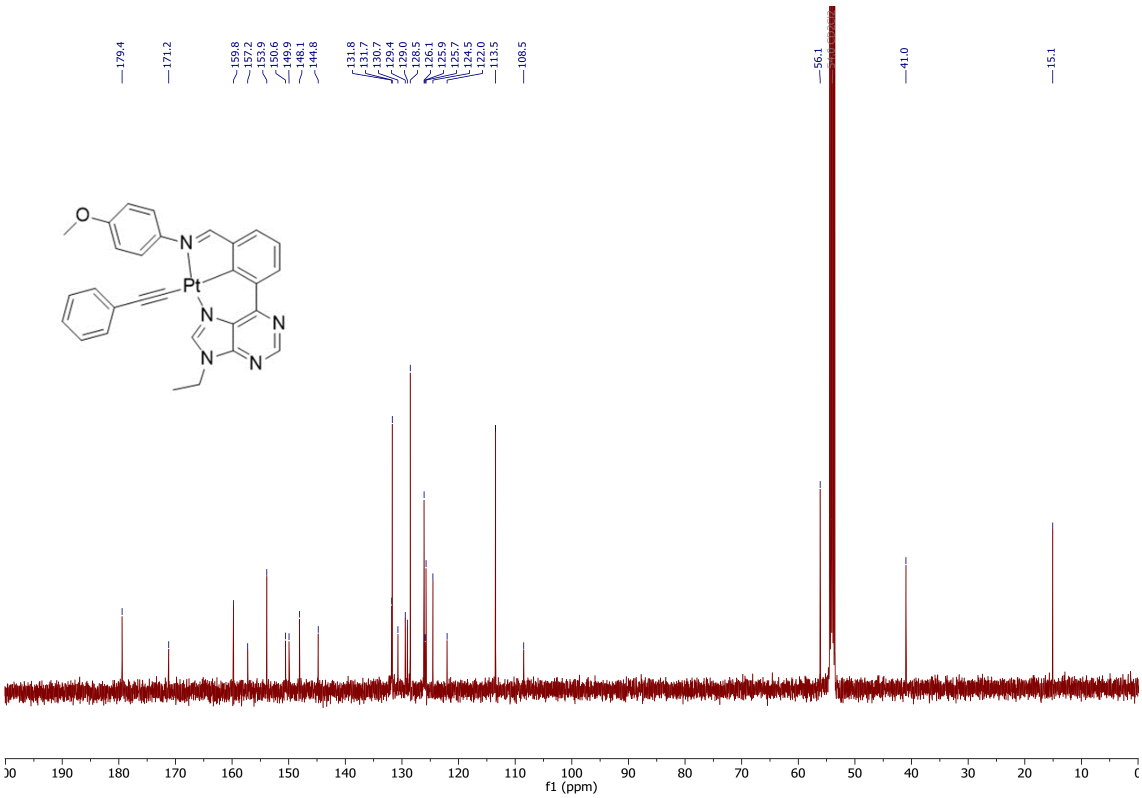

Figure S130. ^{13}C NMR (126 MHz, CD_2Cl_2, 298 K) spectrum of **4b**.

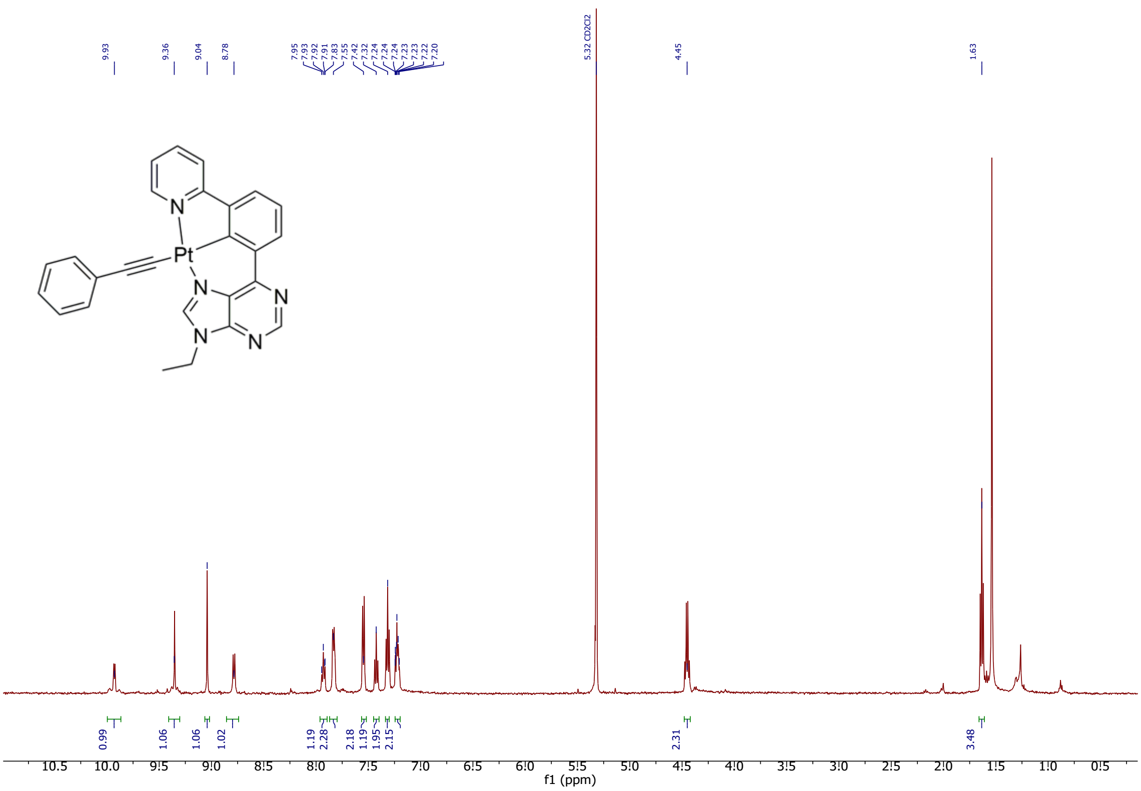

Figure S131. ^{1}H NMR (500 MHz, CD_2Cl_2, 298 K) spectrum of **4c**.

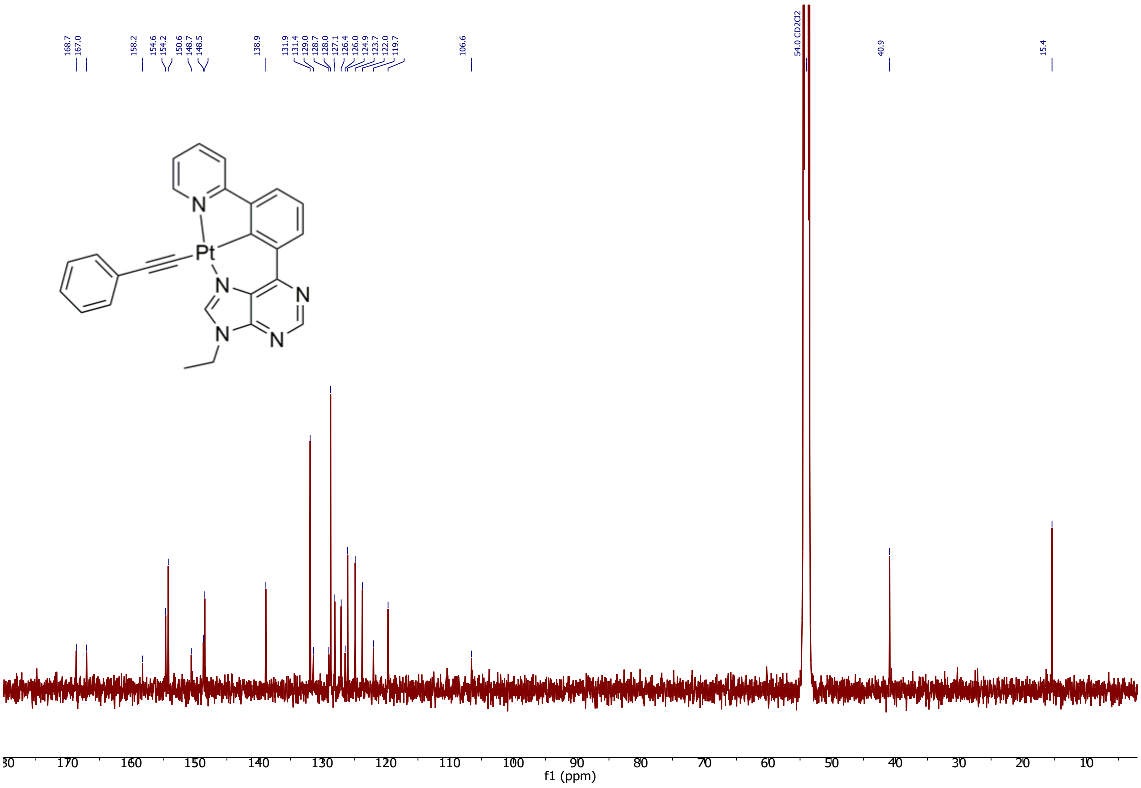

Figure S132. ^{13}C NMR (126 MHz, CD_2Cl_2, 298 K) spectrum of **4c**.

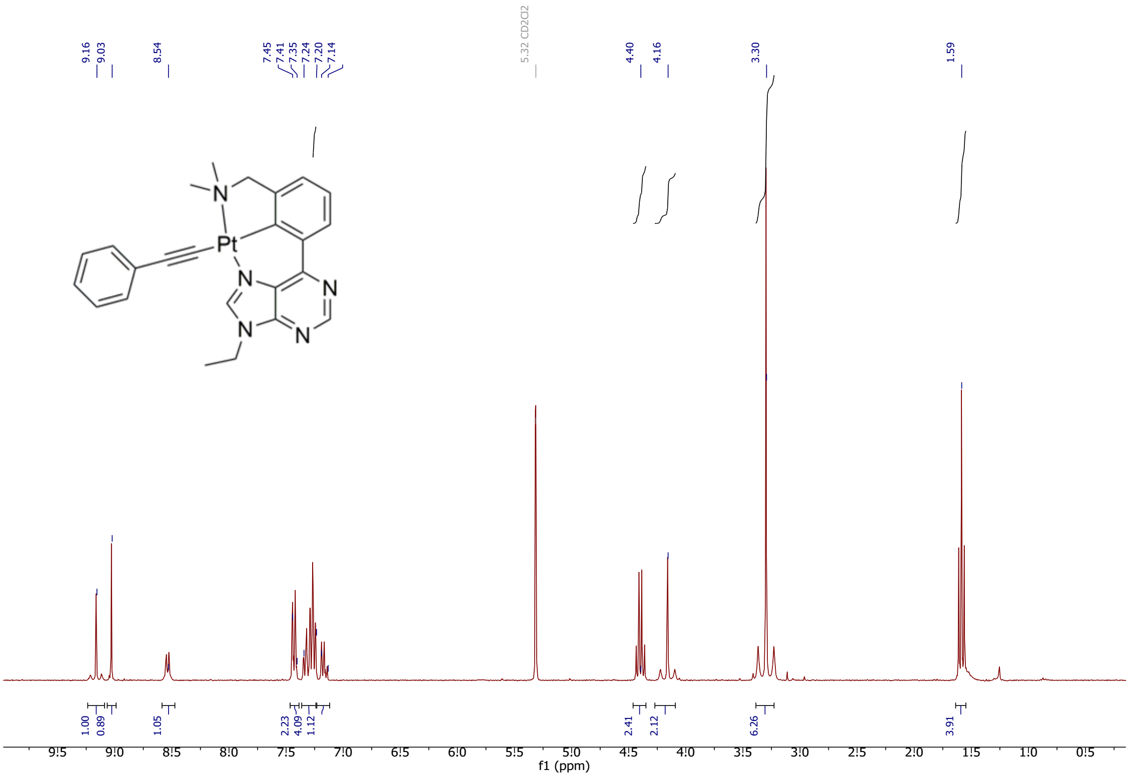

Figure S133. ^{1}H NMR (500 MHz, CD_2Cl_2, 298 K) spectrum of **4d**.

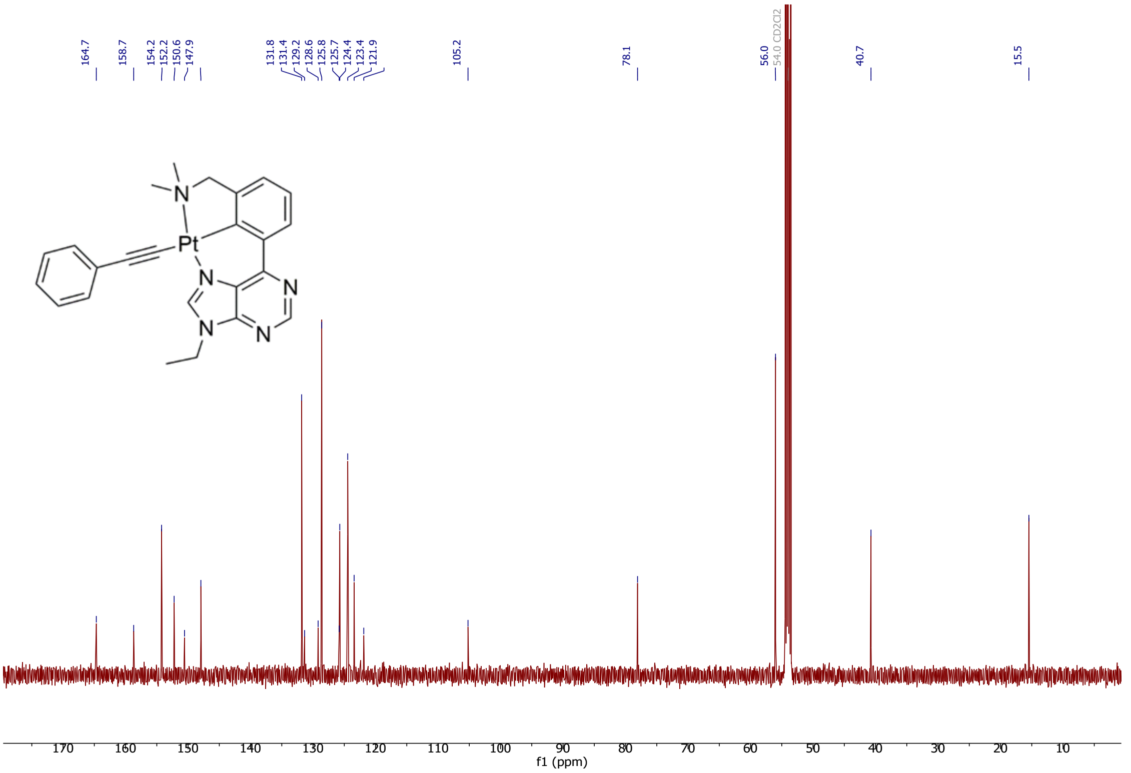

Figure S134. ^{13}C NMR (126 MHz, CD_2Cl_2, 298 K) spectrum of **4d**.

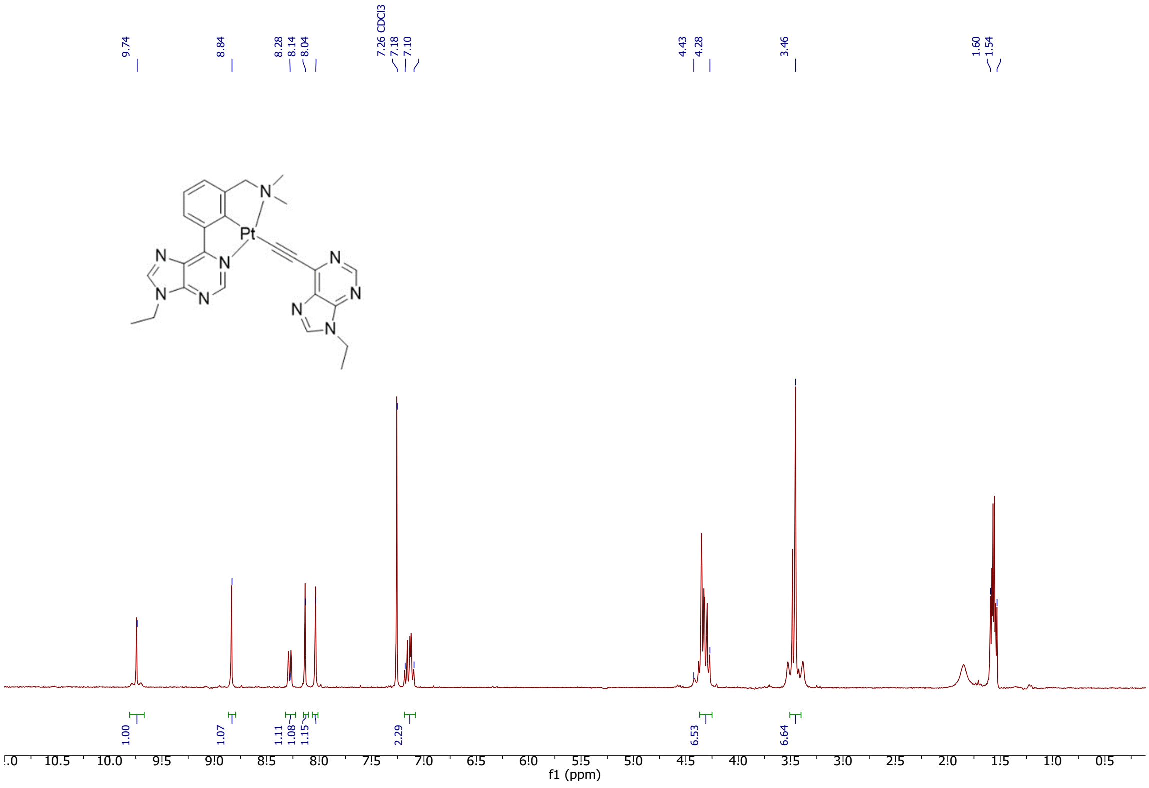

Figure S135. ^{1}H NMR (500 MHz, $CDCl_3$, 298 K) spectrum of **5a**.

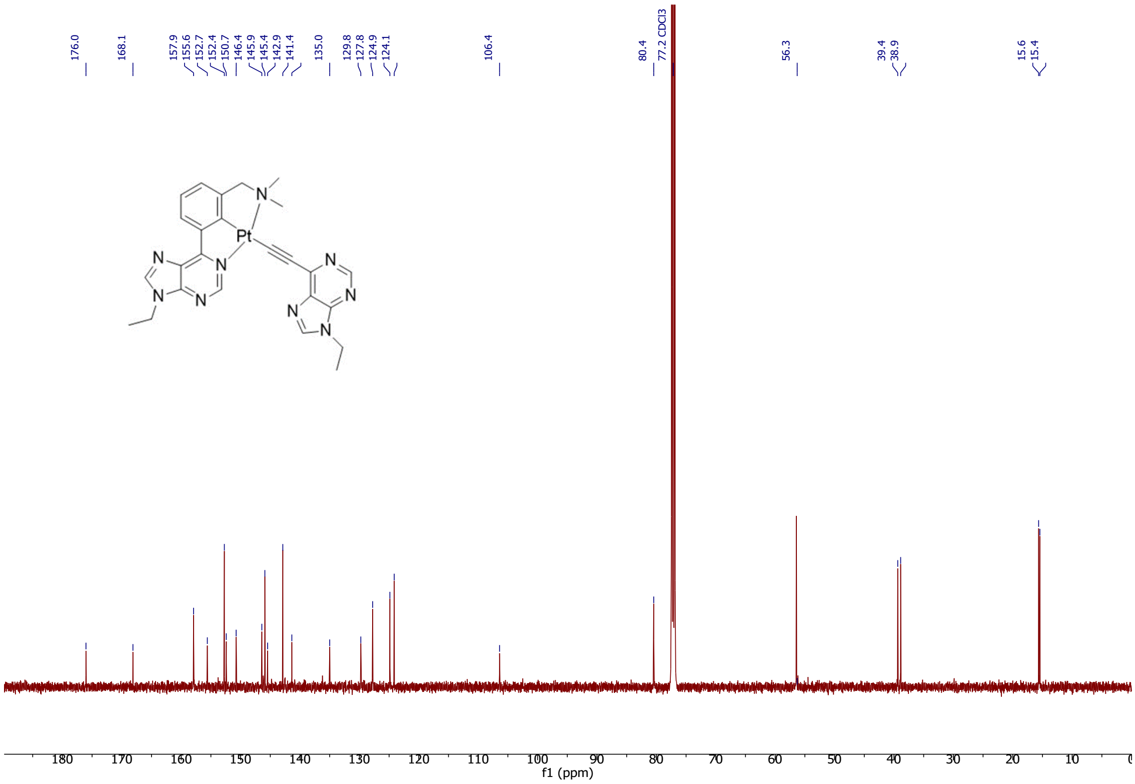

Figure S136. ^{13}C NMR (126 MHz, $CDCl_3$, 298 K) spectrum of **5a**.

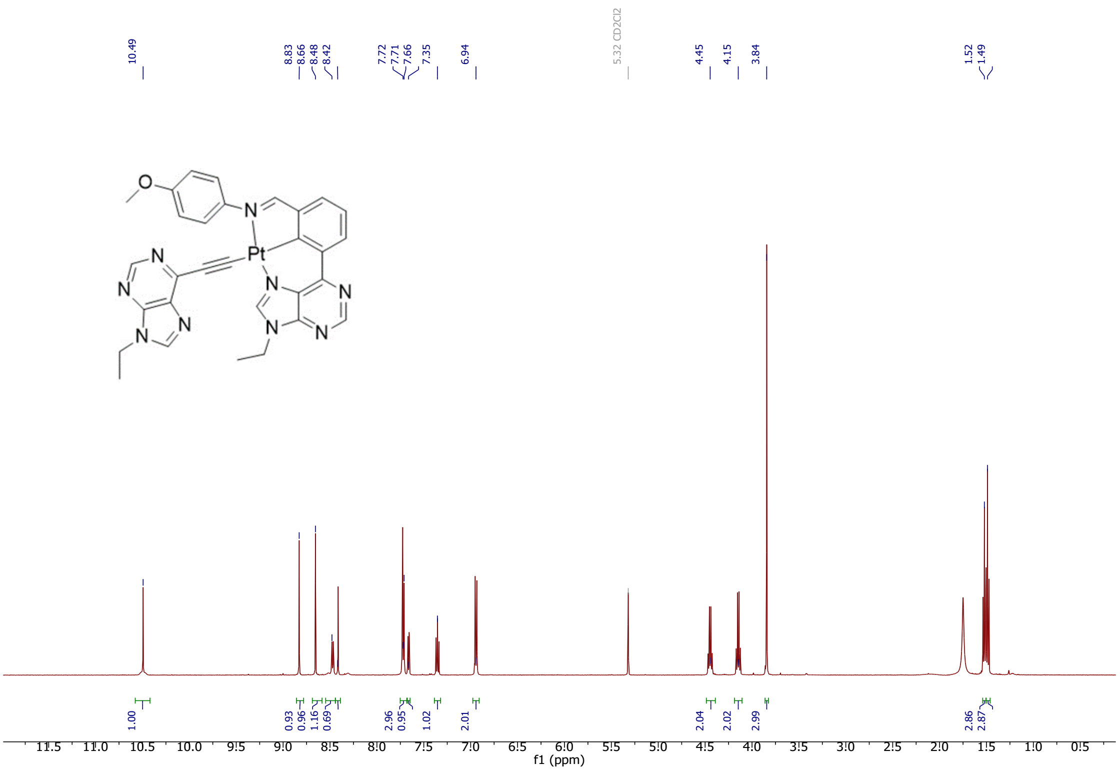

Figure S137. ^{1}H NMR (500 MHz, CD_2Cl_2, 298 K) spectrum of **5b**.

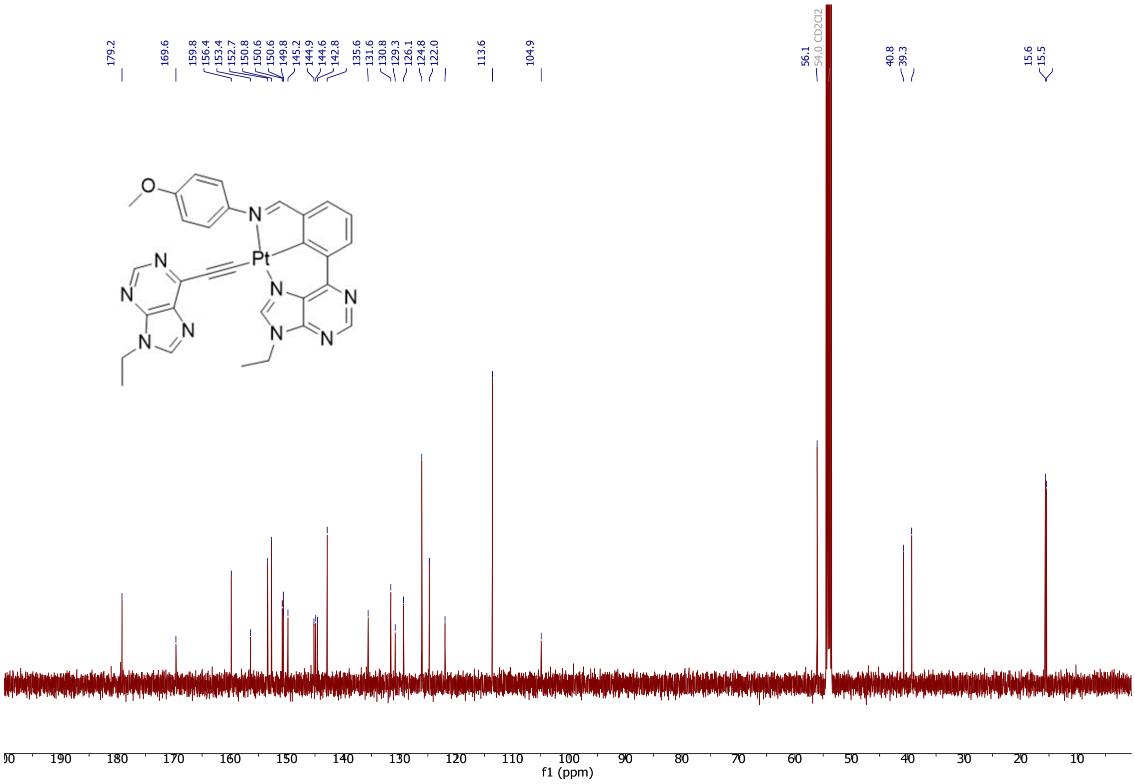

Figure S138. ^{13}C NMR (126 MHz, CD_2Cl_2, 298 K) spectrum of **5b**.

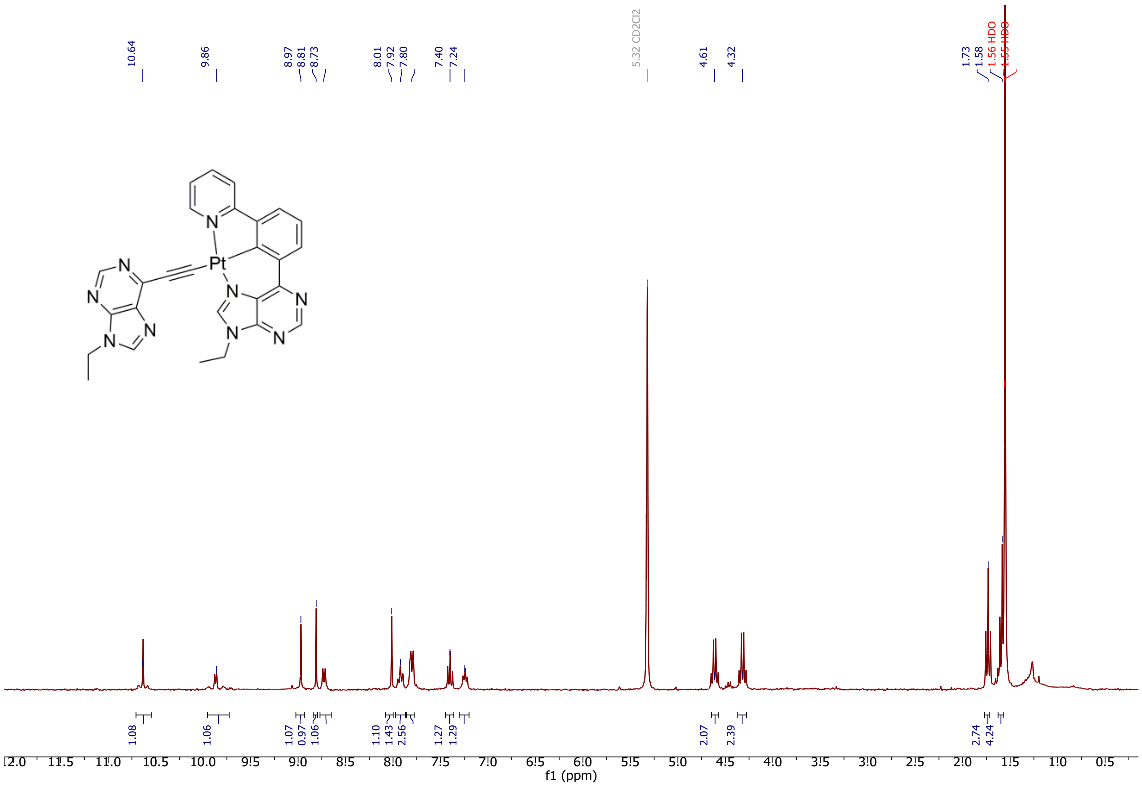

Figure S139. ^{1}H NMR (500 MHz, CD_2Cl_2, 298 K) spectrum of **5c**.

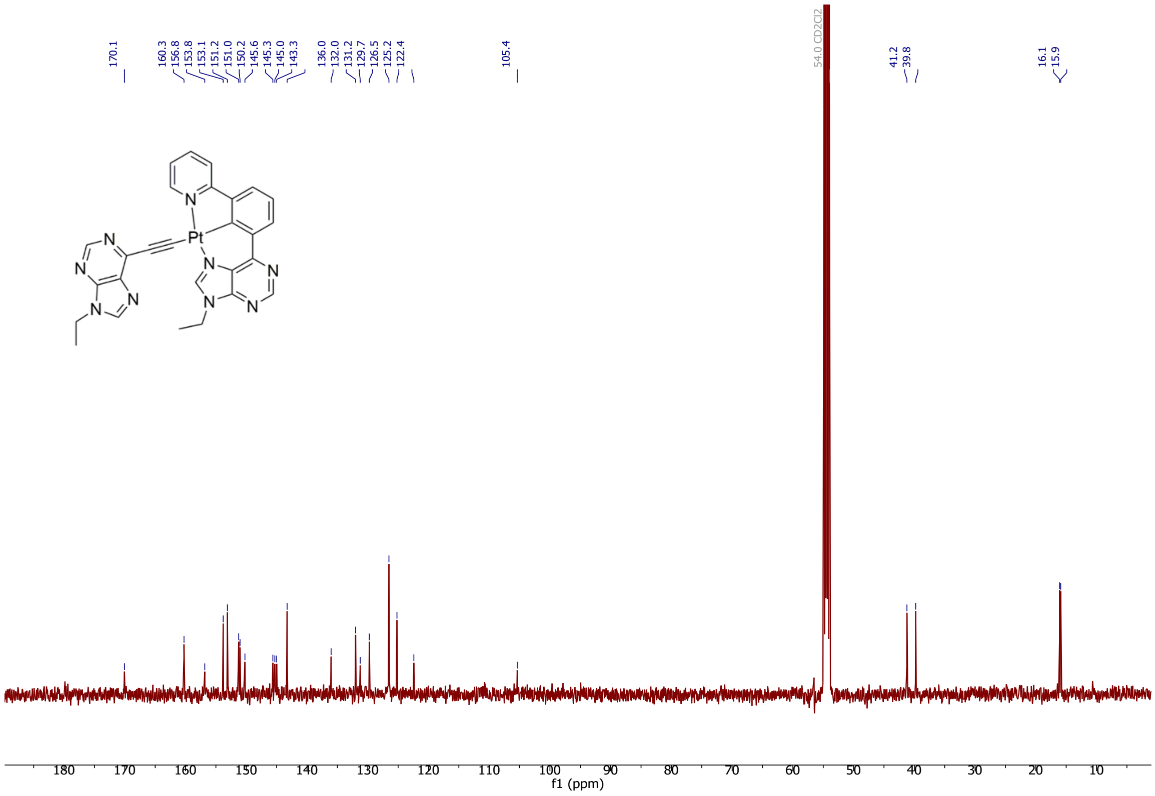

Figure S140. ^{13}C NMR (126 MHz, CD_2Cl_2, 298 K) spectrum of **5c**.

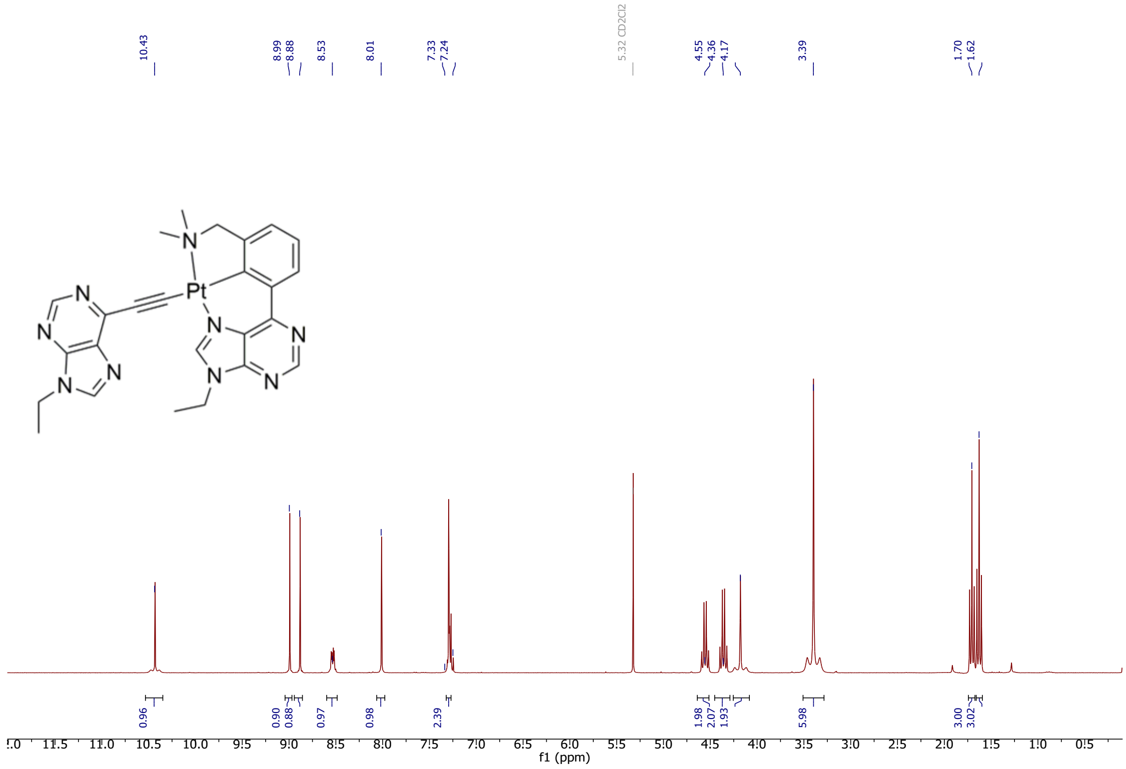

Figure S141. ^{1}H NMR (500 MHz, CD_2Cl_2, 298 K) spectrum of **5d**.

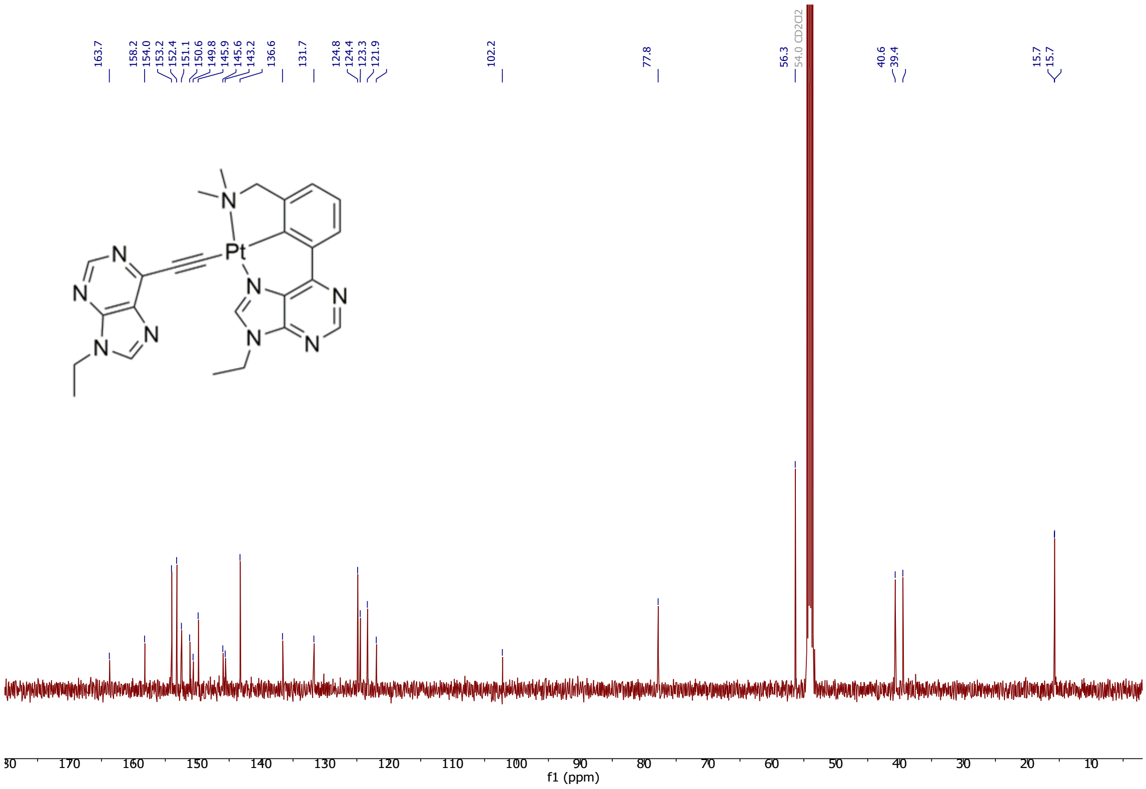

Figure S142. ^{13}C NMR (126 MHz, CD_2Cl_2, 298 K) spectrum of **5d**.

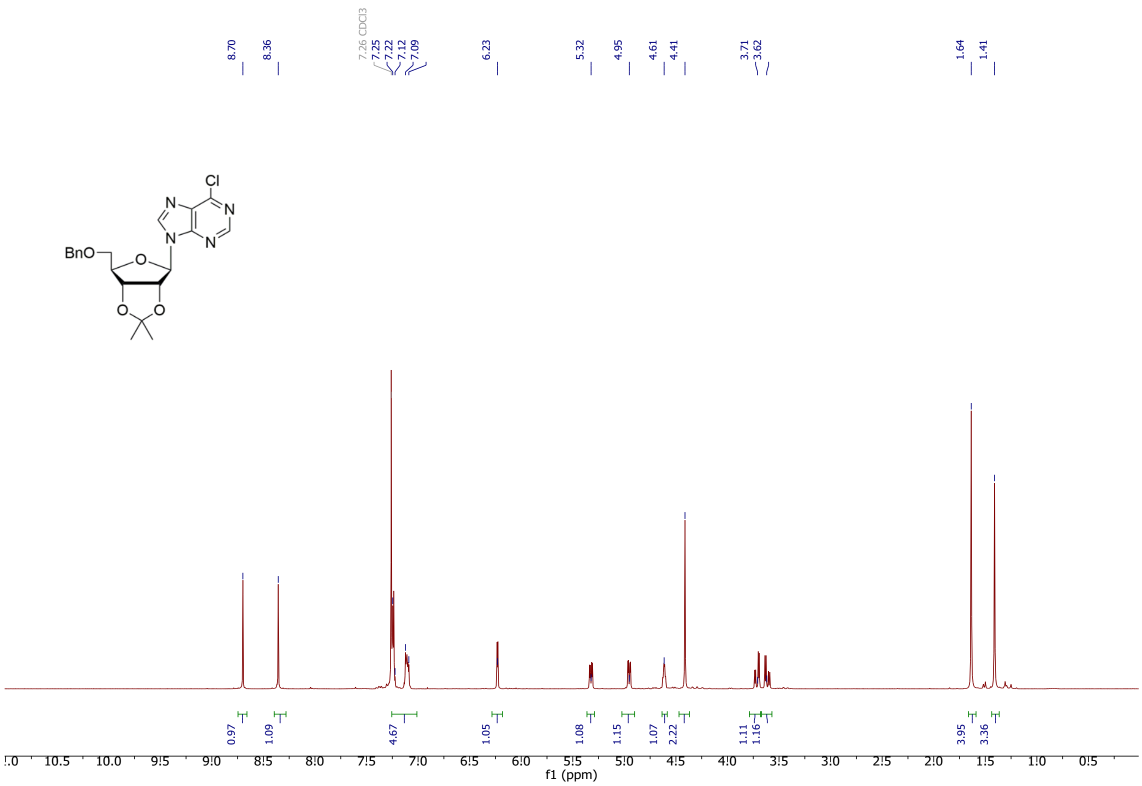

Figure S143. ^{1}H NMR (300 MHz, $CDCl_3$, 298 K) spectrum of **S2**.

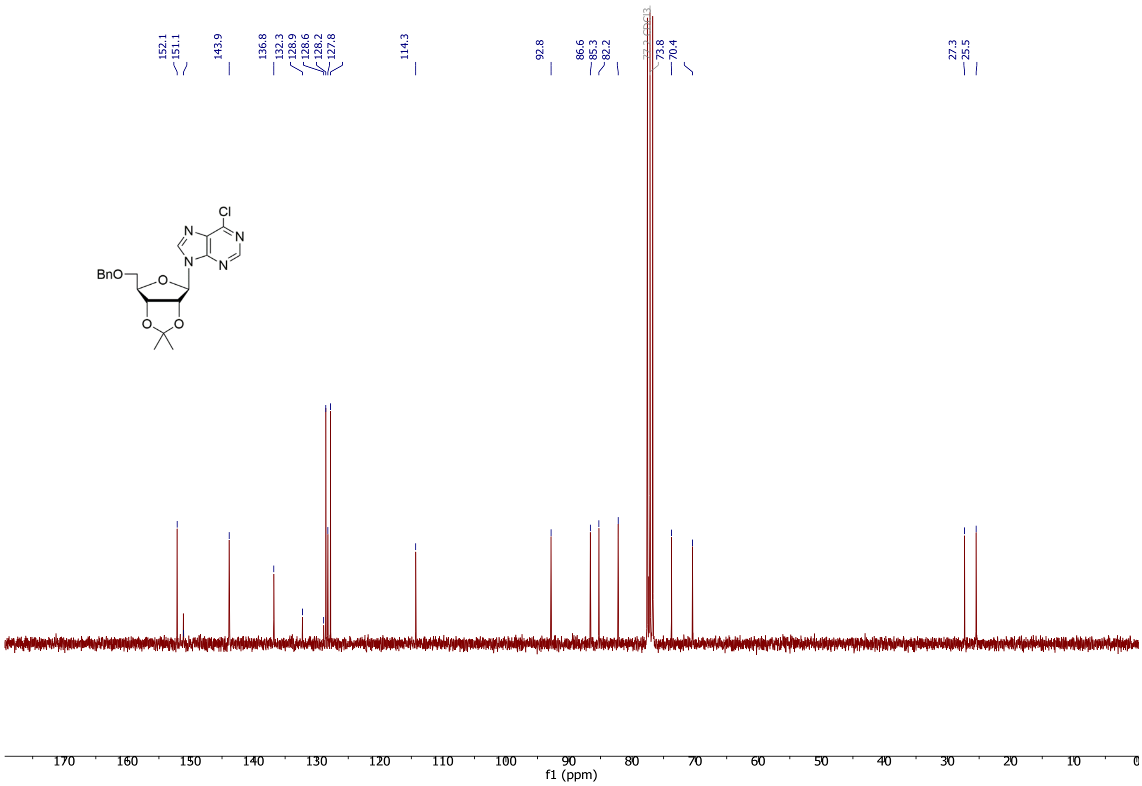

Figure S144. ^{13}C NMR (75 MHz, $CDCl_3$, 298 K) spectrum of **S2**.

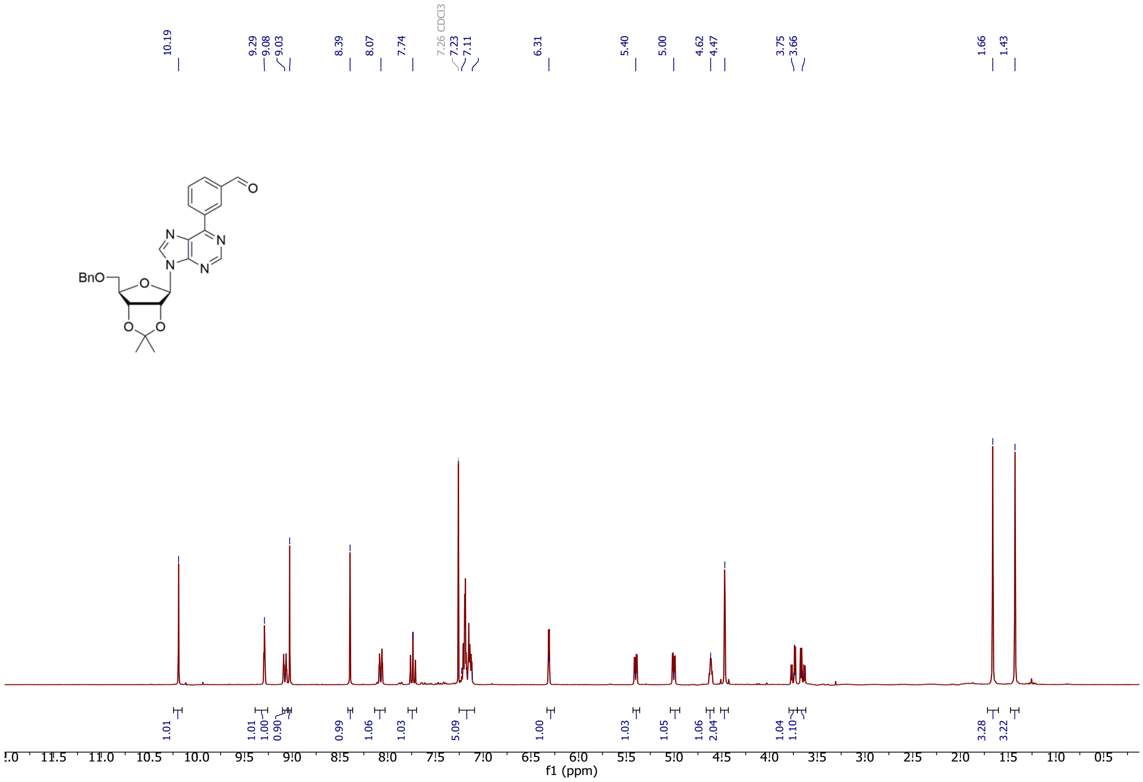

Figure S145. ^{1}H NMR (300 MHz, $CDCl_3$, 298 K) spectrum of **6**.

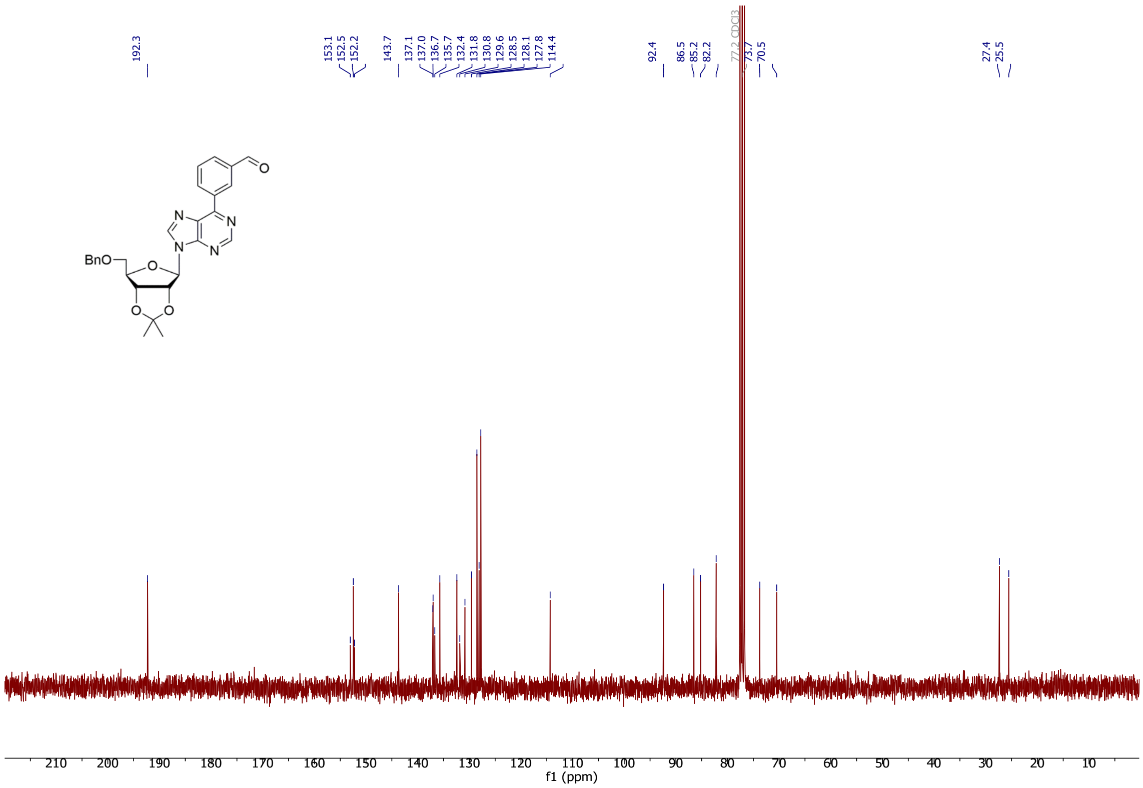

Figure S146. ^{13}C NMR (75 MHz, $CDCl_3$, 298 K) spectrum of **6**.

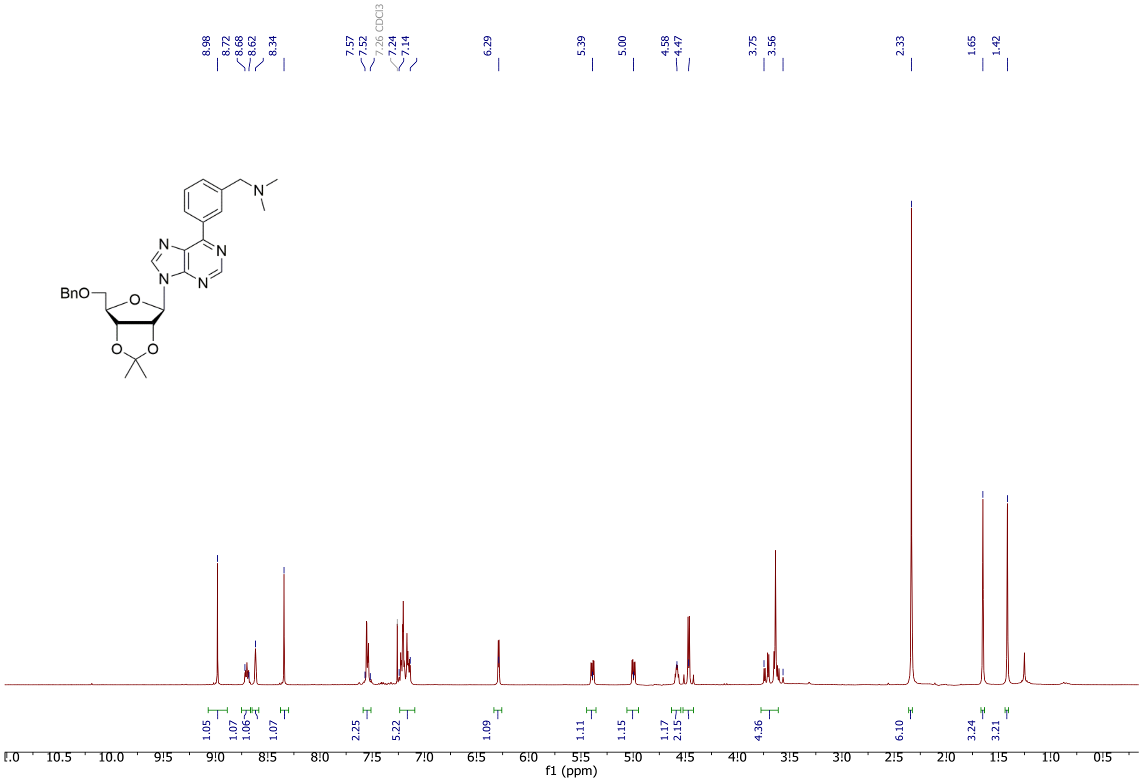

Figure S147. ^{1}H NMR (300 MHz, $CDCl_3$, 298 K) spectrum of **7a**.

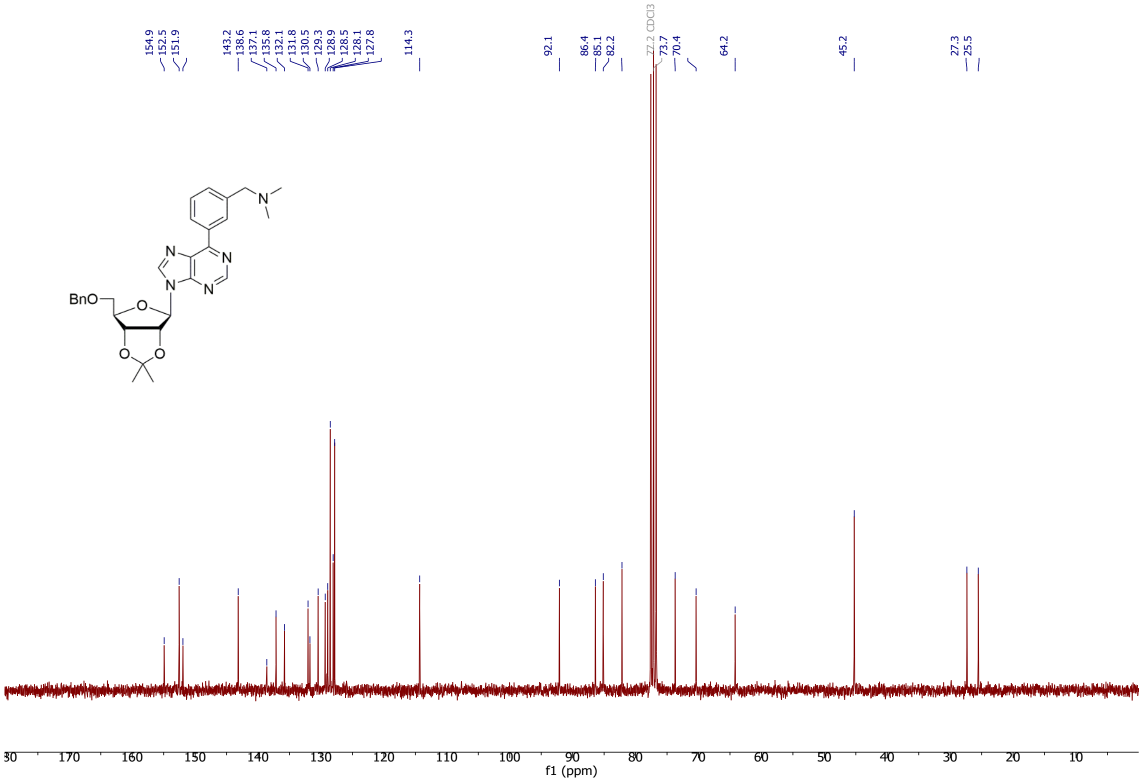

Figure S148. ^{13}C NMR (126 MHz, $CDCl_3$, 298 K) spectrum of **7a**.

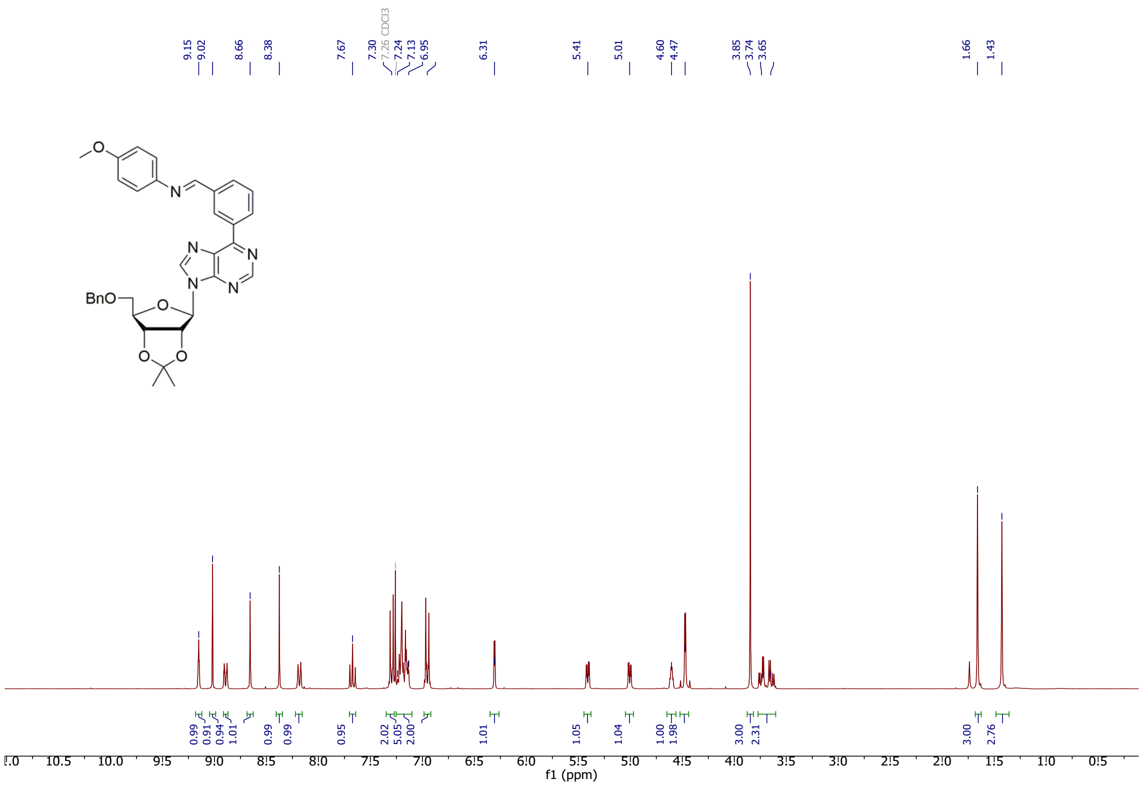

Figure S149. ^{1}H NMR (300 MHz, $CDCl_3$, 298 K) spectrum of **7b**.

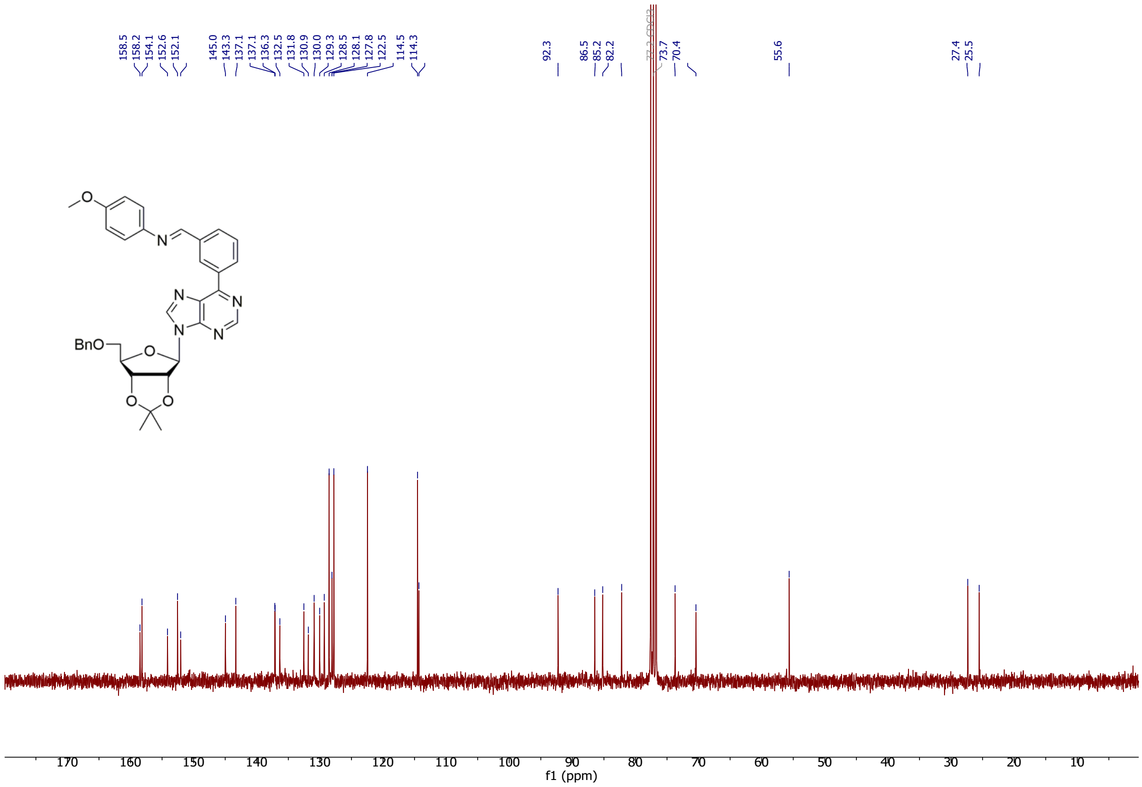

Figure S150. ^{13}C NMR (75 MHz, $CDCl_3$, 298 K) spectrum of **7b**.

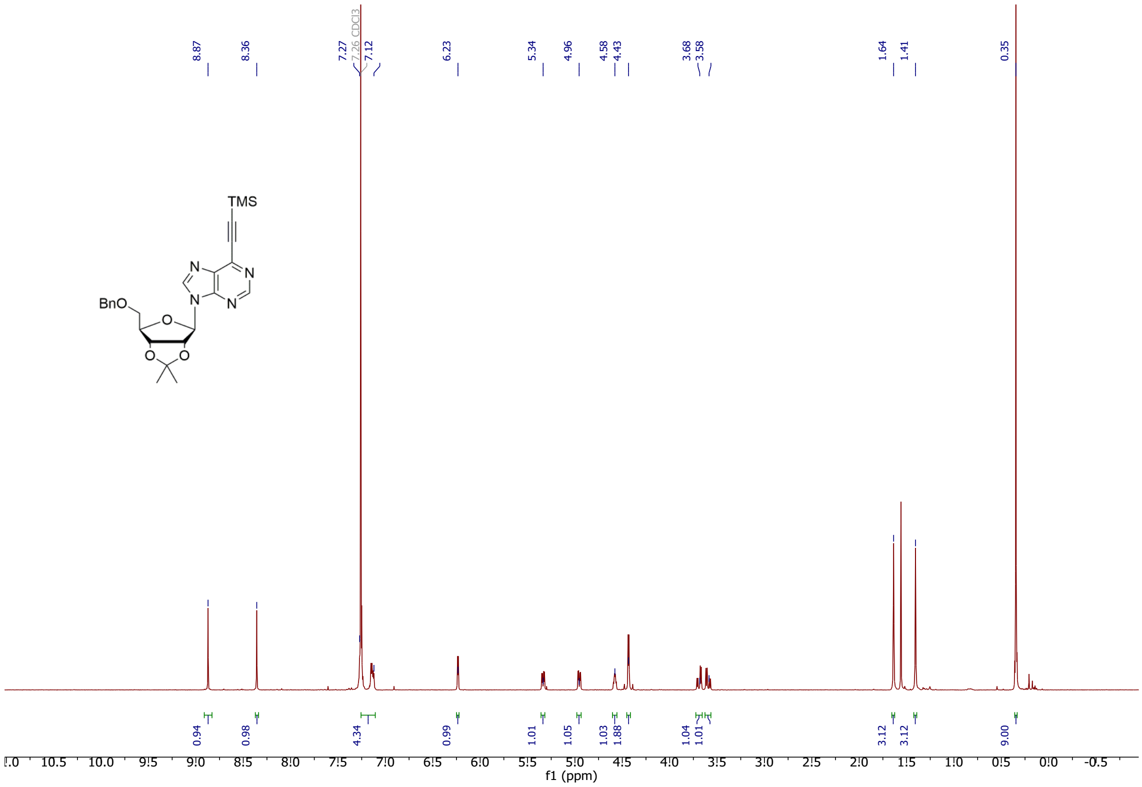

Figure S151. ^{1}H NMR (300 MHz, $CDCl_3$, 298 K) spectrum of **S3**.

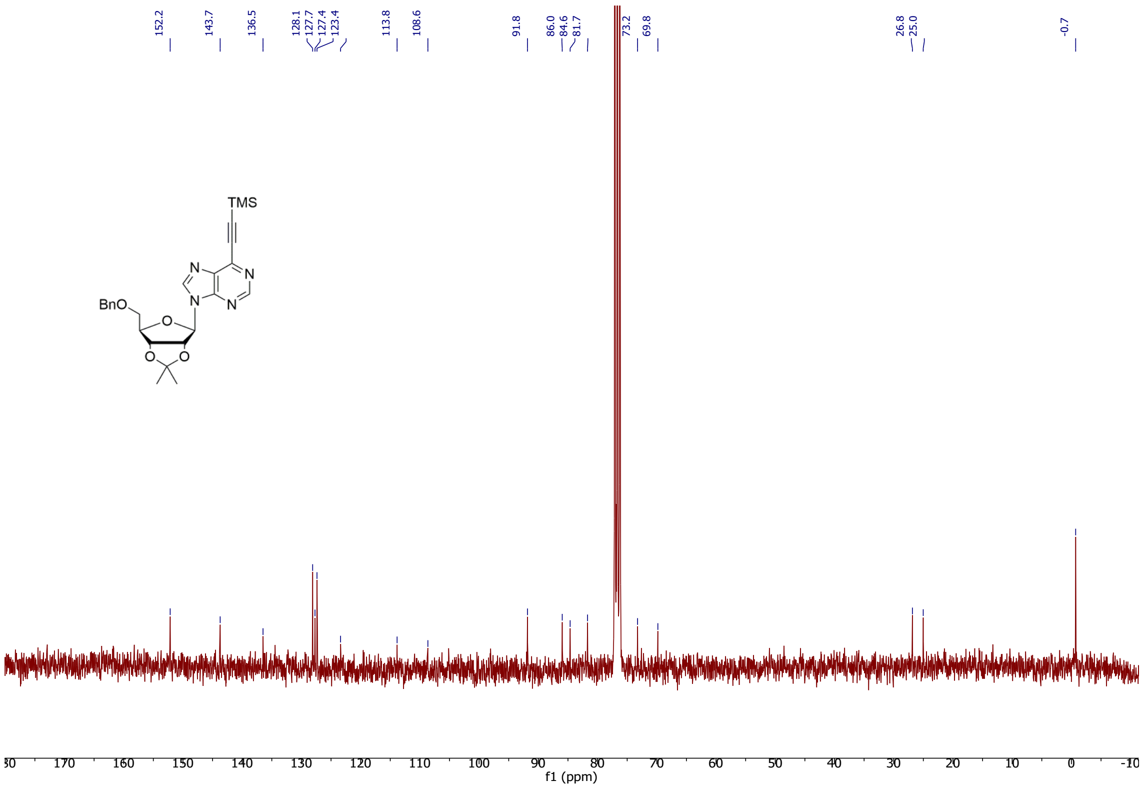

Figure S152. ^{13}C NMR (75 MHz, $CDCl_3$, 298 K) spectrum of **S3**.

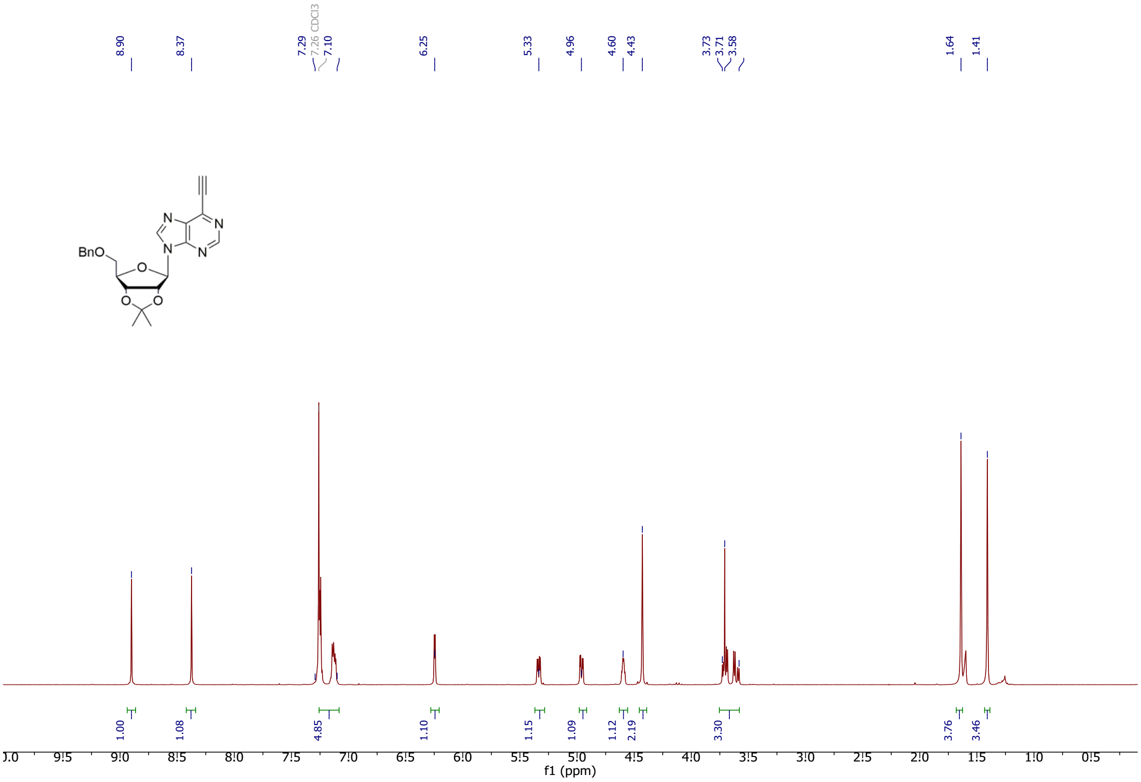

Figure S153. ^{1}H NMR (300 MHz, $CDCl_3$, 298 K) spectrum of **9**.

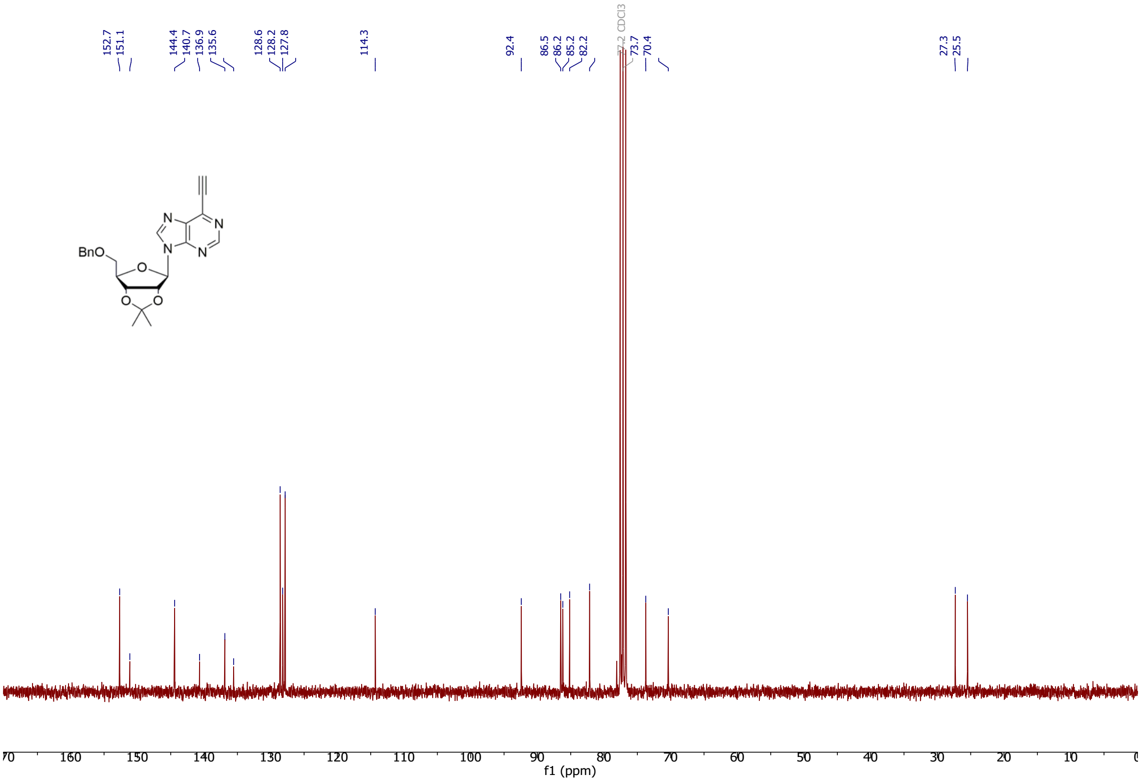

Figure S154. ^{13}C NMR (75 MHz, $CDCl_3$, 298 K) spectrum of **9**.

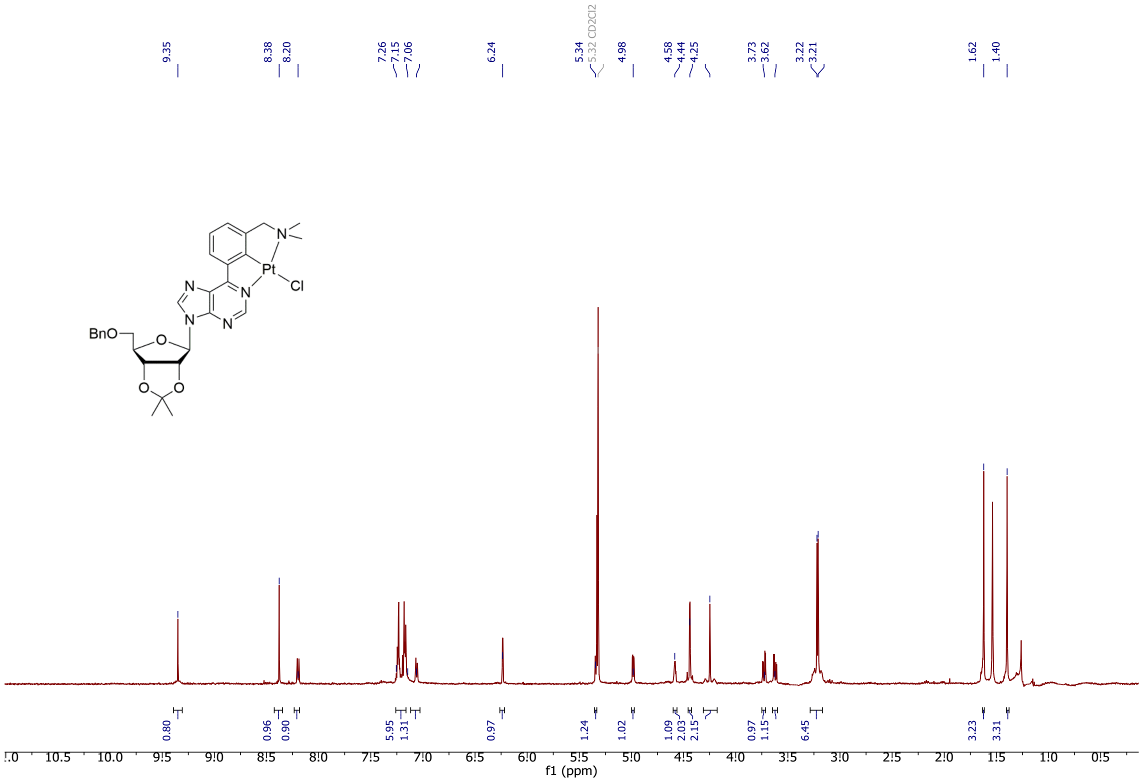

Figure S155. ^{1}H NMR (500 MHz, CD_2Cl_2, 298 K) spectrum of **8a**.

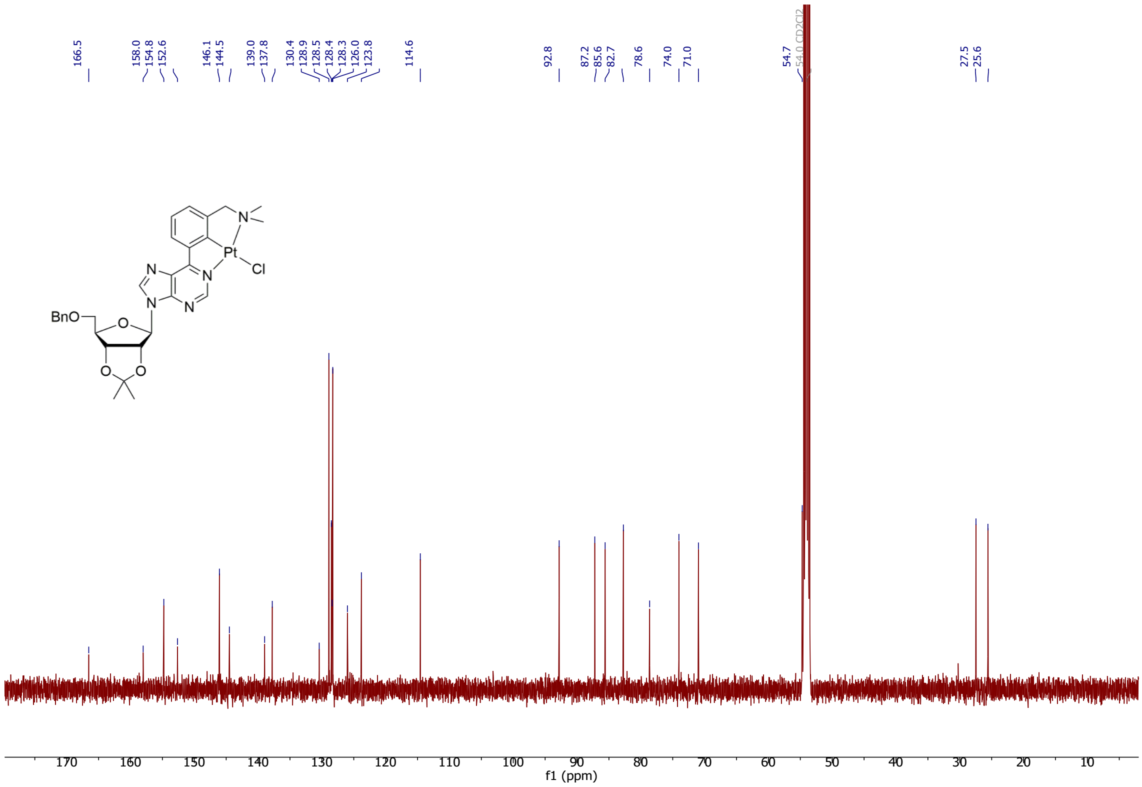

Figure S156. ^{13}C NMR (126 MHz, CD_2Cl_2, 298 K) spectrum of **8a**.

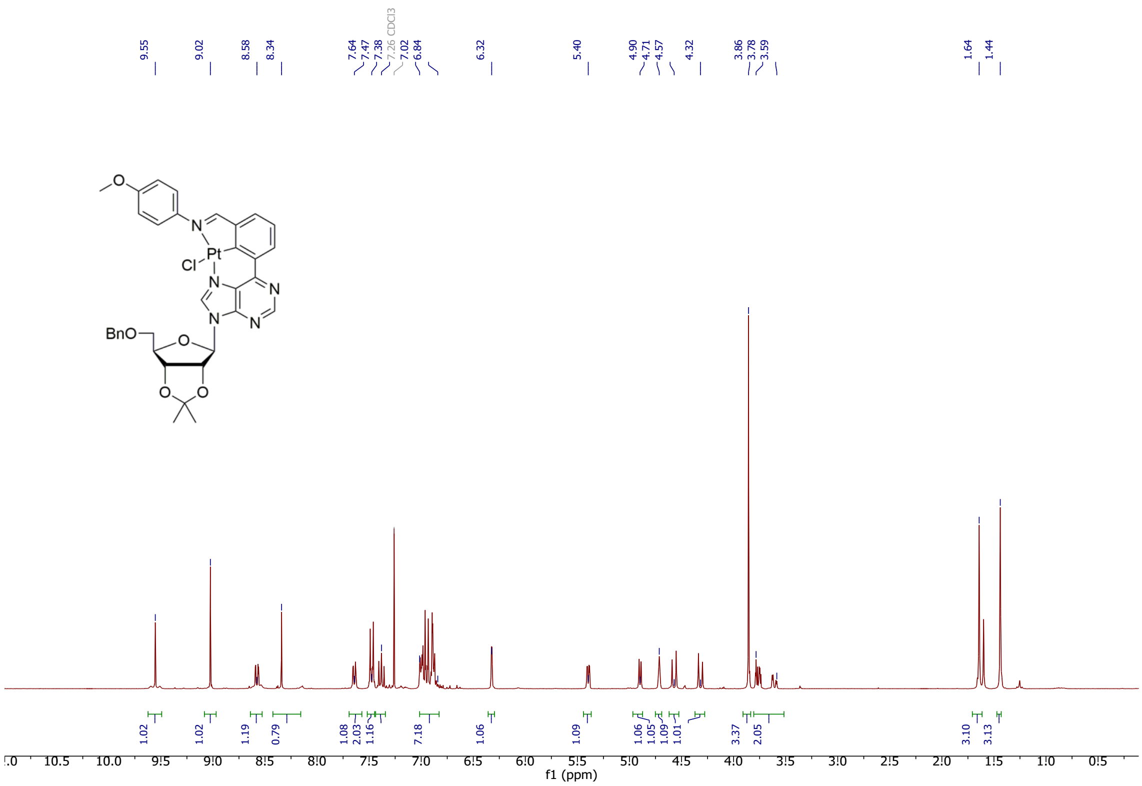

Figure S157. ^{1}H NMR (500 MHz, CD_2Cl_2, 298 K) spectrum of **8b**.

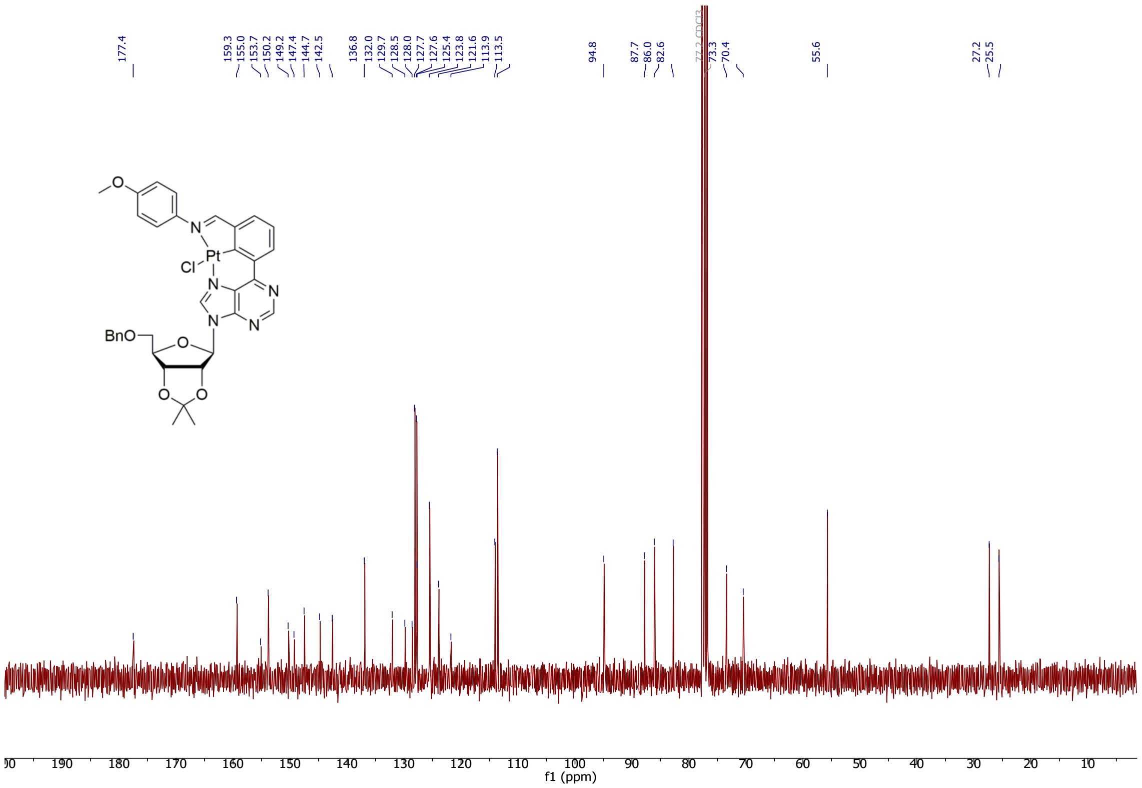

Figure S158. ^{13}C NMR (126 MHz, CD_2Cl_2, 298 K) spectrum of **8b**.

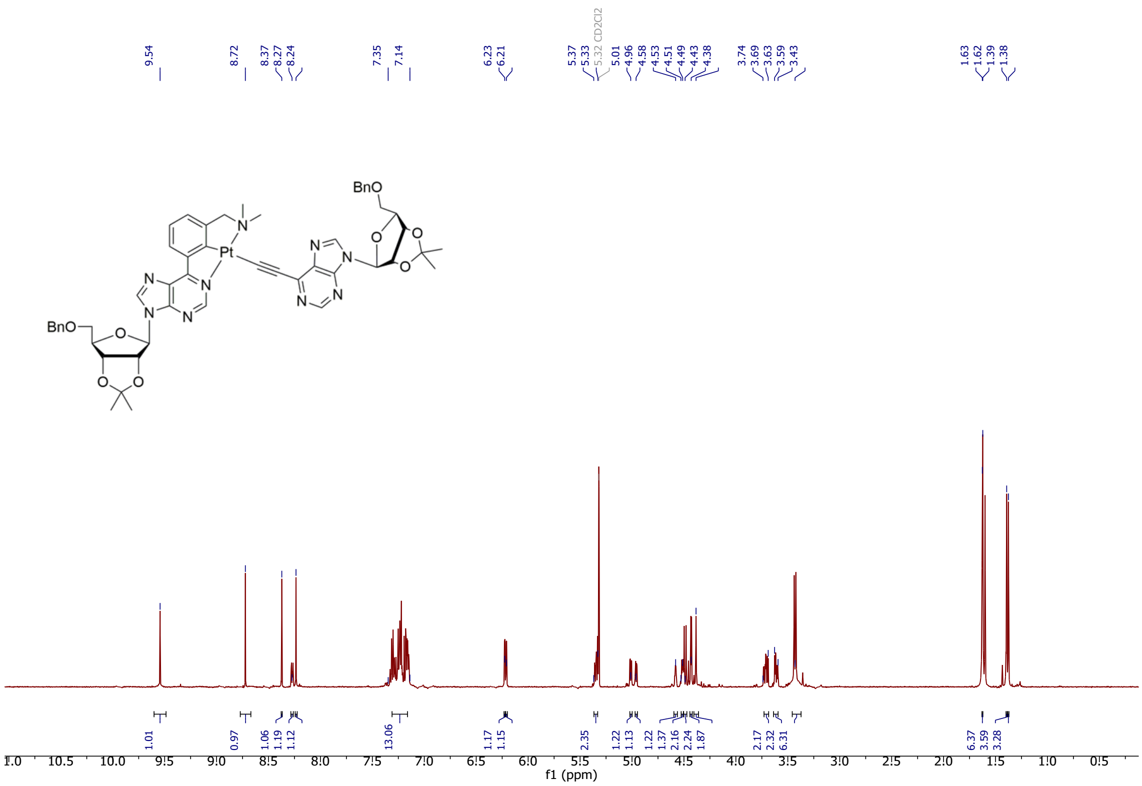

Figure S159. ^{1}H NMR (500 MHz, CD_2Cl_2, 298 K) spectrum of **10a**.

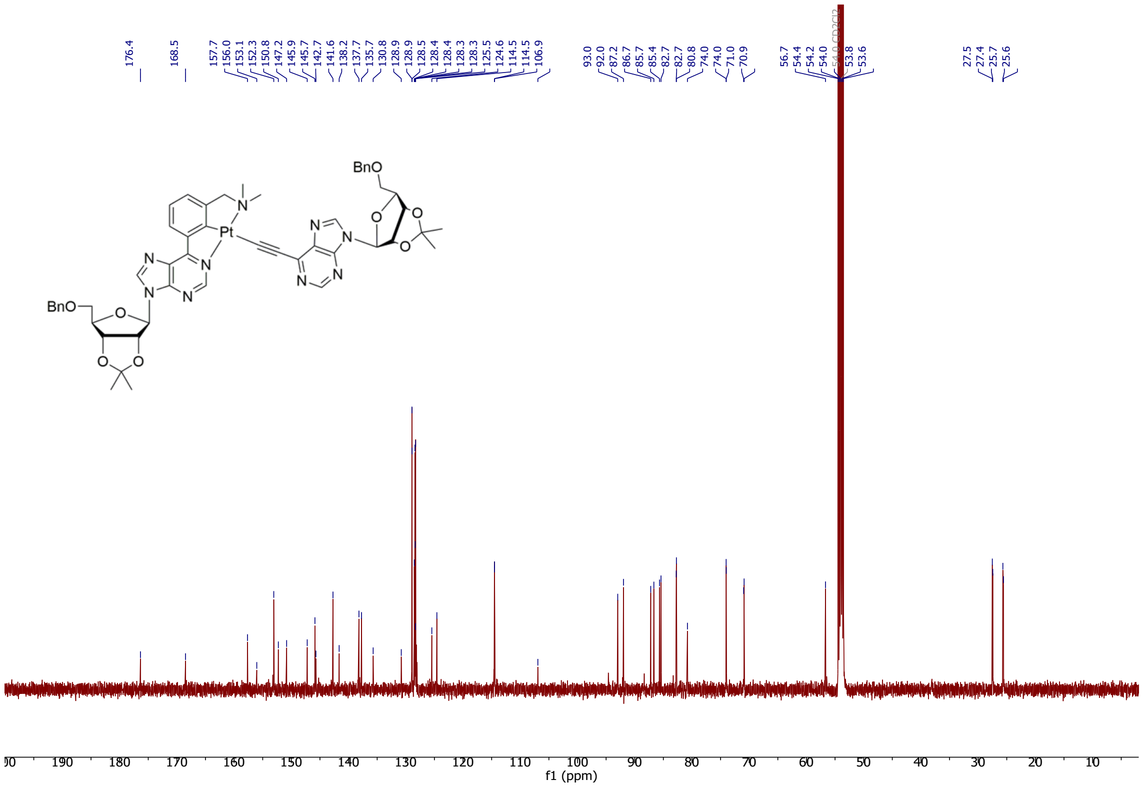

Figure S160. ^{13}C NMR (126 MHz, CD_2Cl_2, 298 K) spectrum of **10a**.

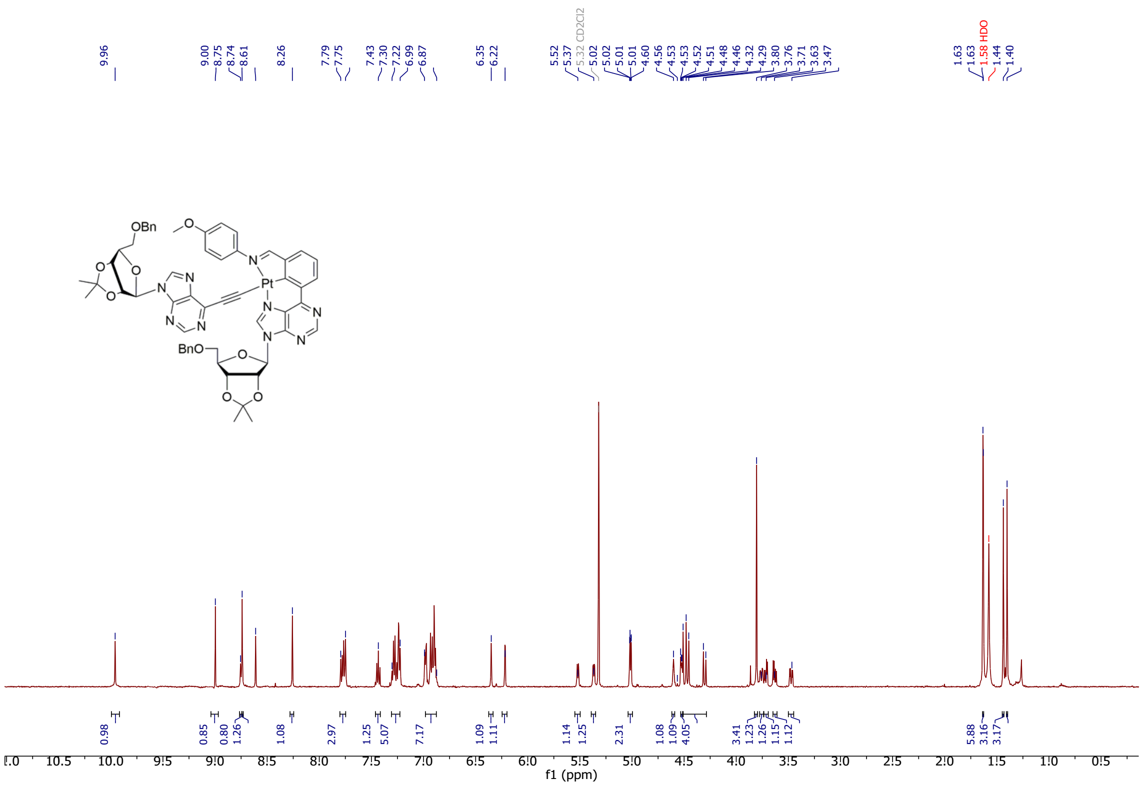

Figure S161. ^{1}H NMR (500 MHz, CD_2Cl_2, 298 K) spectrum of **10b**.

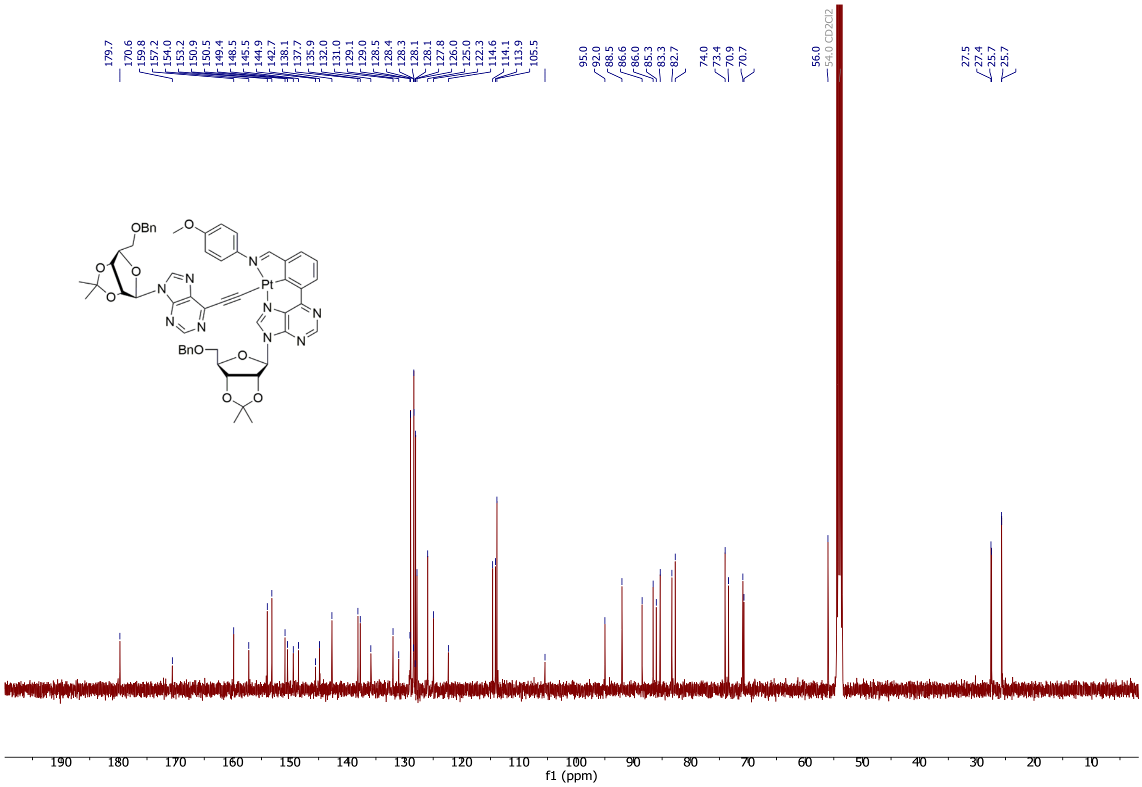

Figure S162. ^{13}C NMR (126 MHz, CD_2Cl_2, 298 K) spectrum of **10b**.

S.8. References

(S1). Chen, J. B.; Liu, E. M.; Chern, T. R.; Yang, C. W.; Lin, C. I.; Huang, N. K.; Lin, Y. L.; Chern, Y.; Lin, J. H.; Fang, J. M. Design and Synthesis of Novel Dual-Action Compounds Targeting the Adenosine A_{2A} Receptor and Adenosine Transporter for Neuroprotection. *ChemMedChem.* **2011**, *6*, 1390-1400.

(S2) a) Blum, T. R.; Miller, Z. D.; Bates, D. M.; Guzei, I. A.; Yoon, T. P. Enantioselective photochemistry through Lewis acid–catalyzed triplet energy transfer. *Science*, **2016**, *354*, 1391–1395.

(S3) Zhao, J.; Brosmer, J. L.; Tang, Q.; Yang, Z.; Houk, K. N.; Diaconescu, P. L.; Kwon, O. Intramolecular Crossed [2+2] Photocycloaddition through Visible Light-Induced Energy Transfer. *J. Am. Chem. Soc.*, **2017**, *139*, 9807–9810

(S4) a) Buzzetti, L.; Crisenza, G. E. M.; Melchiorre, P. Mechanistic Studies in Photocatalysis. *Angew. Chem. Int. Ed.* **2019**, *58*, 373-3747. b) Strieth-Kalthoff, F.; James, M. J.; Teders, M.; Pitzer, L.; Glorius, F. Energy transfer catalysis mediated by visible light: principles, applications, directions. *Chem. Soc. Rev.* **2018,** *47*, 7190-7202.
